# Mechanistically Coupled PK (MCPK) Model to Describe Enzyme Induction and Occupancy Dependent DDI of Dabrafenib Metabolism

**DOI:** 10.3390/pharmaceutics14020310

**Published:** 2022-01-28

**Authors:** Marco Albrecht, Yuri Kogan, Dagmar Kulms, Thomas Sauter

**Affiliations:** 1 Systems Biology Group, Department of Life Science and Medicine, Université du Luxembourg, 4367 Belvaux, Luxembourg; marco.albrecht@esqlabs.com; 2 esqLABS GmbH, 26683 Saterland, Germany; 3 Institute for Medical Biomathematics, Bene Ataroth 6099100, Israel; yuri@imbm.org; 4 Experimental Dermatology, Department of Dermatology, Technical University of Dresden, 01307 Dresden, Germany; dagmar.kulms@uniklinikum-dresden.de

**Keywords:** CYP3A4, dabrafenib, MCPK, PK, DDI, enzyme induction, enzyme kinetics, metabolism

## Abstract

Dabrafenib inhibits the cell proliferation of metastatic melanoma with the oncogenic BRAF(V600)-mutation. However, dabrafenib monotherapy is associated with pERK reactivation, drug resistance, and consequential relapse. A clinical drug-dose determination study shows increased pERK levels upon daily administration of more than 300 mg dabrafenib. To clarify whether such elevated drug concentrations could be reached by long-term drug accumulation, we mechanistically coupled the pharmacokinetics (MCPK) of dabrafenib and its metabolites. The MCPK model is qualitatively based on in vitro and quantitatively on clinical data to describe occupancy-dependent CYP3A4 enzyme induction, accumulation, and drug–drug interaction mechanisms. The prediction suggests an eight-fold increase in the steady-state concentration of potent desmethyl-dabrafenib and its inactive precursor carboxy-dabrafenib within four weeks upon 150 mg b.d. dabrafenib. While it is generally assumed that a higher dose is not critical, we found experimentally that a high physiological dabrafenib concentration fails to induce cell death in embedded 451LU melanoma spheroids.

## 1. Introduction

Melanoma is a cancer type that develops from the pigment-producing melanocytes within the skin. Until recently, metastatic melanoma was considered refractory to treatment with a 3-year survival below 10%. A better understanding of the genetic alterations in metastatic melanoma cells has fundamentally changed systemic therapy and significantly improved the prognosis of patients. The serine-threonine kinase BRAF represents an integral component of the mitogen-activated RAF-MEK-ERK signal transduction pathway [1,2]. Activating mutations of the proto-oncogene BRAF (mutBRAF/wtNRAS, ∼60% of patients) lead to uncontrolled tumor growth [3]. Combinations of mutBRAF inhibitors plus MEK inhibitors are currently accredited in the clinic to treat mutBRAF melanoma, showing a disease control rate of ∼95% and improved median survival [4,5]. However, the vast majority of patients acquire resistance, resulting in tumor relapse.

In this context, a dose determination study revealed higher doses of the mutation-specific BRAF inhibitor dabrafenib to correlate with an increased expression of proliferation markers putatively contributing to tumor relapse. Accordingly, downregulation of ERK phosphorylation (pERK) was shown to be most effective in response to a daily dose of 300 mg dabrafenib, whereas elevated doses of 400 mg and 600 mg, respectively, only presented with reduced pERK inhibition, as determined in human melanoma tissue samples [6]. This observation might be of critical relevance because periodically administered dabrafenib may accumulate in the blood plasma to finally reduce the therapeutically desired pERK inhibition. Dabrafenib itself does not accumulate, but the published dabrafenib pharmacokinetics (PK) model does not include dabrafenib metabolites [6,7]. To include the accumulation potential of dabrafenib metabolites, we extended the current standard PK model of dabrafenib [7] and mechanistically coupled it with the PK (MCPK) model of those metabolites without requiring the detail of physiologically based PK (PBPK) models.

The occurring enzyme induction requires a stringent modeling workflow as the European Medicine Agency (EMA) emphasizes the limited experience in predictive modeling involving enzyme induction and inhibition [8]. Drug metabolism is often described by kinetic enzyme laws such as the Michaelis–Menten kinetics or the kinetic of competitive substrate inhibition [9]. The latter has been used to describe drug–drug-interactions (DDI) between dabrafenib and the anti-fungal drug ketoconazole [10]. However, kinetic enzyme laws are simplifications of reaction network models based on the assumption that enzyme levels remain constant and such assumptions need to remain valid in subsequent use [11]. Consequently, we will use enzyme kinetic laws only for enzymes with a steady total amount and use reaction network modeling otherwise.

This dabrafenib study aims to better understand dabrafenib dose-related tumor relapse by modeling the PK of metabolites [12]. The PK of each metabolite will be intertwined by a biochemical reaction network, which also integrates both enzyme induction and DDI. The MCPK model predicts an accumulation of dabrafenib metabolites, while our experiments with 451LU melanoma spheroids show that an elevated dabrafenib dose fails to induce cell death.

## 2. Materials and Methods

### 2.1. Modeling Methods

**Figure 1:**
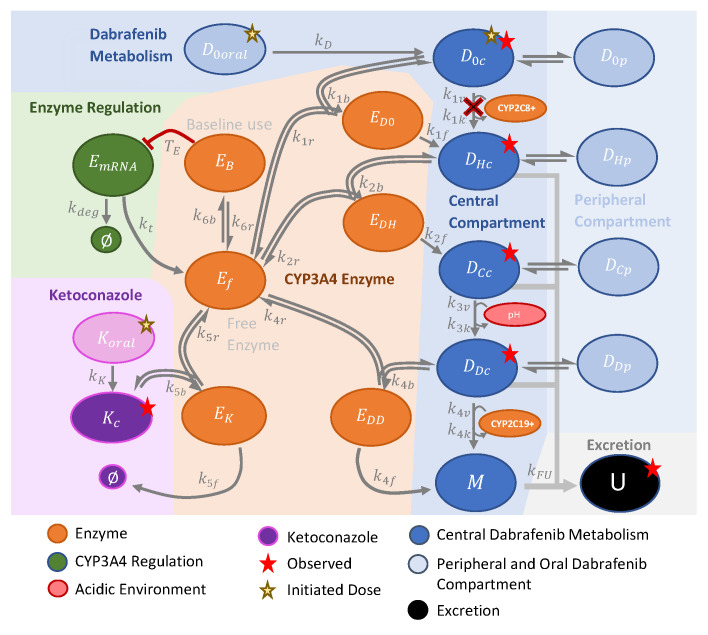
The graphical representation of the model shows the structure of the mathematical equations in the Appendix A. The colored background dissects the model into the blue dabrafenib and purple ketoconazole part, which are connected by the orange area to describe CYP3A4 interactions. The green area describes enzyme regulation, while the grey area highlights excretion U (feces and urine) of dabrafenib metabolites, which distribute in the central (blue, index c) and peripheral compartments (light blue, index p). Dabrafenib, with index 0, appears separately *D*_0_*_oral_* (drug solution) and as part of the central compartment *D*_0_ (solid formulation). It is metabolized to hydroxy-dabrafenib *D_H_* by CYP3A4 but according to sensitivity analysis not by the enzyme pool CYP2C8+ (crossed, in vitro contribution: 56% CYP2C8, 10% CYP2C9, 6.9% CYP2B6, 1.9% CYP1A2 [13]). Hydroxy-dabrafenib is converted by CYP3A4 to carbo-dabrafenib *D_C_*. A low pH environment converts carbo-dabrafenib into desmethyl-dabrafenib *D_D_* being further degraded to metabolites M by CYP3A4 and the enzyme pool CYP2C19+ (in vitro contribution: 22% CYP2C19, 9.6% CYP2C9 [13]). Chemical formulas of dabrafenib metabolites are in Section S2 in Supplemental Information S1. Oral ketoconazole *K_oral_* distributes centrally *K_c_* and forms with the free CYP3A4 enzyme *E_f_* a complex *E_K_* that clears ketoconazole Ø. CYP3A4 builds the enzyme complex *E_B_* to meet baseline requirements, whereby the abundance needs to exceed the threshold *T_E_*. If not, CYP3A4 mRNA *E_mRNA_* is released and translated into CYP3A4 protein with rate *k_t_* until mRNA is depleted (degradation rate *k_deg_*).

The original two-compartment model [7] was reproduced (Section S1 in Supplemental Information S1), and the phenomenological time and dose-dependent clearance terms were replaced by a reaction network model with enzyme and pH-dependent metabolic changes as specified in Figure 1. CYP3A4 builds enzyme-substrate complexes not only with dabrafenib metabolites but also with ketoconazole. The ketoconazole PK is set with a simple compartment model. The enzyme CYP3A4 is modeled as a biochemical reaction network and not as simplified enzyme kinetic, which has two advantages. First, enzyme induction upon enzyme deprivation can be modeled as a regulatory loop. Second, the biochemical reaction network allows the competitive binding of CYP3A4 by dabrafenib and ketoconazole with a DDI effect based on binding capacity.

While the proposed model structure is based on in vitro and pre-clinical experiments, MCPK model parameter values cannot be based on such data. To quantitatively integrate pre-clinical data, a parameter-rich physiologically based pharmacokinetic (PBPK) model is required to provide the spatial and experimental context, which is beyond the scope of this article and its research question. Instead, solely clinical datasets on dabrafenib, ketoconazole, and dabrafenib-ketoconazole interaction [7,14,15,16] were used to determine parameters as in standard PK.

**Figure 2:**
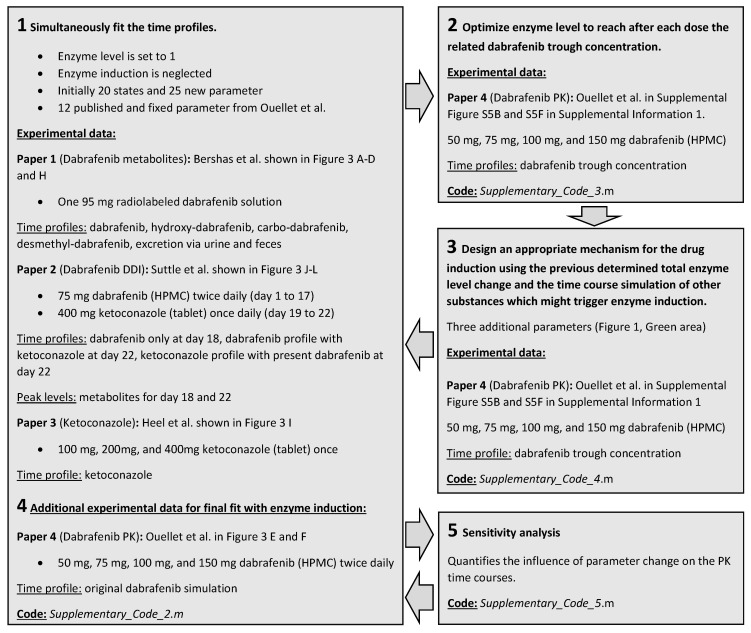
MCPK model parametrization and refinement: 1: Model with constant enzyme levels is fitted to dabrafenib metabolism alone [14], ketoconazole PK alone [15], and DDI [16]. 2: Obtained parameters remain fixed, while the CYP3A4 levels are varied until published trough levels are met [7]. 3: The enzyme level profile is used as input for the design of an enzyme induction model. 4: Main and enzyme induction models are combined, and all parameters are refined. 5: Sensitivity analysis identifies irrelevant model parts.

A workflow (Figure 2) was established to model the enzyme level changes effectively. First, the model fitted the data sets without considering enzyme induction (Figures S3 and S4 in Supplemental Information S1). In step two, parameters are fixed, and the total enzyme concentration is optimized on each dose interval separately to align the model with the reported trough concentration. The temporal change of total enzyme concentration serves as orientation for the next step (Figure S5 in Supplemental Information S1). In step three, the model structure of enzyme induction is designed so that the right amount of total enzyme is released. In step four, the complete model with the enzyme induction motif was refitted to finalize the modeling procedure. During model development, sensitivity analysis was performed to identify unnecessary parameters and model structures (Section S3 in Supplemental Information S1). Following sensitivity analysis, the transformation of dabrafenib to hydroxy-dabrafenib via CYP2C8 was found negligible and removed from the model.

The model is based on ordinary differential equations (ODE), implemented in MATLAB^®^ R2017b with the global optimization toolbox, solved with the stiff variable-step and variable-order (VSVO) solver ode15s, and optimized with either the genetic algorithm *ga* or the single variable optimizer *fminbnd*. The sensitivity analysis can be found Section S8 in Supplemental Information S1 and covers both the simulated dynamic and the overall agreement between data and model via the residual sum of squares (RSS).

### 2.2. Cells and Reagents

Human melanoma cell line 451LU was obtained from the American Type Culture Collection (ATCC) and maintained in RPMI 1640/Glutamax medium (Live Technologies, Karlsruhe, Germany) with 10% FCS (Thermo Scientific, Langenselbold, Germany) and 1% penicillin/streptomycin (Life Technologies) in a humified atmosphere of 5% CO${}_{2}$ at 37 °C. Cells were tested every other month to be mycoplasma-negative as judged by the MocyAlert Mycoplasma Detection Kit (LT-07, Lonza, Basel, Switzerland). The mutation-specific BRAF inhibitor dabrafenib (#S2807; CAS: 1195765-45-7) was purchased (Selleckchem, Munich, Germany) and applied to cells at different doses for the indicated time points.

### 2.3. Hydrogel

Dextran was used as the thiol-reactive polymer that crosslinks with the polyethylene glycol peptide conjugate (CD-Link). The linker can be cleaved by the secreted matrix-metalloproteases MMP1, MMP3, MMP7, and MMP9 at the peptide-motif Pro-Leu-Gly-Leu-Trp-Ala, which enables cells to spread and migrate throughout the gel [17]. More specifically, the slow gelling 3-D Life dextran-CD hydrogel set (#G93-1; Cellendes, Reutlingen, Germany) was prepared and supplemented with fibronectin (Sigma Aldrich, Taufkrichen, Germany). We consecutively mixed water, 10XCB pH 7.2, dextran, fibronectin (human fibroblasts, 0.5 mg/mL), RPMI, CD-linker with the pipetted volumes for the soft gel (14.8, 2,4, 2, 1.8, 6 and 3 μL) and the hard gel (5.7, 2,5, 6, 1.8, 5 and 9 μL). To determine the shear modulus of the soft and hard gel, 67 μL gel solution was first polymerized between hydrophobic glass slices (9 mm diameter, Sigmacode) for 60 min at RT and then swollen to equilibrium in RPMI medium overnight. The height of the cylindric gel discs varied between 0.774 mm and 1.260 mm after they were punched out to a diameter of 8 mm. An Ares LN2 rheometer (TA Instruments, Eschborn, Germany) with an 8 mm parallel plate geometry applied a rotational strain of 5% and compression strain of 10% to perform a frequency sweep over a range of 0.01 to 100 rad/s (Section S6 in Supplemental Information S1 and raw data in Supplemental Information S7).

### 2.4. 3D Melanoma Spheroids

Melanoma spheroids were generated using the ’hanging drop’ method [18]. Briefly, 250 GFP-expressing 451LU cells were resuspended in 25 μL of RPMI containing 20% methocell and individual drops were spotted on the inside of a lid belonging to a 10 cm cell culture dish. The lid was inverted onto the dish filled with 10 mL of 1X PBS before incubating for 7 days at 37 °C with 5% CO${}_{2}$. Each mature spheroid was injected into 30 μL gel-matrix, incubated for 30 min at 37 °C, and covered with medium. The medium was replaced all two days and contained dabrafenib at day four to six. The development of individual spheroids was monitored at injection day, day four, and day six by confocal fluorescence microscopy (LSM 780/FCS inverse, Zeiss, Germany) equipped with a Plan-Apochromat 10×/0.45 M27 objective. For the emitted green fluorescent, the laser emission peak was 488 nm (emission filter 499-597 nm). Z-stacks were taken to quantify the spheroid area (Section S6 in Supplemental Information S1). Spheroid areas at day 4 were normalized to areas at day 0, and areas at day 6 were normalized to areas at day 4. Statistical significance was determined using Welch’s *t*-test implemented in the R-package *ggsignif* (Supplementary-Code-6.r). Z-stacks were processed in one batch using Fiji code (https://fiji.sc (accessed on 30 May 2017); Supplementary Information S1).

## 3. Results

### 3.1. The Model Reproduces Data

The model is shown in Figure 1 to visualize the employed ordinary differential Equations (ODEs).

**Figure 3:**
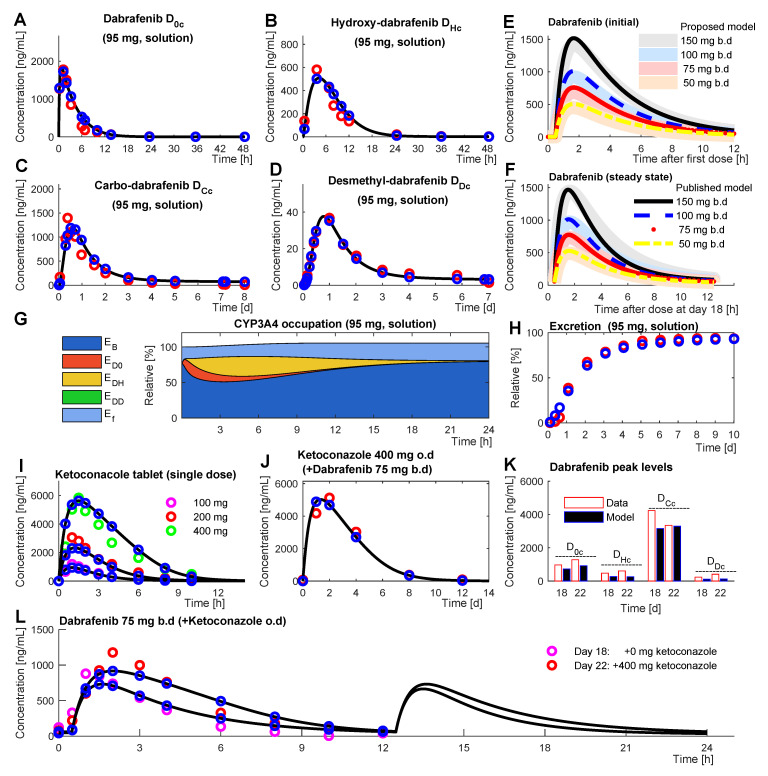
Final simulation result (black line and blue circles) aligns with data (circles of different color) in the profiles of dabrafenib $D_{0c}$ (**A**), hydroxy-dabrafenib $D_{Hc}$ (**B**), carbo-dabrafenib $D_{Cc}$ (**C**), desmethyl-dabrafenib (**D**), and relative excretion (**H**) upon single administered of 95 mg [${}^{14}$C]-dabrafenib solution [14]. The related enzyme occupation and induction profiles are shown over time (**G**). Furthermore, simulated first dose (**E**) and steady-state (**F**) concentration profiles (broad pale bands) align with published profiles (narrow lines) for the dose-escalation of dabrafenib, which was administered in hypromellose (HPMC) capsules [7]. Ketoconazole dose-escalation profiles (**I**) align well [15]. After administrating 75 mg b.d dabrafenib for 22 days and concomitantly dosing ketoconazole (400 mg o.d.) for the last 4 days, the profiles at day 18 (**K**,**L**) and at day 22 (**J**–**L**) confirm a sufficient model fit [16]. Due to limited data availability from the original sources for the considered time courses of dabrafenib, we refrained from generating confidence intervals in all plots.

The final model (Appendix A) was able to reproduce the data [7,14,15,16] and simulation results are shown in Figure 3. The model was mostly quantitatively accurate, with rather slightly underestimated than overestimated drug concentrations. Figure 3 has only one entry that is not qualitatively correct, and that is the carbo-dabrafenib bar in Figure 3K. The carbo-dabrafenib concentration increases slightly instead to decline from day 18 to day 22, while the metabolites are generally underestimated in Figure 3K [16].

**Figure 4:**
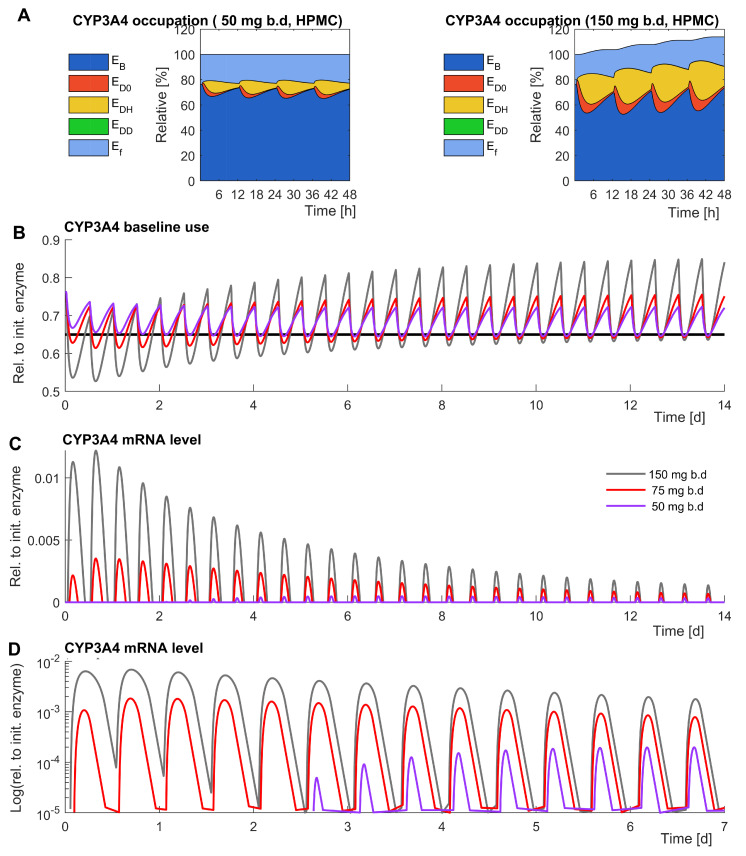
The final enzyme regulation motif is enzyme occupancy dependent. Without dabrafenib, the enzyme is either free $E_{f}$ or occupied with other baseline processes $E_{B}$ (**A**,**B**). After administrating 50 mg b.d dabrafenib (**A**), CYP3A4 is occupied mostly by hydroxy-dabrafenib $E_{DH}$, moderately by dabrafenib $E_{D0}$, and to a negligible extend by desmethyl-dabrafenib $E_{DD}$. With 150 mg b.d dabrafenib (**A**), the dabrafenib metabolites bind an increasing fraction of CYP3A4, and the total amount of enzymes increases, while 50 mg b.d. dabrafenib reduces the CYP3A4 availability for baseline processes to minimal requirements (black horizontal line), 150 mg b.d. dabrafenib leads to a clear deficit in baseline activities (**B**), which is highlighted by plotting the difference to the threshold (**C**) and log-transformed presentation of this difference (**D**). Gray: 150 mg b.d dabrafenib (HPMC). Red: 75 mg b.d dabrafenib (HPMC). Purple: 50 mg b.d dabrafenib (HPMC).

The model fit was only reached with the enzyme induction motif $E_{mRNA}$ and the associated parameters $k_{r},\;T_{E},\;k_{deg}$ (Figure 1). We assumed that the CYP3A4 enzyme level is dabrafenib dose-dependent (Figure 4A) and was controlled by the amount of substrate processed during baseline use. Only if the baseline use was insufficient and below the threshold (Figure 4B), CYP3A4 is released (Figure 4C). We depicted the enzyme induction process with 50mg, 75mg, and 150mg dabrafenib, respectively. The dose 50mg induced the enzyme slightly around day 3 (Figure 4D) without an overall effect on the total enzyme level (Figure 4A). The dose 150mg induced dabrafenib in total amount (Figure 4A) and triggered a sustained protein synthesis within the seven days simulated (Figure 4D).

#### 3.1.1. Carbo- and Desmethyl-Dabrafenib Accumulate According to the Model

**Figure 5:**
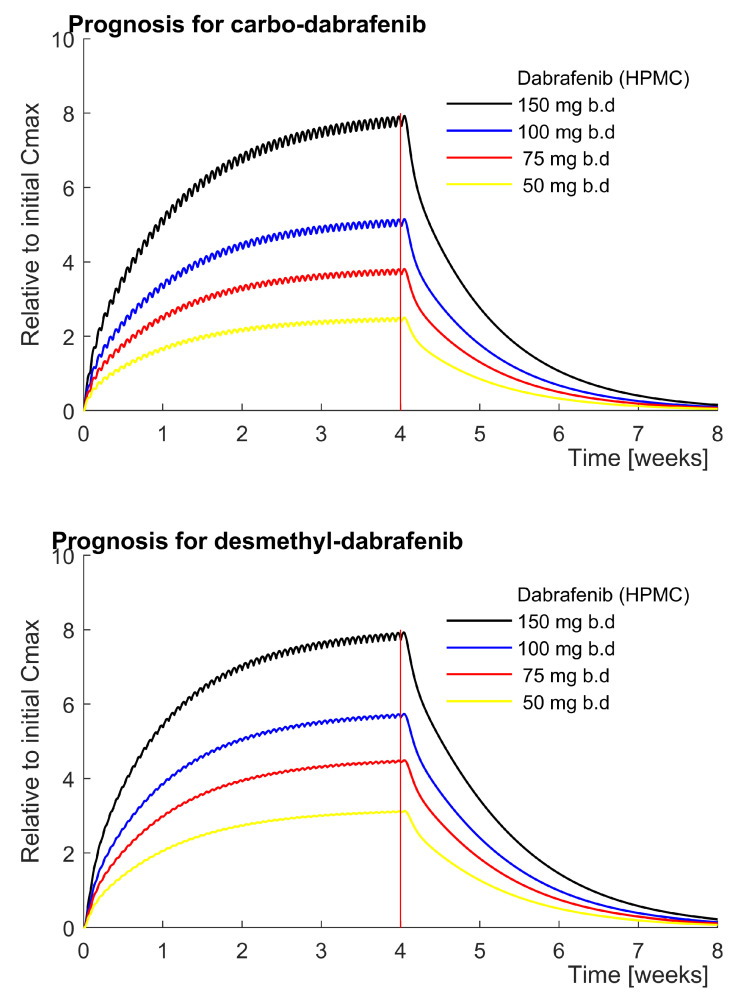
The predicted steady-state accumulation of carbo- and desmethyl-dabrafenib reaches the maximum after four weeks for clinically relevant doses of dabrafenib [6]. After 4 weeks, the maximal metabolite plasma concentration is approximately twice as high for 50 mg b.d dabrafenib compared to the first dose and eight-fold higher for elevated 150 mg b.d. dabrafenib. At all doses investigated, drug metabolites completely vanished four weeks after drug discontinuation.

After sufficient alignment with clinical data, the model can be carefully used to reconstruct the most likely PK-profiles of dabrafenib metabolites in relevant scenarios. Hereby, we obtained a saturation profile of both ineffectual carbo-dabrafenib and potent desmethyl-dabrafenib (Figure 5). A steady-state concentration was reached after four weeks, whereby the vast majority of accumulation occurred within the first two weeks. According to this prediction, four weeks are necessary for full blood plasma clearance after therapeutic dabrafenib discontinuation. At steady-state, we obtained the following peak concentration fold-changes. With a dose of 150 mg b.d dabrafenib, Cmax for both carbo-dabrafenib and desmethyl-dabrafenib increased 8-fold. With a dose of 100 mg b.d dabrafenib, Cmax increased 4–6 fold while Cmax increased 3–4 fold at a dose of 75 mg b.d.

#### 3.1.2. Higher Dabrafenib Levels Do Not Significantly Reduce Growth of 451LU Spheroids

To underline the relevance of elevated active desmethyl-dabrafenib levels on tumor outgrowth, we determined the impact of higher dabrafenib doses on 3D spheroids. 451LU melanoma spheroids were embedded into fibronectin supplemented hydrogels and exposed to 10 nM, 50 nM, and 100 nM dabrafenib, respectively.

**Figure 6:**
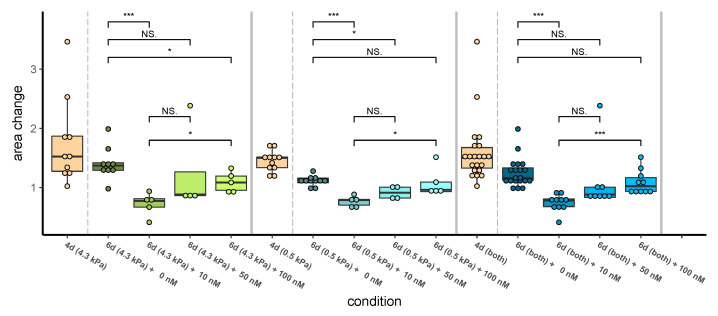
Higher doses of dabrafenib fail to reduce the growth of melanoma spheroids in fibronectin-coated dextran hydrogels with a mechanical shear modulus of 4.3 kPa (stiff, left part) and 0.5 kPa (soft, central part), respectively (Supplemental Information S2). Spheroids grew until day 4 with none and then for 48 h with 0, 10, 50, and 100 nM dabrafenib, respectively. Spheroid size/area at day 6 was normalized to spheroid area at day 4, which in turn was normalized to the spheroid area at day 0. The right panel combines data from soft and stiff hydrogels and shows that neither 50 nM nor 100 nM dabrafenib has a significant effect on spheroid growth. All three scenarios confirm a significant size reduction when 10 nM dabrafenib is administrating and a significant reduction of this effect if the dose is tenfold. ***: *p* < 0.001, **: *p* < 0.01, *: *p* < 0.05.

Confocal imaging confirms that 10 nM dabrafenib causes a significant reduction in the spheroid size, as expected (Figure 6). In contrast, higher doses with 50 nM and 100 nM dabrafenib did not significantly affect spheroid areas. Moreover, 100 nM dabrafenib was significantly less effective in reducing spheroid size, than treatment with 10 nM dabrafenib. Two spheroids, exposed to the highest dose of dabrafenib, even showed enhanced outgrowth compared to untreated spheroids, implying a tumor promotive function (Figure S9 in Supplemental Information S1), which could be the focus of future studies. Moreover, we observed enhanced growth of untreated 451LU melanoma spheroids in stiffer gels (Section S7 in Supplemental Information S1, raw data in Supplemental Information S3, video in Supplemental Information S6) and further complemented this observation with spheroids consisting of two additional BRAF-mutated A375 (Raw data in Supplemental Information S4) and SK-MEL-2 cell lines (Raw data in Supplemental Information S5).

## 4. Discussion

### 4.1. Model Extensions Are Well Supported by Data

In this work, the mechanistic model of dabrafenib metabolism of Ouellet et al. [7] has been complemented with dabrafenib metabolites, CYP3A4 induction, and ketoconazole DDI. The Ouellet model is not detailing dabrafenib metabolites and describes enzyme induction only as a phenomenological equation [7] applying a dose and time-dependent clearance term. Enzyme regulation was not further elaborated therein. The Ouellet model allowed to recapitulate four different experiments with two-week periodic administration of dabrafenib in four different doses and for the initial dynamic phases and the steady-state conditions [7]. The model presented in this work allows integrating 11 additional time-resolved data sets on the dabrafenib metabolism, ketoconazole, and the interaction of both (Figure 3) [7,14,15,16]. This mechanistically coupled PK model has 28 additional parameters, which is relatively few given the simultaneous fitting of eleven quite different time courses.

By not only considering the enzyme effect but also the presence of CYP3A4 in the free and bound form, the enzyme occupancy can be analyzed. Changes in occupancy of the enzyme directly impact CYPs enzyme capacity and explain DDI without requiring inhibitory enzyme kinetic terms. Consequently, the balance of already bound and still free CYP3A4 delivers an additional base for the mechanisms of enzyme induction. Indeed, the occupancy of enzymes is sufficient to describe the dabrafenib-ketoconazole DDI in our model. Relinquishing the simplifying assumptions for enzyme laws may be advised when enzyme occupancy is the expected core mechanism and simplifying assumptions cannot be maintained valid in subsequent model development. Eventually, the quantity and quality of data and underlying assumptions determine the degree to which mechanistic models based on reaction network theory may extrapolate, interpolate, and predict. This inductive research approach with mechanistic equation sets supports a more functional understanding than descriptive equations such as enzyme kinetic terms [19,20].

### 4.2. More Experimental Evidence Might Allow the Consideration of PXR

To model CYP3A4 induction, we assumed that CYP3A4 levels remain constant as long as dabrafenib leaves sufficient CYP3A4 capacity to process other substances. Any drug using CYP3A4 reduces the enzyme capacity and, if insufficient, induces CYP3A4. This mechanism is functionally equivalent to the previously assumed direct dabrafenib-dependent CYP3A4 mRNA level increase via the pregnane X receptor (PXR) [16]. However, assuming a PXR mediated mechanism necessitates an additional model extension, which requires further data on PXR-dabrafenib binding and activation. Even then, an experimentally proven PXR activation in vitro does not necessarily confirm functional changes in vivo due to often multiple interactions of xenobiotic drugs [21]. For example, ketoconazole inhibits CYP3A activity and moderately increases CYP3A mRNA expression [22]. A constant level of 6–25 μM ketoconazole blocks PXR- dependent CYP3A4 mRNA induction upregulation in vitro [23], however not in vivo [24]. This can be explained with the changing ketoconazole levels in blood plasma, where sufficient concentrations above 6 μM are only temporarily reached for a few hours (Cmax = 6000 ngmL=11.3μM) [15]. Furthermore, in vitro data may only validate a lower or upper limit because additional molecules might further reduce enzyme capacity in vivo. Consequently, it seems reasonable to keep model assumptions sparse, to restrict quantitative data integration to clinical sources, and to use in vitro data only for structural model properties. Only with sufficient data, a PXR-dependent enzyme regulation can be considered in future studies.

### 4.3. Accumulating Carbo-Dabrafenib and Desmethyl-Dabrafenib Concentrations Are Plausible

Based on the currently recommended drug dose of 150 mg b.d dabrafenib, our model predicted an 8-fold accumulation of carbo-dabrafenib and desmethyl-dabrafenib compared to the respective initial Cmax. This implies that these metabolites were still present to relevant levels when the next dose was administered, as it is also shown in Figure 3C,D. Several clinical studies supported our model predictions. 18 days after daily oral administration of 150 mg dabrafenib total exposure (AUC) in blood plasma were 13.5 times higher compared to dabrafenib and also 18.4–20.9 times higher compared to the other dabrafenib-related metabolites [16] (Supplemental Table S4 of Suttle et al.). Furthermore, in response to a single dose of 95 mg radiolabeled dabrafenib solution, the total exposure (AUC) of carbo-dabrafenib was 4.9 times increased compared to dabrafenib [14]. After 15 days of daily treatment with ≥70 mg b.d dabrafenib, exposure of carbo-dabrafenib in blood plasma was 2.78 to 8.77 times enriched compared to day one, and desmethyl-dabrafenib exposure was increased 12.6 to 35.0 fold compared to day one [6]. Consequently, the prediction is reasonable and aligns qualitatively with several clinical studies.

The clinical relevance of this accumulation can be interpreted if the declining drug potency of the dabrafenib metabolites is considered: dabrafenib > hydroxy-dabrafenib ≈ desmethyl-dabrafenib ≫ carboxy-dabrafenib [14]. While carboxy-dabrafenib is not expected to contribute to the clinical activity because of the low potency of 1/22 compared to the parental drug [6], its abundance and potential conversion into clinical active desmethyl-dabrafenib are of particular interest.

### 4.4. Acidity Might Shift the Local Balance towards Active Desmethyl-Dabrafenib

Concomitant administration of ketoconazole and dabrafenib diminishes the levels of carbo-dabrafenib, while the model suggests the opposite as shown in Figure 3K. However, the model considers only the general acidity-dependent turnover from inactive carboxy-dabrafenib into active desmethyl-dabrafenib [14], while in reality, the rate constant in the pH-dependent Michaelis–Menten term may be time-variant or location-dependent. Tumour tissues, as well as cancer cells in vitro, are known to harbor a low pH environment [25]. The extracellular acidification rate of the melanoma cell lines FM55-M2 and SK-MEL-28 was shown to be 15-fold increased compared to primary melanocytes [26]. Thus, acidity-dependent metabolite transformation may lead to unknown local consequences at the tumor side and may also explain why desmethyl-dabrafenib accumulates more (12.6–35 fold increase) than carbo-dabrafenib (2.8–8.8 fold increase) [6]. Location dependency of the individual drug accumulation could generally be investigated in tissue samples; to our knowledge, however, sufficient local drug level measurements do currently not exist for dabrafenib metabolites and are not recommended [27]. While the MCPK-based model is minimal, assumption-sparse, sufficiently resembles the clinical data, and help identify and interpret such potential knowledge gaps, PBPK-based models might be more suitable to model the effect of locally acidic spaces in further studies (Section S4 in Supplemental Information S1).

### 4.5. Dabrafenib Is Ineffective If Highly Dosed in a Fibronectin-Supplemented Environment

We demonstrated that low dabrafenib doses (10 nM) effectively reduce outgrowth of 451LU melanoma spheroids embedded into fibronectin-supplemented dextran hydrogels. In contrast, high dabrafenib concentrations (50 nM–100 nM) did not affect tumor outgrowth, while two melanoma spheroids even responded with accelerated outgrowth compared to untreated spheroids.

The dabrafenib concentrations applied to our in vivo mimicking 3D in vitro setting were within the physiological range, since dabrafenib temporarily reaches 3000 nM in blood plasma, according to the reproduced dabrafenib PK [7] (Figure S1 in Supplemental Information S1). However, concentration levels are likely lower in tissues and are usually applied at doses ranging from 3 to 100 nM in two-dimensional cell culture [28].

Local drug accumulations in patients are not dangerous per sé because of the low toxicity of targeted kinase inhibitors [6]; however, adverse effects and protective environments can emerge under these conditions [29]. BRAF inhibitors tend to trigger paradoxical hyper-activation of pERK and induction of neoplasia in the skin, such as cutaneous squamous cell carcinoma, whereby vemurafenib cause such effects more remarkably than dabrafenib or encorafenib [30]. Reactivation of pERK occured in melanoma spheroids embedded into stiff and fibronectin-supplemented hydrogels following treatment with vemurafenib [29]. Similar adverse effects of vemurafenib were confirmed in a variety of cell lines and tissue microarrays depending on fibronectin [31], as well as in fibronectin supplemented 3D collagen matrices [32]. In the present study we were able to show similar effects to be caused by the mutated BRAF-specific kinase inhibitor dabrafenib and it may have to be investigated whether accumulation of dabrafenib metabolites aggravates the risk profile of dabrafenib toward vemurafenib.

Recent studies in cancer research explained this effect to be dependent on the stiffness of the matrix and on the presence of fibronectin [29]. In this context, fibroblasts were shown to be able to switch the phenotype of melanoma cells to the mesenchymal state by shifting the signaling to the PI3K/mTOR pathway in a fibronectin-dependent manner [33]. The epithelial-mesenchymal transition as well as the metastatic potential were triggered by the mechanical characteristics of the microenvironment [34,35], causing a fibronectin-mediated transduction of the mechanical cues in the microenvironment into intracellular signals such as ERK, PI3K, ROCK-RHO through activation of the mechanosensor FAK. In parallel, mechanical cues also influenced Wnt and TGFB signaling thereby controlling YAP/TAZ mediated hippo signaling pathways [36,37]. It is increasingly accepted that tumor cells may develop non-cell autonomous resistance mechanisms [38], which not only requires targeting of the stroma [39] but also underlines the importance of three-dimensional cell culture-based screening systems for drug testing prior to their implementation into clinical trials [40].

## 5. Conclusions

The present study provides evidence that a dose of 150 mg b.d dabrafenib as currently administered to patients with BRAF-mutated malignant melanoma might be too high, and in the long run, may therefore lead to counter-intuitive ramifications. We demonstrate dabrafenib to lose its anti-tumor activity when applied at higher doses to the metastatic melanoma cell line 451LU, and accordingly, our study raises concerns about desmethyl-dabrafenib accumulation in vivo. It might be recommended to reduce the daily dose or to employ sequential therapy discontinuation to allow modulation of carbo-dabrafenib and desmethyl-dabrafenib levels in blood plasma. According to the drug selection study, 75 mg b.d turned out to be almost as beneficial as 150 mg b.d. dabrafenib in treating BRAF-mutated melanoma [6]. However, the low sample size, strong variability, and occasional dose escalation should be considered [6]. Hence, to further support our findings, experimental validation using additional BRAF-mutated melanoma cell lines or patient samples is recommended. Subsequently, a clinical study should be conducted to confirm safety and efficacy of a reduced dabrafenib dose, but also to provide further information on variability and population characteristics. While PBPK [41] and population models [7] may have more explanatory power to determine the individual dose decision, MCPK could support modeling of DDI and PK regarding drug metabolism with enzyme induction and hence provides a specialized niche in the comprehensive toolbox of contemporary pharmacokinetics.

## Data Availability

The authors declare that all codes and the data supporting the findings of this study are available within the publication and its Supplementary Information files. Raw image data require 20 GB storage space and are available on request. However, processed images with related area quantification are available in the Supplemental Information and are believed to sufficiently support the findings.

## Supplementary Materials

The following are available online at https://www.mdpi.com/article/10.3390/pharmaceutics14020310/s1, Supplemental Information S1: Model and experimental details (Additional references: [42,43,44,45,46]), Supplemental Information S2: Dabrafenib on 451LU Spheroids (Figure 6); Supplemental Information S3: 451LU spheroid growth images; Supplemental Information S4: A375 spheroid growth images; Supplemental Information S5: SK-Mel-2 spheroid growth images; Supplemental Information S6: Video of melanoma growth; Supplemental Information S7: Raw data of mechanical testing; Supplementary-Code-1.m: Reproduction of original model; Supplementary-Code-2.m: Main model; Supplementary-Code-3.m: Enzyme level determination; Supplementary-Code-4.m: Control loop; Supplementary-Code-5.m: Sensitivity analysis; Supplementary-Code-6.r: Plotting file for dabrafenib impact on spheroid.

## Appendix A. Model Equations and Parameters

The published model by Oeullet et al. [7] on dabrafenib PK is reproduced and discussed in Supplemental Information S1. The descriptive terms for compound clearance were substituted by the enzymatic dabrafenib clearance in the central compartment $CL_{met}\left(D_{0c},t\right)$:

$$\begin{array}{cc} \frac{dD_{0c}}{dt}=-\frac{\frac{Q}{F}}{\frac{V_{c}}{F}}\left(D_{0c}-D_{0p}\right) & +\frac{k_{D}\cdot D}{\frac{V_{c}}{F}}\cdot e^{-K_{D}\cdot \left(t-t_{lag}\right)}\\ & +CL_{met}\left(D_{0c},t\right)\\ \frac{dD_{0p}}{dt}=+\frac{\frac{Q}{F}}{\frac{V_{p}}{F}}\left(D_{0c}-D_{0p}\right) \end{array}$$

The parameters of the remaining original part are dabrafenib dose *D*, mass concentration of dabrafenib in central $D_{0c}$ and periperal $D_{0p}$ compartment, volume in central $V_{c}$ and peripheral compartment $V_{p}$, apparent inter-compartment clearance *Q*, formulation factor *F*, time lag $t_{lag}$, and absorption rate constant for dabrafenib $K_{D}$. As data on drug metabolism was obtained after administration of a dabrafenib suspension, we provide an alternative three-compartment model for a dabrafenib solution

$$\begin{array}{cc} \frac{dD_{0\mathrm{oral}}}{dt} & =-k_{D}\cdot D_{0\mathrm{oral}}\\ \frac{dD_{0c}}{dt} & =-\frac{Q_{Ds}}{V_{Dc}}\left(D_{0c}-D_{0p}\right)+\frac{k_{D}\cdot D_{0\mathrm{oral}}}{V_{Dc}}+CL_{met}\left(D_{0c},t\right)\\ \frac{dD_{0p}}{dt} & =+\frac{Q_{Ds}}{V_{Dc}}\left(D_{0c}-D_{0p}\right), \end{array}$$

which accounts for the mass concentrations in the oral $D_{0\mathrm{oral}}$, central $D_{0c}$, and peripheral $D_{0p}$ compartment.

We modeled the ketoconazole PK with a two-compartment model

$$\begin{array}{cc} \frac{dK_{oral}}{dt} & =-k_{K}\cdot K_{oral}\\ \frac{dK_{c}}{dt} & =\frac{k_{K}\cdot K_{oral}}{V_{Kc}}-k_{5b}\cdot E_{f}\cdot K_{c}+k_{5r}\cdot E_{K} \end{array}$$

with the ketoconazole level in the oral $K_{oral}$ and central $K_{c}$ compartment, absorption rate constant $k_{K}$, and central distribution volume $V_{Kc}$. The time derivative ddt on the left-hand side tracks molecule level changes as determined by the terms on the right-hand side. The second right-hand side term combines the ketoconazole levels in the central compartment $K_{c}$ with the free CYP3A4 enzyme levels $E_{f}$, whereby the molecule binding occurs with the binding affinity $k_{5b}$. All equations listed here must be seen as one coupled equation system, so that a negative term in one equation occurs as a positive term in another equation (transport) to ensure that no quantities disappear. For example, the positive term $k_{5r}E_{K}$ represents the disintegration of the ketoconazole-CYP3A4 complex $E_{K}$, which leads to more ketozonasole in the ketozonasole equation and more CYP3A4 enzyme in the free CYP3A4 equation. However the complex disintegration reduces the enzyme-ketoconazole complex levels and is thus considered as a negative term in the enzyme-ketoconazole-complex differential equation:

$$\begin{array}{cc} \frac{dE_{K}}{dt} & =k_{5b}\cdot E_{f}\cdot K_{c}-k_{5r}\cdot E_{K}-k_{5f}\cdot E_{K}, \end{array}$$

which is modelled similar to the enzyme-dabrafenib-complex

$$\begin{array}{cc} \frac{dE_{D0}}{dt} & =k_{1b}\cdot E_{f}\cdot D_{0c}-k_{1r}\cdot E_{D0}-k_{1f}\cdot E_{D0}, \end{array}$$

the enzyme-hydroxy-dabrafenib-complex

$$\begin{array}{cc} \frac{dE_{DH}}{dt} & =k_{2b}\cdot E_{f}\cdot D_{Hc}-k_{2r}\cdot E_{DH}-k_{2f}\cdot E_{DH}, \end{array}$$

or the enzyme-desmethyl-dabrafenib-complex

$$\begin{array}{cc} \frac{dE_{DD}}{dt} & =k_{4b}\cdot E_{f}\cdot D_{Dc}-k_{4r}\cdot E_{DD}-k_{4f}\cdot E_{DD}. \end{array}$$

The unspecific nature of CYP3A4 enzymes might lead to unfunctional competitive product binding [47] of carboxy-dabrafenib

$$\begin{array}{cc} \frac{dE_{DC}}{dt} & =k_{3b}\cdot E_{f}\cdot D_{Cc}-k_{3r}\cdot E_{DC}\;\left(\mathrm{neglected}\right) \end{array}$$

while other dabrafenib metabolites are converted and can consequently not be classified as product competition. Considering the subsequent and probably dominant acidity dependent transformation

$$\frac{dD_{Dc}}{dt}=\frac{k_{3v}D_{Cc}}{\left(k_{3km}+D_{Cc}\right)},$$

a product inhibition appeared mathematically redundant, which has been identified by sensitivity analysis. Consequently, the originally considered enzyme complex $E_{DC}$ has been removed and is not shown in Figure 1. The second element eliminated by sensitivity analysis is the CYP2C8+ pool:

$$\frac{k_{1v}\cdot D_{0c}}{\left(k_{1km}+D_{0c}\right)}:=0.$$

The enzymatic clearance of dabrafenib

$$CL_{met}\left(D_{0c},t\right)=\underset{\mathrm{CYP}3A4}{\underbrace{-k_{1b}\cdot E_{f}\cdot D_{0c}+k_{1r}\cdot E_{D0}}}$$

depends only on the enzyme CYP3A4 and releases hydroxy-dabrafenib. Hydroxy-dabrafenib depletion

$$\begin{array}{cc} \frac{dD_{Hc}}{dt}= & k_{1f}\cdot E_{D0}-k_{2b}\cdot E_{f}\cdot D_{Hc}+k_{2r}\cdot E_{DH}\\ & -\frac{Q}{V_{c}}\left(D_{Hc}-D_{Hp}\right)-k_{FU}\cdot D_{Hc} \end{array}$$

is CYP3A4 dependent only. The conversion from carboxy-dabrafenib to desmethyl-dabrafenib occures in acidic biological structures such as lysosomes [14]. The Michaelis-Menten kinetic is used for the pH-dependent transformation:

$$\begin{array}{cc} \frac{dD_{Cc}}{dt}= & -\underset{\mathrm{pH}}{\underbrace{\frac{k_{3v}\cdot D_{Cc}}{\left(k_{3km}+D_{Cc}\right)}}}+k_{2f}\cdot E_{DH}\\ & -k_{3b}\cdot E_{f}\cdot D_{Cc}+k_{3r}\cdot E_{DC}\\ & -\frac{Q}{V_{c}}\left(D_{Cc}-D_{Cp}\right)-k_{FU}\cdot D_{Cc}. \end{array}$$

The final depletion of desmethyl-dabrafenib

$$\begin{array}{cc} \frac{dD_{Dc}}{dt}= & \underset{\mathrm{pH}}{\underbrace{\frac{k_{3v}\cdot D_{Cc}}{\left(k_{3km}+D_{Cc}\right)}}}-\underset{\mathrm{CYP}2C19+}{\underbrace{\frac{k_{4v}\cdot D_{Dc}}{\left(k_{4km}+D_{Dc}\right)}}}\\ & -k_{4b}\cdot E_{f}\cdot D_{Dc}+k_{4r}\cdot E_{DD}\\ & -\frac{Q}{V_{c}}\left(D_{Cc}-D_{Cp}\right)-k_{FU}\cdot D_{Dc} \end{array}$$

and conversion to other metabolites (M)

$$\begin{array}{cc} \frac{dM}{dt}= & \underset{\mathrm{CYP}2C19+}{\underbrace{\frac{k_{4v}\cdot D_{Dc}}{\left(k_{4km}+D_{Dc}\right)}}}+k_{4f}\cdot E_{DD}-k_{FU}\cdot M \end{array}$$

is not only CYP3A4 mediated but also catalysed by the enzyme pool CYP2C19+ [13]. Each dabrafenib metabolite has a corresponding peripheral compartment

$$\begin{array}{cc} \frac{dD_{Hp}}{dt} & =\frac{Q}{V_{p}}\left(D_{Hc}-D_{Hp}\right)\\ \frac{dD_{Cp}}{dt} & =\frac{Q}{V_{p}}\left(D_{Cc}-D_{Cp}\right)\\ \frac{dD_{Dp}}{dt} & =\frac{Q}{V_{p}}\left(D_{Dc}-D_{Dp}\right) \end{array}$$

as in Ouellets dabrafenib model [7]. Additionally, parameters in Ouellets dabrafenib model are unaltered parameters of the extended model proposed. All metabolites eventually leave the system with the rate $k_{FU}$ and partly occur in the urine compartment (including faeces):

$$\frac{dU}{dt}=k_{FU}\cdot \left(D_{Hc}+D_{Cc}+D_{Dc}+M\right).$$

Dabrafenib and ketoconazole are assumed to be faster metabolised than excreted. The free and unbound CYP3A4 enzyme

$$\frac{dE_{f}}{dt}=-\frac{dE_{K}}{dt}-\frac{dE_{D0}}{dt}-\frac{dE_{DH}}{dt}-\frac{dE_{DC}}{dt}-\frac{dE_{DD}}{dt}-\frac{dE_{B}}{dt}+E_{reg}$$

is regulated $E_{reg}$ and does baseline work

$$\frac{dE_{B}}{dt}=k_{6b}\cdot E_{f}-k_{6r}\cdot E_{B},$$

which is generic and not known in detail as it is assumed that enzymes exists for a natural purpose independent of human created molecules. Accordingly, a steady state solution for free enzyme $E_{f0}$ and enzyme bound to baseline work $E_{B0}$ is calculated

$$E_{f0}=\frac{\frac{k_{6r}}{k_{6b}}E_{tot}}{\left(1+\frac{k_{6r}}{k_{6b}}\right)}\;;\;E_{B0}=E_{tot}-E_{f0}\;;\;E_{tot}=1$$

to provide molecular start values before dabrafenib is administered.

The enzyme level for the baseline use needs to be maintained above the threshold $T_{E}$. Otherwise, CYP3A4 mRNA is released

$$\frac{dE_{mRNA}}{dt}=k_{st}\max\left(\left(T_{E}-E_{B}\right),0\right)-k_{mRNAdeg}\cdot E_{mRNA},$$

and disappears with the mRNA degradation rate $k_{mRNAdeg}$. The first term contains the synthesis threshold $k_{st}=1\;$h${}^{-1}$. Additional CYP3A4 protein

$$E_{reg}=k_{t}\cdot E_{mRNA}$$

is released with translation rate $k_{t}$. CYP3A4 protein and mRNA levels are moderately correlated ($R^{2}=0.511$), whereby the correlation between CYP3A4 protein level and protein activity is better ($R^{2}=0.64$) [48]. Additional design options, such as considering CYP3A4 protein degradation, did not improve the model sufficiently enough to justify additional parameters. Table A1 lists the final parameters.

**Table A1:** Estimated parameter for dabrafenib metabolism.

Name	Value	Unit	Name	Value	Unit	Name	Value	Unit
$k_{D}$ (sol)	4.55	[1/h]	$k_{1b}$	8.63	$\left[\frac{L}{\mathrm{mg}\cdot h}\right]$	$k_{4f}$	2.26	[1/h]
VcFS	40.1	[L]	$k_{1r}$	13.5	[1/h]	$k_{4v}$	1.18	$\frac{\mathrm{mg}}{L\cdot h}$
VpFS	240.2	[L]	$k_{1f}$	2.72	[1/h]	$k_{4km}$	8.8	[mg/L]
QFS	0.98	[L/h]	$k_{2b}$	39.4	$\left[\frac{L}{\mathrm{mg}\cdot h}\right]$	$k_{5b}$	24.0	$\left[\frac{L}{\mathrm{mg}\cdot h}\right]$
$k_{K}$	1.83	[1/h]	$k_{2r}$	11.1	[1/h]	$k_{5r}$	5.05	[1/h]
VcK	51.05	[L]	$k_{2f}$	0.62	[1/h]	$k_{5f}$	1.03	[1/h]
FU	0.02	[−]	$k_{3v}$	0.14	$\left[\frac{\mathrm{mg}}{L\cdot h}\right]$	$k_{6b}$	5.76	[1/h]
$T_{E}$	0.65	[mg/L]	$k_{3km}$	17.28	[mg/L]	$k_{6r}$	1.79	[1/h]
$k_{t}$	0.61	[1/h]	$k_{4b}$	2.16	$\left[\frac{L}{\mathrm{mg}\cdot h}\right]$			
$k_{RNAdeg}$	10.107	[1/h]	$k_{4r}$	20.2	[1/h]			
