# Supplementary material for: Mechanistically Coupled PK (MCPK) Model to Describe Enzyme Induction and Occupancy Dependent DDI of Dabrafenib Metabolism
Source: Pharmaceutics. 2022 Jan 28;14(2):310. doi: 10.3390/pharmaceutics14020310 (PMC8875124; doi:10.3390/pharmaceutics14020310)
Supplement: Supplementary file 1 [file pharmaceutics-14-00310-s001.zip › pharmaceutics-1479084-supplementary/Supplementary_Information/Supplemental_Information_S1.pdf]

Supplemental Information S1: Mechanistically Coupled PK (MCPK) model to describe enzyme induction and occupancy dependent DDI of dabrafenib metabolism

Marco Albrecht[1,2], Yuri Kogan[3], Dagmar Kulms[4], and Thomas Sauter[5]

[1]*Université du Luxembourg; Systems Biology Group; 4367 Belvaux; Luxembourg; Email: marco.albrecht@posteo.eu*

[2]*esqLABS GmbH; 26683 Saterland; Germany; Email: marco.albrecht@esqlabs.com*

[3]*Institute for Medical Biomathematics (IMBM); 60991 Bnei Atarot; Israel; Email: yuri@imbm.com*

[4]*Technical University of Dresden; Experimental Dermatology; 01307 Dresden; Germany; Tel.: +49 (0)351 458-18973 ; Email: dagmar.kulms@uniklinikum-dresden.de*

[5]*Université du Luxembourg; Systems Biology Group; 4367 Belvaux; Luxembourg; Tel.: (+352) 46 66 44 6296; Email: thomas.sauter@uni.lu*

Contents

1 Reproduction of the Original Model 2

2 Chemical Reactions of Dabrafenib Metabolism 5

3 Model Development 5

4 MCPK Compared to Other Methods 13

5 Gel Characteristics 15

6 Image Quantification 16

7 451LU Spheroid Growth Behaviour 18

8 Sensitivity Analysis **20**
8.1 Impact on dabrafenib PK profile 20
8.2 Impact on Ketoconazole PK Profile 33
8.3 Impact on Ketoconazole Dabrafenib Interaction 46

9 References **53**

1 Reproduction of the Original Model

The current standard PK model [1]

$$\frac{dD_{0c}}{dt} = -\frac{\frac{Q}{F}}{\frac{V_c}{F}}(D_{0c} - D_{0p}) - \frac{\frac{CL}{F}}{\frac{V_c}{F}} \cdot D_{0c} + \frac{k_D \cdot D_{0c}}{\frac{V_c}{F}} \cdot e^{-k_D \cdot (t_{ld} - t_{\text{lag}})}$$
$$\frac{dD_{0p}}{dt} = +\frac{\frac{Q}{F}}{\frac{V_p}{F}}(D_{0c} - D_{0p}) - \frac{\frac{CL_{ind}}{F}}{\frac{V_p}{F}} \cdot D_{0p}$$

describes the central dabrafenib concentration D_{0c} in the central compartment volume V_c and the peripheral dabrafenib concentration D_{0p} in the peripheral compartment volume V_p [1]. The index 0 indicates unmodified dabrafenib in its original form. The equation of the oral compartment is analytically solved and appears as last first-order term in the central dabrafenib compartment. This term is influenced by the adsorption constant k_D and the time point of last administered dose t_{ld}. The time lag t_{lag} accounts for the delay in transition from the oral compartment. The transfer between central and peripheral compartment is mediated via the distribution clearance Q corrected by the bioavailability change from gelatin capsule to hypromellose (HPMC) capsule F and F_{Gel}. The oral clearance

$$\frac{CL}{F} = \frac{CL_0}{F} + \frac{CL_{ind}}{F}$$
$$\frac{CL_{ind}}{F} = CL_{ind,ss} \cdot \left(\frac{D \cdot F_{\text{Gel}}}{D_{Ref}}\right)^{\gamma} \left(1 - e^{\frac{-\log(2)t}{T_{50}}}\right).$$

sums up the initial oral clearance CL_0 and the inducible oral clearance CL_{ind}. The inducible clearance includes a time-dependent term with drug half-life T_{50} and the last administered drug dose D relative to the reference dose of 150 mg BID dabrafenib D_{ref}, adjusted via exponent γ. The additional inducible clearance for the peripheral compartment has been introduced to mimic both the steep decline in the trough concentration and the alignment of the curves in the steady state according to the original paper [1]. We reproduced the original work [1] shown in Supplemental Figure 1 with additional information, such as the concentration in the peripheral compartment. The code can be found in the supplemental files:

Supplemental Table 1: Parameter transferred from previous publication [1]

parameter	value	unit	Description
$tlag$	0.482	[h]	absorption lag-time Dabrafenib
$Fgel$	0.555	[-]	relative bioavailability of gelatin capsule to HPMC capsule
K_D	1.88	[L/h]	absorption rate
$CL0F$	17.0	[L/h]	apparent initial clearence
$CLindSS$	17.3	[L/h]	apparent inducible clearence at steady state
$Dref$	170.0	[mg]	reference dose
y	0.927	[-]	power of dependence of CLindSS on absorbed dose (LDOS X Fgel)
$T50$	67.3	[h]	half-life of clearance induction
$tlag$	0.482	[h]	absorption lag-time
VcF	70.3	[L]	apparent volume of central compartment
VpF	174.0	[L]	apparent volume of peripheral compartment
QF	3.3	[L/h]	apparent inter-compartmental

SupplementalCode1.m. Because the original model does not provide information about dabrafenib metabolites and because the clearance term is artificially time and dose dependent, we created a new model that explicitly addresses those issues. The proposed model uses the parameters of the original model listed in Table 1. The molecular weight of the dabrafenib molecule is 519.56 $^{g}/_{mol}$ [2]. The units from the the original work [1] in $^{ng}/_{mL}$ can be translated as follows

$$1\frac{\text{ng}}{\text{mL}} = \frac{10^{-9}\text{g} \cdot \text{mol}}{10^{-3}\text{L} \cdot 519.56\text{g}} = 1.925 \cdot 10^{-9}\frac{\text{mol}}{\text{L}} = 1.925\text{nM}$$

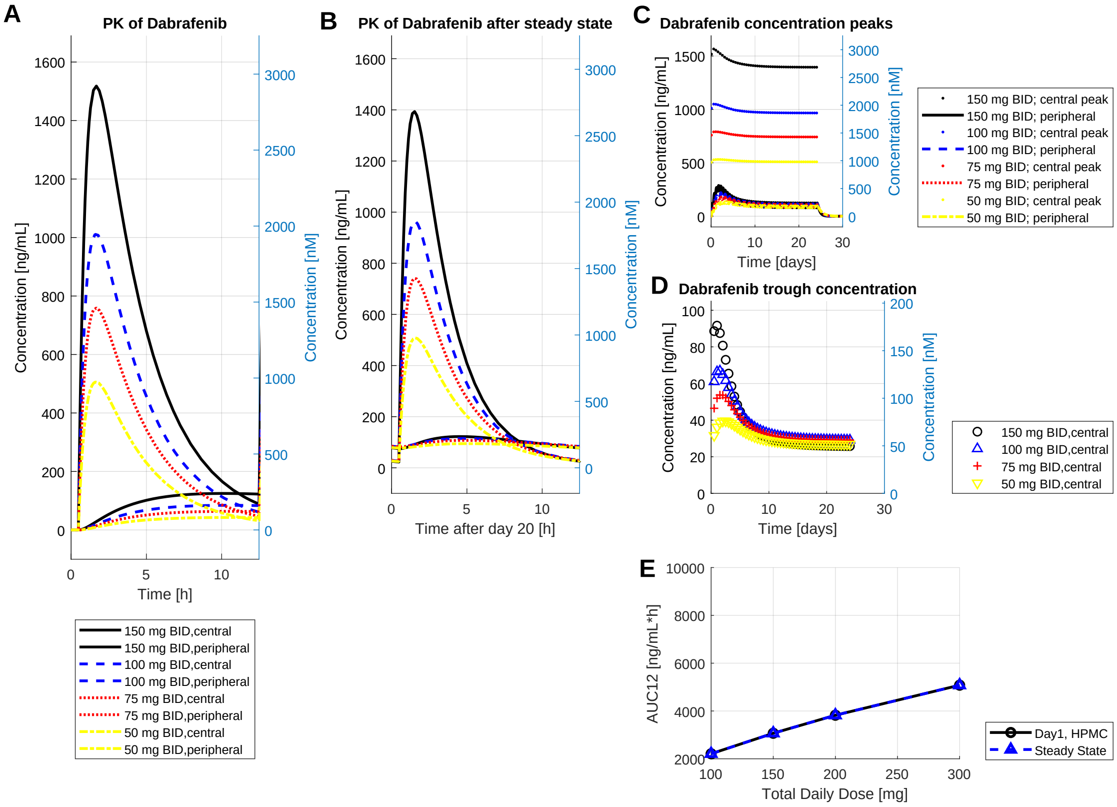

Supplemental Figure 1: **Reproduction of the original model [1].** A: Dabrafenib concentration pattern for a single dose. B: Dabrafenib concentration pattern after periodic drug administration. C: Peak values of dabrafenib PK after periodic dabrafenib administration. D: Trough concentration of dabrafenib PK after periodic dabrafenib administration. E: Drug exposure as area under the curve (AUC). All profiles are shown for 150 mg, 100 mg, 75 mg, and 50 mg dabrafenib. We show additionally the peripheral compartment and the related concentration in nanomolar nM.

2 Chemical Reactions of Dabrafenib Metabolism

The chemical modifications during dabrafenib metabolism are visualized in Supplemental Figure 2.

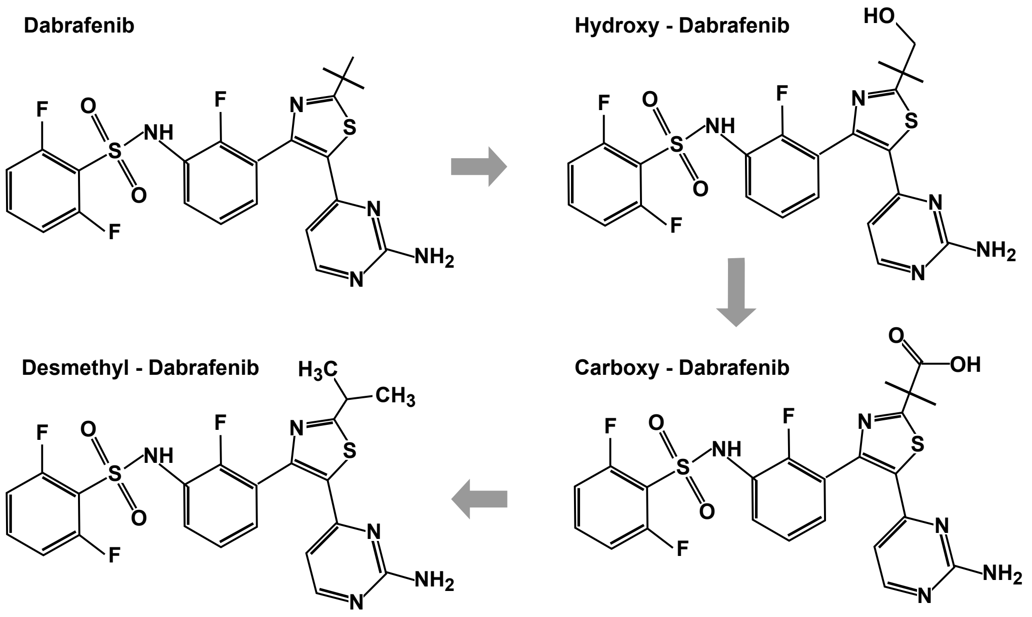

Supplemental Figure 2: Chemical reaction network of dabrafenib and most relevant metabolites based on Bershas *et. al.* [4].

3 Model Development

Model of Ouellet *et al.* Serves as a Template for Metabolite Dynamics

Ouellet *et al.* has described the reversible transport reaction of dabrafenib between the central D_{0c} and the peripheral compartment D_{0p} [1]. The upper right corner of Figure 1 represents the original model, whereby the index 0 indicates unmodified dabrafenib. This model incorporates an oral and an inducible clearance term, whereby the inducible clearance must appear in both the central and the peripheral compartment to reproduce the published model (coded in *Supplementary_Code_1.m* and visualized in the Supplementary Figure 1). We used the computational model of Ouellet *et al.* as a starting point of our model and replaced the descriptive clearance terms by mechanistic terms for enzyme-dependent clearance.

The bi-directional dabrafenib transport between the central and the peripheral

compartment was copied three times to represent dabrafenib metabolites. Hydroxy-dabrafenib D_{Hc}, carbo-dabrafenib D_{Cc}, and desmethyl-dabrafenib D_{Dc} are in balance with the allocated peripheral concentrations (D_{Hp}, D_{Cp}, D_{Dp}). The balance between dabrafenib metabolite in the central and the allocated peripheral compartment obey the model parameters of Ouellet *et al.* The definition of disjunct models of the dabrafenib metabolites lack interconnections, which were modeled on the base of enzyme tests as described in the following subsection.

In Vitro Experiments Guide the Model Structure on the Biotransformation from One Metabolite to Another

The metabolic flow from dabrafenib to the final metabolites required a study on recombinant CYP enzymes and human liver microsomes for the model structure. The study determined the relative contribution of each tested CYP to the metabolite depletion [3]. According to this study, CYP3A4 depleted 23% of dabrafenib. The enzymes CYP1A2 (1.9%), CYP2B6 (6.9%), CYP2C9 (10%), and CYP2C8 (56%) depleted 74,8 % of dabrafenib, and their activity was combined to the CYP2C8+ enzyme pool. In the next step, hydroxy-dabrafenib turns into carbo-dabrafenib by modification through CYP3A4 exclusively [3]. Carbo-dabrafenib converts to desmethyl-dabrafenib in an enzyme-independent but pH-dependent reaction [4, 3]. Subsequently, desmethyl-dabrafenib converts into several metabolites being united as element M. Desmethyl-dabrafenib depletion requires CYP3A4 (69%) as well as the enzymes CYP2C9 (9.6%) and CYP2C19 (22%) combined to the CYP2C19+ enzyme pool [3]. The dabrafenib metabolism is hereby fully described as shown in Figure 1 and supplemental Figure 2.
A few modeling assumptions were made to create a predictive and computational feasible model. First, we assumed that the pH-dependent turnover, as well as the enzyme pools CYP2C8+ and CYP2C19+, can be approximated by the Michaelis-Menten kinetics. Second, due to multi-functionality of the free enzyme CYP3A4, we assume that the reaction network model is the best representation of CYP3A4 function and experienced that simplification to a kinetic law neither lead to fewer parameters nor facilitates DDI and enzyme induction modeling. In this network model, the total CYP3A4 amount subdivides in the free enzyme form E_f without any bound substrate and the CYP3A4-metabolite complexes with bound dabrafenib E_{D0}, hydroxy-dabrafenib E_{DH}, or desmethyl-dabrafenib E_{DD} as shown in Figure 1. Third, the enzyme CYP2C9 appears twice but accounts only for a proportion of around 10%. It is, therefore, reasonable to integrate its contribution into the two enzyme pools CYP2C8+ and CYP2C19+ instead to model it as detailed as CYP3A4. Fourth, A sensitivity analysis revealed that our model fit rendered the CYP2C8+ pool redundant, wherefore we removed the equation for the CYP2C8+ pool. Fifth, the unspecific characteristic

of CYPs enables the reversible binding to all kind of substances, which can affect the time behavior [5]. Thus, the enzyme product carbo-dabrafenib might inhibit CYP3A4 without being converted. This product inhibition is not the case because the allocated parameters do not influence the simulated time profiles in the sensitivity analysis. We assumed instead that the pH-dependent change from carbo-dabrafenib to desmethyl-dabrafenib might be dominant over a potential product inhibition. The experimentally determined values *in vitro* were used to determine the model structure, but the model adjusted was based only on clinical *in vivo* data. Quantitative *in vitro* data is inappropriate for our kinetic parameters not only because systemic transport rates modify them but also for the following reasons:
First, the CYP enzyme capacity *in vitro* remains unclear about the enzyme capacity for the drug of interest if the enzymes are exposed to the various substrates *in vivo*. The liver detoxifies various metabolites, and each CYP type takes a different part of it with consequently less CYP capacity for the drug of interest than *in vitro*.
Second, the enzyme CYP3A4 is not only present in liver but also in the intestinal duodenum and jejunum processing, *e.g.*, almost half of a CYP3A4 substrate denoted midazolam [6]. Hence, liver microsome capacity is not representative of CYP composition and capacity of both liver and intestine.
Third, the fixation of the experimentally determined ratio of CYP3A4 (23%) to CYP2C8+ (75 %) turnover *in vitro* [3] resulted in the underestimation of the DDI effect in a preliminary simulation. The model shows only agreement with the data if CYP3A4 itself has more impact on the dabrafenib PK than the CYP2C8+ pool. Clinical data support our model outcome because the inhibition of CYP2C8 with gemfibrozil had less impact on dabrafenib PK than CYP3A4 inhibition with ketoconazole [7]. The sensitivity analysis revealed that the variation of the CYP2C8+ parameter was irrelevant for the simulation outcome.
In conclusion, *in vitro* data cannot be used to directly determine the kinetic model parameters of the proposed PK model, but are needed to determine the model structure. The model structure constrains the flexibility with which the model can approach experimental values. However, not all structure elements are needed to match the model with experimental observations. The CYP2C8+ pool allowed a CYP3A4 independent conversion from dabrafenib to hydroxy-dabrafenib *in vivo*, but the sensitivity analysis indicated a minor impact of this structure, which was consequentially removed.

Other Drugs, Such as Ketoconazole, Influence CYP3A4 Capacity and Thus Dabrafenib Metabolism

The previously described mechanistic ODE model on dabrafenib metabolism depends only on dabrafenib and might lack parameter identifiability because of the abundance of parameters. Perturbation of the CYP3A4 network module by an-

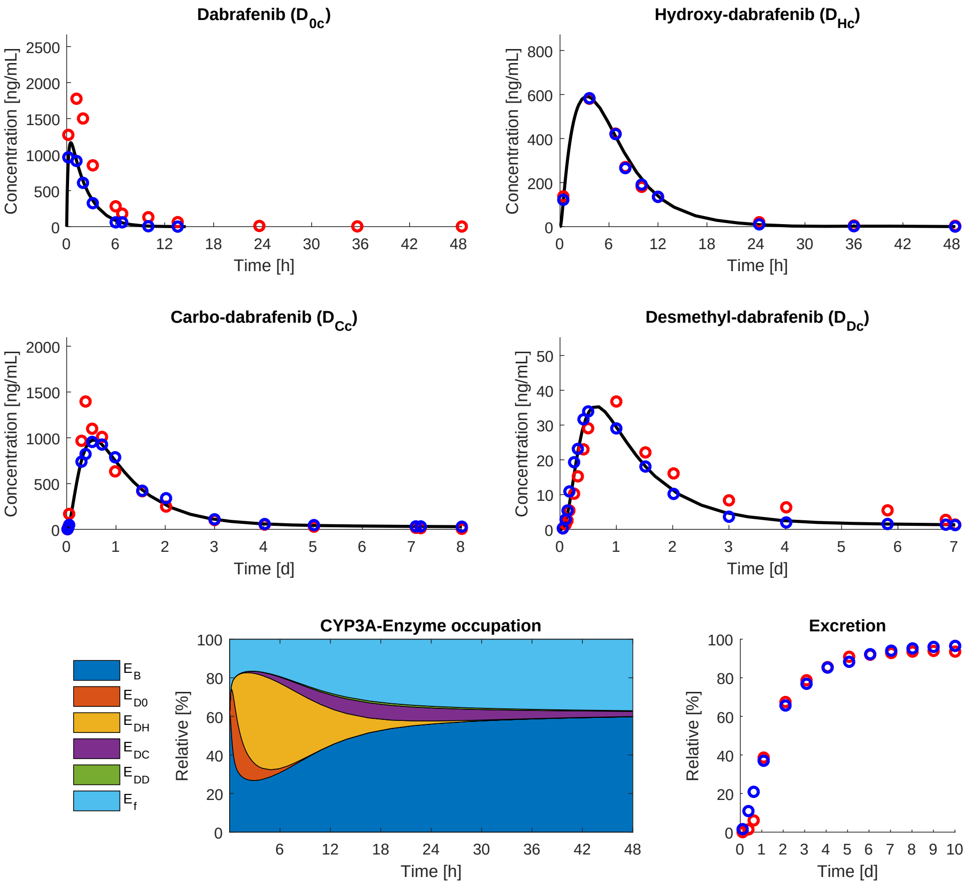

Supplemental Figure 3: Data fit without enzyme induction. This Figure might be compared with Figure 3 A-D,G and H. The PK profile for dabrafenib is underestimated probably because of the relative excess of CYP3A4 enzyme. Data source: [4].

other drug may help to constrain the parameter set further. Additionally, the modeling of DDI extends the model applicability for patients with multiple medications.

To improve both parameter robustness and the predictive power of the model, we used a PK model of ketoconazole, whereby the interaction between dabrafenib metabolism and the anti-fungal drug ketoconazole is mediated by CYP3A4, which modifies both dabrafenib and ketoconazole. Each drug occupying CYP3A4 for modification reduces the enzyme capacity of CYP3A4 and thus causes an inhibitory effect. CYP3A4 converts ketoconazole into the major metabolite N-deacetyl-ketoconazole, which is consecutively metabolized by flavin-containing monooxygenase (FMO) [8]. This CYP3A4 dependent route leads to 6 ketocona-

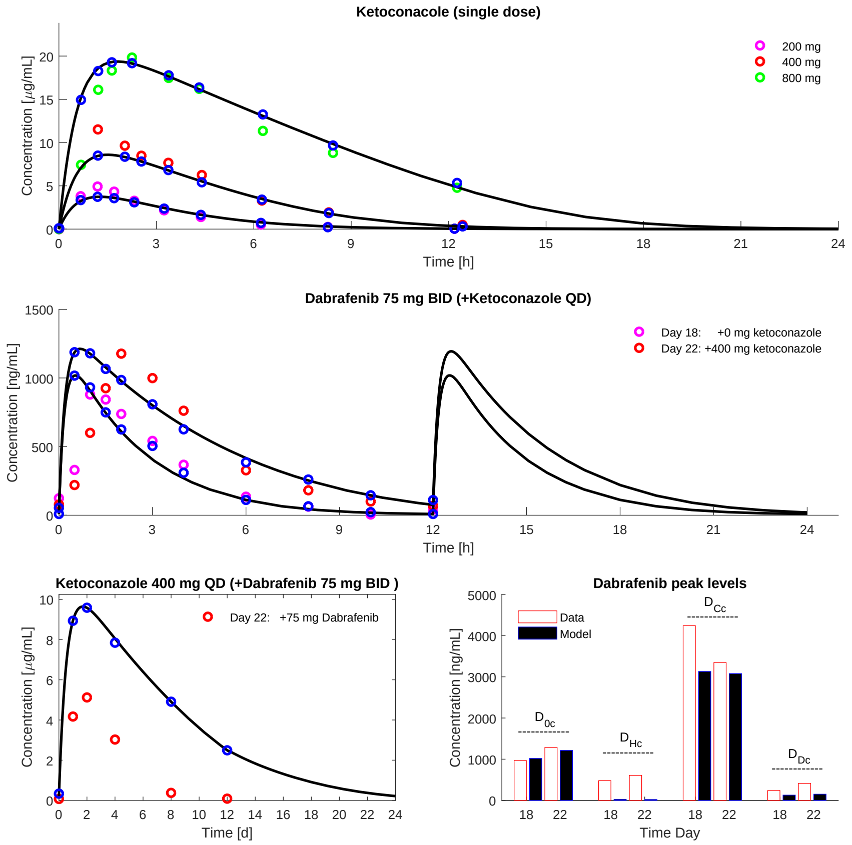

Supplemental Figure 4: Data fit without enzyme induction. This Figure might be compared with Figure 3 I-L. Overestimated abundance of Dabrafenib and ketoconazole at day 18 and day 22 probably because of relative lack of enzyme. Data source: [9, 7].

zole metabolites, and we assume that the remaining 22 ketoconazole metabolites do not affect our system [8].

In order to integrate ketoconazole data, we constructed a two-compartment PK model, which describes the transfer from the oral to the central compartment, and the first step of ketoconazole catalysis. The ketoconazole module is sketched in Figure 1 bottom left.

Model and Data Align Partially Without Enzyme Induction

The model presented so far was fitted to clinical data for dabrafenib, ketoconazole, and DDI between dabrafenib and ketoconazole according to the workflow scheme (Figure 2), but excluded CYP3A4 enzyme induction. The total CYP3A4 enzyme concentration was initially assumed to be constant and set to one. The performed variation of all other model parameters changed the concentration levels of model elements until a maximal agreement between data and model was archived. Because each dataset has been generated under different conditions, the parameter set has to be universally valid for all experimental conditions. Therefore, each experimental condition was matched by a comparable model configuration encompassing initial concentration levels, dosage forms, and time-points of drug administration. All context-specific models were then fitted simultaneously.

The following datasets have been selected and required minor model extensions for optimal data integration.

The first *in vivo* dataset showed how the blood plasma concentration of the dabrafenib metabolites changed over time [4]. In this clinical study, the dosage form was a drug solution with 95 mg radiolabeled dabrafenib. A drug solution is a dosage form that required an alternative three-compartment model, and therewith an oral compartment appeared on the top of Figure 1. The three-compartment model replaced the original model of Ouellet *et al.*, which is based on the pill dosage form [1]. Moreover, the dataset contained the drug fraction measured in urine and feces over time. The excreted end-products of dabrafenib metabolism appear as an element on the bottom right of Figure 1.

The second dataset about DDI between dabrafenib and ketoconazole shows time courses of dabrafenib metabolites at day 18 when administrated individually, and at day 22 if co-administrated with ketoconazole [7]. This dataset was essential to interconnect the dabrafenib metabolism module with the ketoconazole module.

The third integrated dataset was used to determine the parameters of the ketoconazole pharmacokinetic module [9]. Several ketoconazole datasets were assessable whereby datasets based on patients with food intake had delayed ketoconazole peak concentrations [10]. To select a dataset being appropriate in the modeling context, we took into account that dabrafenib is chemically unstable and should be ingested at least one hour before, or at least 2 hours after a meal according to EMA. Assuming that ketoconazole and dabrafenib are given together in the morning for the DDI study, the dataset for fasting patients has been taken as reference for the ketoconazole fit. In this study, 100 mg, 200 mg, or 400 mg ketoconazole in pill form were administrated as a single dose [9].

After simultaneously fitting all profiles to the selected datasets, we obtained the preliminary fit in Supplemental Figure 3 and 4. The model underestimated dabrafenib concentrations for the first dose PK and overestimated ketoconazole and dabrafenib

concentrations with periodic drug administration for two weeks. This misfit was expected under conditions omitting CYP3A4 induction. The CYP3A4 level was too high for the first-dose dabrafenib measurements and was too low for the drug levels after periodic drug administration for two weeks.

Identifying Optimal CYP3A4 Concentrations for Each Dose Period Reveals a Simple Enzyme Up-Regulation

In order to overcome the model inadequacy, the CYP3A4 level had to change over time, and an underlying mechanism for CYP3A4 enzyme induction had to be identified. Identifying the temporal change of CYP3A4 levels in workflow step two (Figure 2) was necessary to understand the underlying enzyme induction mechanism. Therefore, the parameter of the previously developed model got fixed, and the total CYP3A4 enzyme concentration was varied for each dose period. The resulting concentration pattern change of CYP3A4 and other model elements served as orientation for workflow step three.
The model was fitted to the trough concentration [1], which provided information about a potential drug accumulation or a change in the clearance rate over time. The trough concentration is the lowest drug level reached in each dose period before the next dose is administrated, and it declines with an increased enzyme concentration. The dosing period lasts 12 hours for dabrafenib. For 150 mg b.d dabrafenib, the trough concentration dropped immediately [1], indicating a fast induction of CYP3A4. Contrary, administration of 50 mg b.d dabrafenib caused an accumulation of the trough concentration for a couple of days before the trough concentration slowly declined [1], in this case suggesting a delayed and slow CYP3A4 induction.
In workflow step two, the total CYP3A4 concentration was adjusted for each drug period to reach the published trough concentration for 50 mg, 75 mg, 100 mg, and 150 mg b.d dabrafenib [1]. The determined enzyme levels revealed a simple saturation pattern with dose-dependent maximal levels of enzyme induction (Supplementary Figure 5). Because the CYP3A4 level pattern is less complex than the pattern of the trough concentration [1], the underlying regulatory mechanism must also be simpler than initially expected, which facilitated the control design in the next step.

A Three-Parameter Model of Enzyme Regulation is Sufficient to Bring the Whole PK Model in Agreement with Data

The identified saturation pattern of the enzyme increase might be best generated by a simple negative feedback loop with three parameters as shown in Figure 1 top left. Therefore, we assumed that a fraction of the total amount of CYP3A4

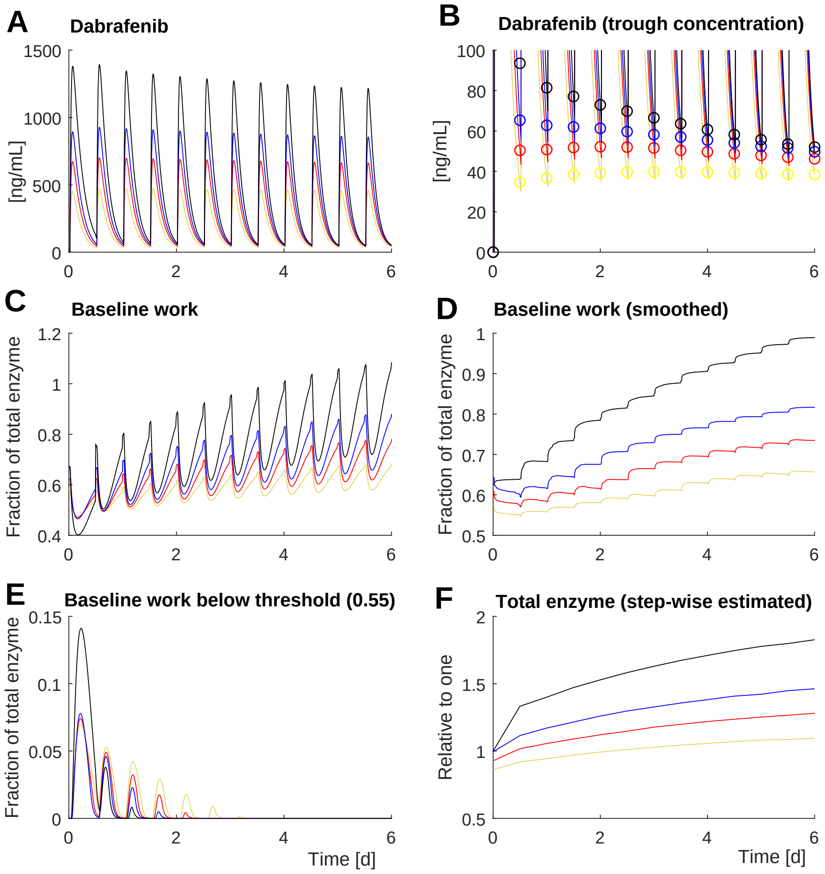

Supplemental Figure 5: Step-wise optimization of CYP3A4 level. A: the dabrafenib model without enzyme induction is used with fixed parameters. B: the algorithm optimizes the enzyme concentration for each dose period to reach the reported trough level. C: The enzyme-dependent baseline work is increasing with the increase of total enzyme (F). D: as C but with smoothed time course. E: the difference between CYP3A4 that performs baseline work and the minimal accepted concentration level of it. F: total enzyme calculated for each dose period.

catalyzes unknown metabolic processes *in vivo* E_B. The regulatory motif states that enough CYP3A4 must be dedicated to baseline processes to avoid the generation of additional CYP3A4. Accordingly, the difference between a threshold of minimal enzyme availability for baseline processes T_E and the actual level of enzyme performing baseline work correlated best with the change in total CYP3A4 enzyme level, depending on the mRNA translation rate k_t (Supplementary Figure 5 E and F). This mechanism ensures an optimal CYP3A4 level, which depends on the situational demand of CYP3A4.

After rationalizing the assumption of a negative feedback loop as a mechanism for enzyme induction, the regulatory motif was implemented in the main model, and several fitting circles refined the complete parameter set (Supplemental Table 1). The three-parameter-model for the regulation brought the model in agreement with clinical data sets (Figure 3). The final model delivered the expected CYP3A4 occupation for different drug doses and the time behavior of the CYP3A4 regulatory elements, as shown in Figure 4. We obtained instant and strong CYP3A4 production in response to 150 mg b.d dabrafenib and a delayed and slower CYP3A4 production for 50 mg b.d dabrafenib. Both are in agreement with the published trough concentration [1]. The simulation showed a moderate underestimation of long time metabolite concentrations in Figure 3 E and K, whereby the model remained qualitatively correct except for the carbo-dabrafenib bar in Figure 3 K [7].

4 MCPK Compared to Other Methods

The methodological place of MCPK models is illustrated in Supplemental Figure 6. MCPK models use *in vitro* data solely to inform structural relationships between different PK-profiles and is qualitatively only informed by clinical data. In contrast, PBPK models are equally informed by clinical data and *in vitro* data. Potential conflicts between PK profiles and *in vitro* data in PBPK models can be resolved by adjusting parameters, such as lipophilicity, solubility, and gastric

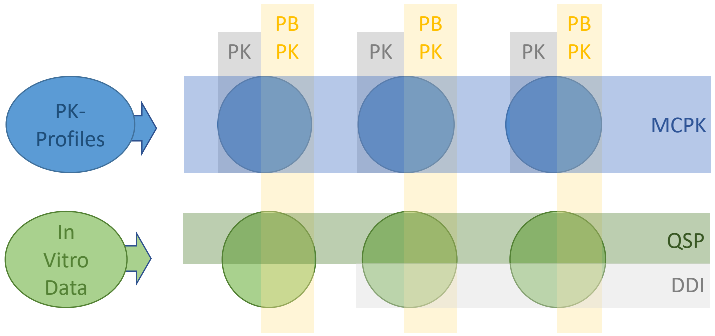

Supplemental Figure 6: In contrast to PK models, which fit PK profiles independent of each other, MCPK models mechanistically couples several related PK-profiles together on the high-level and fits them simultaneously. PBPK models fit PK profiles together with related *in vitro* data, while QSP models often interlink several PBPK models at the cellular level. Typical DDI models often interlink only models of two compounds.

emptying rate. As MCPK models strictly focuses on relationships between PK profiles, it has neither the ability to integrate quantitative *in vitro* data nor, has the flexibility within the causal chain between molecular and clinical data to achieve consistency. In the end, MCPK and PBPK models complement rather than exclude each other, and MCPK can be most likely be used as a bridge between PK and PBPK models.

The aspect of uncertainty and population characteristics in MCPK models is open. MCPK models have neither as few phenomenological parameters as PK models, nor precise and experimentally definable parameters as in PBPK models. When fitting an MCPK model to population data, the assumed coupling mechanism between PK profiles restricts fitting flexibility, while offering flexibility to the phenomenological and high-level causative effect of enzymatic kinetic activity with locally unknown parameter values. This means that fitting MCPK models to population data might force uncertainty and diversity to allocate more to biologically more meaningful parameters instead of being absorbed in inter-PK-profile inconsistencies. If considering population parameters such as weight, age, and height, PBPK remains the best choice, whereby our network can also be integrated as quantitative systems pharmacologic (QSP) model within the PBPK framework.

5 Gel Characteristics

The hydrogel polymerizes after pipetting the ingredients together as shown in Supplemental Figure 7. The gel is a cell degradable gel, when CD-linker is added and a non-degradable gel, when PEG-linker is added. Depending on the volume-fraction of thiol-reactive polymer, the CD or PEG gel is classified as as a 2, 4, 6, or 8 gel. Consequently, we work with eight gels: CD2, CD4, CD6, CD8, PEG2, PEG4, PEG6, and PEG8.

Volume [µl]				Ingredients
**2**	**4**	**6**	**8**	
14.8	10.7	5.7	0.7	Water
2.4	2.5	2.5	2.5	10xCB PH 7.2
**2**	**4**	**6**	**8**	**Thiol-Reactive Polymer**
1.8	1.8	1.8	1.8	Fibronectin
6	5	5	5	Cell Suspension
**3**	**6**	**9**	**12**	**CD (cell degradable) or PEG Link**
30	30	30	30	

Supplemental Figure 7: Pipetting scheme to produce hydrogel.

However, linearly increasing the volume fraction of polymer does not automatically lead to a similar linear increase in mechanical strength. Thus, we performed mechanical testing with the result summarized in Supplemental Figure 8.

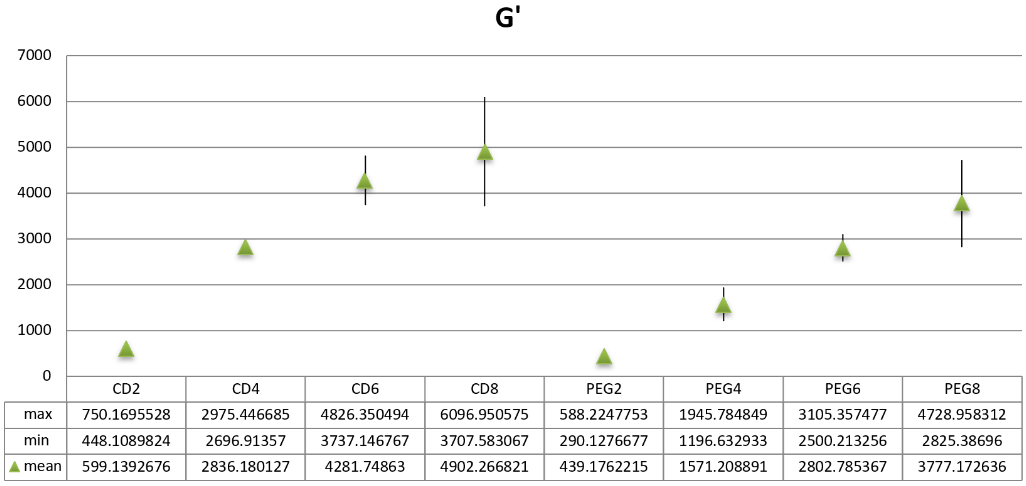

	CD2	CD4	CD6	CD8	PEG2	PEG4	PEG6	PEG8
max	750.1695528	2975.446685	4826.350494	6096.950575	588.2247753	1945.784849	3105.357477	4728.958312
min	448.1089824	2696.91357	3737.146767	3707.583067	290.1276677	1196.632933	2500.213256	2825.38696
▲ mean	599.1392676	2836.180127	4281.74863	4902.266821	439.1762215	1571.208891	2802.785367	3777.172636

Supplemental Figure 8: Shear modulus of all eight hydrogels.

6 Image Quantification

The spheroid areas where recorded as shown in Supplementary Figure 9. Image quantification was performed with Fiji in batch process using the following code:

```
t=getTitle();
Stack.setChannel(2)
run("Delete Slice", "delete=channel");
run("Z Project...", "projection=[Max Intensity]");
run("16-bit");
run("Green");
run("Gamma...", "value=0.50");
close("\\Others")
run("Enhance Contrast", "saturated=0.35");
run("Apply LUT", "slice");
run("8-bit");
run("Duplicate...", " ");
run("Smooth", "slice");
run("Smooth", "slice");
run("Smooth", "slice");
run("Smooth", "slice");
setAutoThreshold("Intermodes dark");
run("Convert to Mask");
run("Analyze Particles...", "size=2-Infinity show=Outlines clear include sum-
marize");
run("Images to Stack", "name=Stack title")
run("Next Slice [>]");
run("Delete Slice");
run("Z Project...", "projection=[Sum Slices]");
rename(t);
saveAs("Jpeg", "E:\\ResultImageQuant\\");
```

The performance of the code can be seen in Supplemental Figure 10 by comparing the original image with the detected boundary.

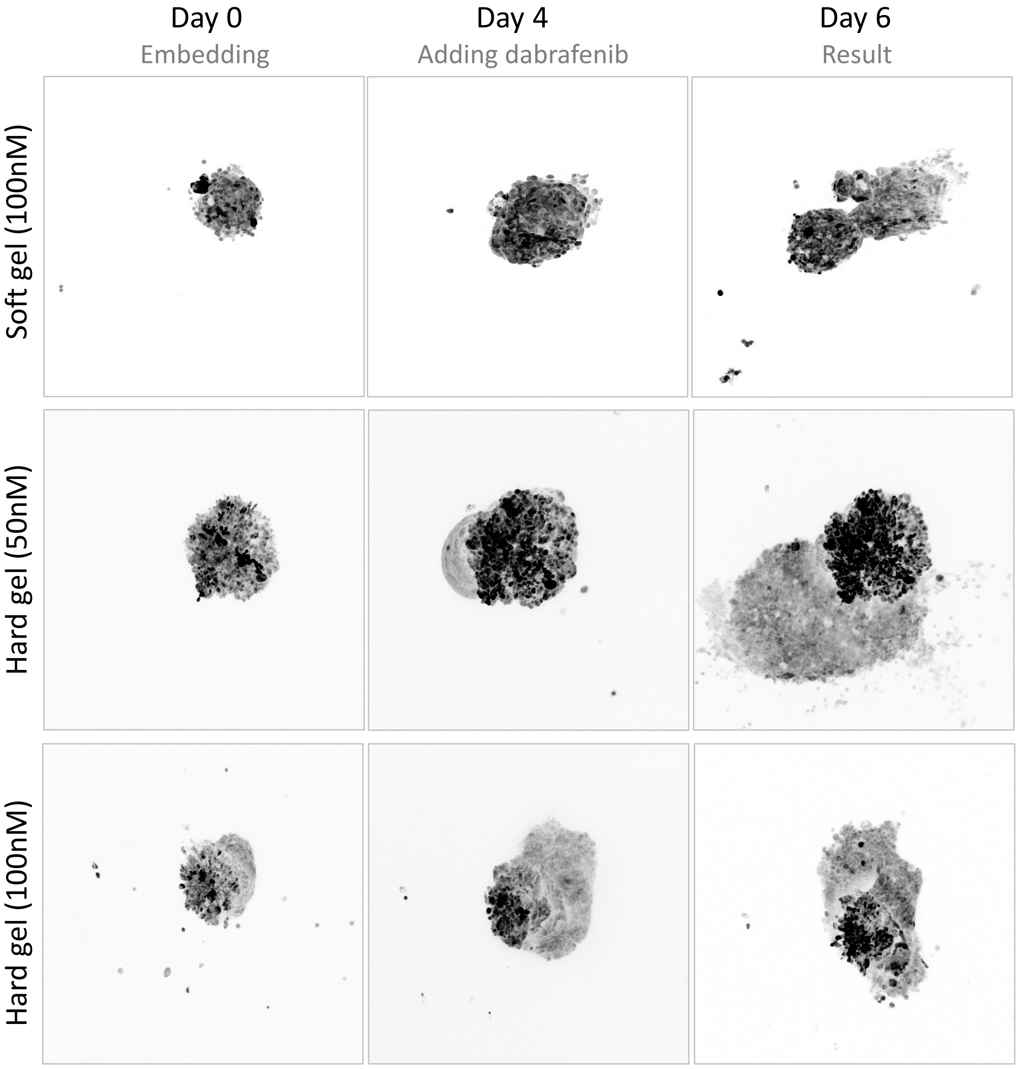

Supplemental Figure 9: Three spheroids with unexpectedly high growth rate, despite or because of the high dabrafenib concentration. Spheroids were treated at day 4 and evaluated at day 6. The occasional outburst of growth above the related control values was observed for two samples exposed to high drug concentrations. The spheroid at the bottom was not striking in area growth but seemed to grow toward the background.

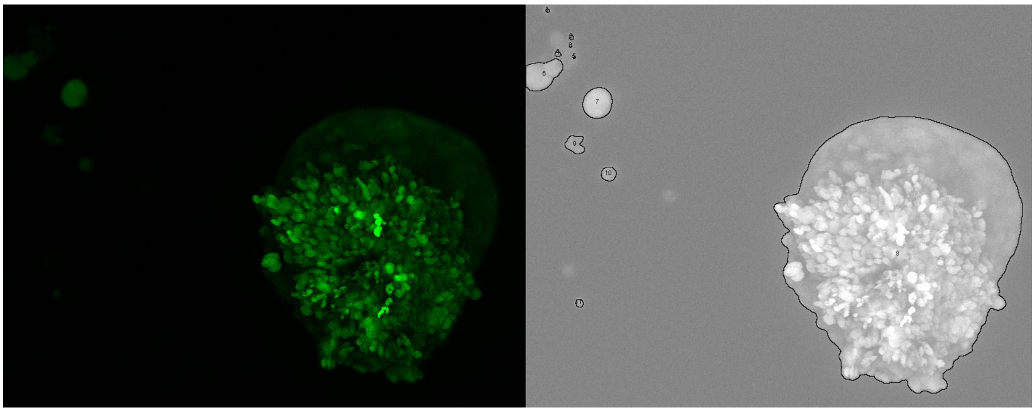

Supplemental Figure 10: Image quantification analyses the original image (left) and generates the control figure (right). The control figure contains the light gray version of the original image and the dark gray boundary, which enfolds the area to be quantified.

7 451LU Spheroid Growth Behaviour

We observed spheroid growth over 9 days in different stiff gels. For the 451LU cell line, we provide the analysis result in Supplemental Figure 11 and some exemplary spheroids in Supplemental Figure 12. Raw data for 451LU is available in Supplemental Information 3 and for A375 and SK-Mel-2 in Supplemental Information 4 and 5.

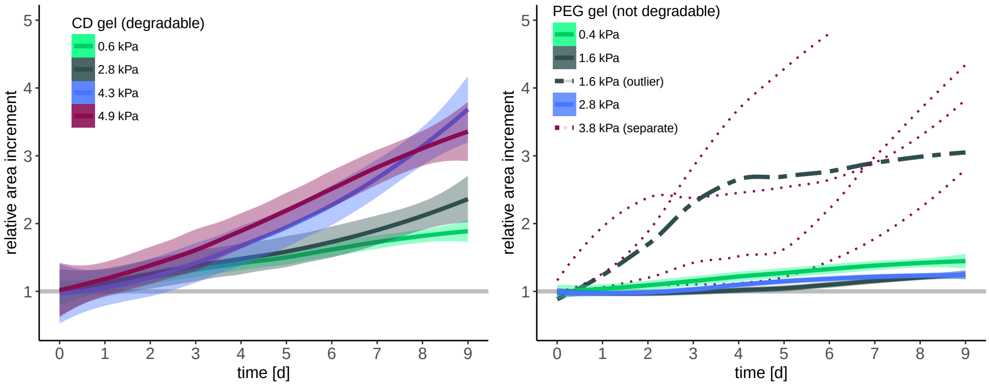

Supplemental Figure 11: 451LU spheroid growth in four CD and four PEG gels. The not degradable gel is inhibiting the growth, except in the stiffest gel, where results of the individual spheroids are depicted.

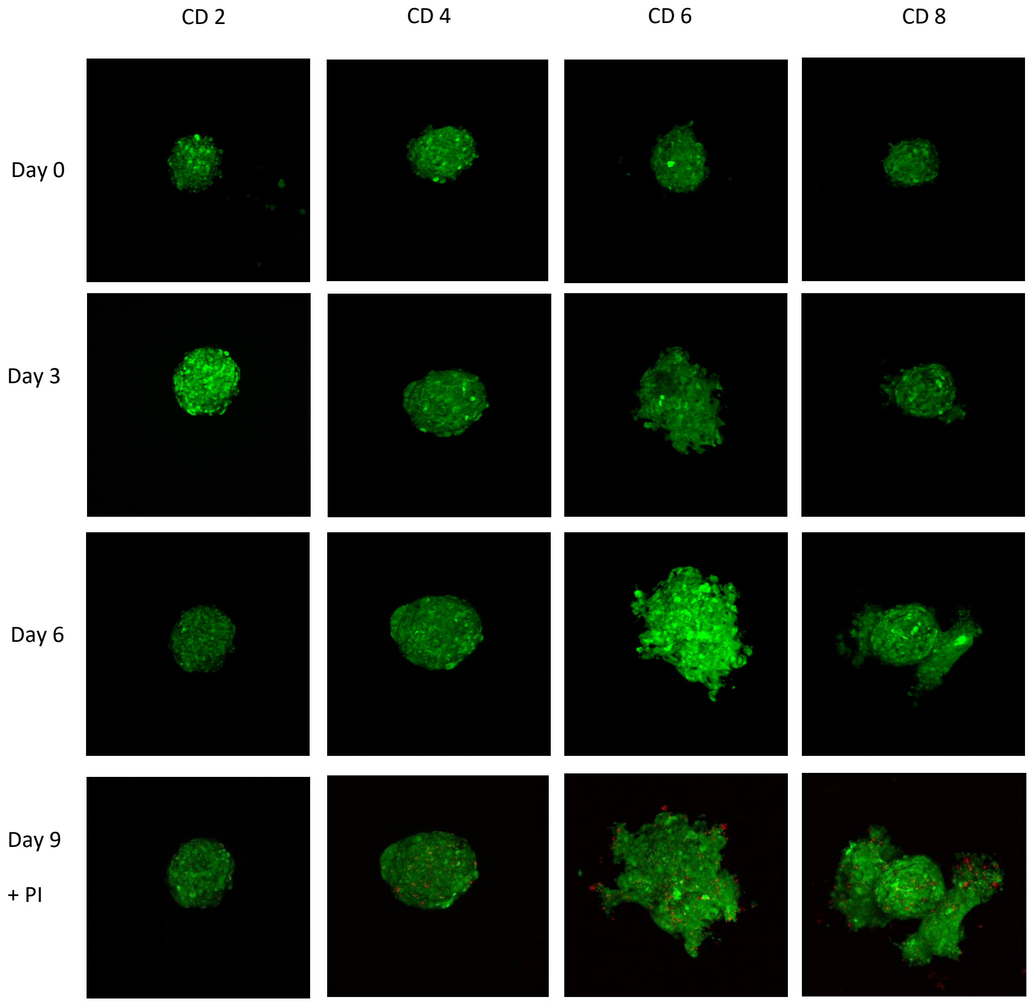

Supplemental Figure 12: 451LU spheroid growth in four CD gels with different stiffness. Exemplary spheroids (GFP positive to appear green) from the first biological replicate. PI is the red cell-death indicator propidium iodide.

8 Sensitivity Analysis

Each parameter is incrementally decreased to 10% or increased to 1000% of the original parameter value. Consequentially, the time course profile and the fitting quality changes for sensitive parameters and does not change for not sensitive parameters. Two versions of sensitivity analysis have been generated. The first version shows how the residual sum of squares (RSS) changes with the change of parameter values in respect to the measurement values. The displayed residuals are normalized by the residuals, which come from the fit with the optimal parameter set. The second version does not pay attention to the correct measurement values. It shows how the time profile pattern changes with the parameter change in comparison to the time profile based on optimal parameters. Well-fitted profiles tend to have v-shaped RSS profiles. Insensitive parameters have a perfectly flat RSS profile and an empty time course profile.

8.1 Impact on dabrafenib PK profile

The following sensitivity plots were computed with the Supplementary Code 5 (Supplementary-Code-5.m). Not all parameter influence all time profiles with the consequence that some plots are flat and the heat-maps are empty. A parameter is considered valuable if it influences at least one of the tested profiles.

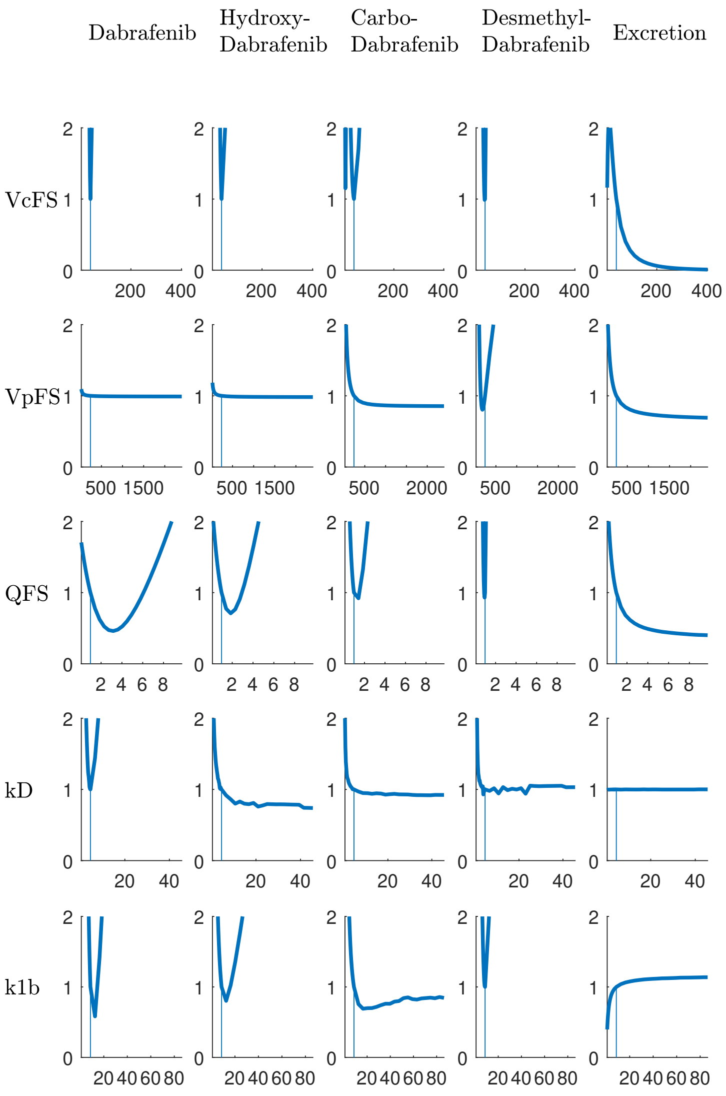

Supplemental Figure 13: Sensitivity Analysis: relative change of fitting quality (RSS) if parameter value is changed.

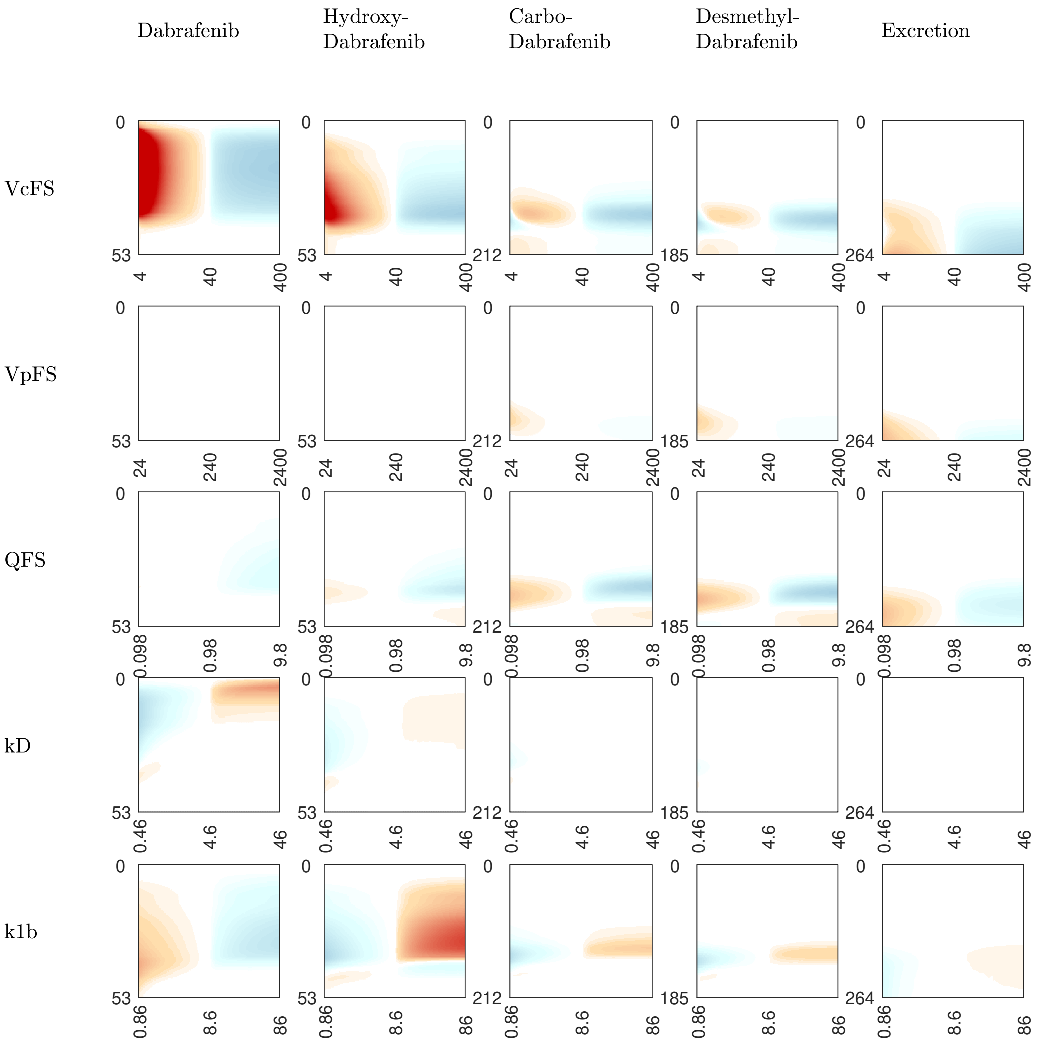

Supplemental Figure 14: Sensitivity Analysis: time course change over different parameter values. Red areas indicate that these periods the time-course is above the original dynamic. Cyan areas indicate that the dynamic descend below the original dynamic.

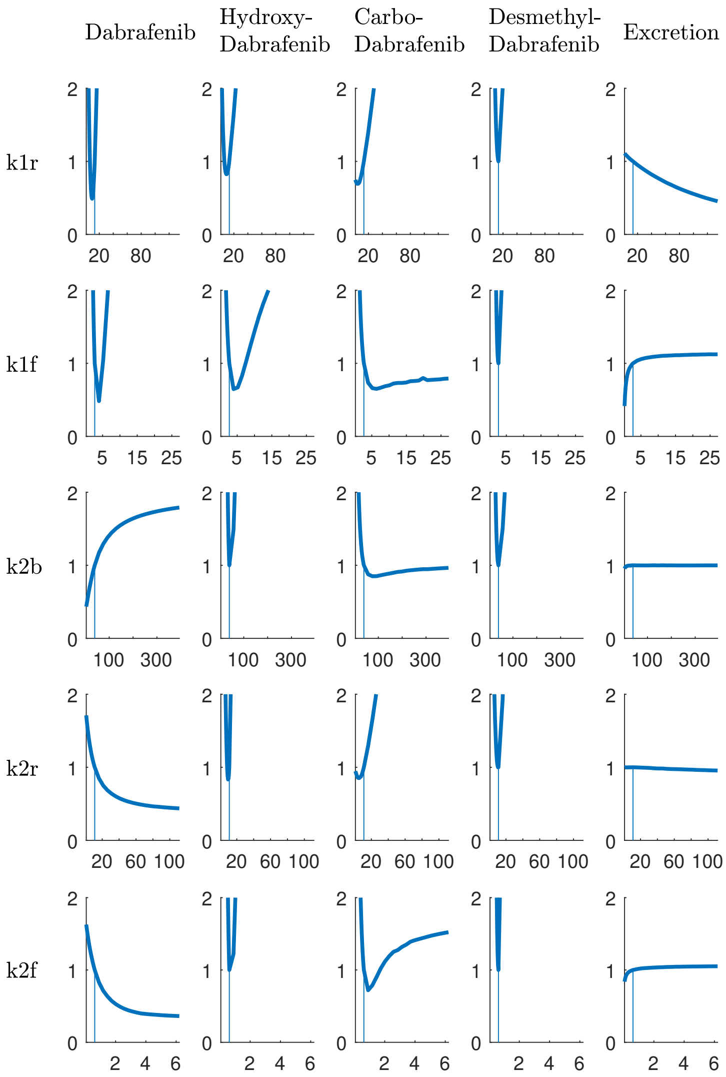

Supplemental Figure 15: Sensitivity Analysis: relative change of fitting quality (RSS) if parameter value is changed.

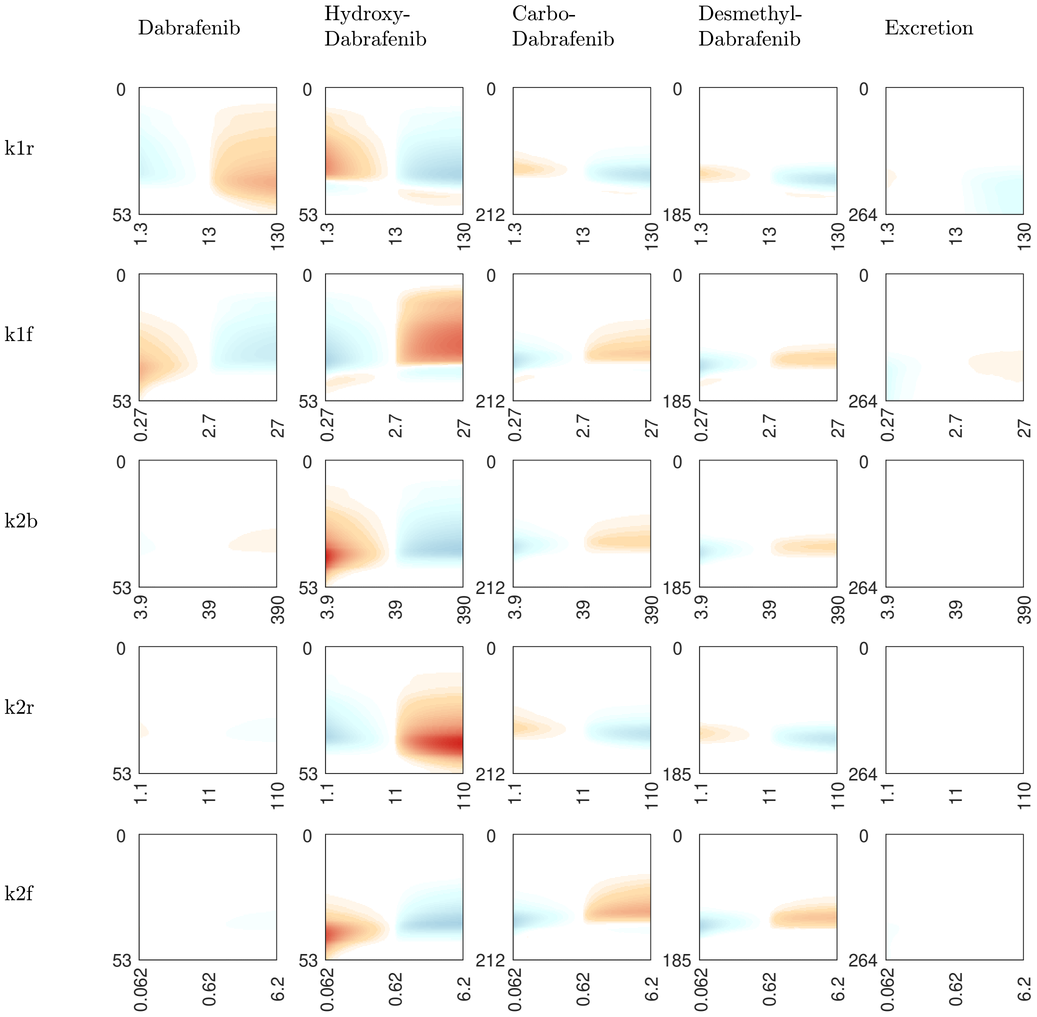

Supplemental Figure 16: Sensitivity Analysis: time course change over different parameter values. Red areas indicate that these periods the time-course is above the original dynamic. Cyan areas indicate that the dynamic descend below the original dynamic.

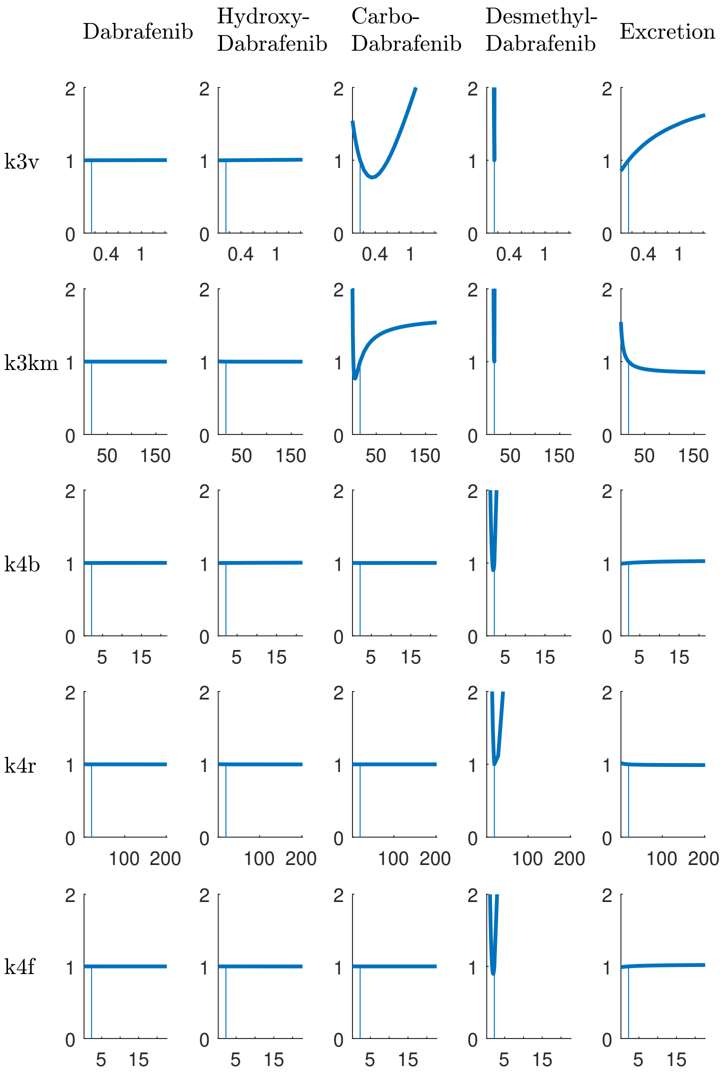

Supplemental Figure 17: Sensitivity Analysis: relative change of fitting quality (RSS) if parameter value is changed.

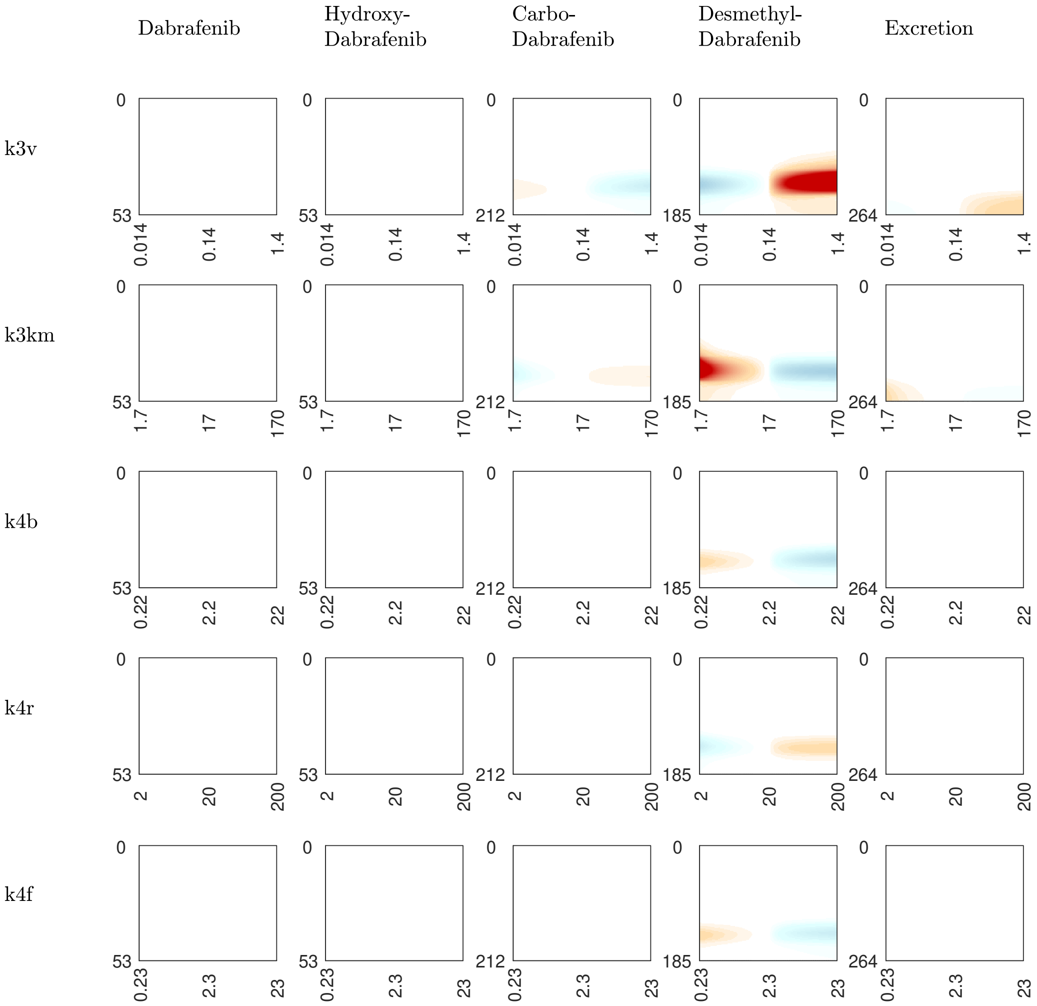

Supplemental Figure 18: Sensitivity Analysis: time course change over different parameter values. Red areas indicate that these periods the time-course is above the original dynamic. Cyan areas indicate that the dynamic descend below the original dynamic.

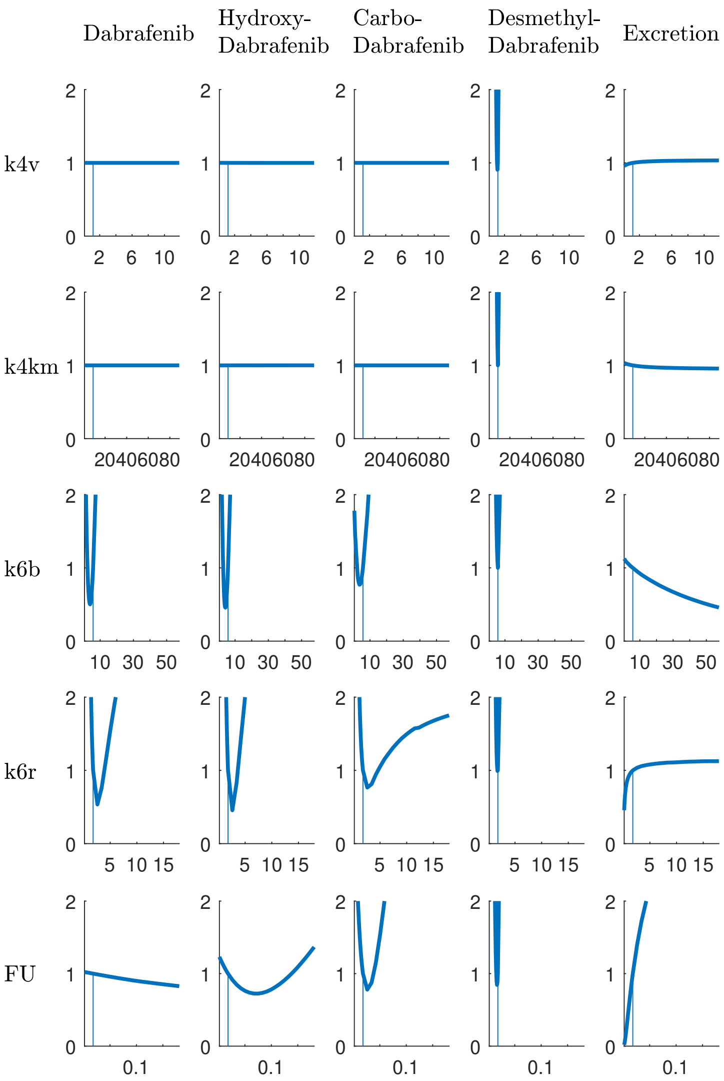

Supplemental Figure 19: Sensitivity Analysis: relative change of fitting quality (RSS) if parameter value is changed.

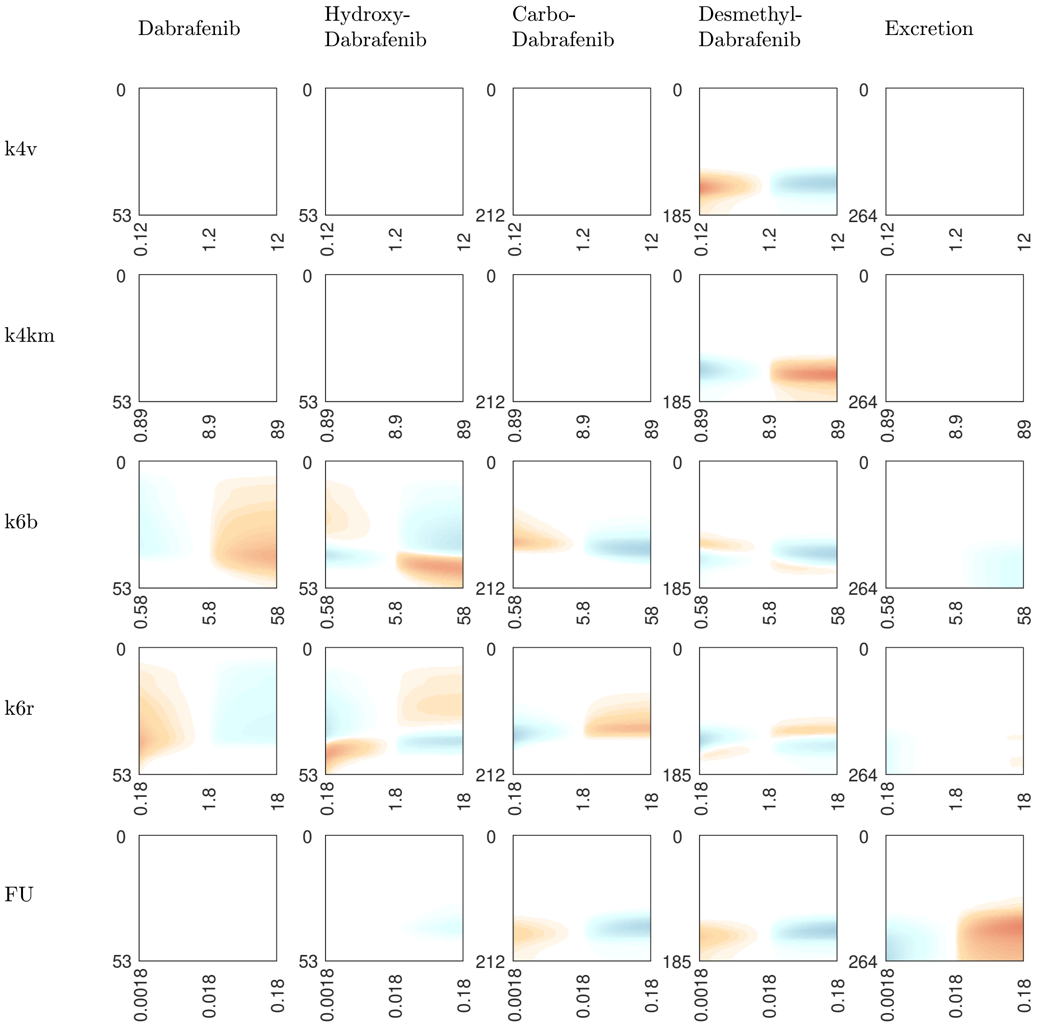

Supplemental Figure 20: Sensitivity Analysis: time course change over different parameter values. Red areas indicate that these periods the time-course is above the original dynamic. Cyan areas indicate that the dynamic descend below the original dynamic.

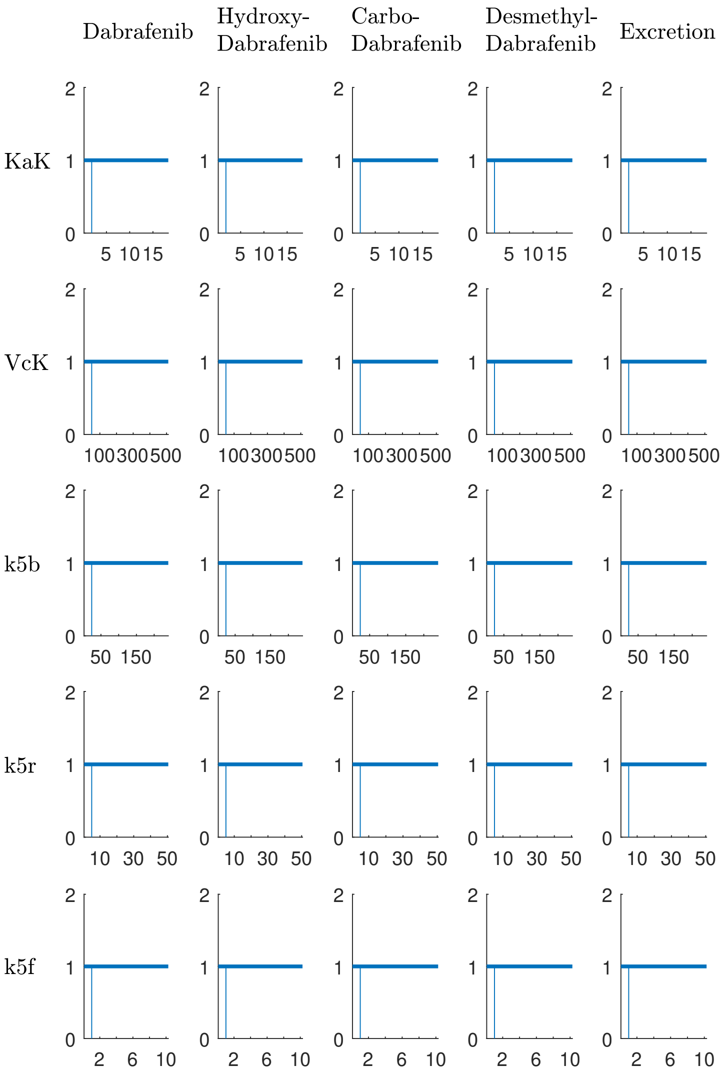

Supplemental Figure 21: Sensitivity Analysis: relative change of fitting quality (RSS) if parameter value is changed.

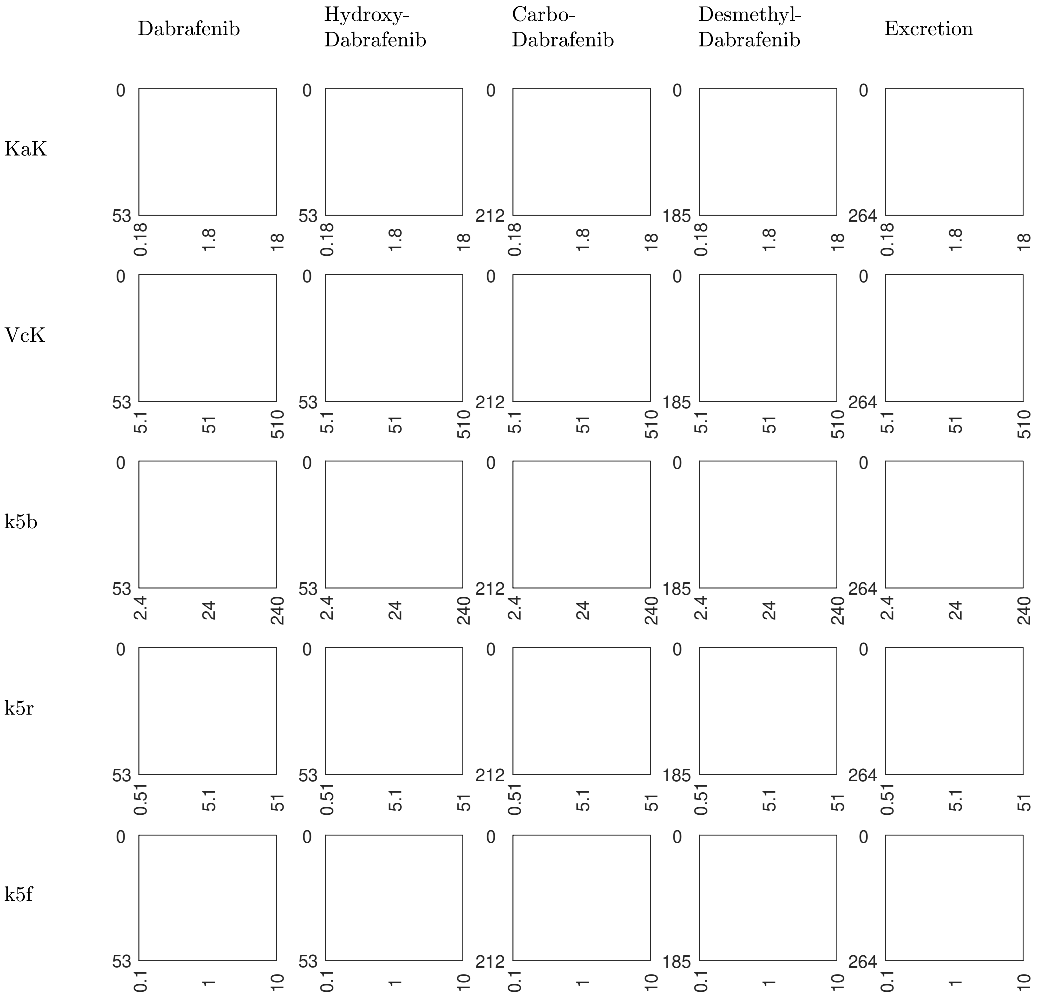

Supplemental Figure 22: Sensitivity Analysis: time course change over different parameter values. Red areas indicate that these periods the time-course is above the original dynamic. Cyan areas indicate that the dynamic descend below the original dynamic.

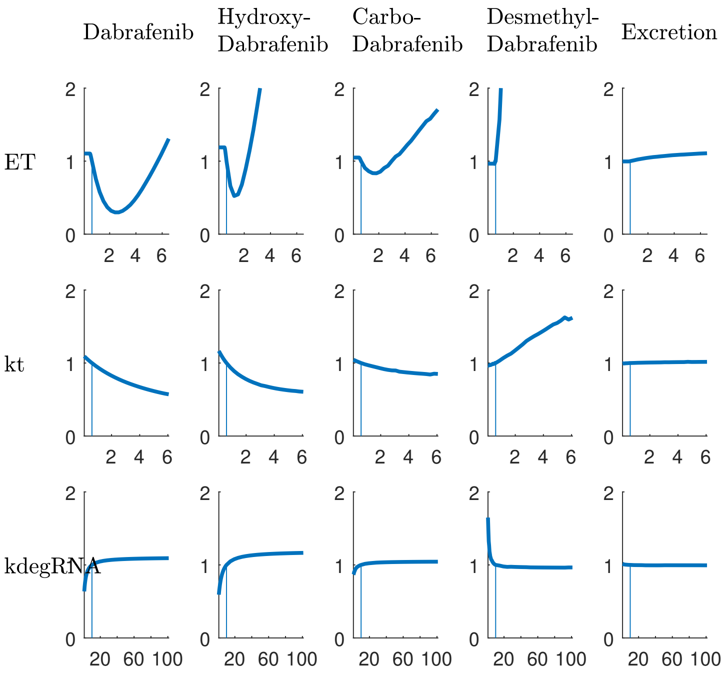

Supplemental Figure 23: Sensitivity Analysis: relative change of fitting quality (RSS) if parameter value is changed.

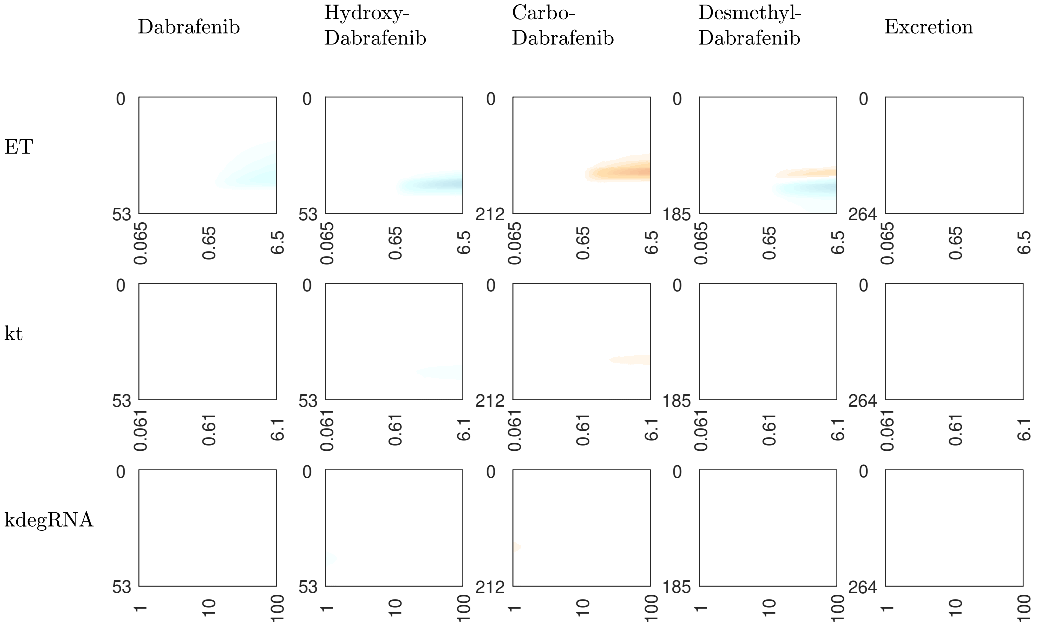

Supplemental Figure 24: Sensitivity Analysis: time course change over different parameter values. Red areas indicate that these periods the time-course is above the original dynamic. Cyan areas indicate that the dynamic descend below the original dynamic.

8.2 Impact on Ketoconazole PK Profile

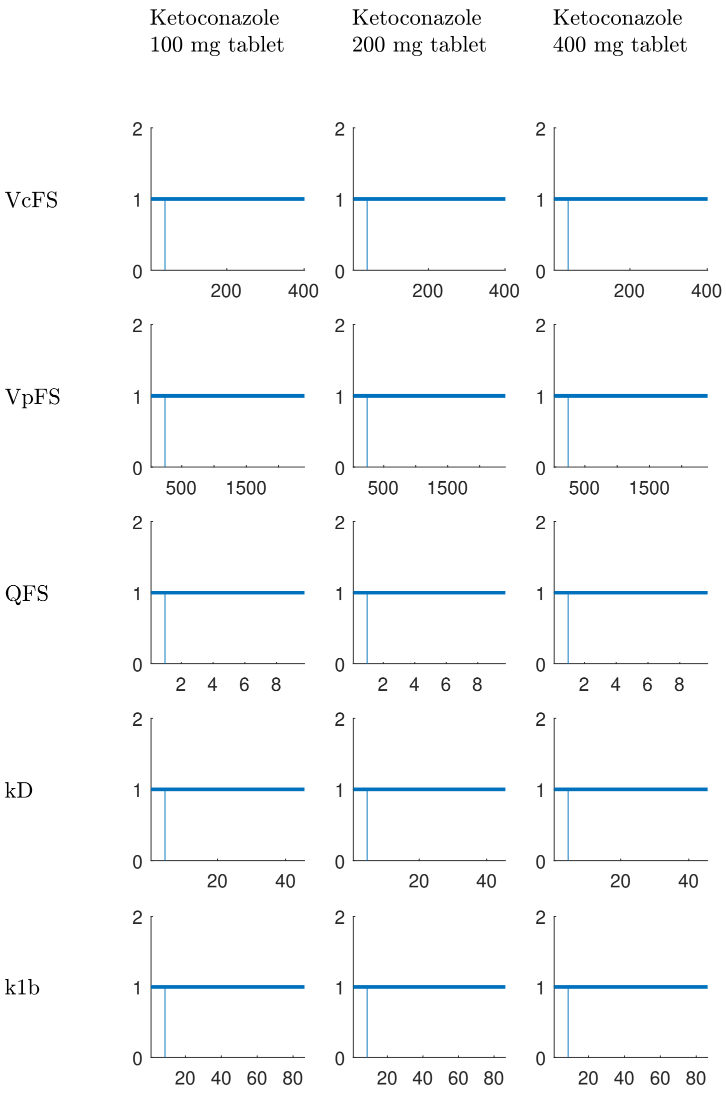

Supplemental Figure 25: Sensitivity Analysis: relative change of fitting quality (RSS) if parameter value is changed.

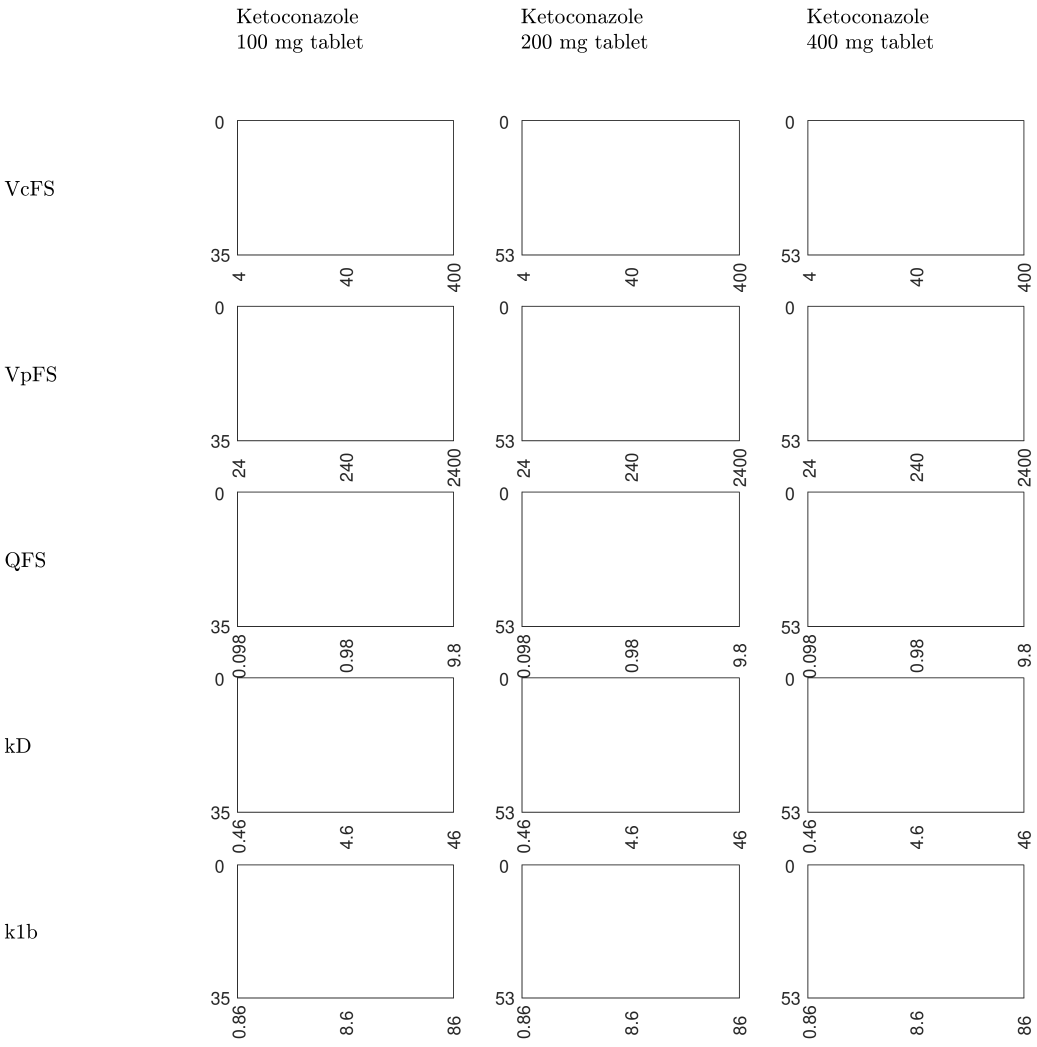

Supplemental Figure 26: Sensitivity Analysis: time course change over different parameter values. Red areas indicate that these periods the time-course is above the original dynamic. Cyan areas indicate that the dynamic descend below the original dynamic.

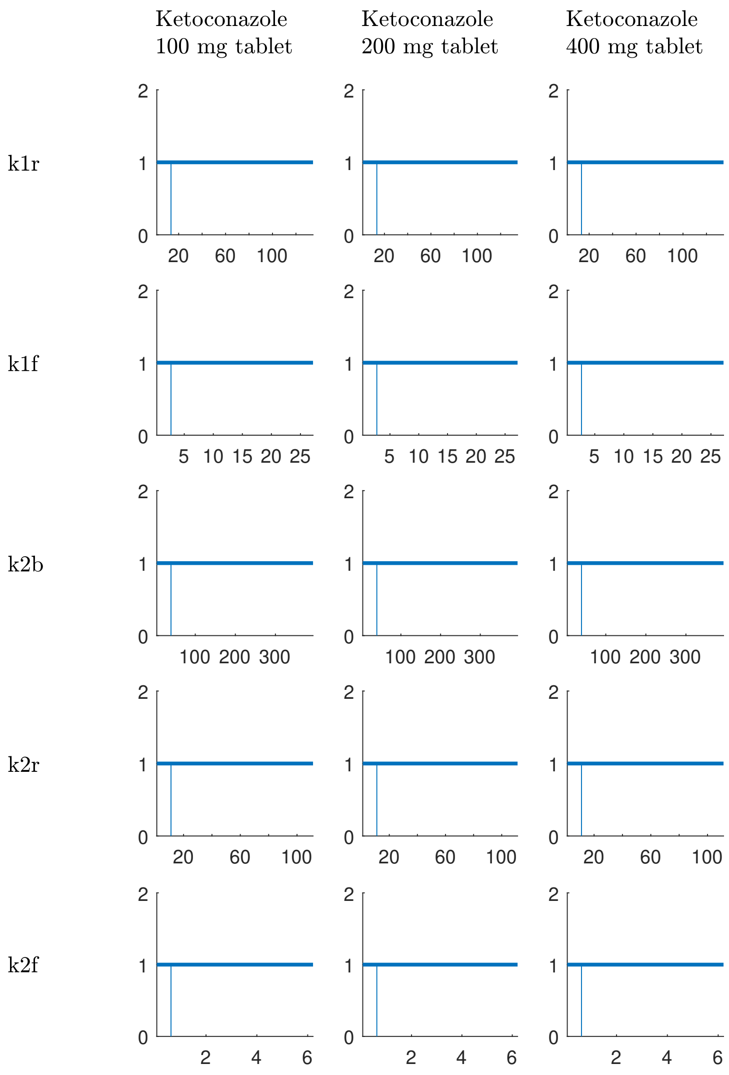

Supplemental Figure 27: Sensitivity Analysis: relative change of fitting quality (RSS) if parameter value is changed.

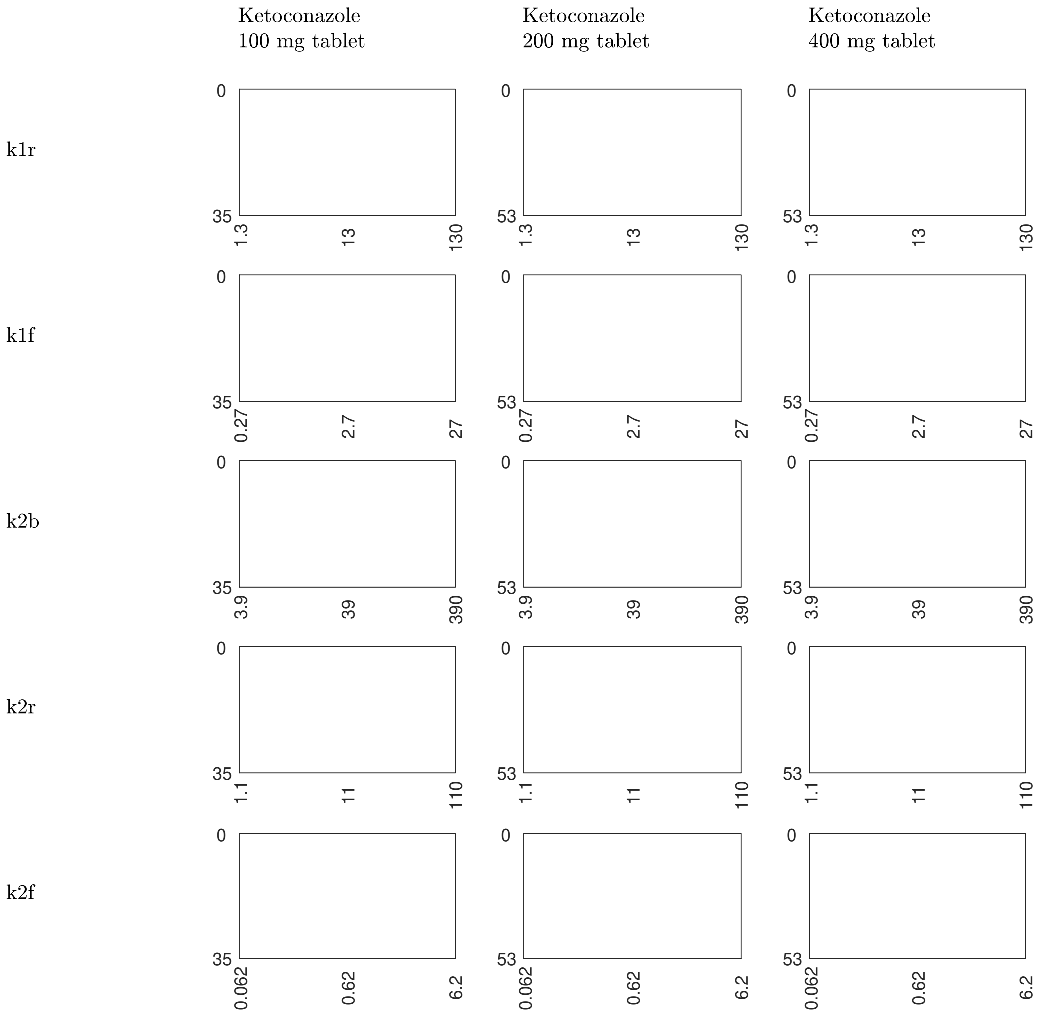

Supplemental Figure 28: Sensitivity Analysis: time course change over different parameter values. Red areas indicate that these periods the time-course is above the original dynamic. Cyan areas indicate that the dynamic descend below the original dynamic.

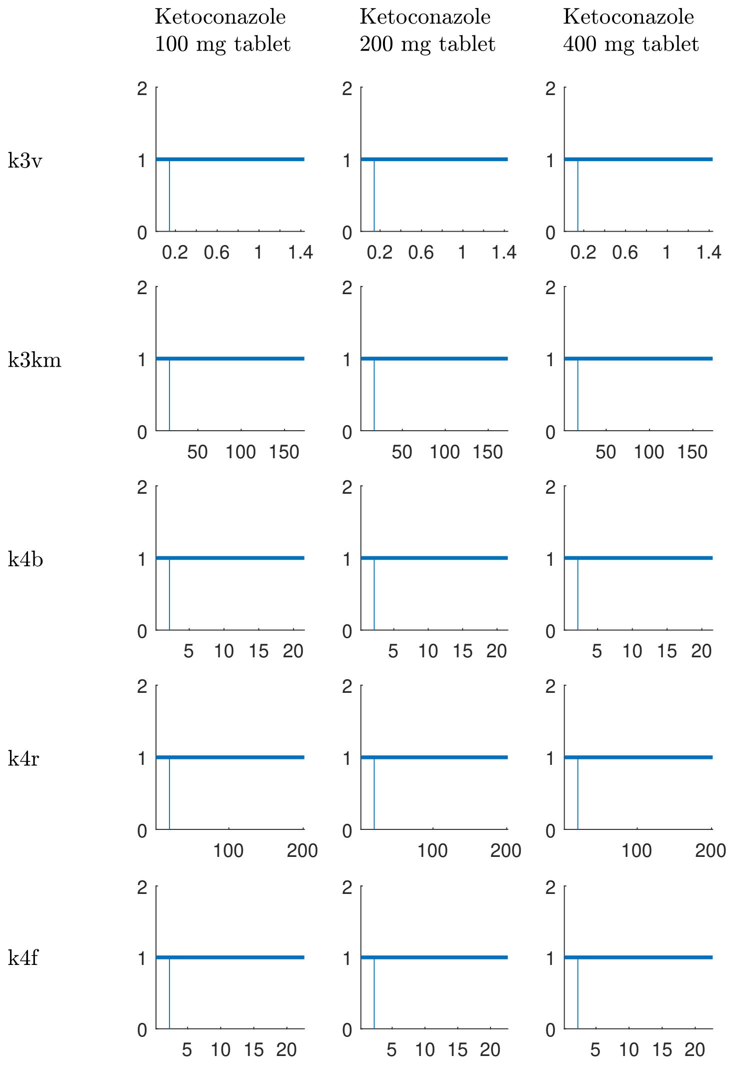

Supplemental Figure 29: Sensitivity Analysis: relative change of fitting quality (RSS) if parameter value is changed.

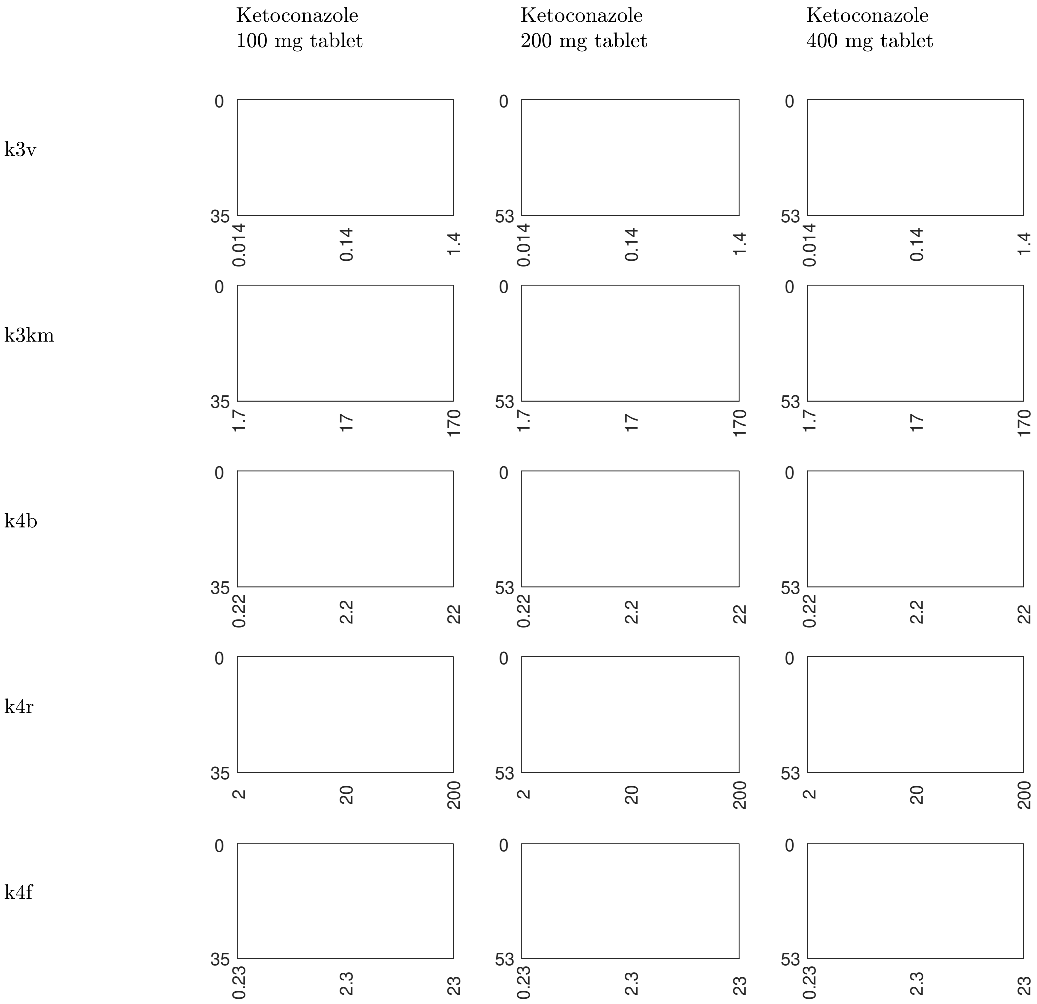

Supplemental Figure 30: Sensitivity Analysis: time course change over different parameter values. Red areas indicate that these periods the time-course is above the original dynamic. Cyan areas indicate that the dynamic descend below the original dynamic.

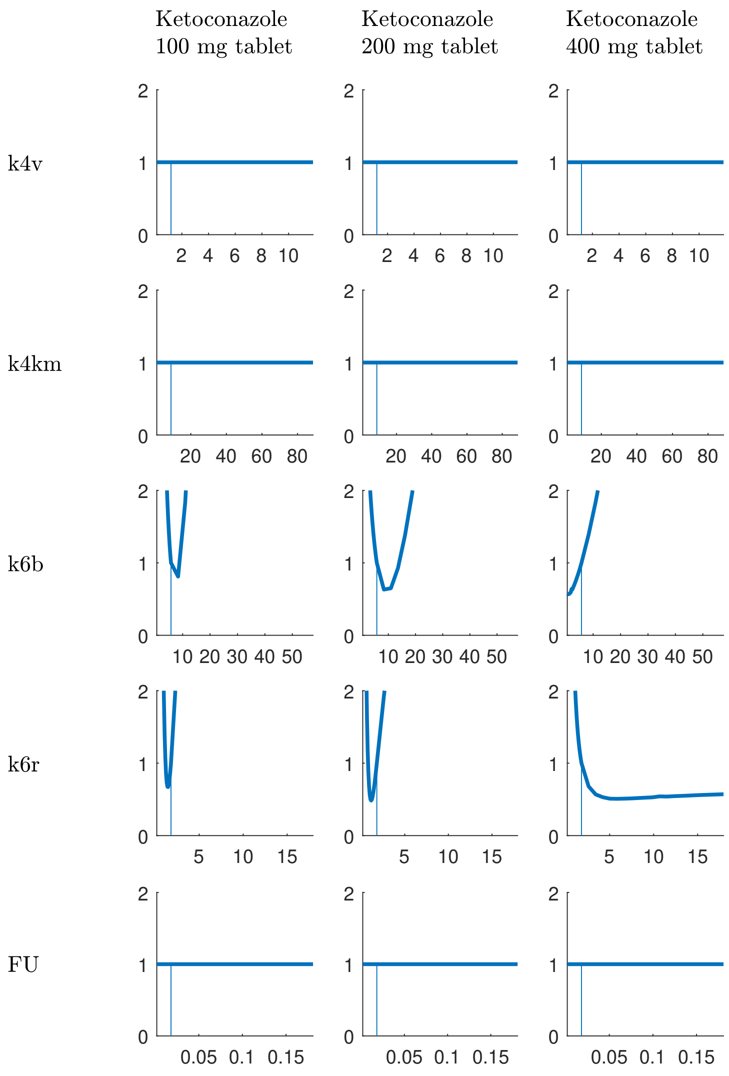

Supplemental Figure 31: Sensitivity Analysis: relative change of fitting quality (RSS) if parameter value is changed.

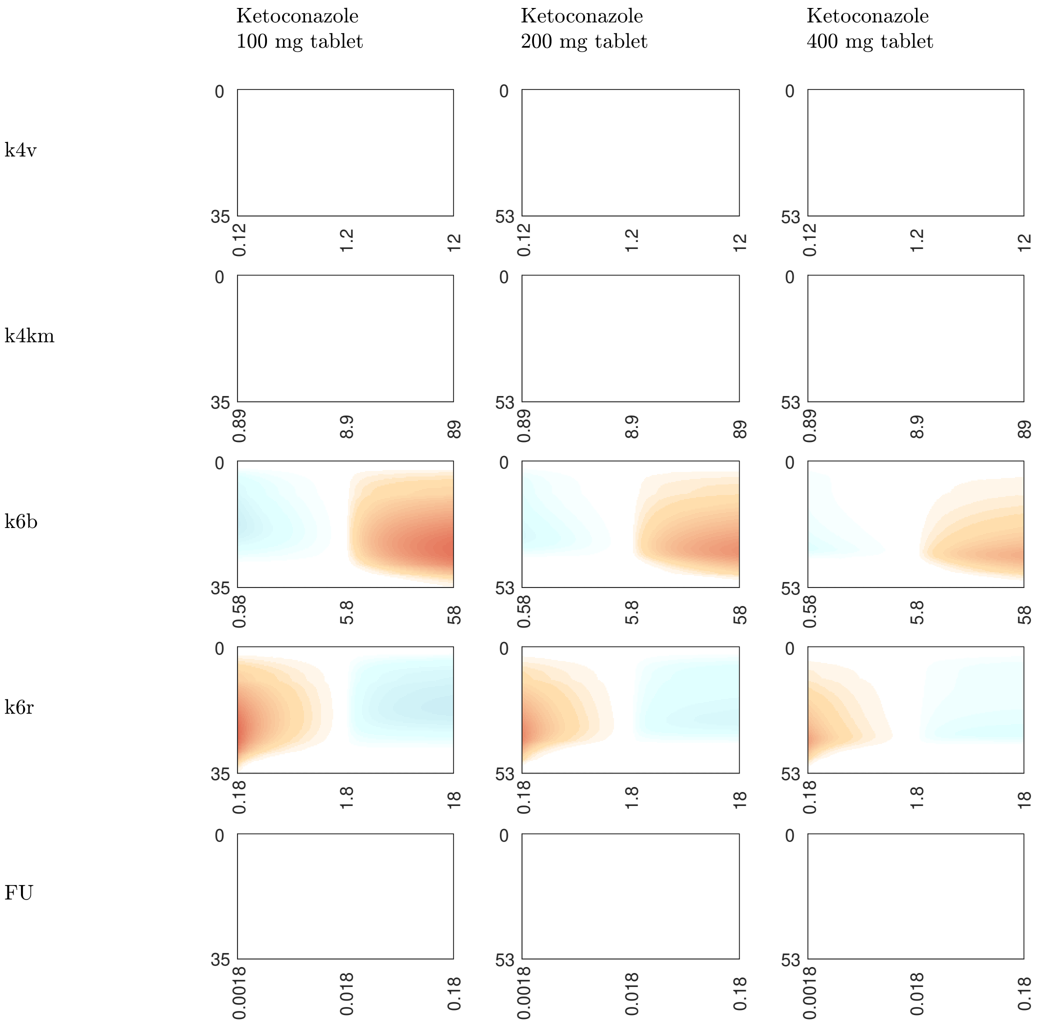

Supplemental Figure 32: Sensitivity Analysis: time course change over different parameter values. Red areas indicate that these periods the time-course is above the original dynamic. Cyan areas indicate that the dynamic descend below the original dynamic.

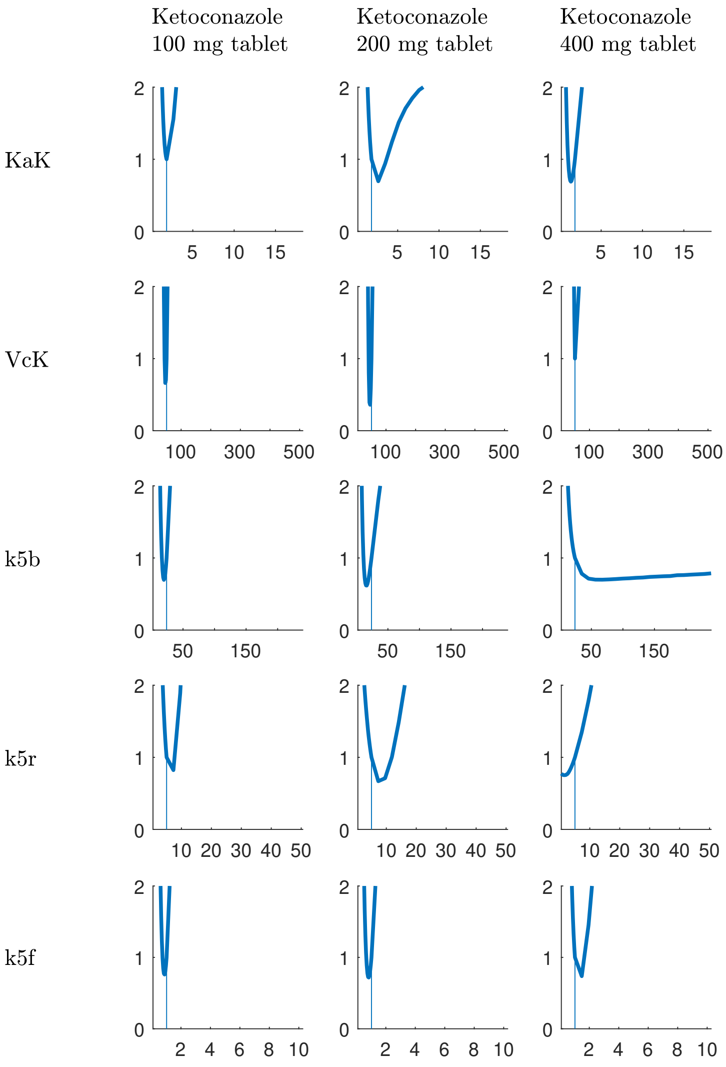

Supplemental Figure 33: Sensitivity Analysis: relative change of fitting quality (RSS) if parameter value is changed.

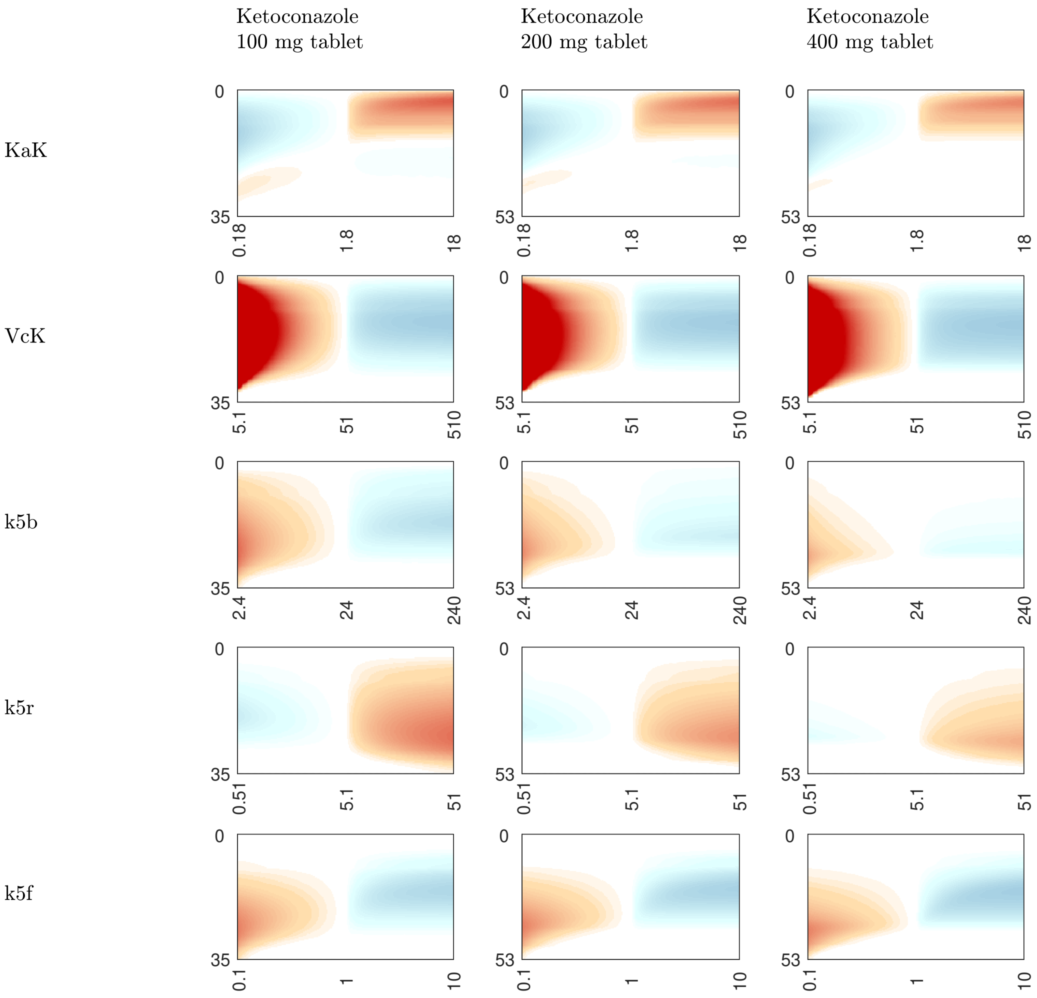

Supplemental Figure 34: Sensitivity Analysis: time course change over different parameter values. Red areas indicate that these periods the time-course is above the original dynamic. Cyan areas indicate that the dynamic descend below the original dynamic.

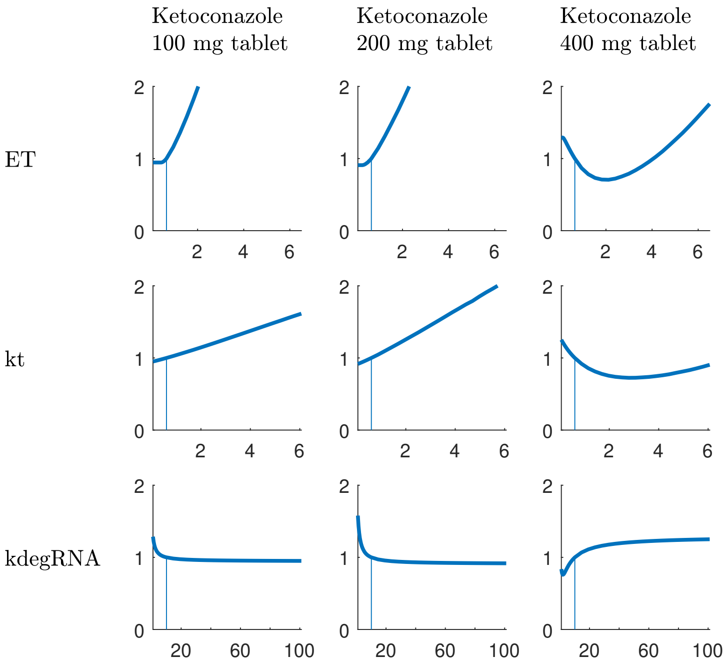

Supplemental Figure 35: Sensitivity Analysis: relative change of fitting quality (RSS) if parameter value is changed.

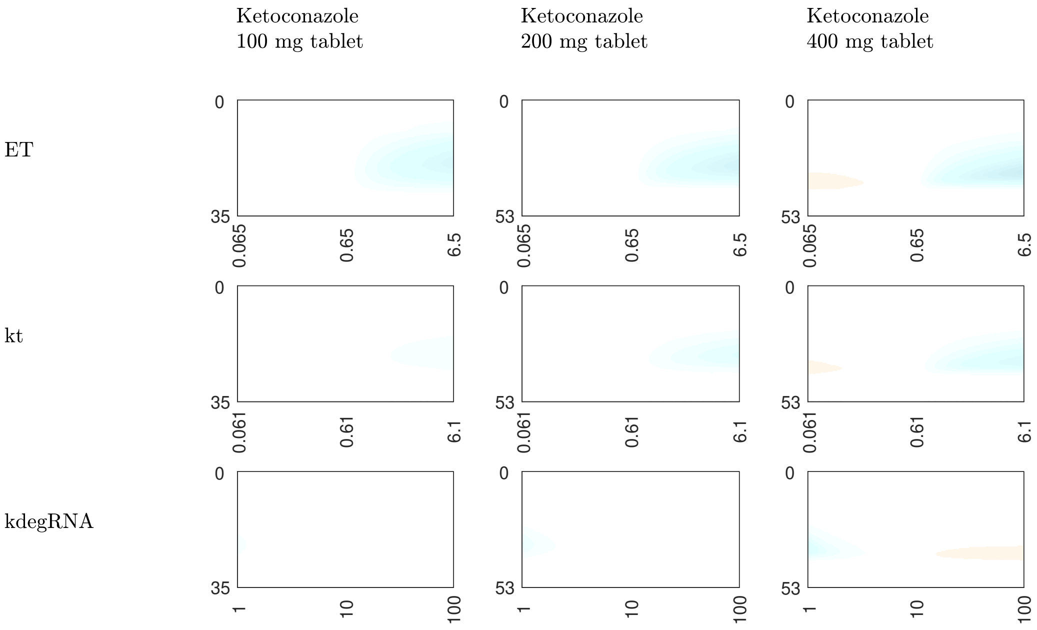

Supplemental Figure 36: Sensitivity Analysis: time course change over different parameter values. Red areas indicate that these periods the time-course is above the original dynamic. Cyan areas indicate that the dynamic descend below the original dynamic.

8.3 Impact on Ketoconazole Dabrafenib Interaction

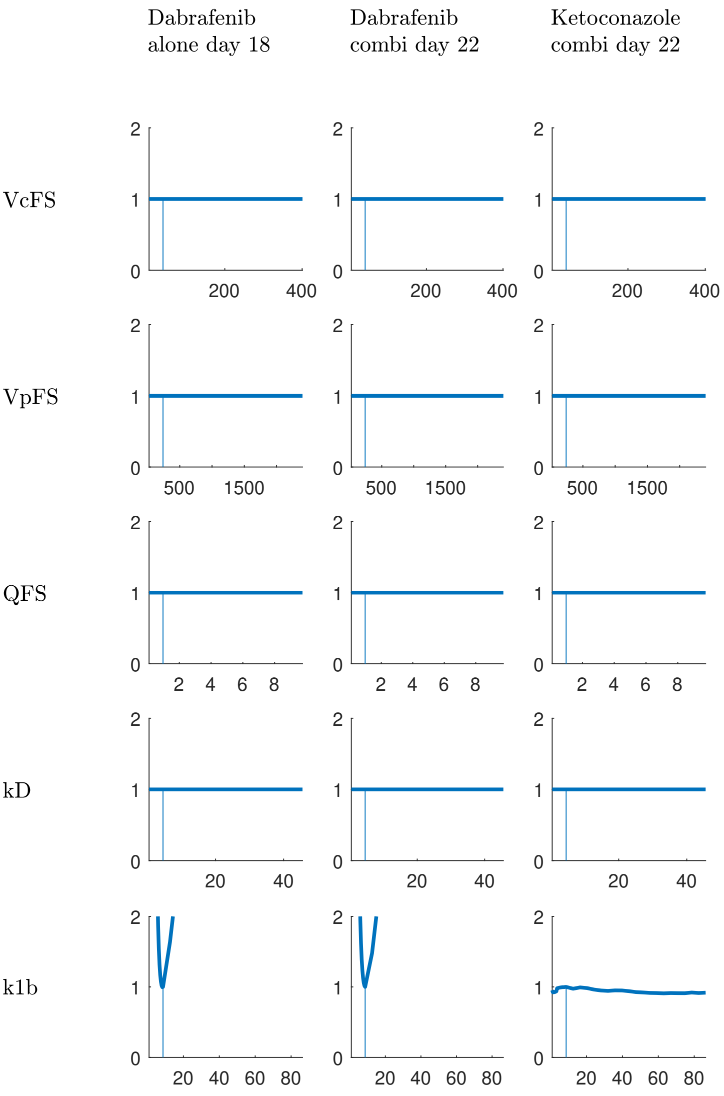

Supplemental Figure 37: Sensitivity Analysis: relative change of fitting quality (RSS) if parameter value is changed.

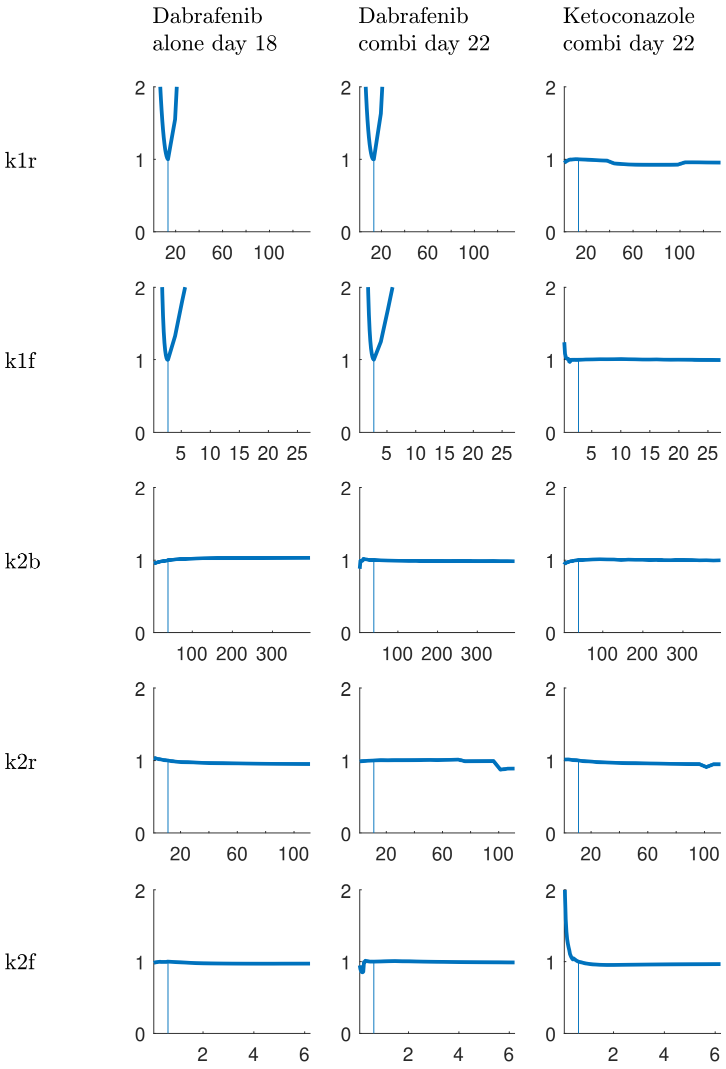

Supplemental Figure 38: Sensitivity Analysis: relative change of fitting quality (RSS) if parameter value is changed.

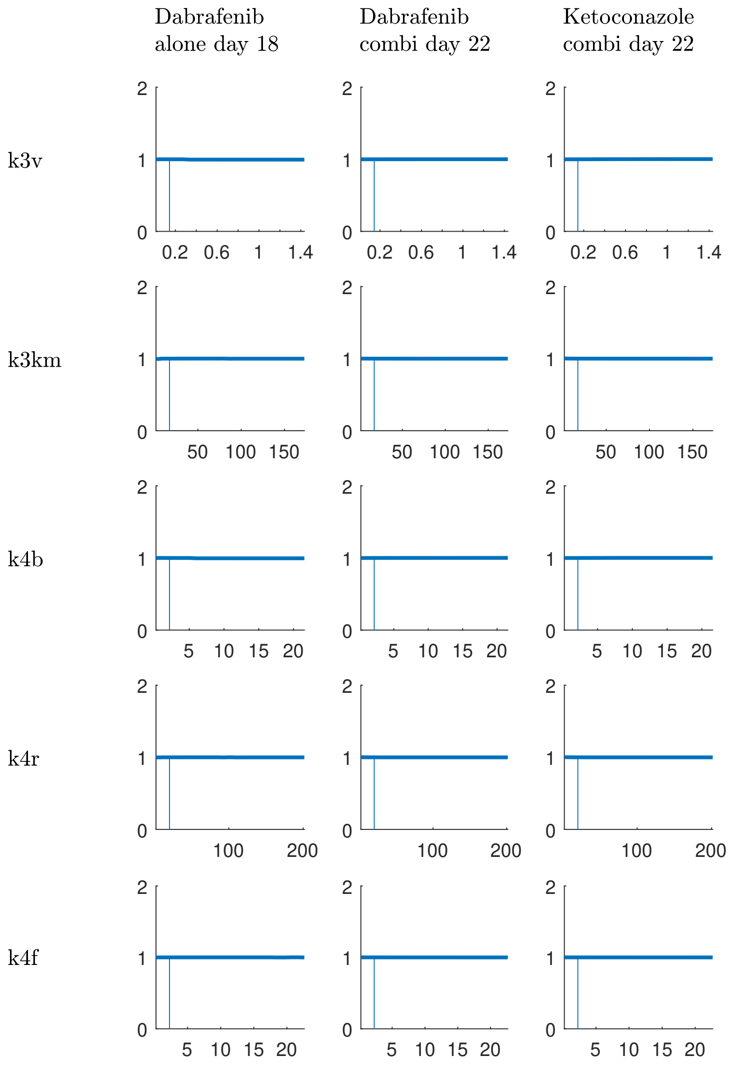

Supplemental Figure 39: Sensitivity Analysis: relative change of fitting quality (RSS) if parameter value is changed.

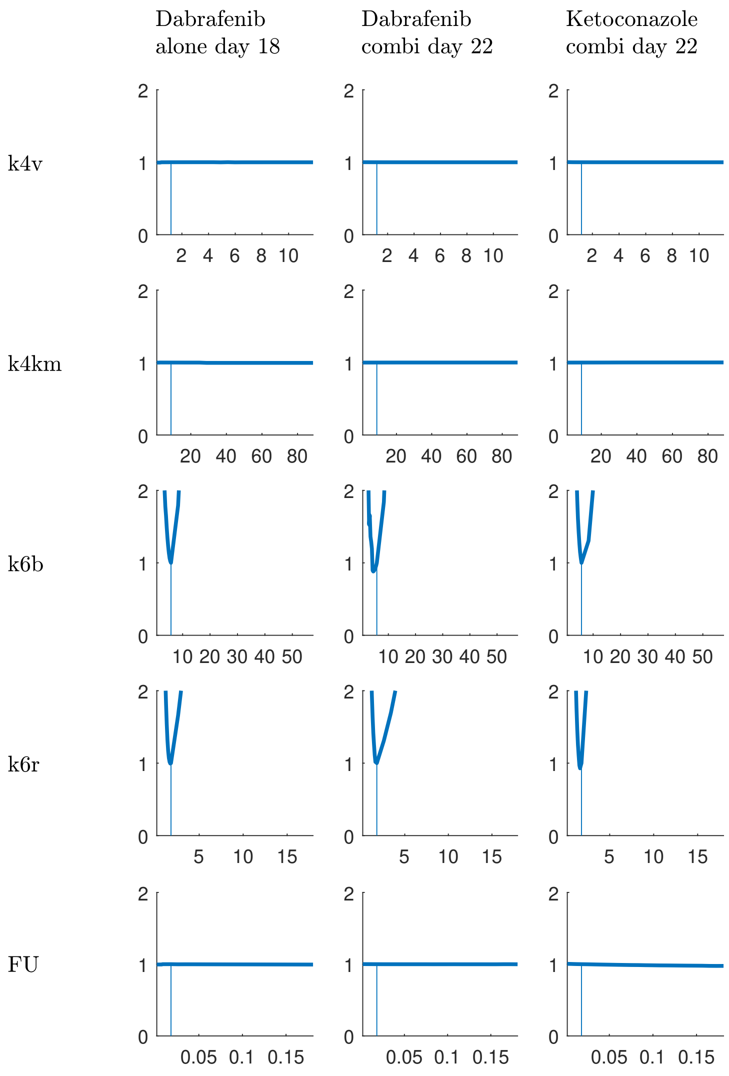

Supplemental Figure 40: Sensitivity Analysis: relative change of fitting quality (RSS) if parameter value is changed.

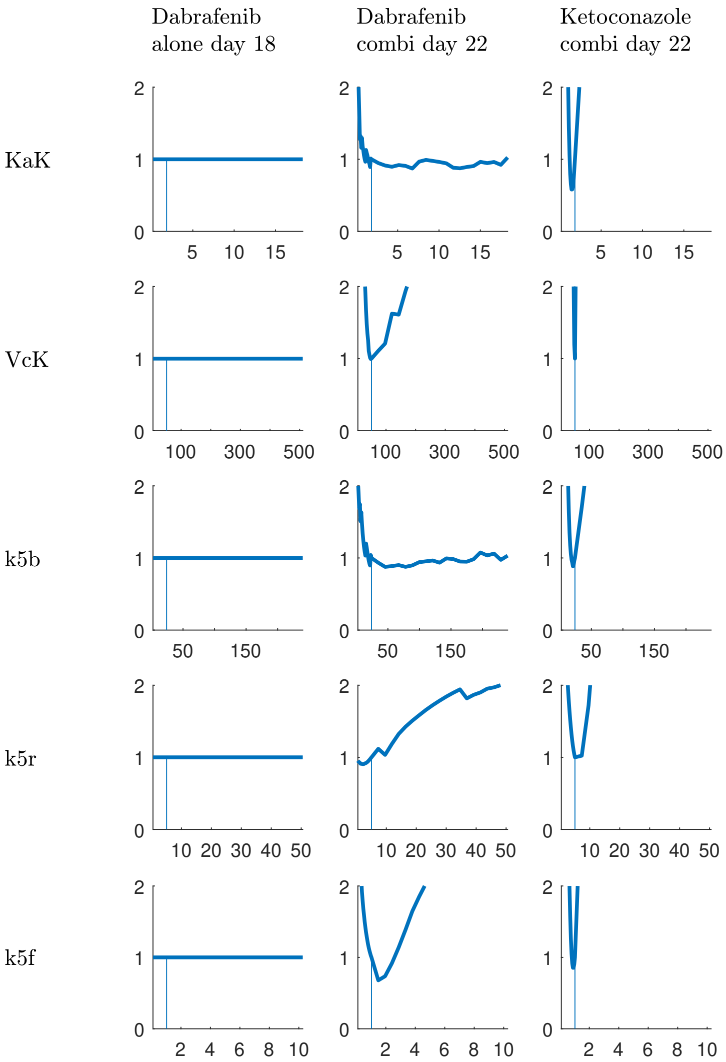

Supplemental Figure 41: Sensitivity Analysis: relative change of fitting quality (RSS) if parameter value is changed.

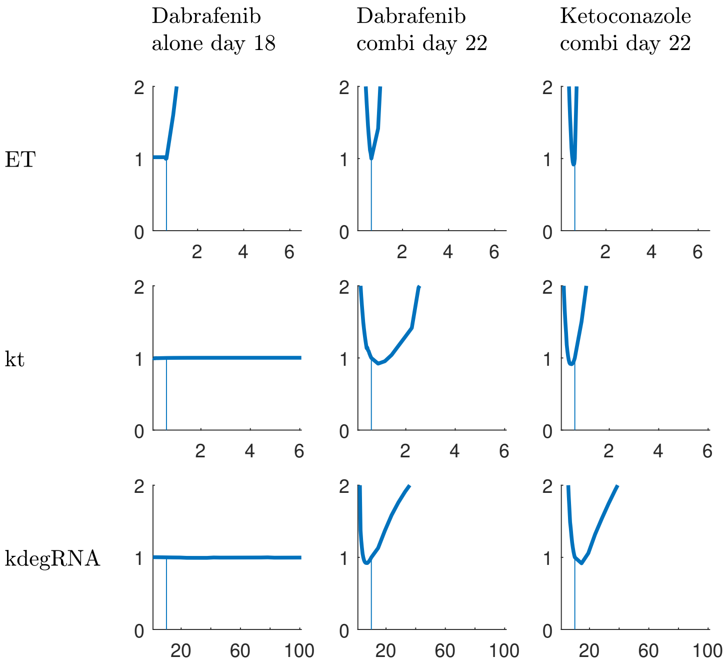

Supplemental Figure 42: Sensitivity Analysis: relative change of fitting quality (RSS) if parameter value is changed.

9 References

[1] Ouellet, D. *et al.* Population pharmacokinetics of dabrafenib, a BRAF inhibitor: effect of dose, time, covariates, and relationship with its metabolites. *J. Clin. Pharmacol.* 54, 696–706 (2014).

[2] Huynh, H., *et al.* Development and validation of a simultaneous quantification method of 14 tyrosine kinase inhibitors in human plasma using LC-MS/MS. *Ther. Drug. Monit.* 39, 43–54 (2017).

[3] Lawrence, S. K., Nguyen, D., Bowen, C., Richards-Peterson, L. & Skordos, K. W. The metabolic drug-drug interaction profile of dabrafenib: in vitro investigations and quantitative extrapolation of the P450-mediated DDI risk *Drug Metab. Dispos.* 42, 1180–1190 (2014).

[4] Bershas, David A *et al.* Metabolism and disposition of oral dabrafenib in cancer patients: proposed participation of aryl nitrogen in carbon-carbon bond cleavage via decarboxylation following enzymatic oxidation. *Drug Metab. Dispos.* 41, 2215–2224 (2013).

[5] Schäuble, S., Stavrum, A. K., Puntervoll, P., Schuster, S. & Heiland, I. Effect of substrate competition in kinetic models of metabolic networks *FEBS Lett.* 587, 2818–2824 (2013).

[6] Bruyere, A. *et al.* Effect of variations in the amounts of P-glycoprotein (ABCB1), BCRP (ABCG2) and CYP3A4 along the human small intestine on PBPK models for predicting intestinal first pass. *Molecular pharmaceutics* 7, 1596–1607 (2010).

[7] Suttle, A. B. *et al.* Assessment of the drug interaction potential and single- and repeat-dose pharmacokinetics of the BRAF inhibitor dabrafenib. *J. Clin. Pharmacol.* 55, 392–400 (2015).

[8] Kim, J.-H., Choi, W.-G., Lee, S. & Lee, H. S. Revisiting the metabolism and bioactivation of ketoconazole in human and mouse using liquid chromatography–mass spectrometry-based metabolomics. *Int. J. Mol. Sci.* 18, 621 (2017).

[9] Heel, R. C. *et al.* Ketoconazole: a review of its therapeutic efficacy in superficial and systemic fungal infections. *Drugs* 23, 1–36 (1982).

[10] Daneshmend, T. K. *et al.* Influence of food on the pharmacokinetics of ketoconazole. *Antimicrob. Agents Chemother.* 25, 1–3 (1984).
