# Supplementary material for: Mechanistically Coupled PK (MCPK) Model to Describe Enzyme Induction and Occupancy Dependent DDI of Dabrafenib Metabolism
Source: Pharmaceutics. 2022 Jan 28;14(2):310. doi: 10.3390/pharmaceutics14020310 (PMC8875124; doi:10.3390/pharmaceutics14020310)
Supplement: Supplementary file 1 [file pharmaceutics-14-00310-s001.zip › pharmaceutics-1479084-supplementary/Supplementary_Information/Supplemental_Information_S2.pdf]

Supplemental Information S2: Mechanistically Coupled PK (MCPK) model to describe enzyme induction and occupancy dependent DDI of dabrafenib metabolism

Marco Albrecht[1,2], Yuri Kogan[3], Dagmar Kulms[4], and Thomas Sauter[5]

[1]*Université du Luxembourg; Systems Biology Group; 4367 Belvaux; Luxembourg; Email: marco.albrecht@posteo.eu*
[2]*esqLABS GmbH; 26683 Saterland; Germany; Email: marco.albrecht@esqlabs.com*
[3]*Institute for Medical Biomathematics (IMBM); 60991 Bnei Atarot; Israel; Email: yuri@imbm.com*
[4]*Technical University of Dresden; Experimental Dermatology; 01307 Dresden; Germany; Tel.: +49 (0)351 458-18973 ; Email: dagmar.kulms@uniklinikum-dresden.de*
[5]*Université du Luxembourg; Systems Biology Group; 4367 Belvaux; Luxembourg; Tel.: (+352) 46 66 44 6296; Email: thomas.sauter@uni.lu*

Spheroid Image Data used for Paper Figure 6

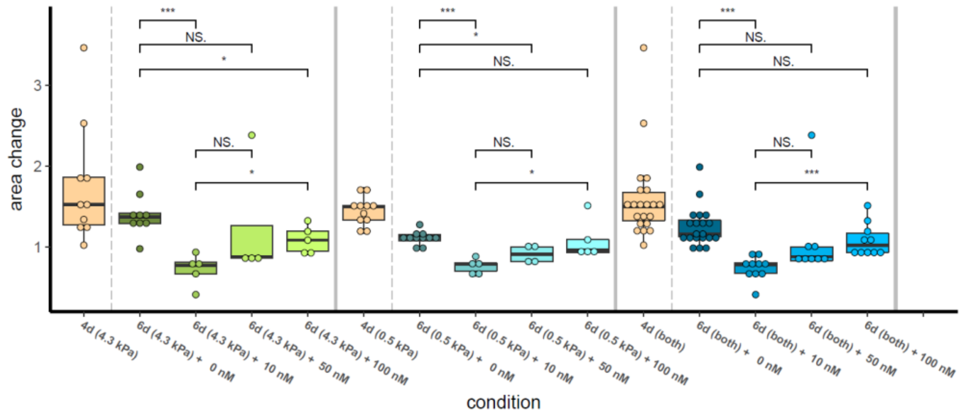

Legend

CD: Cell degradable gel
R1-4: Biological Replicate Series 1 to 4.
XCD2/6: X is one spheroid of a Biological Replicate Series
451LU: Metastatic Melanoma Cell Line with **green** flourecent protein
PI: Propidium iodide as a **red** cell death marker at day 6
DMSO: Dimethyl sulphoxide.

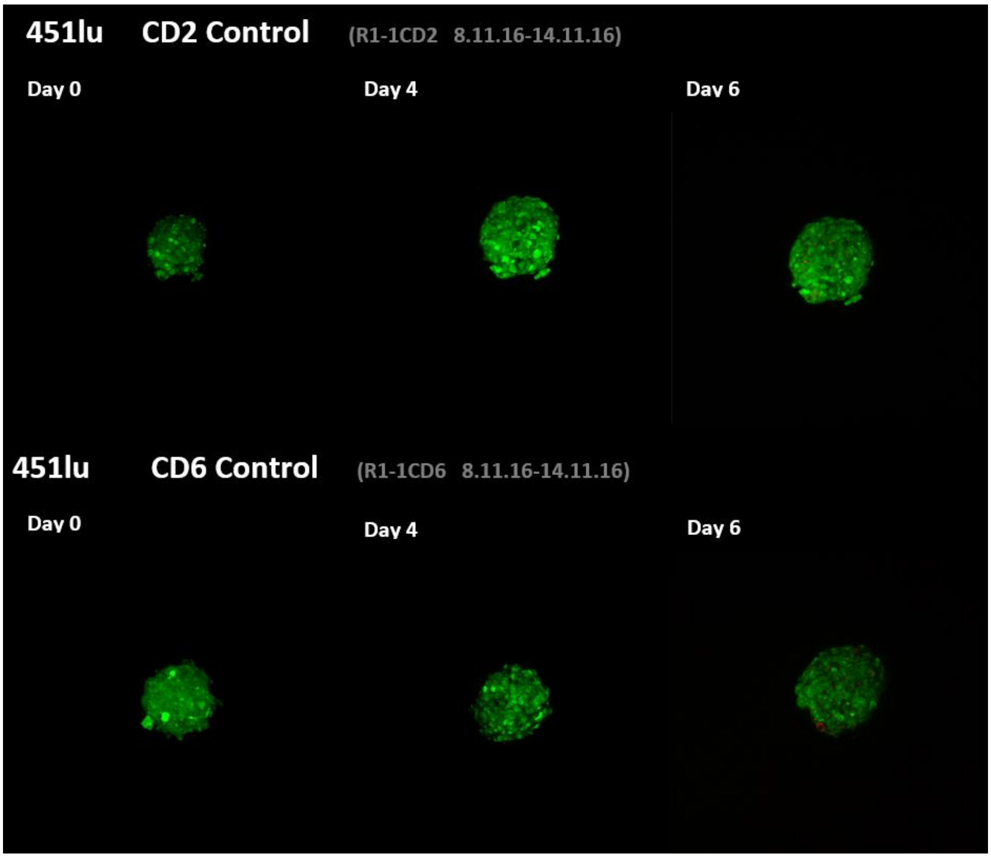
451lu CD2 Control (R1-1CD2 8.11.16-14.11.16)
Day 0
Day 4
Day 6
451lu CD6 Control (R1-1CD6 8.11.16-14.11.16)
Day 0
Day 4
Day 6

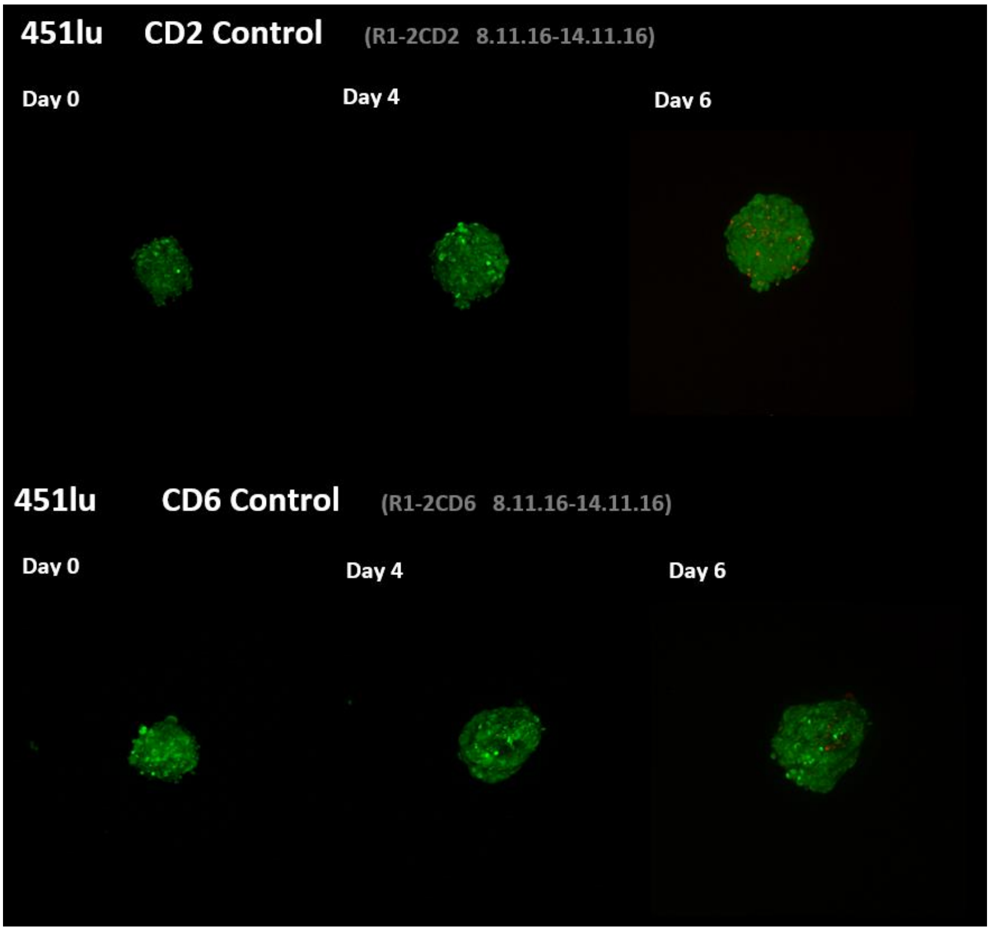
451lu CD2 Control (R1-2CD2 8.11.16-14.11.16)
Day 0
Day 4
Day 6
451lu CD6 Control (R1-2CD6 8.11.16-14.11.16)
Day 0
Day 4
Day 6

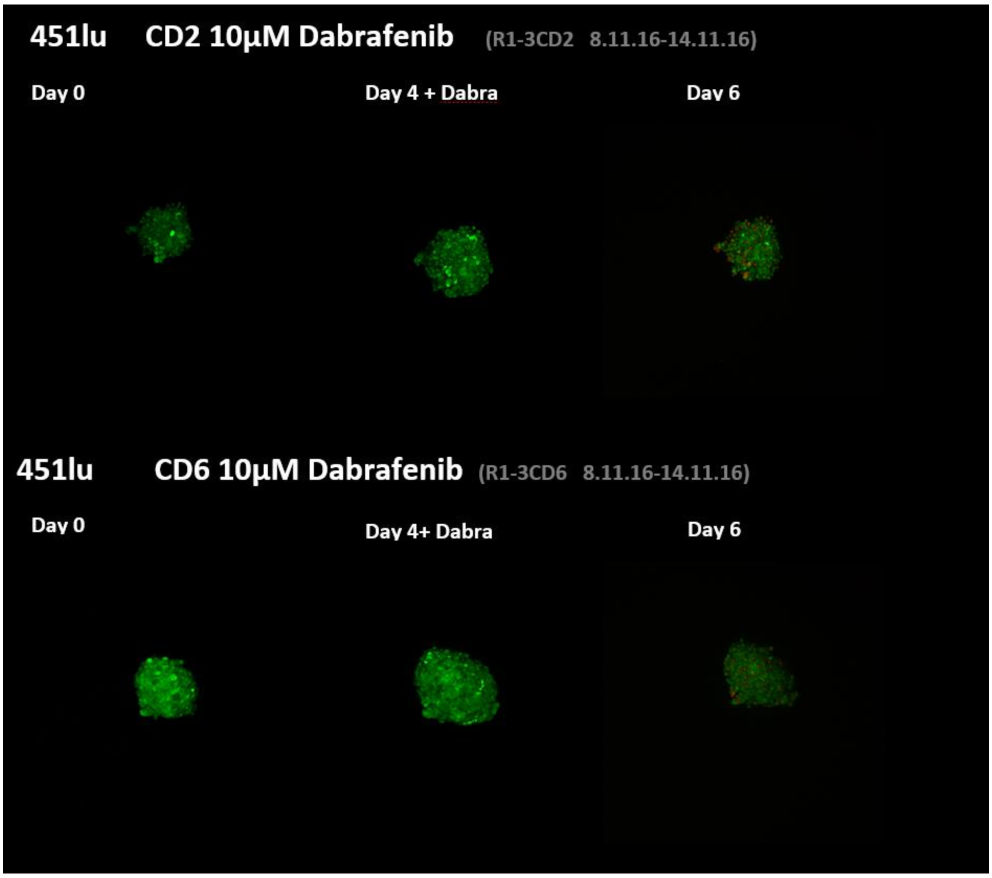
451lu CD2 10µM Dabrafenib (R1-3CD2 8.11.16-14.11.16)
Day 0
Day 4 + Dabra
Day 6
451lu CD6 10µM Dabrafenib (R1-3CD6 8.11.16-14.11.16)
Day 0
Day 4+ Dabra
Day 6

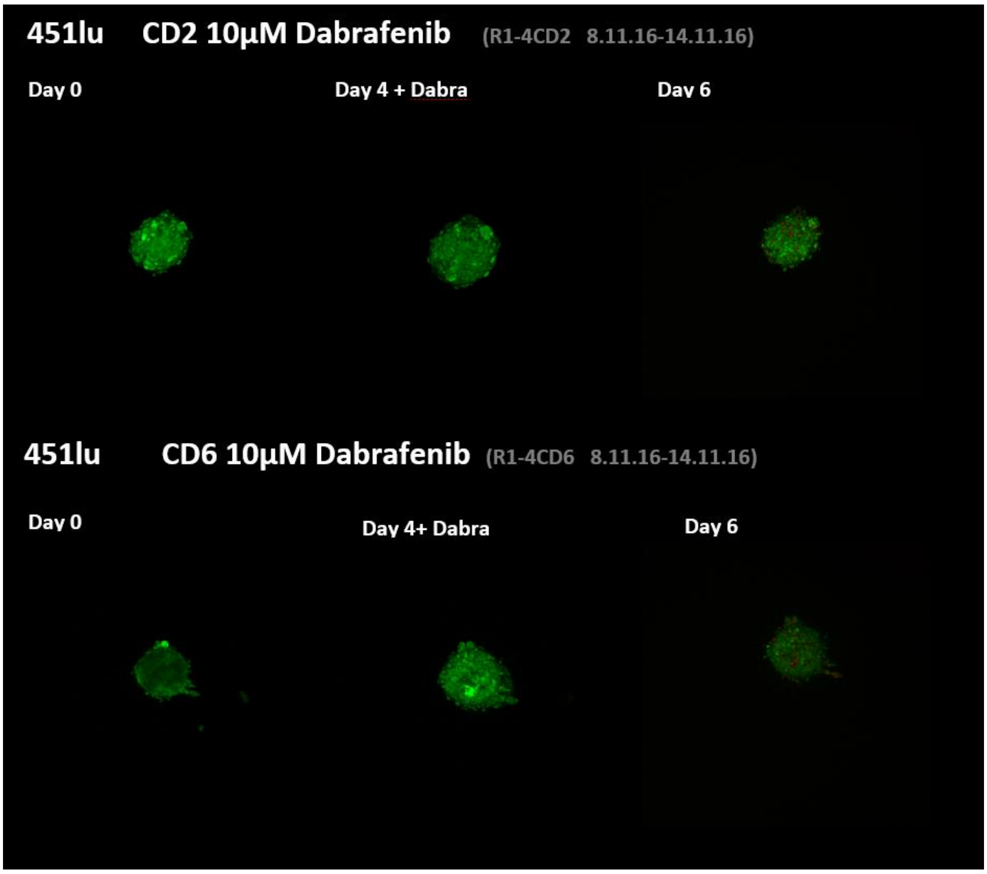
451lu CD2 10µM Dabrafenib (R1-4CD2 8.11.16-14.11.16)
Day 0
Day 4 + Dabra
Day 6
451lu CD6 10µM Dabrafenib (R1-4CD6 8.11.16-14.11.16)
Day 0
Day 4+ Dabra
Day 6

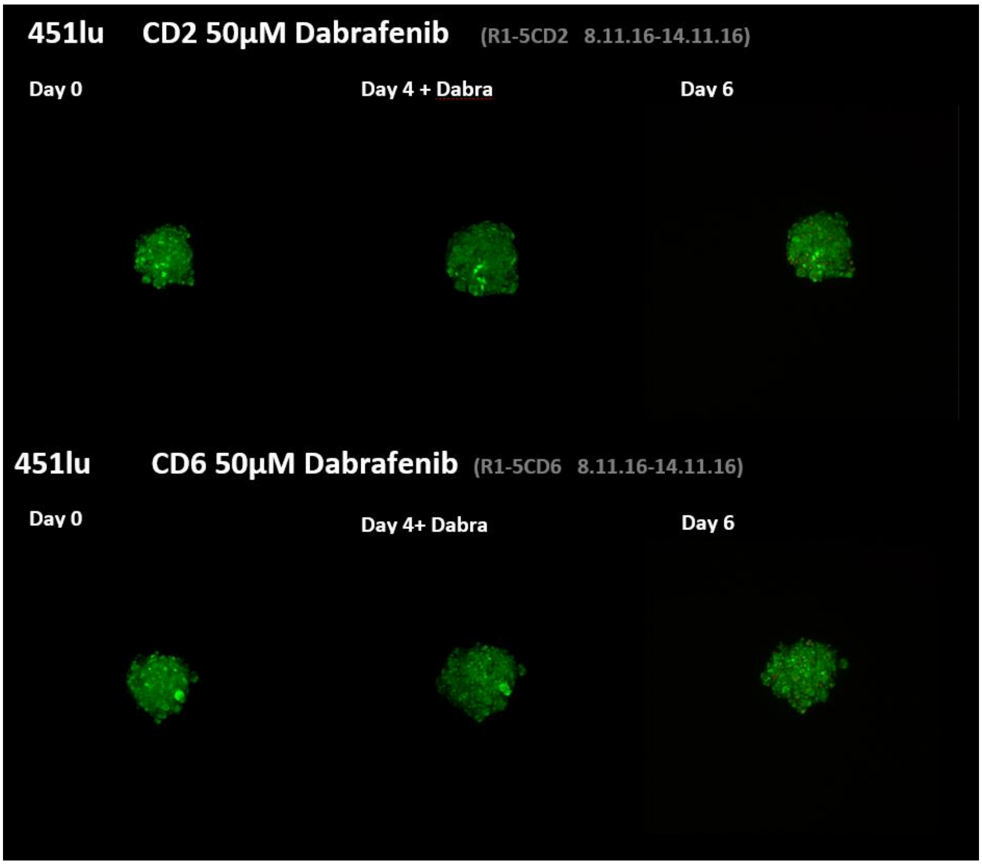
451lu CD2 50µM Dabrafenib (R1-5CD2 8.11.16-14.11.16)
Day 0
Day 4 + Dabra
Day 6
451lu CD6 50µM Dabrafenib (R1-5CD6 8.11.16-14.11.16)
Day 0
Day 4+ Dabra
Day 6

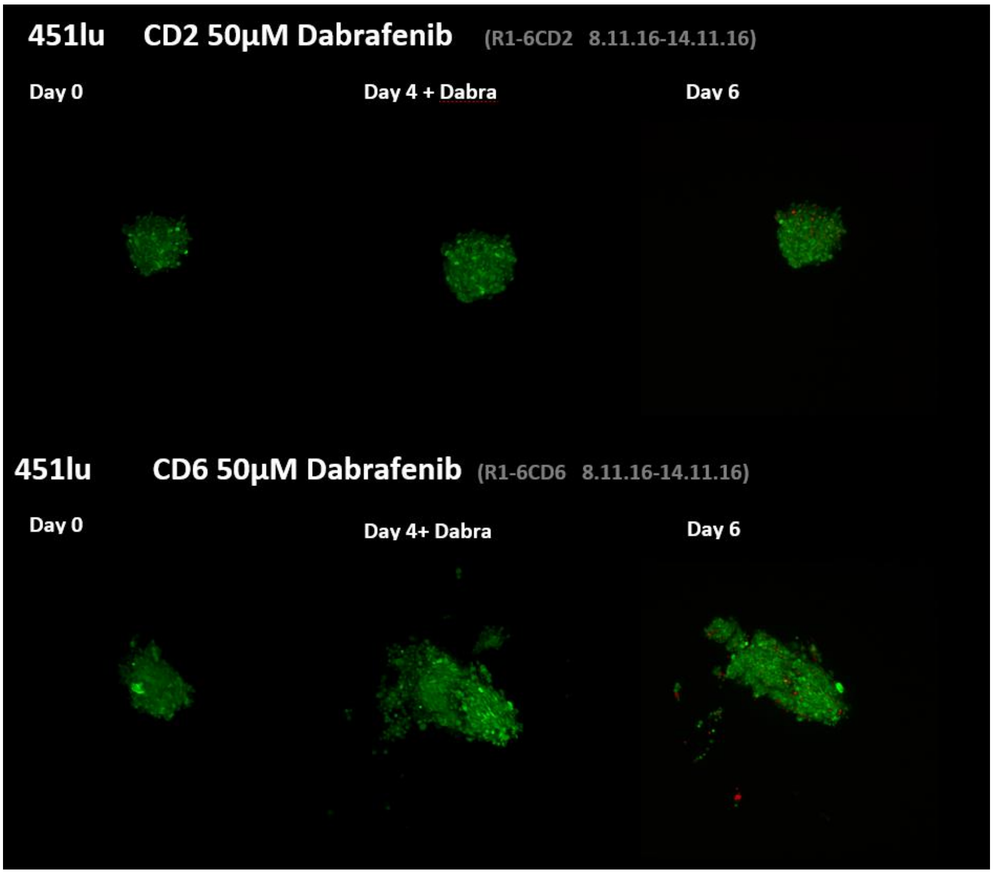
451lu CD2 50µM Dabrafenib (R1-6CD2 8.11.16-14.11.16)
Day 0
Day 4 + Dabra
Day 6
451lu CD6 50µM Dabrafenib (R1-6CD6 8.11.16-14.11.16)
Day 0
Day 4+ Dabra
Day 6

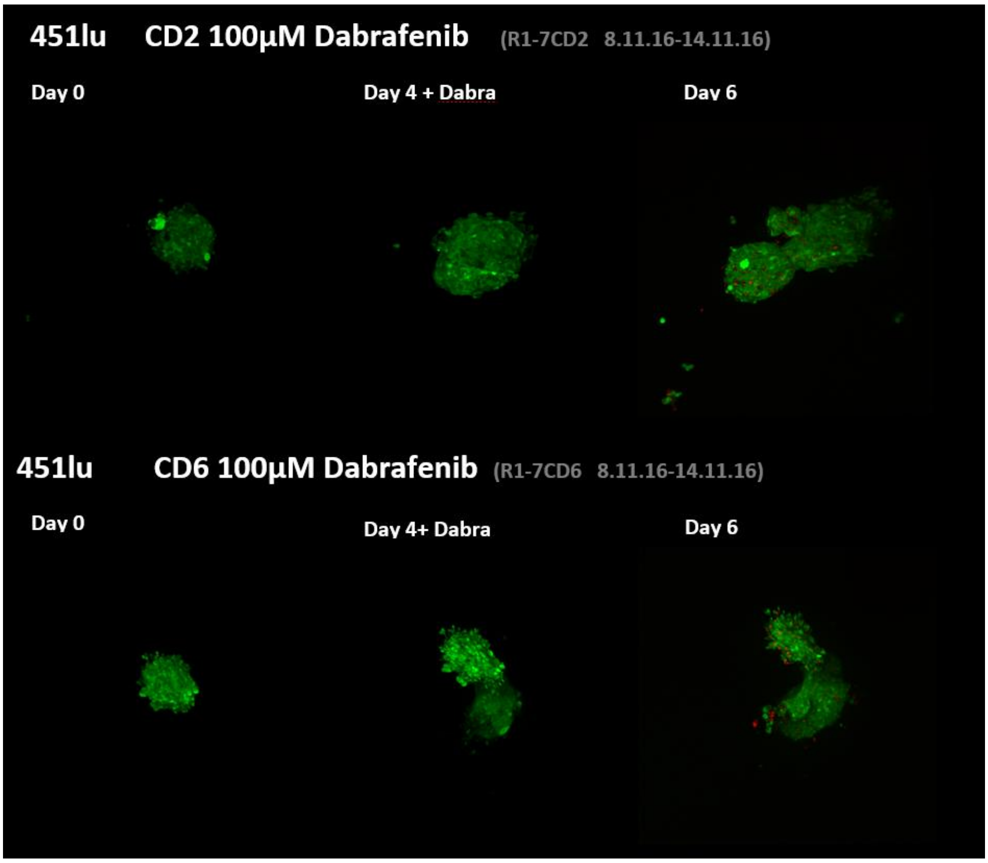
451lu CD2 100µM Dabrafenib (R1-7CD2 8.11.16-14.11.16)
Day 0
Day 4 + Dabra
Day 6
451lu CD6 100µM Dabrafenib (R1-7CD6 8.11.16-14.11.16)
Day 0
Day 4+ Dabra
Day 6

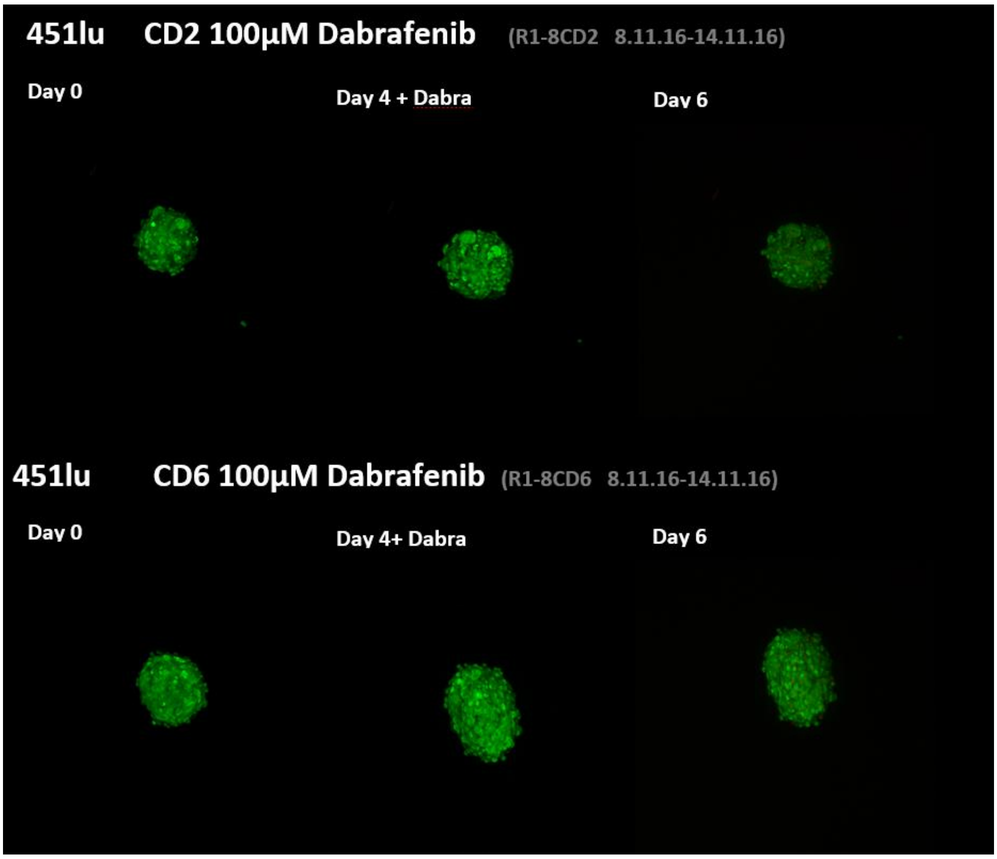
451lu CD2 100µM Dabrafenib (R1-8CD2 8.11.16-14.11.16)
Day 0
Day 4 + Dabra
Day 6
451lu CD6 100µM Dabrafenib (R1-8CD6 8.11.16-14.11.16)
Day 0
Day 4+ Dabra
Day 6

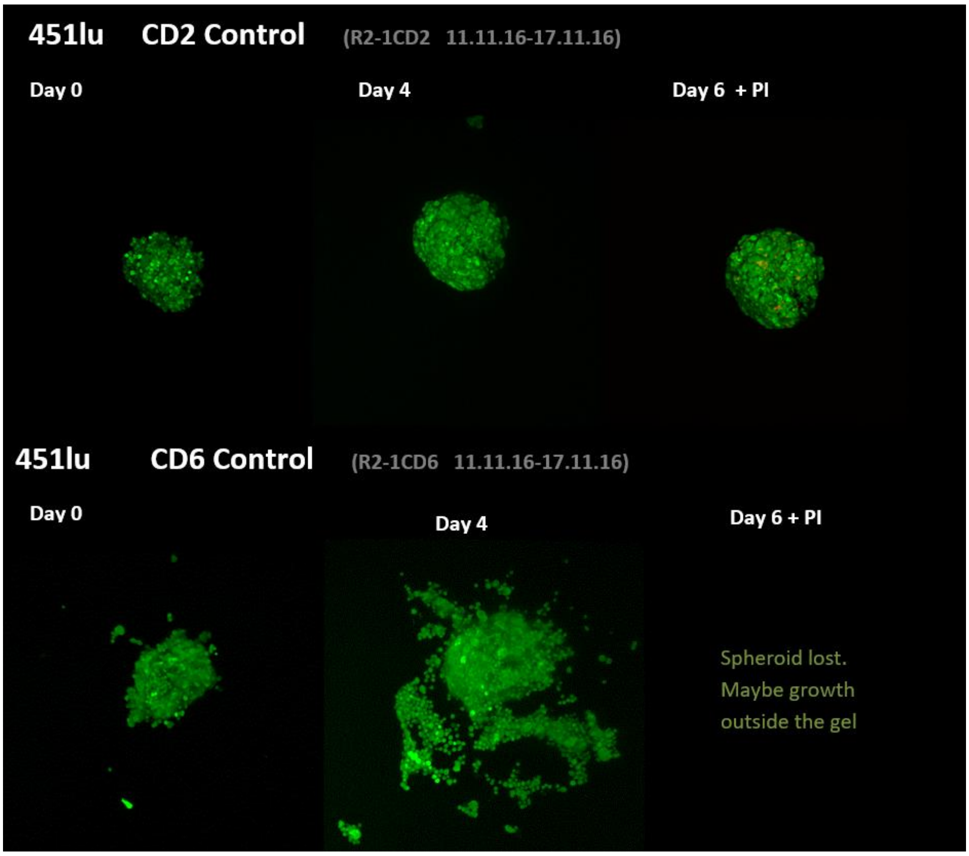
451lu CD2 Control (R2-1CD2 11.11.16-17.11.16)
Day 0
Day 4
Day 6 + PI
451lu CD6 Control (R2-1CD6 11.11.16-17.11.16)
Day 0
Day 4
Day 6 + PI
Spheroid lost.
Maybe growth
outside the gel

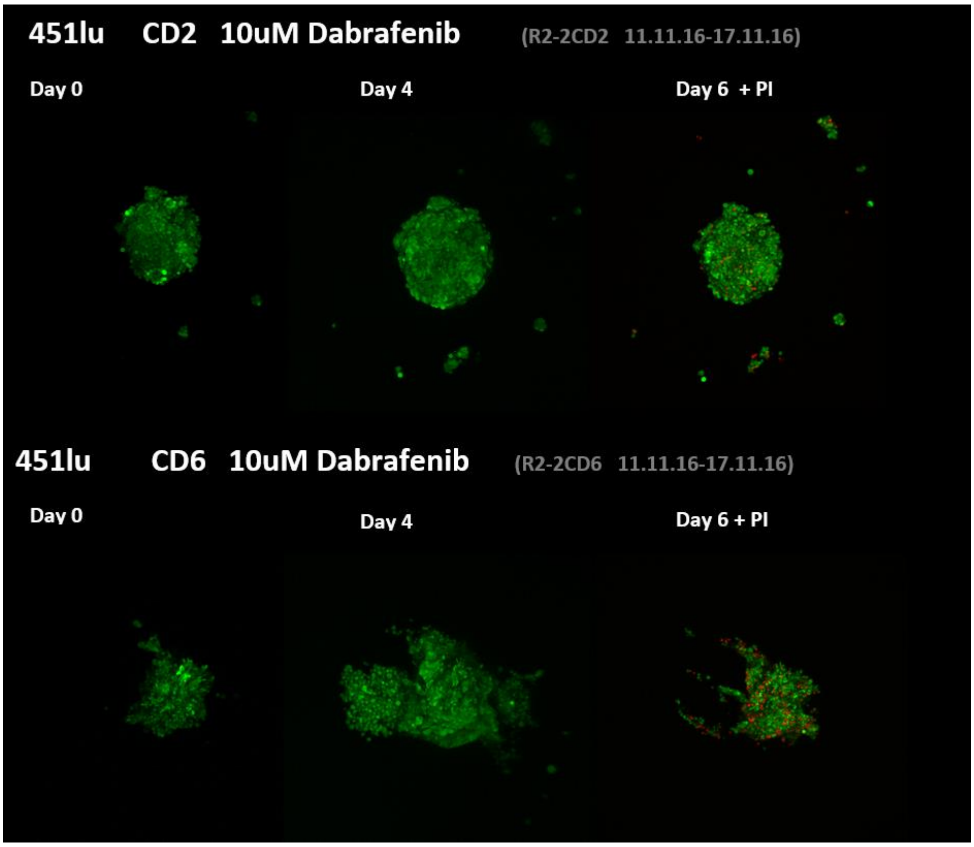
451lu CD2 10uM Dabrafenib (R2-2CD2 11.11.16-17.11.16)
Day 0
Day 4
Day 6 + PI
451lu CD6 10uM Dabrafenib (R2-2CD6 11.11.16-17.11.16)
Day 0
Day 4
Day 6 + PI

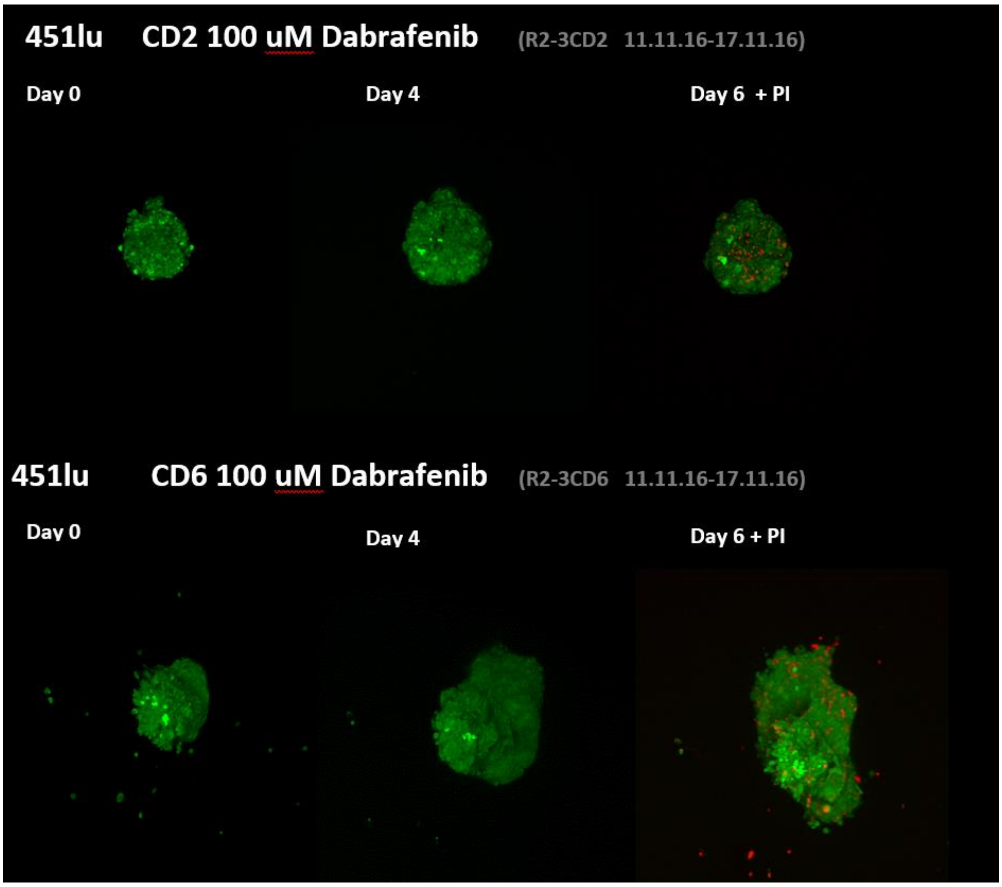
451lu CD2 100 uM Dabrafenib (R2-3CD2 11.11.16-17.11.16)
Day 0
Day 4
Day 6 + PI
451lu CD6 100 uM Dabrafenib (R2-3CD6 11.11.16-17.11.16)
Day 0
Day 4
Day 6 + PI

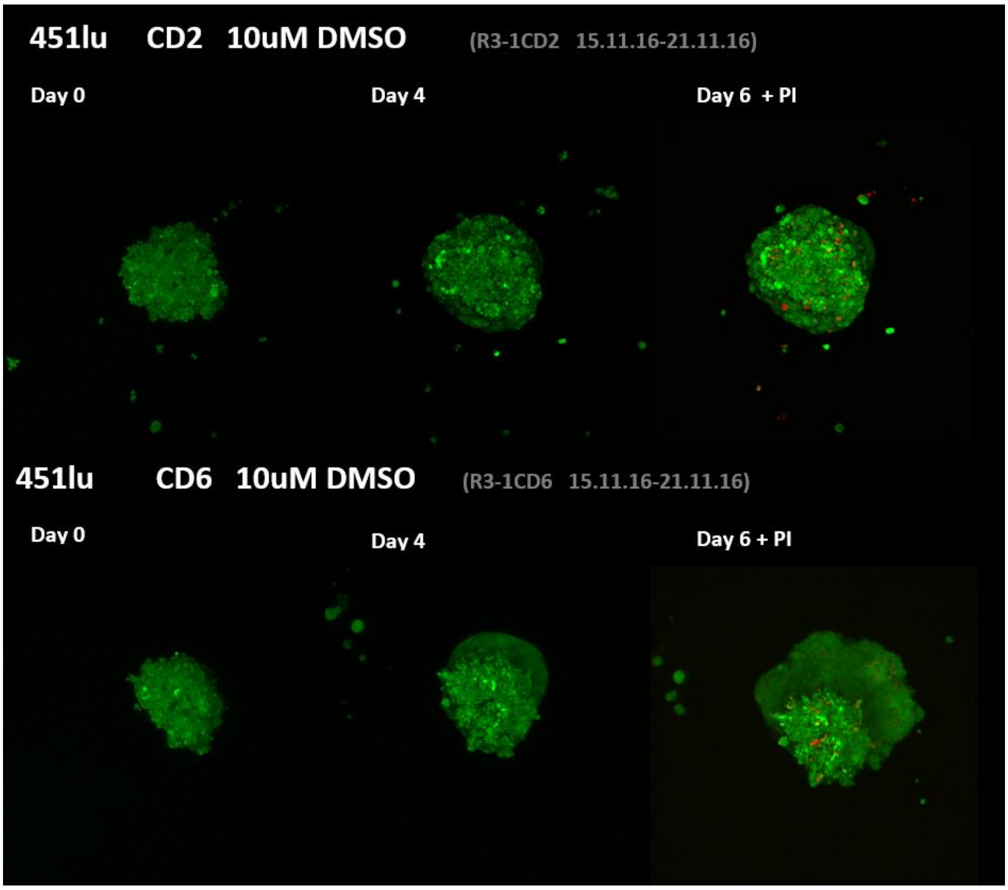
451lu CD2 10uM DMSO (R3-1CD2 15.11.16-21.11.16)
Day 0
Day 4
Day 6 + PI
451lu CD6 10uM DMSO (R3-1CD6 15.11.16-21.11.16)
Day 0
Day 4
Day 6 + PI

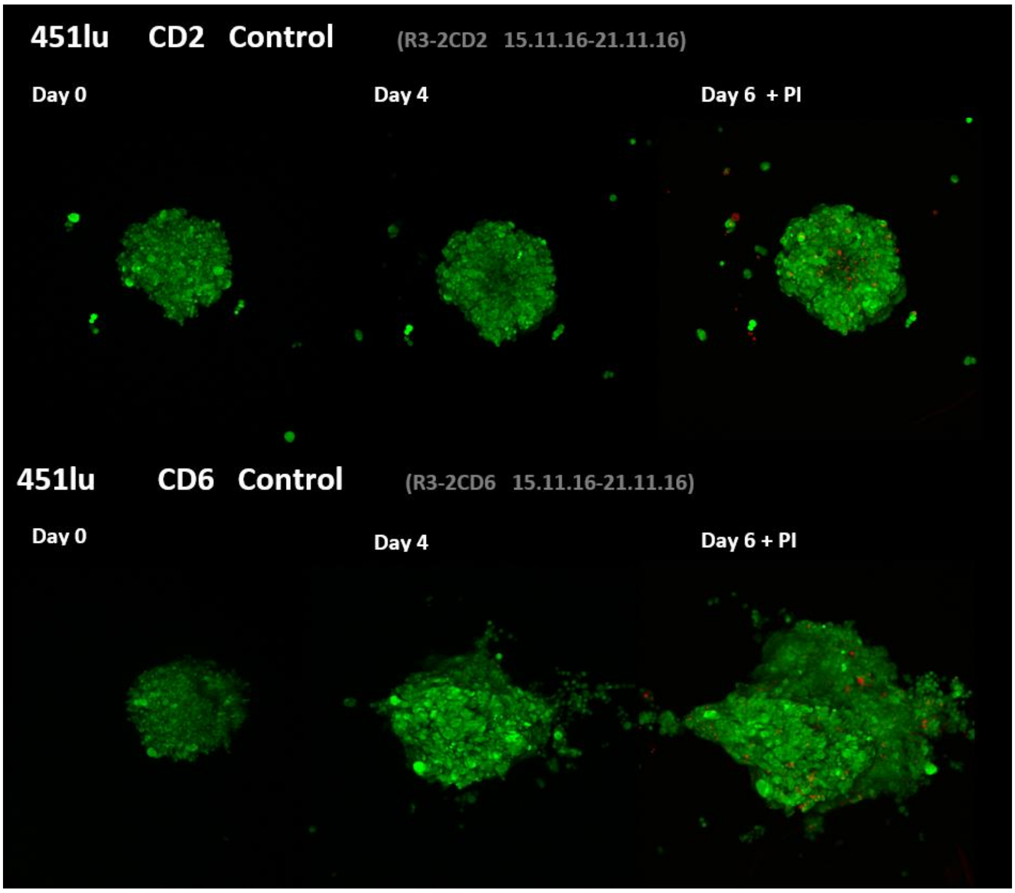
451lu CD2 Control (R3-2CD2 15.11.16-21.11.16)
Day 0
Day 4
Day 6 + PI
451lu CD6 Control (R3-2CD6 15.11.16-21.11.16)
Day 0
Day 4
Day 6 + PI

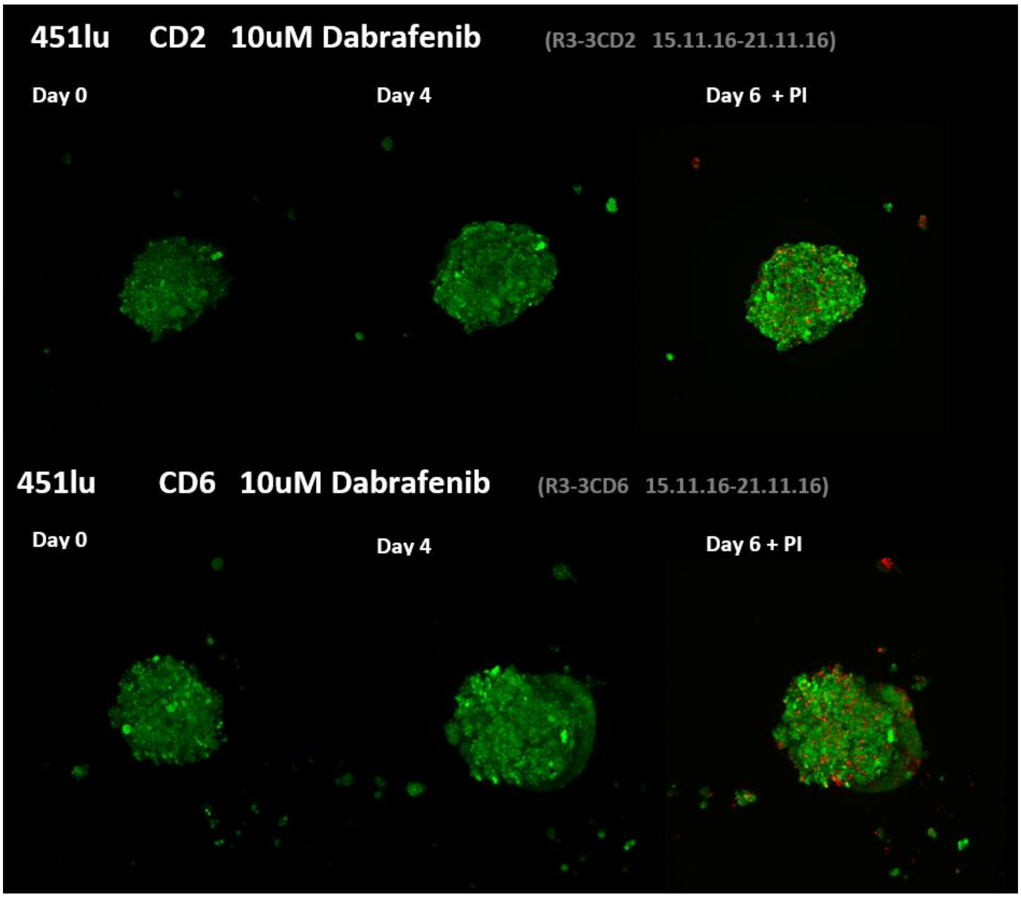
451lu CD2 10uM Dabrafenib (R3-3CD2 15.11.16-21.11.16)
Day 0
Day 4
Day 6 + PI
451lu CD6 10uM Dabrafenib (R3-3CD6 15.11.16-21.11.16)
Day 0
Day 4
Day 6 + PI

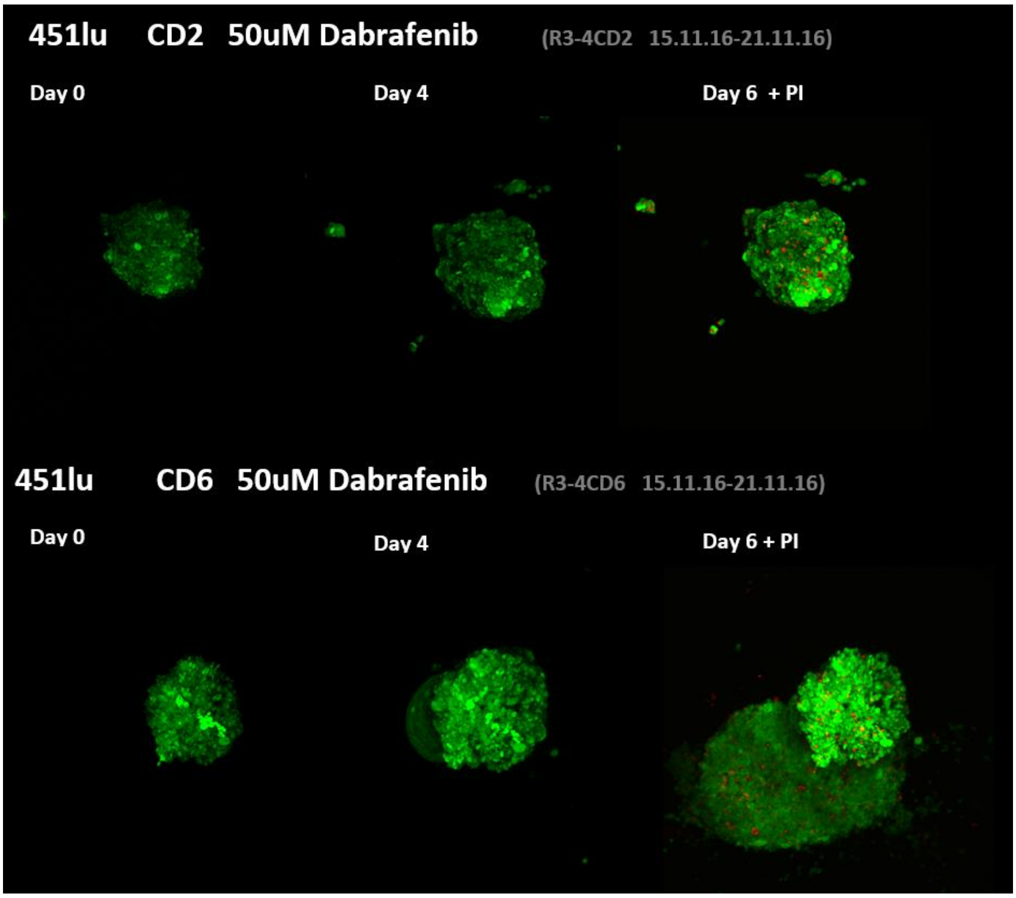
451lu CD2 50uM Dabrafenib
(R3-4CD2 15.11.16-21.11.16)
Day 0
Day 4
Day 6 + PI
451lu CD6 50uM Dabrafenib
(R3-4CD6 15.11.16-21.11.16)
Day 0
Day 4
Day 6 + PI

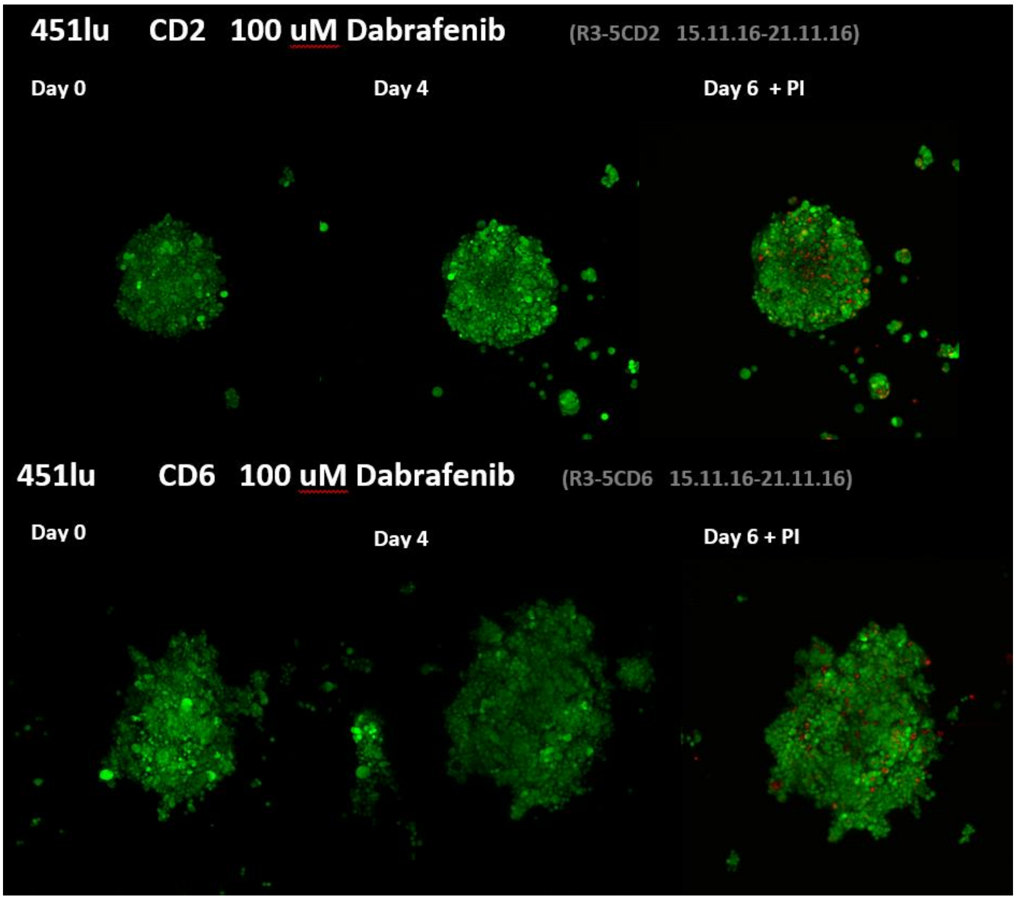
451lu CD2 100 uM Dabrafenib
(R3-5CD2 15.11.16-21.11.16)
Day 0
Day 4
Day 6 + PI
451lu CD6 100 uM Dabrafenib
(R3-5CD6 15.11.16-21.11.16)
Day 0
Day 4
Day 6 + PI

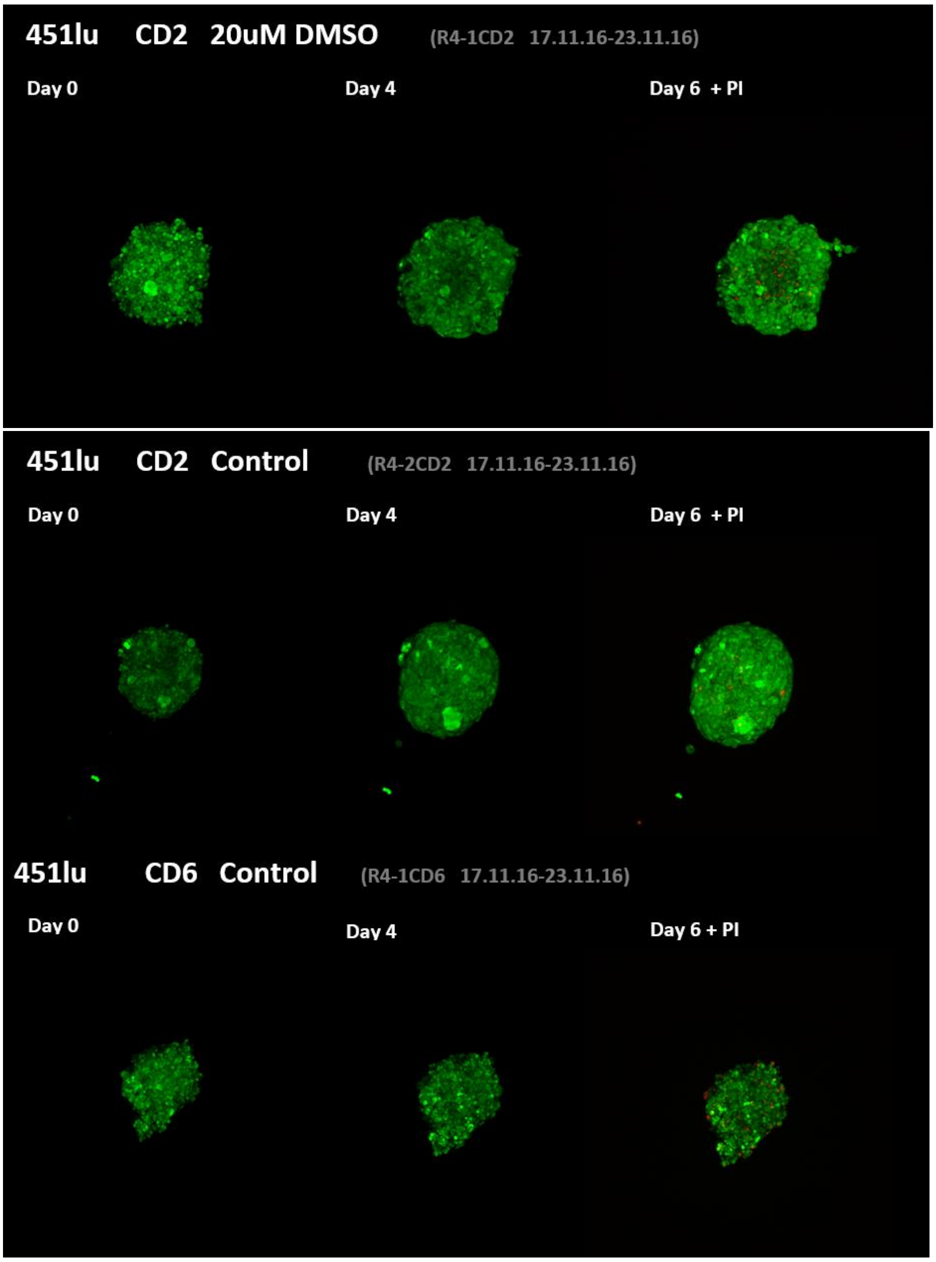
451lu CD2 20uM DMSO (R4-1CD2 17.11.16-23.11.16)
Day 0
Day 4
Day 6 + PI
451lu CD2 Control (R4-2CD2 17.11.16-23.11.16)
Day 0
Day 4
Day 6 + PI
451lu CD6 Control (R4-1CD6 17.11.16-23.11.16)
Day 0
Day 4
Day 6 + PI

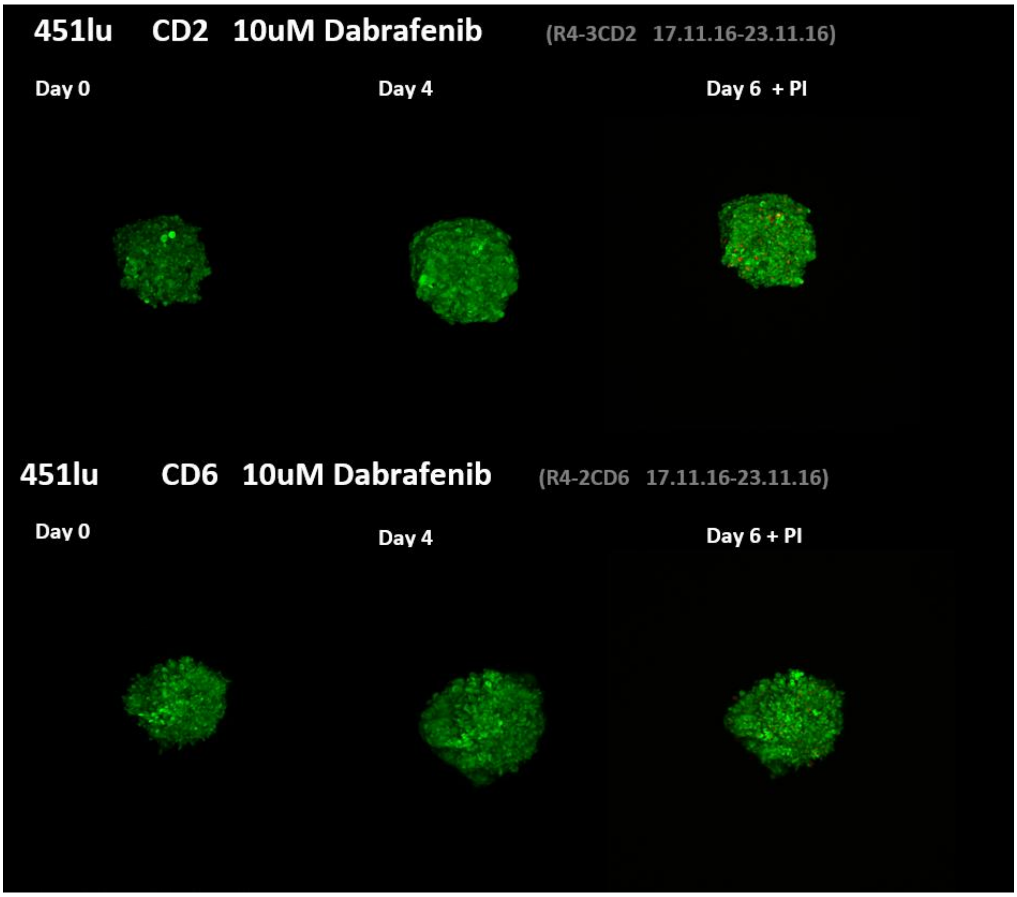
451lu CD2 10uM Dabrafenib
(R4-3CD2 17.11.16-23.11.16)
Day 0
Day 4
Day 6 + PI
451lu CD6 10uM Dabrafenib
(R4-2CD6 17.11.16-23.11.16)
Day 0
Day 4
Day 6 + PI

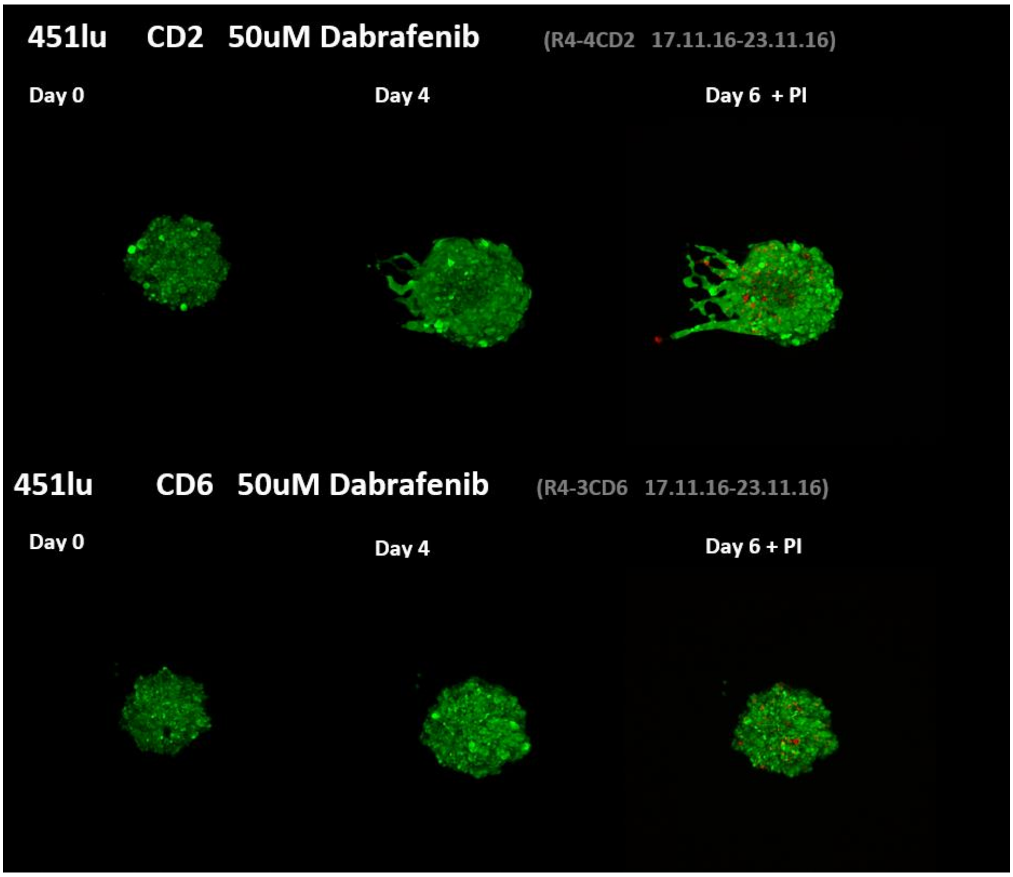
451lu CD2 50uM Dabrafenib
(R4-4CD2 17.11.16-23.11.16)
Day 0
Day 4
Day 6 + PI
451lu CD6 50uM Dabrafenib
(R4-3CD6 17.11.16-23.11.16)
Day 0
Day 4
Day 6 + PI

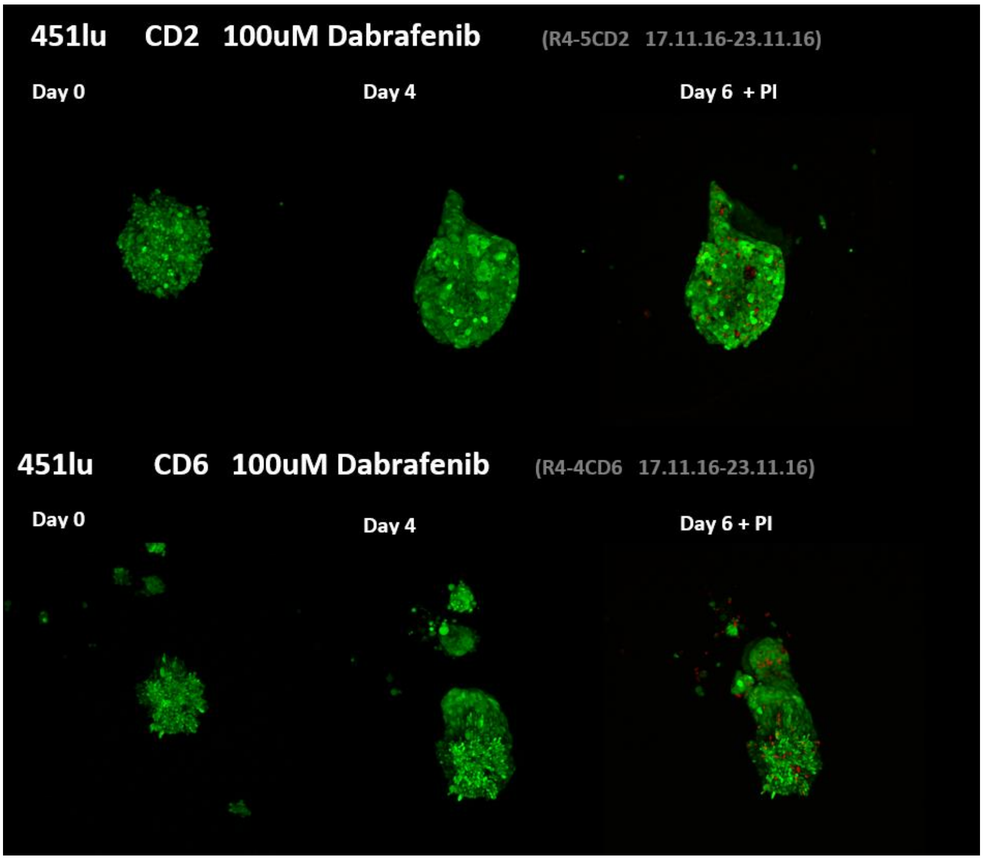
451lu CD2 100uM Dabrafenib (R4-5CD2 17.11.16-23.11.16)
Day 0
Day 4
Day 6 + PI
451lu CD6 100uM Dabrafenib (R4-4CD6 17.11.16-23.11.16)
Day 0
Day 4
Day 6 + PI
