# Supplementary material for: Mechanistically Coupled PK (MCPK) Model to Describe Enzyme Induction and Occupancy Dependent DDI of Dabrafenib Metabolism
Source: Pharmaceutics. 2022 Jan 28;14(2):310. doi: 10.3390/pharmaceutics14020310 (PMC8875124; doi:10.3390/pharmaceutics14020310)
Supplement: Supplementary file 1 [file pharmaceutics-14-00310-s001.zip › pharmaceutics-1479084-supplementary/Supplementary_Information/Supplemental_Information_S3.pdf]

Supplemental Information S3: Mechanistically Coupled PK (MCPK) model to describe enzyme induction and occupancy dependent DDI of dabrafenib metabolism

Marco Albrecht[1,2], Yuri Kogan[3], Dagmar Kulms[4], and Thomas Sauter[5]

[1] *Université du Luxembourg; Systems Biology Group; 4367 Belvaux; Luxembourg; Email: marco.albrecht@posteo.eu*
[2] *esqLABS GmbH; 26683 Saterland; Germany; Email: marco.albrecht@esqlabs.com*
[3] *Institute for Medical Biomathematics (IMBM); 60991 Bnei Atarot; Israel; Email: yuri@imbm.com*
[4] *Technical University of Dresden; Experimental Dermatology; 01307 Dresden; Germany; Tel.: +49 (0)351 458-18973 ; Email: dagmar.kulms@uniklinikum-dresden.de*
[5] *Université du Luxembourg; Systems Biology Group; 4367 Belvaux; Luxembourg; Tel.: (+352) 46 66 44 6296; Email: thomas.sauter@uni.lu*

Spheroid Image Data of 451LU Spheroid Growth

Spheroid Image Data of 451LU Spheroid Growth 1
Legend 1
451LU - First Iteration 2
451LU - Second Iteration 9

Legend

CD: Gel with cell degradable linker
PEG: Gel with non-degradable polyethylene glycol linker
R1-3: Biological Replicate 1 to 3.
451LU: Metastatic Melanoma Cell Line with **green** flourecent protein
PI: Propidium iodide as a **red** cell death marker at last day

451LU - First Iteration

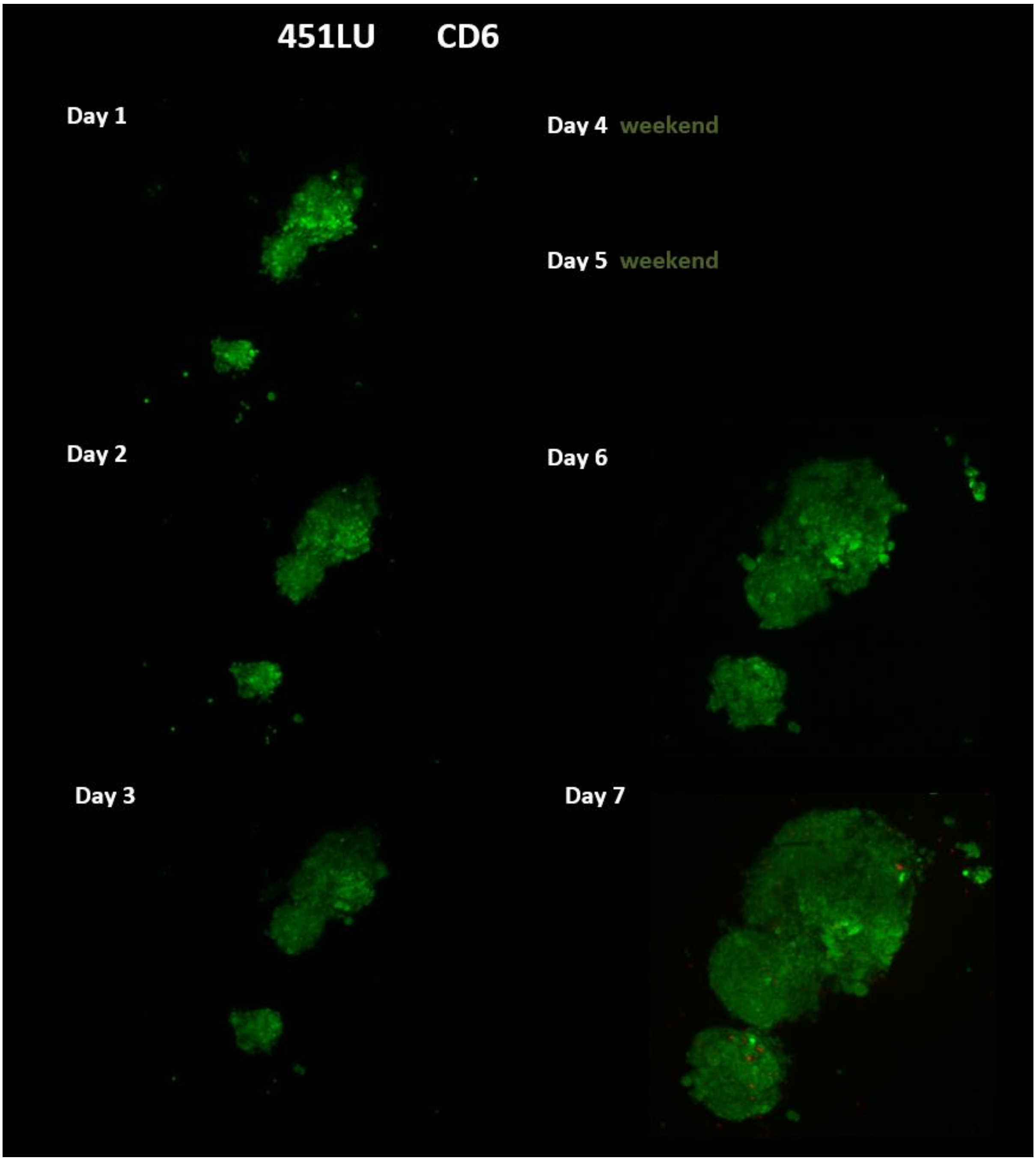

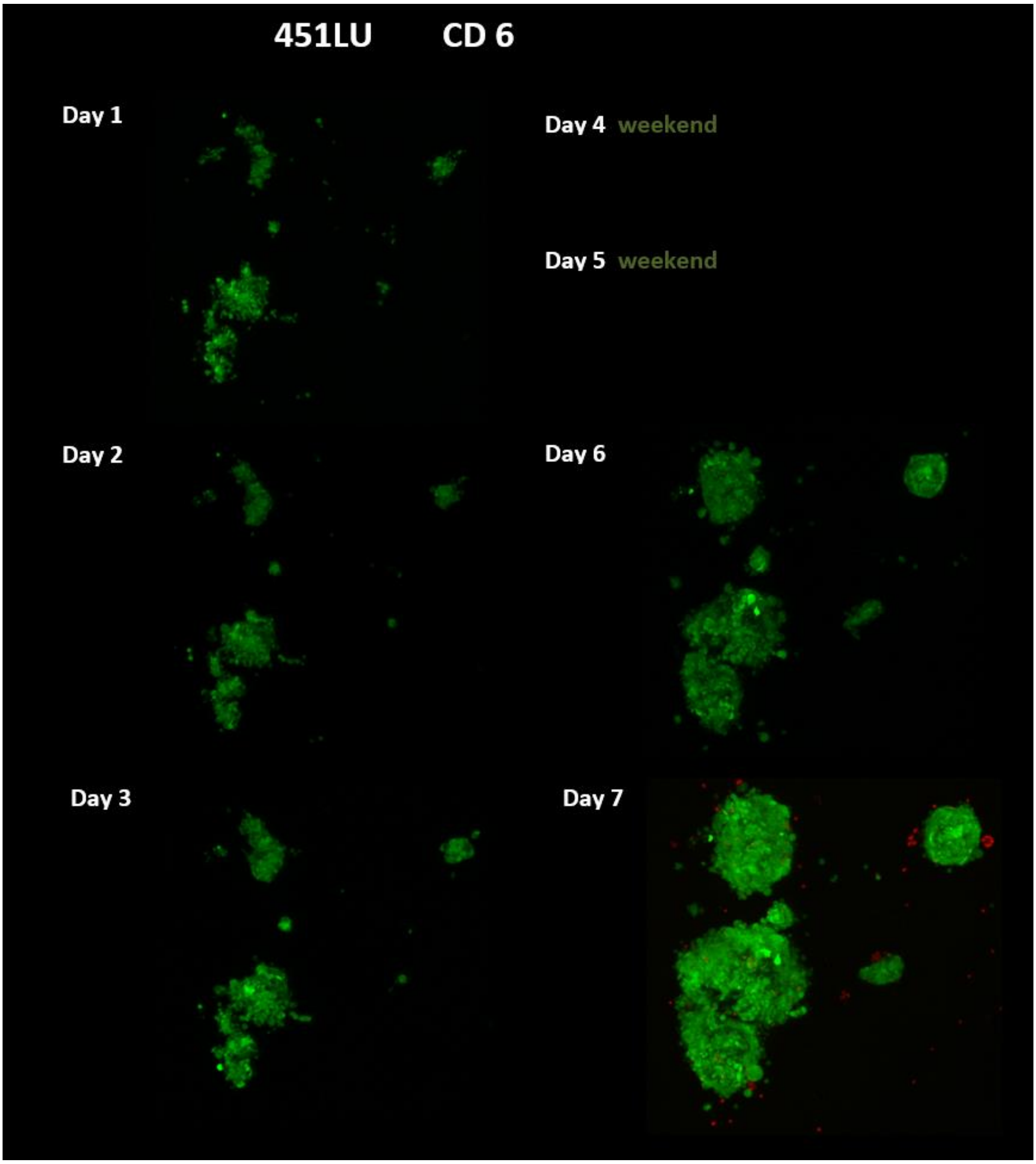
451LU
CD 6
Day 1
Day 4 weekend
Day 5 weekend
Day 2
Day 6
Day 3
Day 7

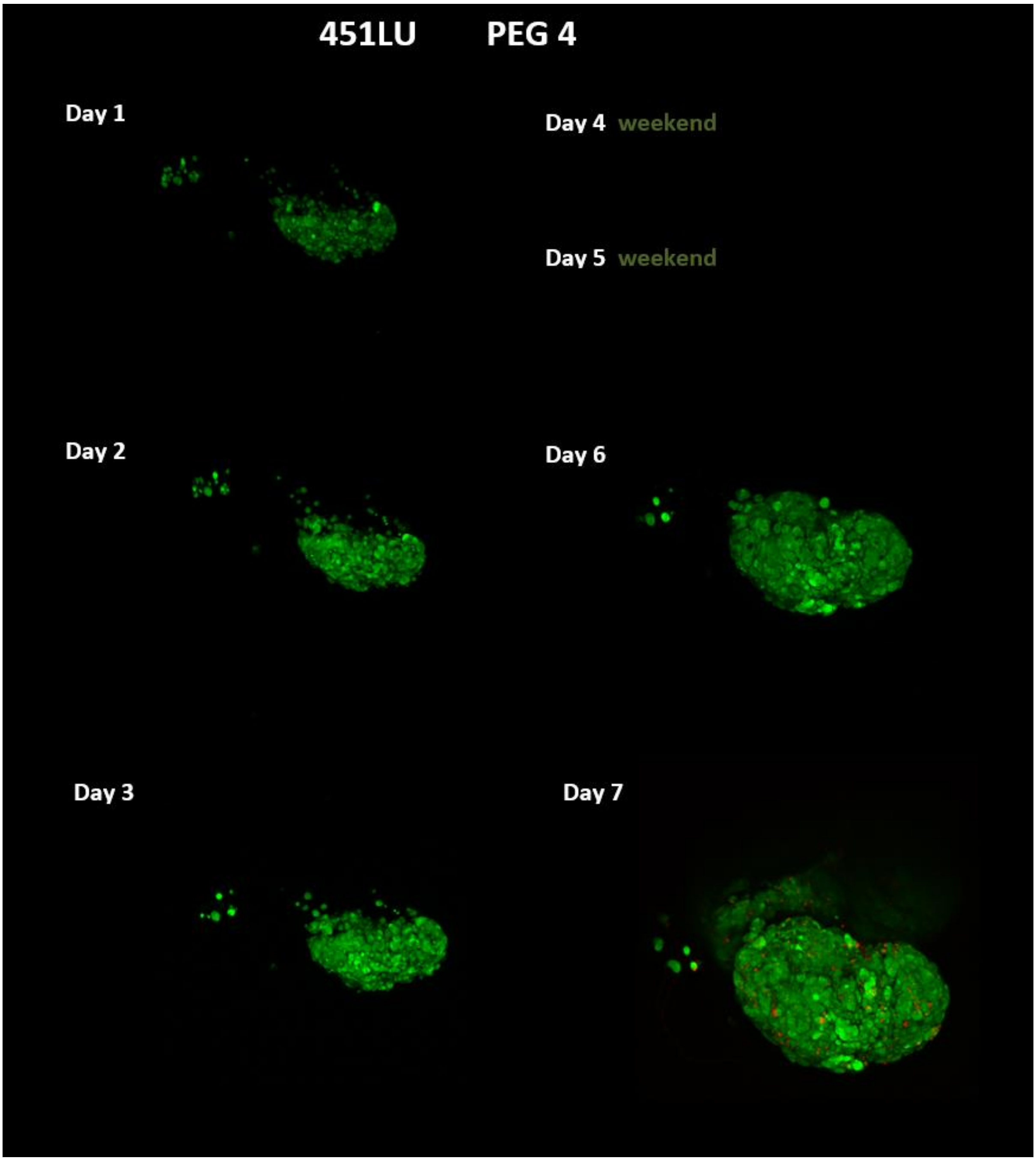
451LU PEG 4
Day 1
Day 4 weekend
Day 5 weekend
Day 2
Day 6
Day 3
Day 7

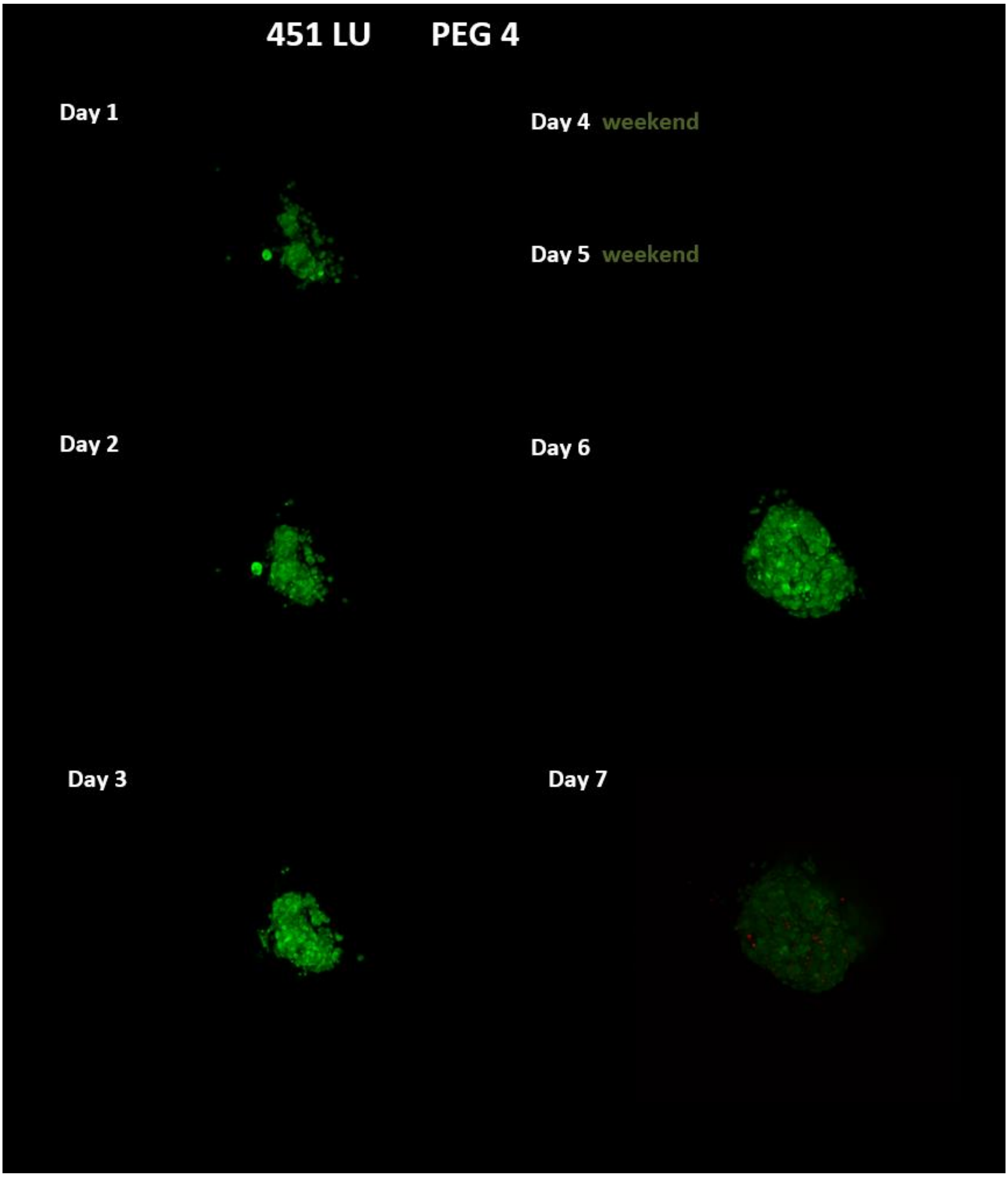
451 LU
PEG 4
Day 1
Day 4 weekend
Day 5 weekend
Day 2
Day 6
Day 3
Day 7

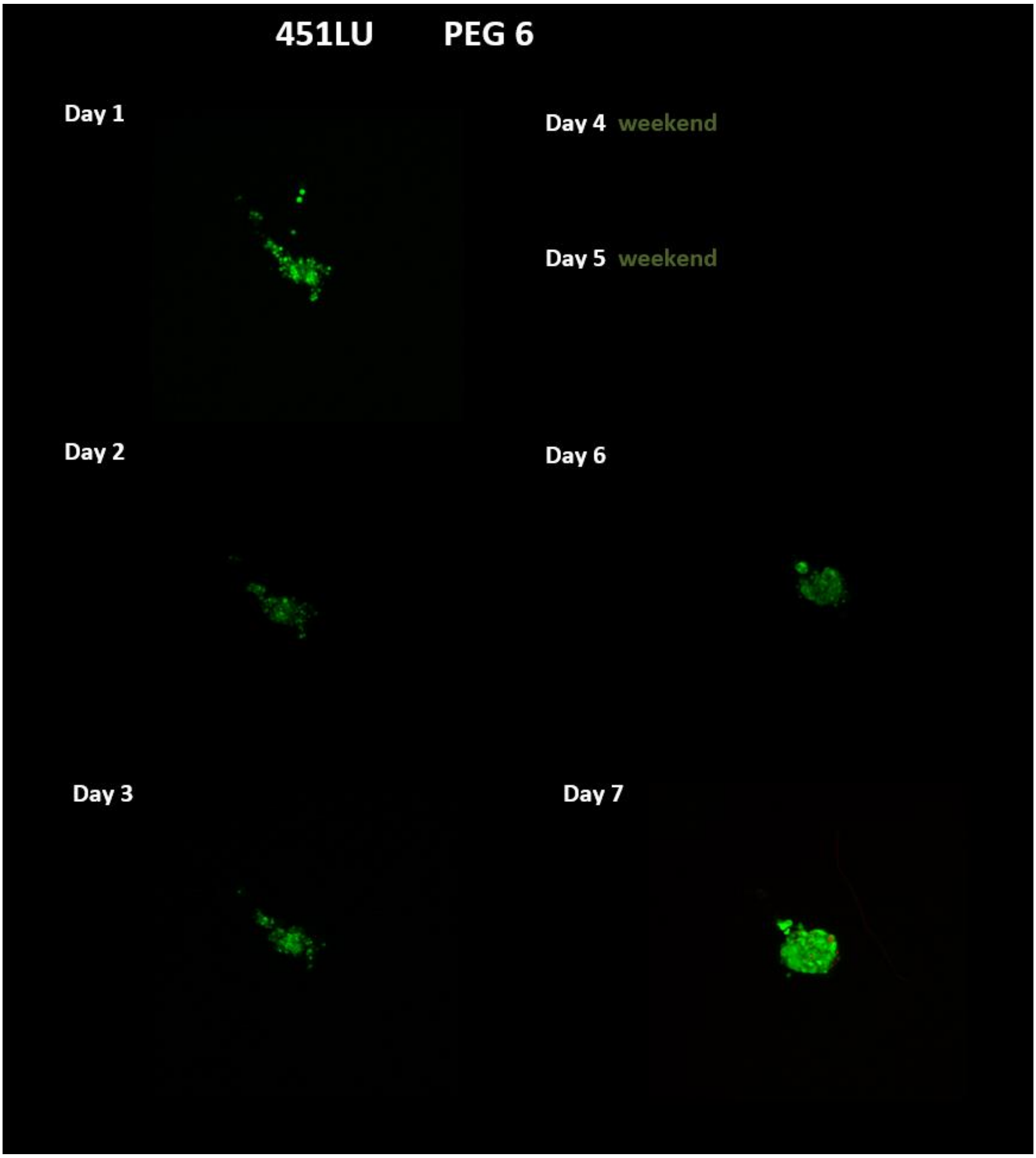
451LU PEG 6
Day 1
Day 4 weekend
Day 5 weekend
Day 2
Day 6
Day 3
Day 7

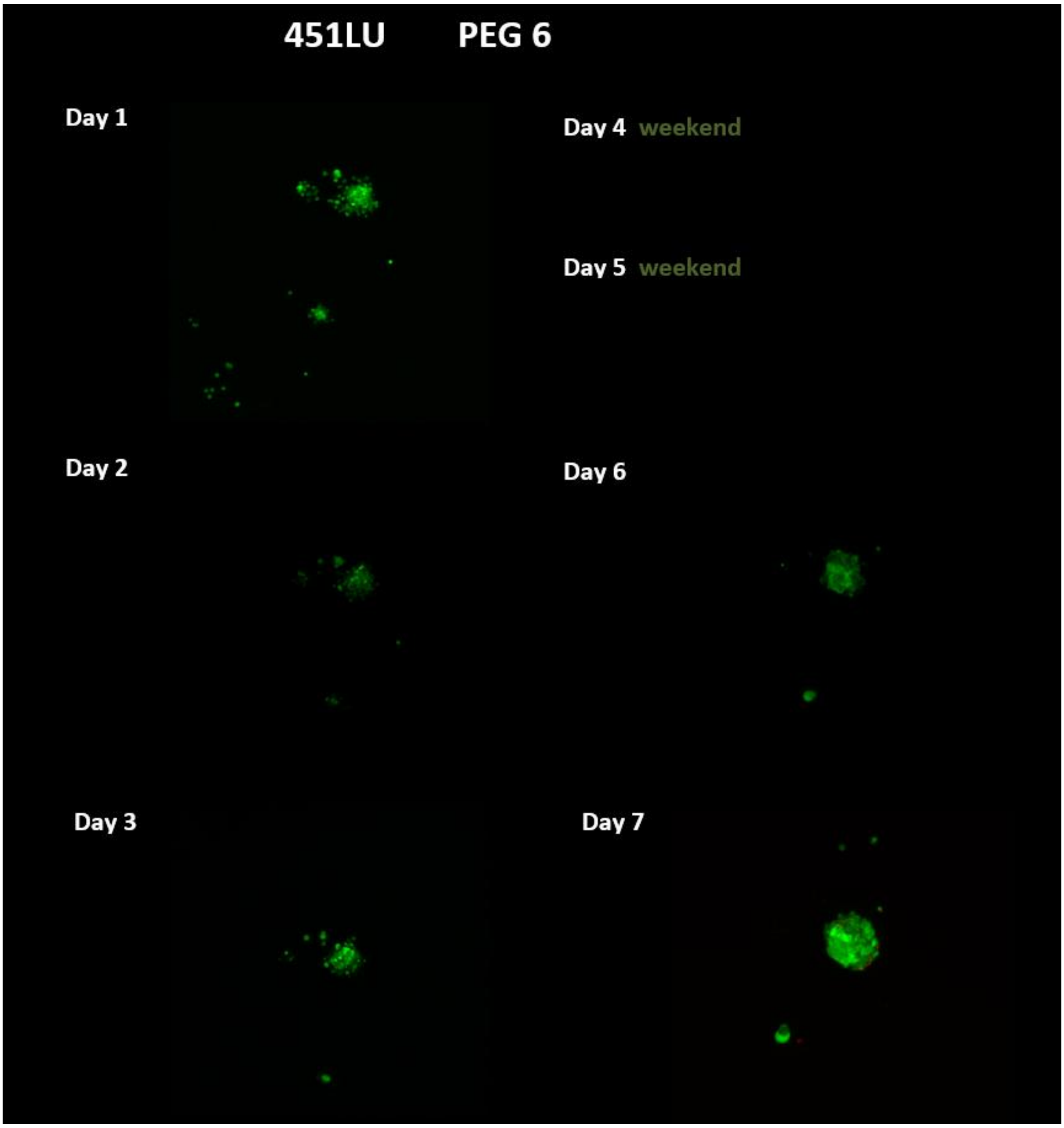
451LU PEG 6
Day 1
Day 4 weekend
Day 5 weekend
Day 2
Day 6
Day 3
Day 7

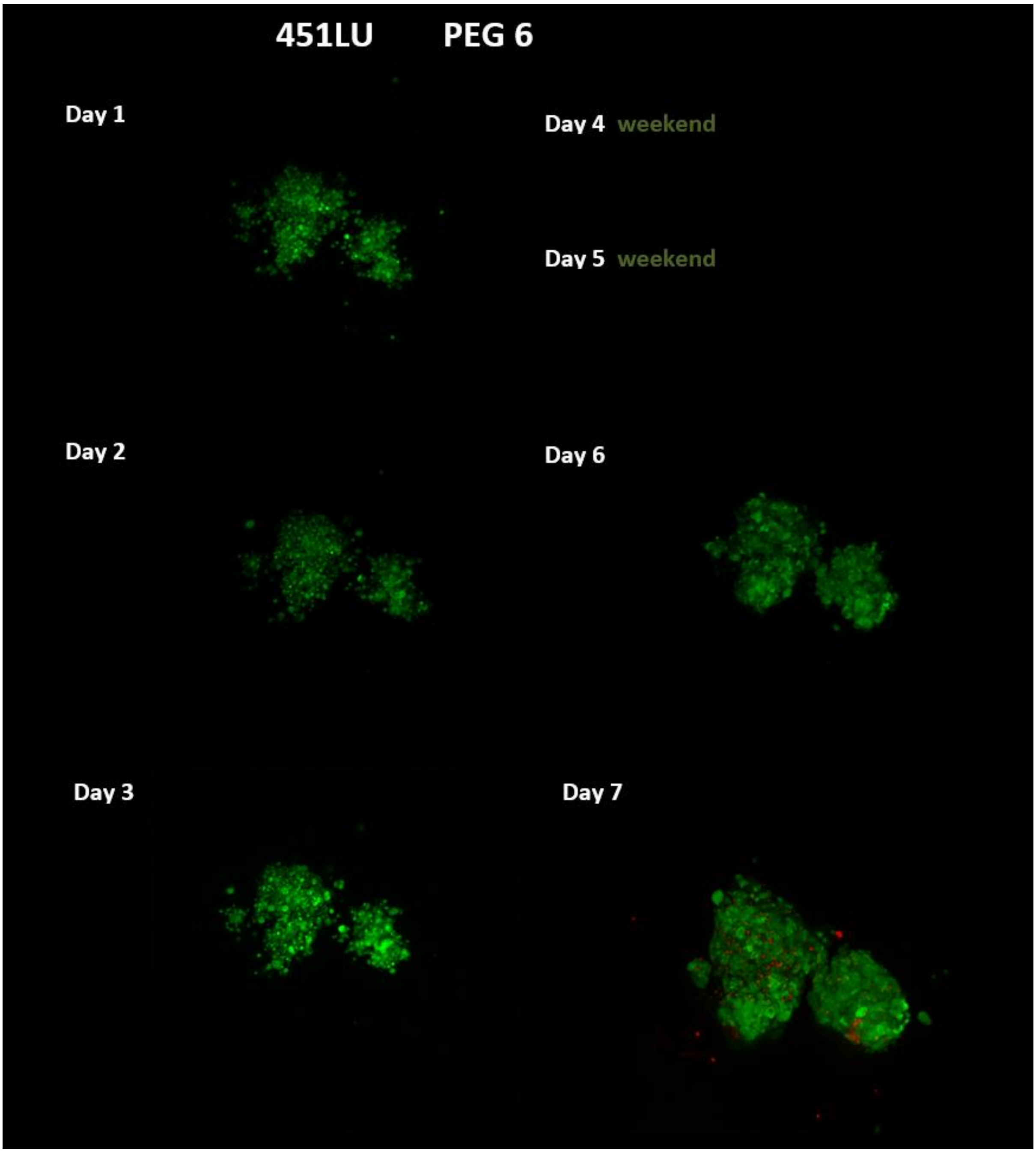
451LU PEG 6
Day 1
Day 4 weekend
Day 5 weekend
Day 2
Day 6
Day 3
Day 7

451LU - Second Iteration

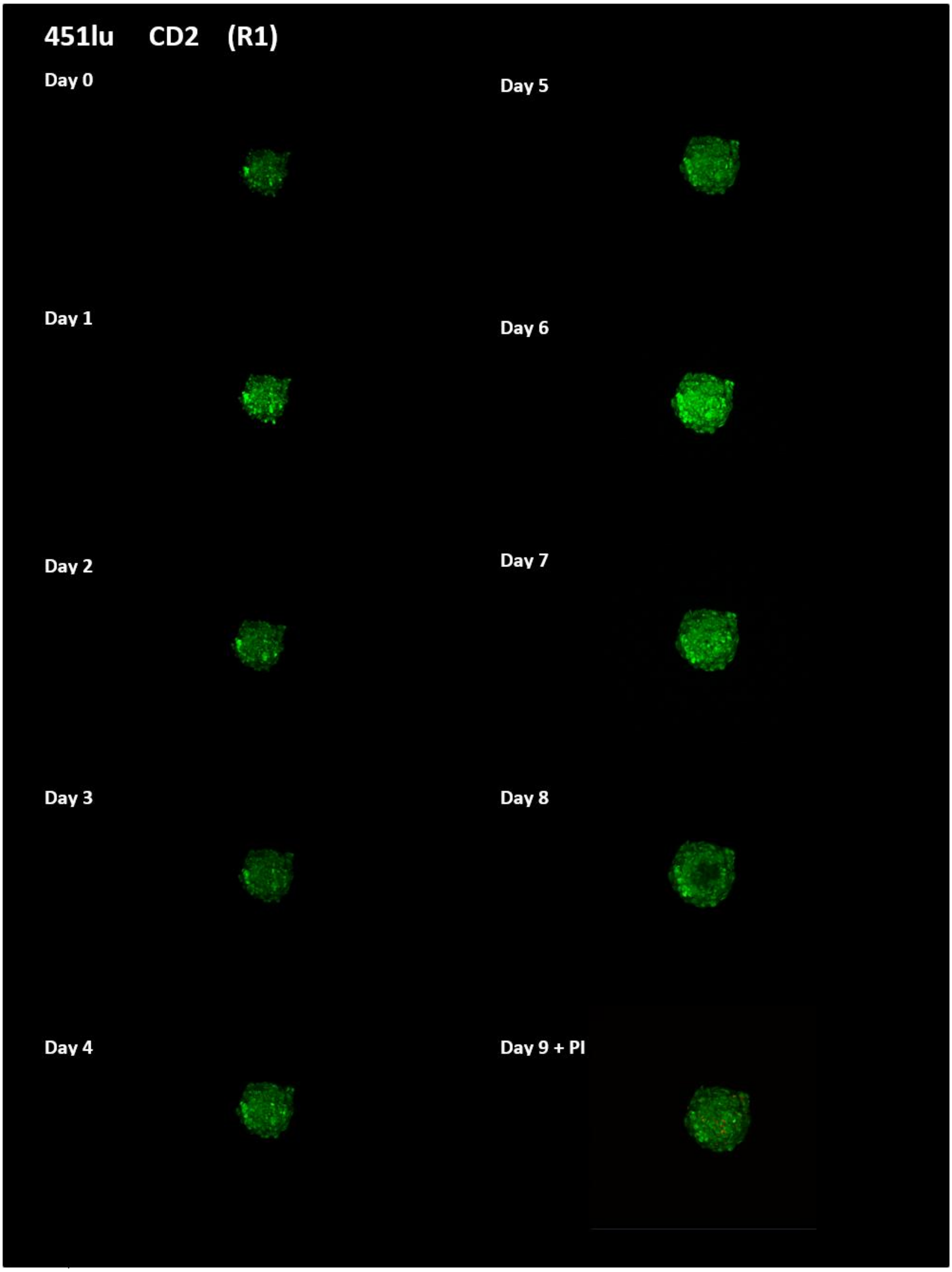

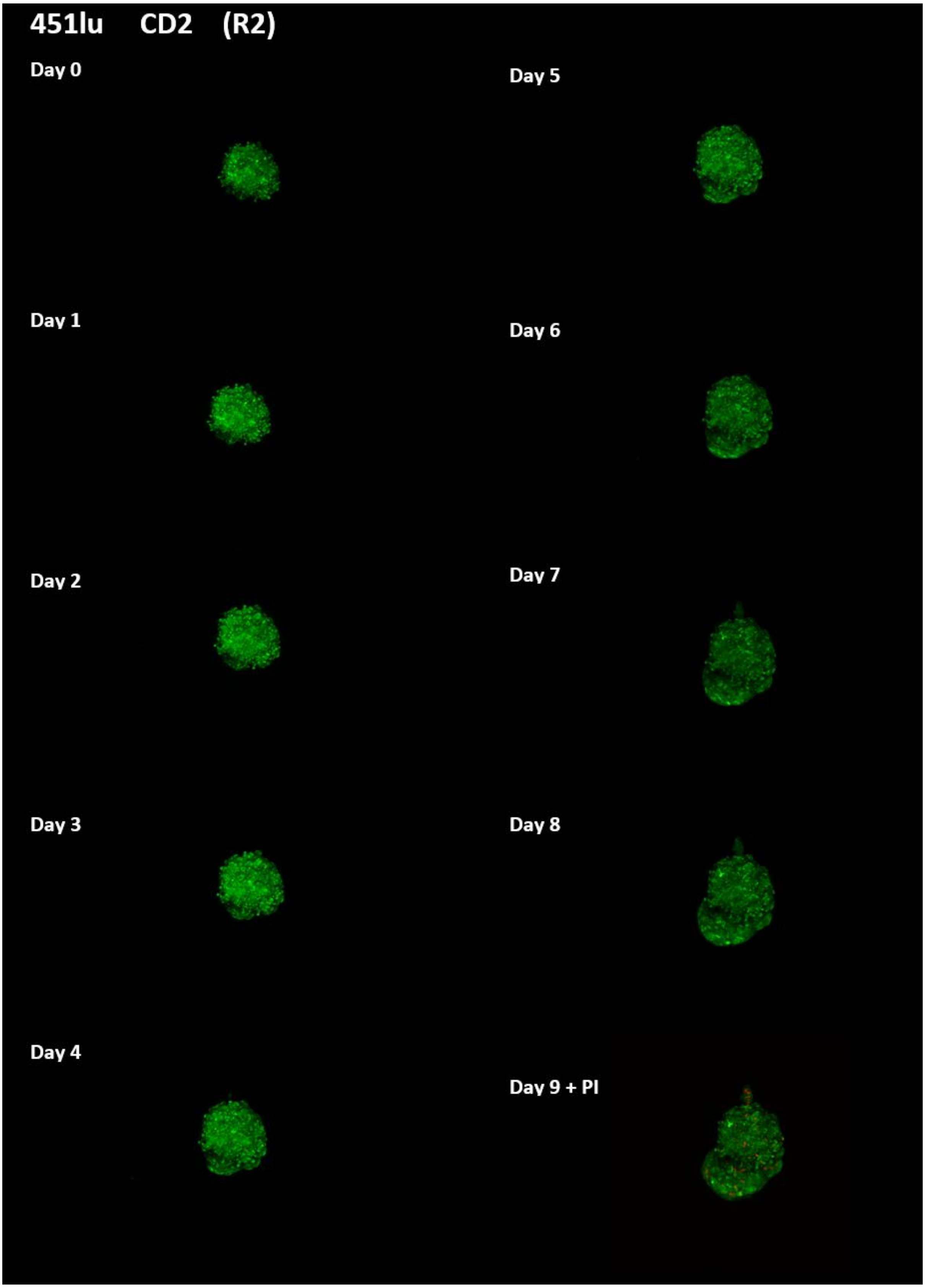
451lu CD2 (R2)
Day 0
Day 5
Day 1
Day 6
Day 2
Day 7
Day 3
Day 8
Day 4
Day 9 + PI

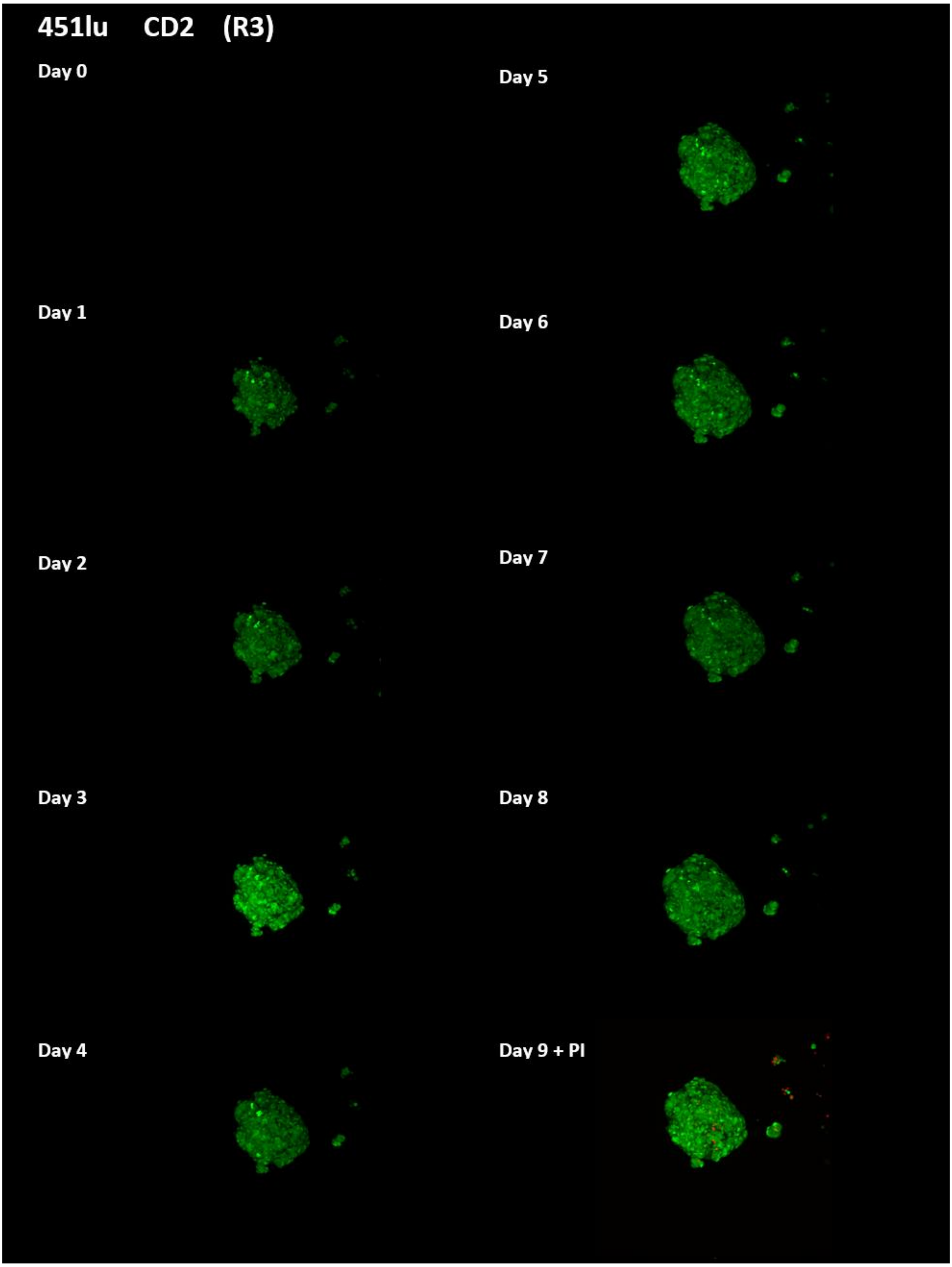
451lu CD2 (R3)
Day 0
Day 5
Day 1
Day 6
Day 2
Day 7
Day 3
Day 8
Day 4
Day 9 + PI

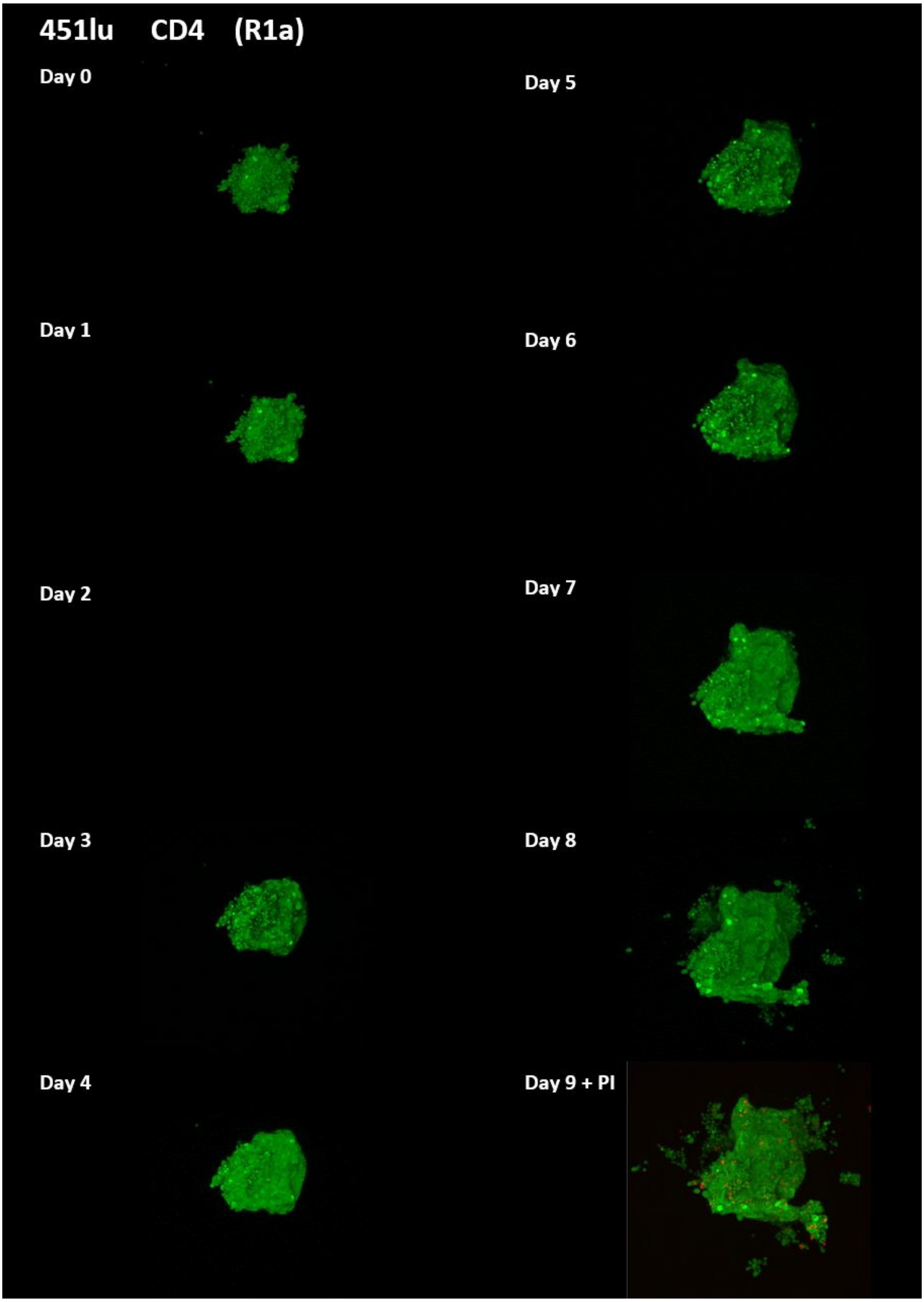
451lu CD4 (R1a)
Day 0
Day 5
Day 1
Day 6
Day 2
Day 7
Day 3
Day 8
Day 4
Day 9 + PI

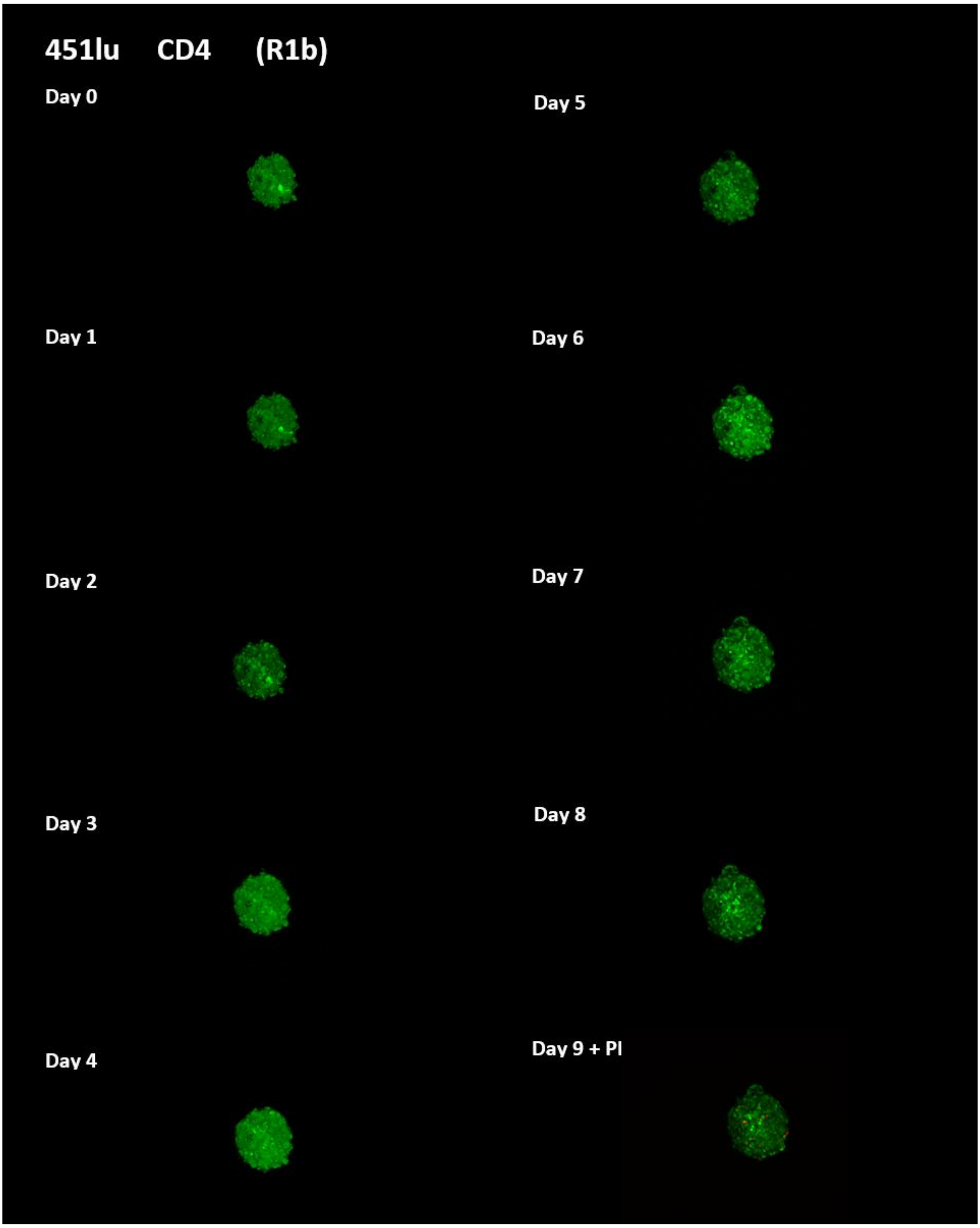
451lu CD4 (R1b)
Day 0
Day 1
Day 2
Day 3
Day 4
Day 5
Day 6
Day 7
Day 8
Day 9 + PI

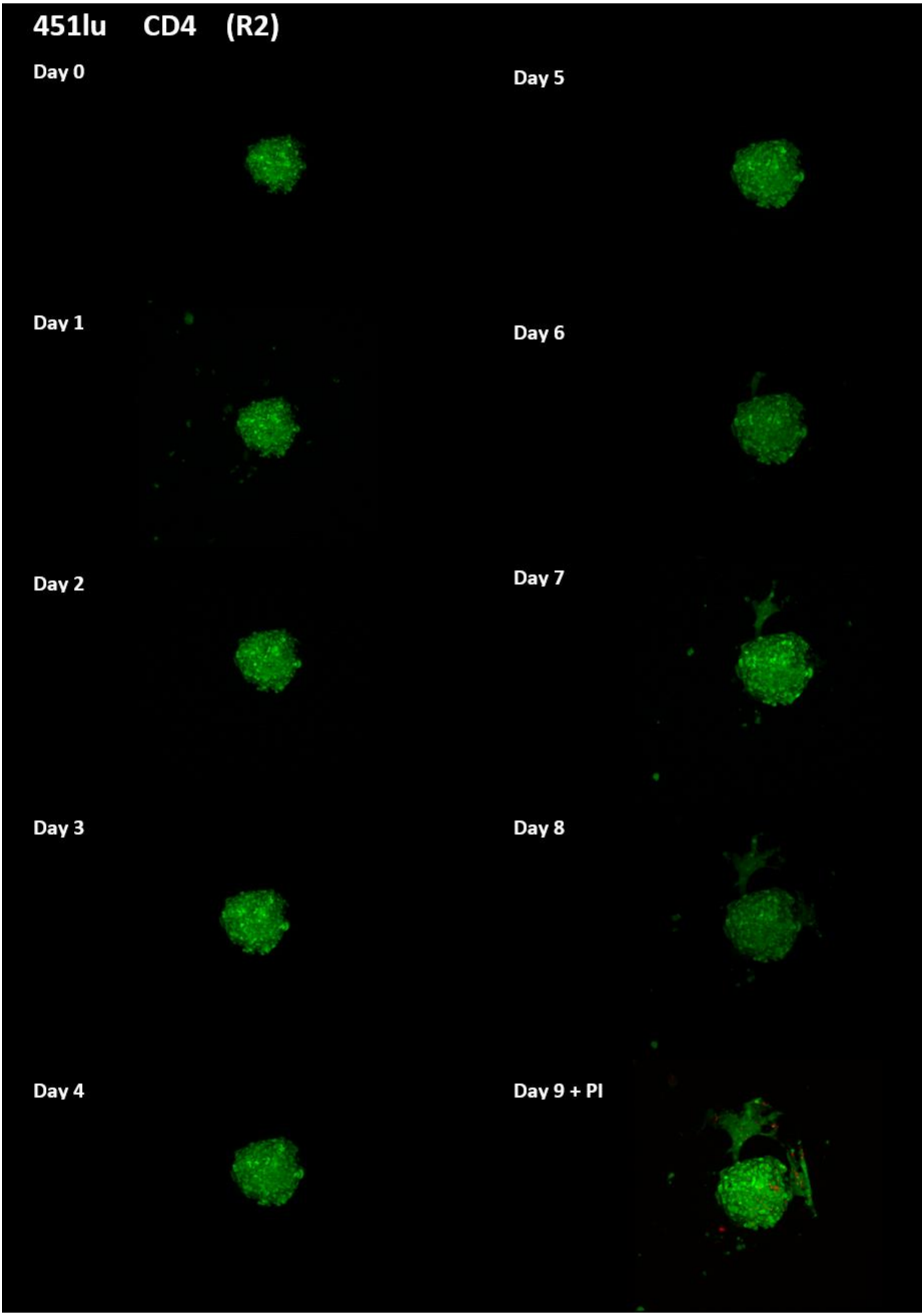
451lu CD4 (R2)
Day 0
Day 5
Day 1
Day 6
Day 2
Day 7
Day 3
Day 8
Day 4
Day 9 + PI

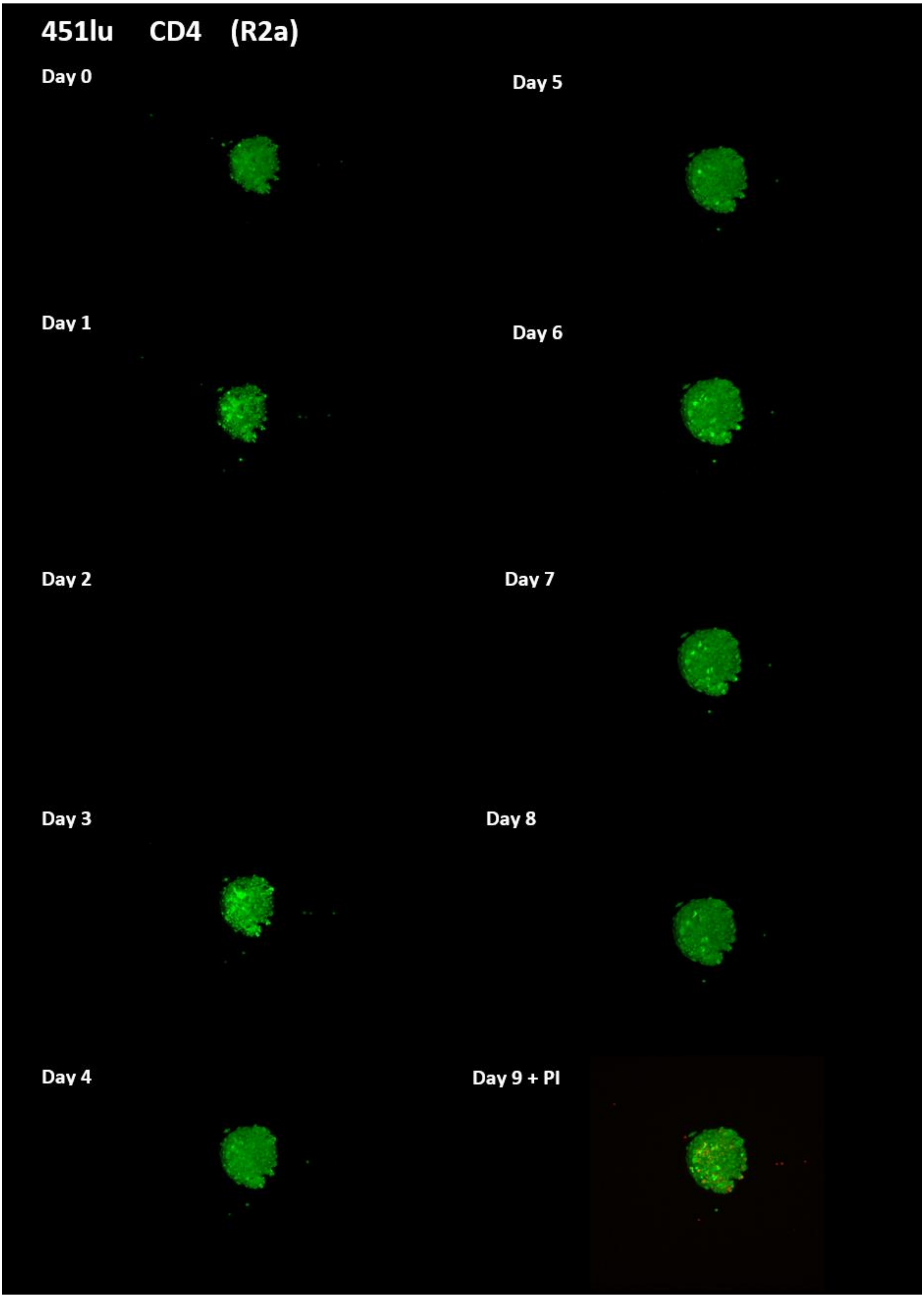
451lu CD4 (R2a)
Day 0
Day 5
Day 1
Day 6
Day 2
Day 7
Day 3
Day 8
Day 4
Day 9 + PI

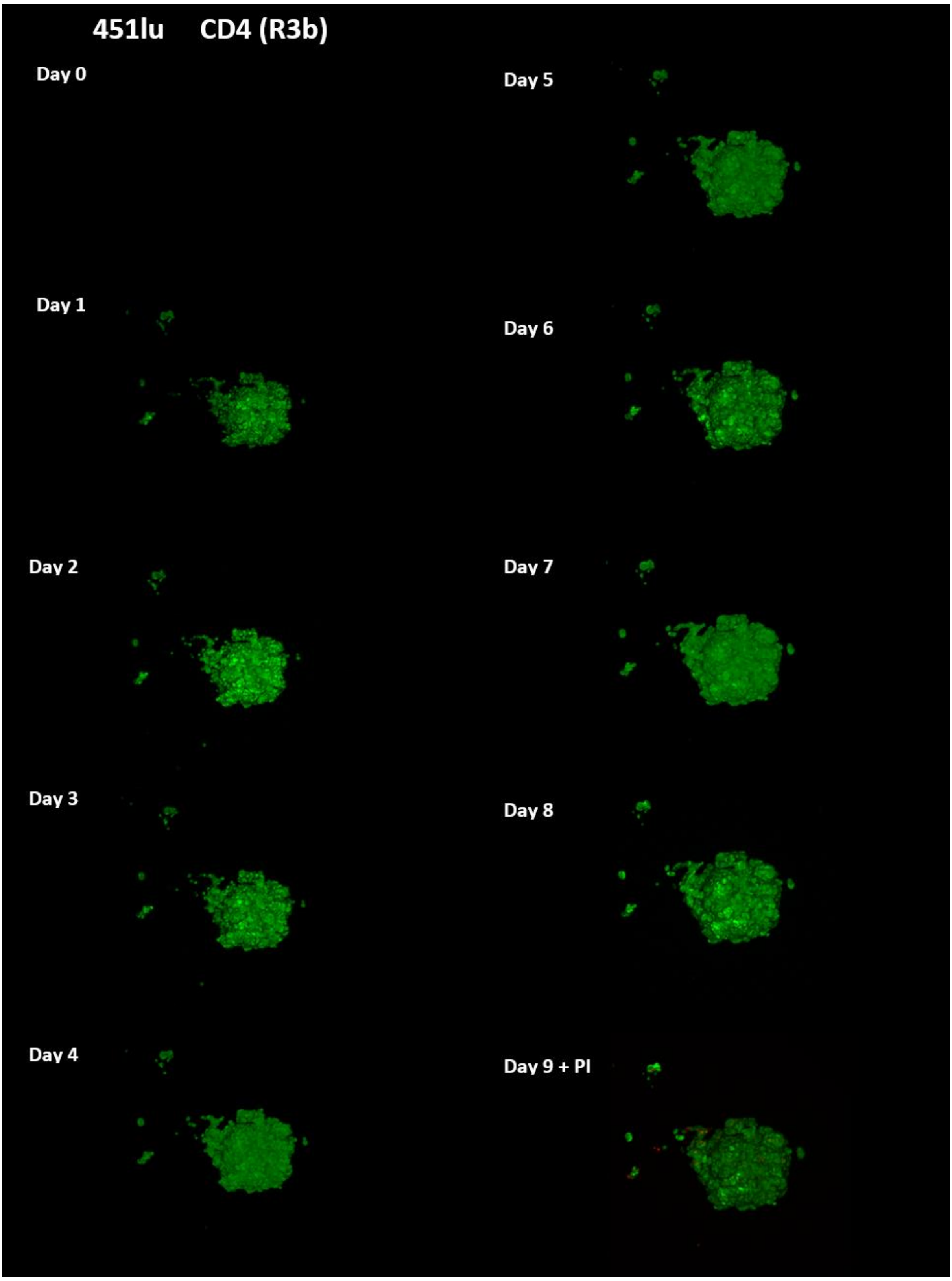
451lu CD4 (R3b)
Day 0
Day 1
Day 2
Day 3
Day 4
Day 5
Day 6
Day 7
Day 8
Day 9 + PI

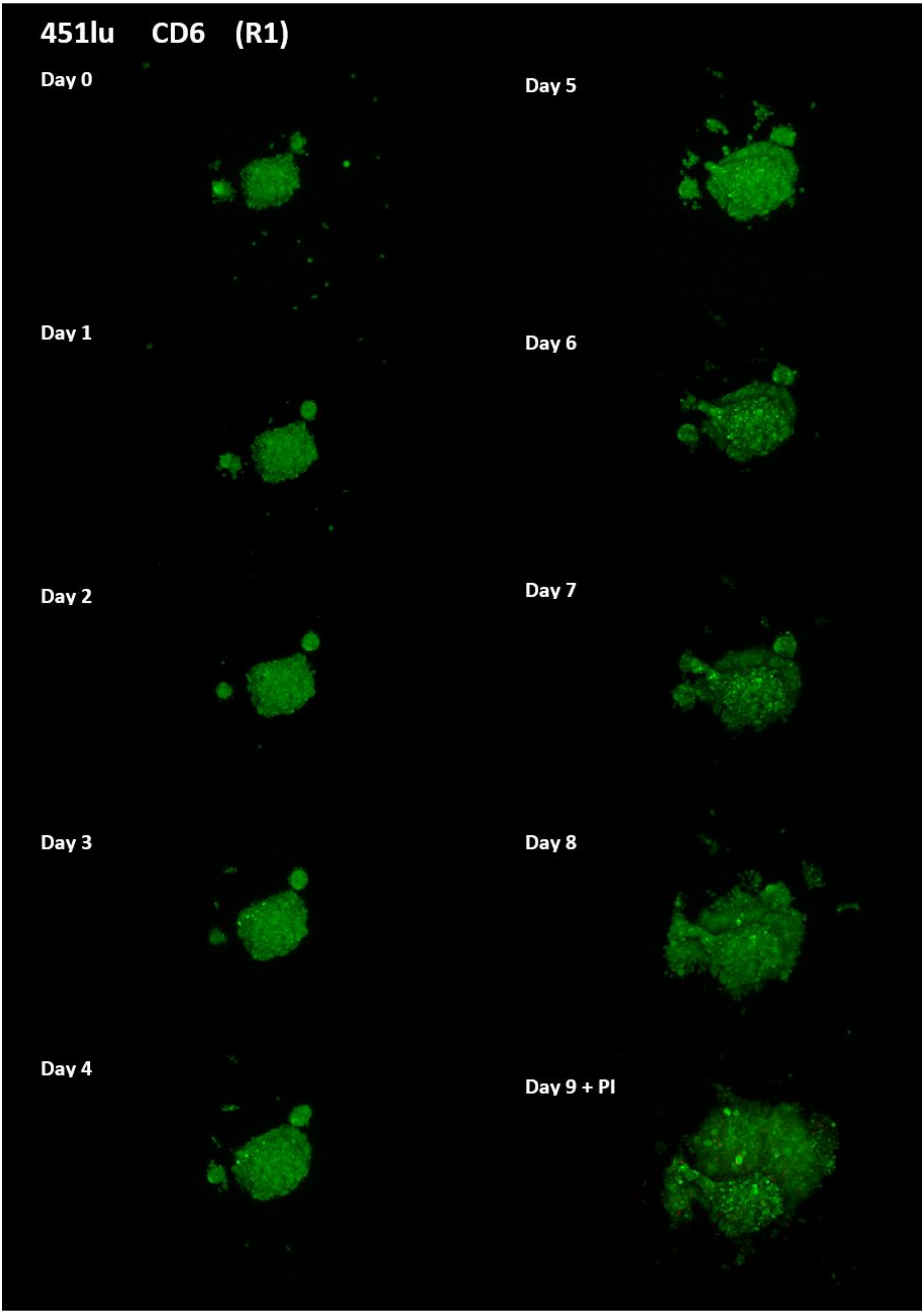
451lu CD6 (R1)
Day 0
Day 1
Day 2
Day 3
Day 4
Day 5
Day 6
Day 7
Day 8
Day 9 + PI

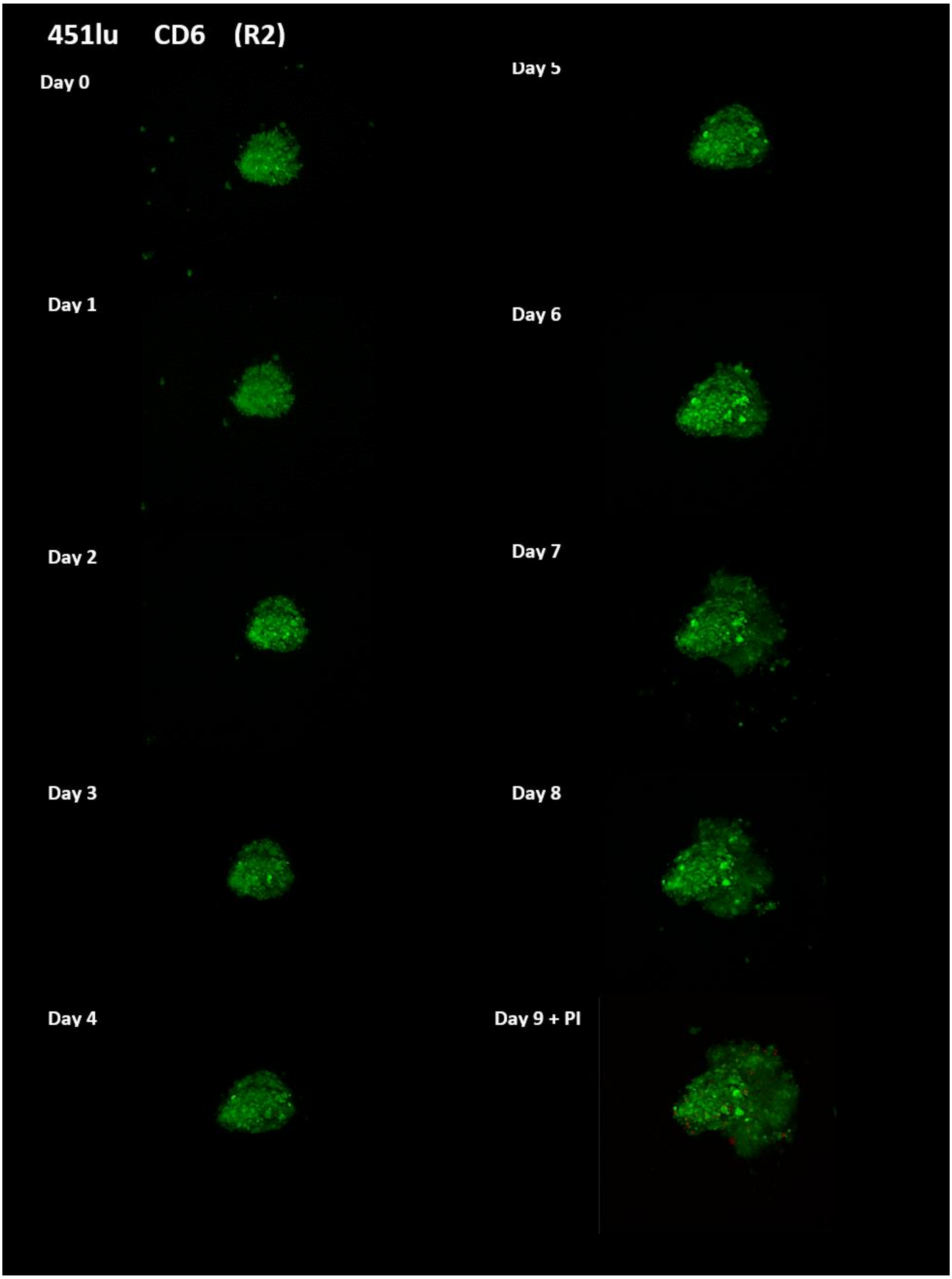
451lu CD6 (R2)
Day 0
Day 1
Day 2
Day 3
Day 4
Day 5
Day 6
Day 7
Day 8
Day 9 + PI

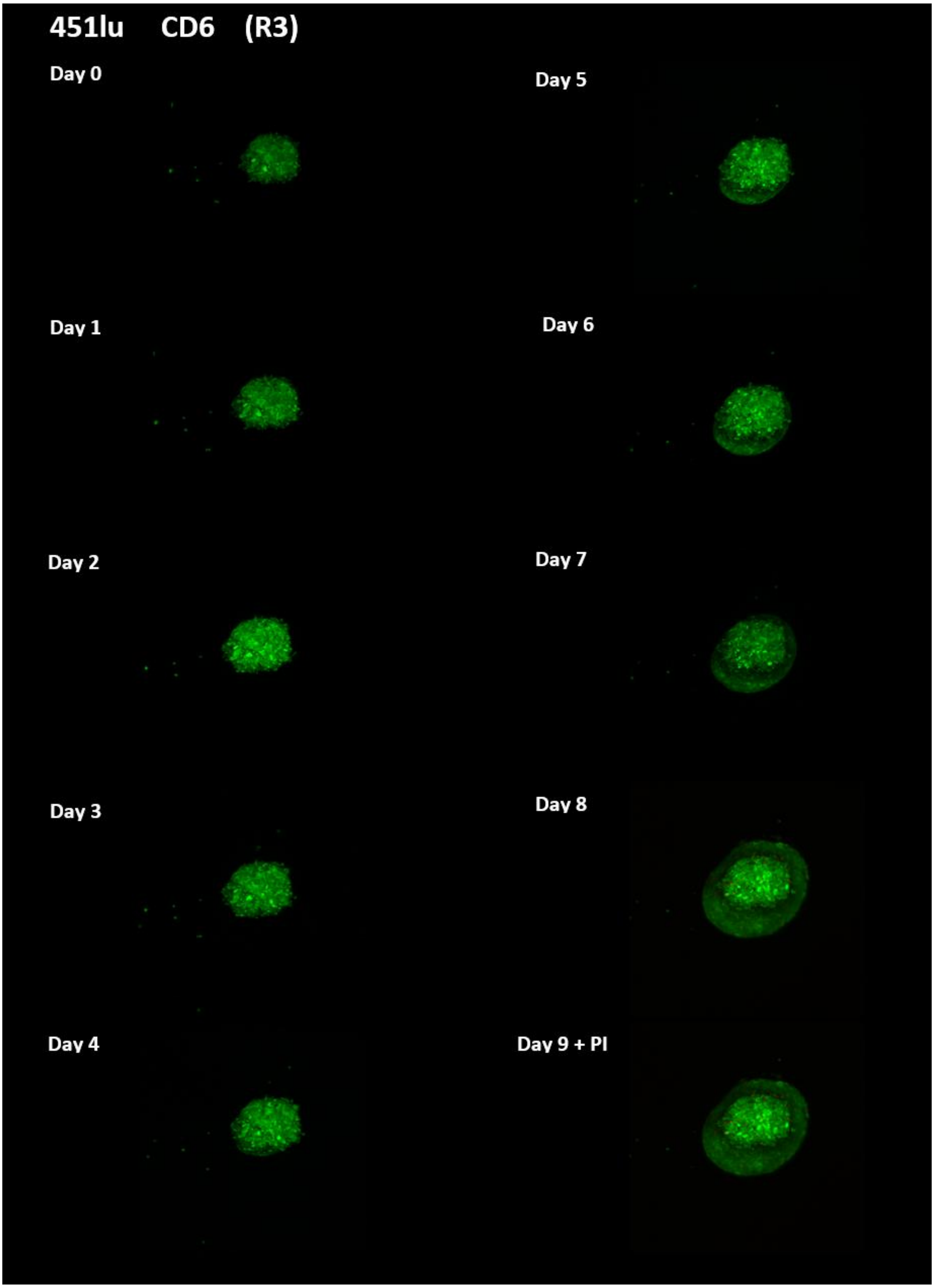
451lu CD6 (R3)
Day 0
Day 5
Day 1
Day 6
Day 2
Day 7
Day 3
Day 8
Day 4
Day 9 + PI

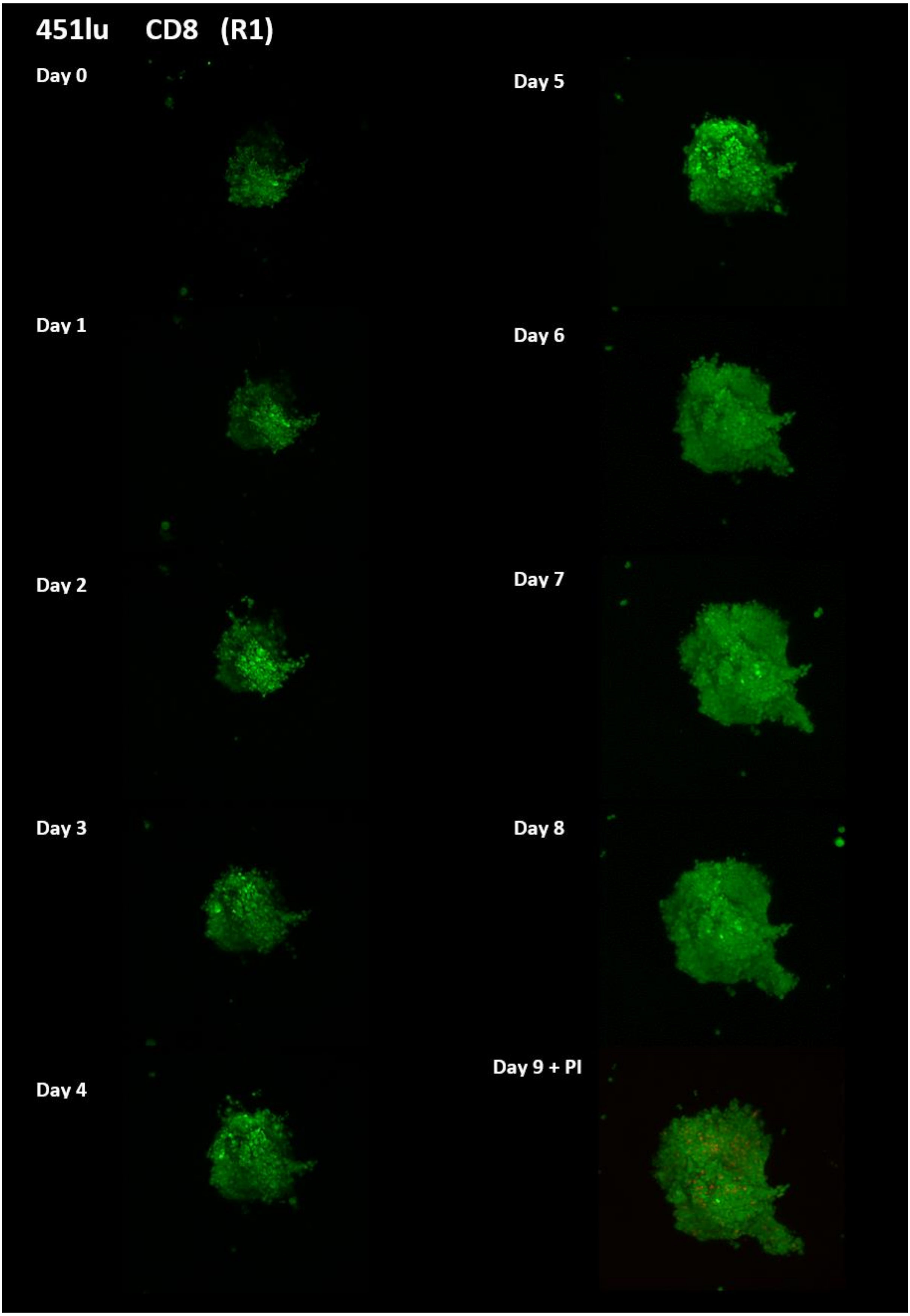
451lu CD8 (R1)
Day 0
Day 1
Day 2
Day 3
Day 4
Day 5
Day 6
Day 7
Day 8
Day 9 + PI

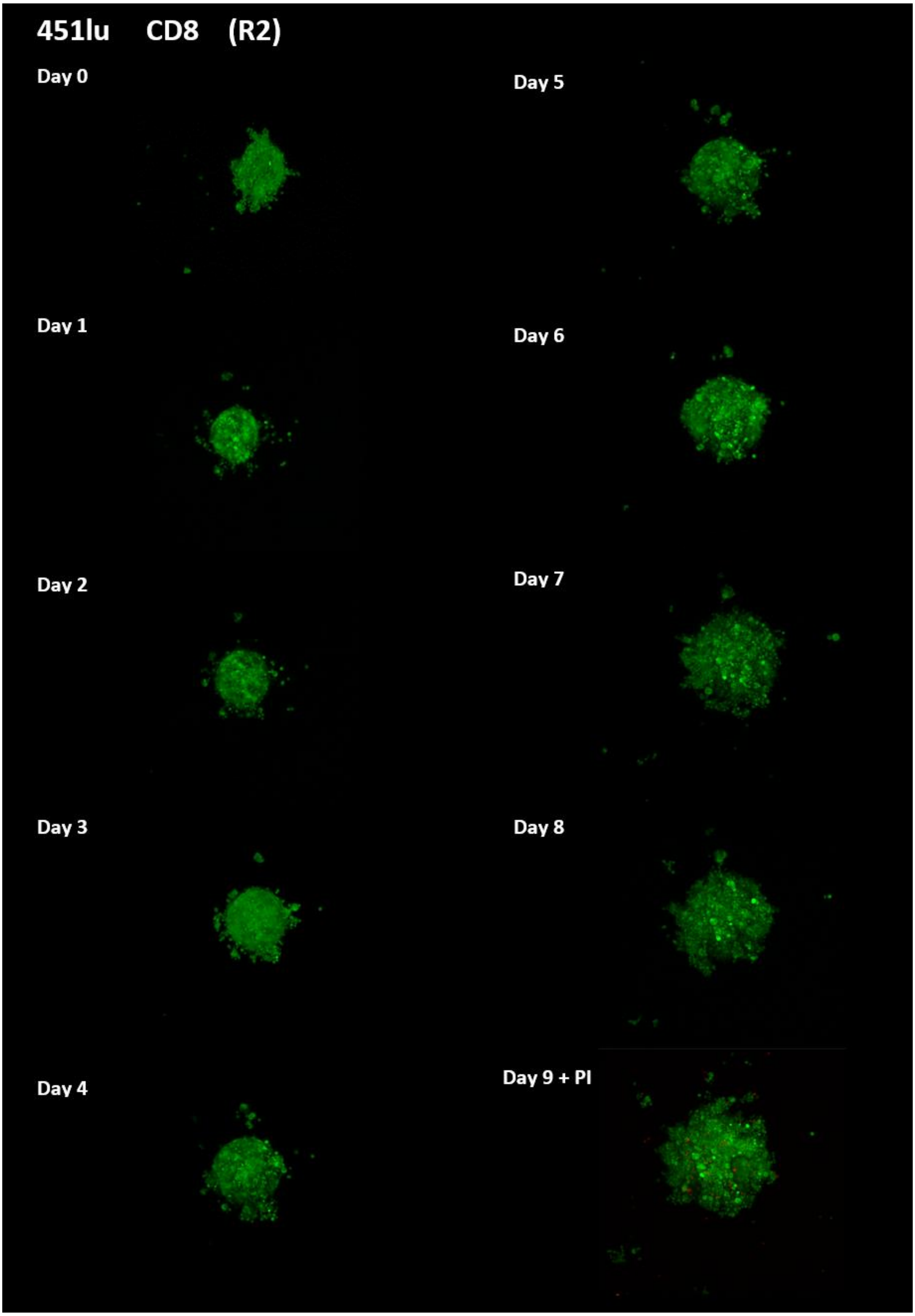
451lu CD8 (R2)
Day 0
Day 5
Day 1
Day 6
Day 2
Day 7
Day 3
Day 8
Day 4
Day 9 + PI

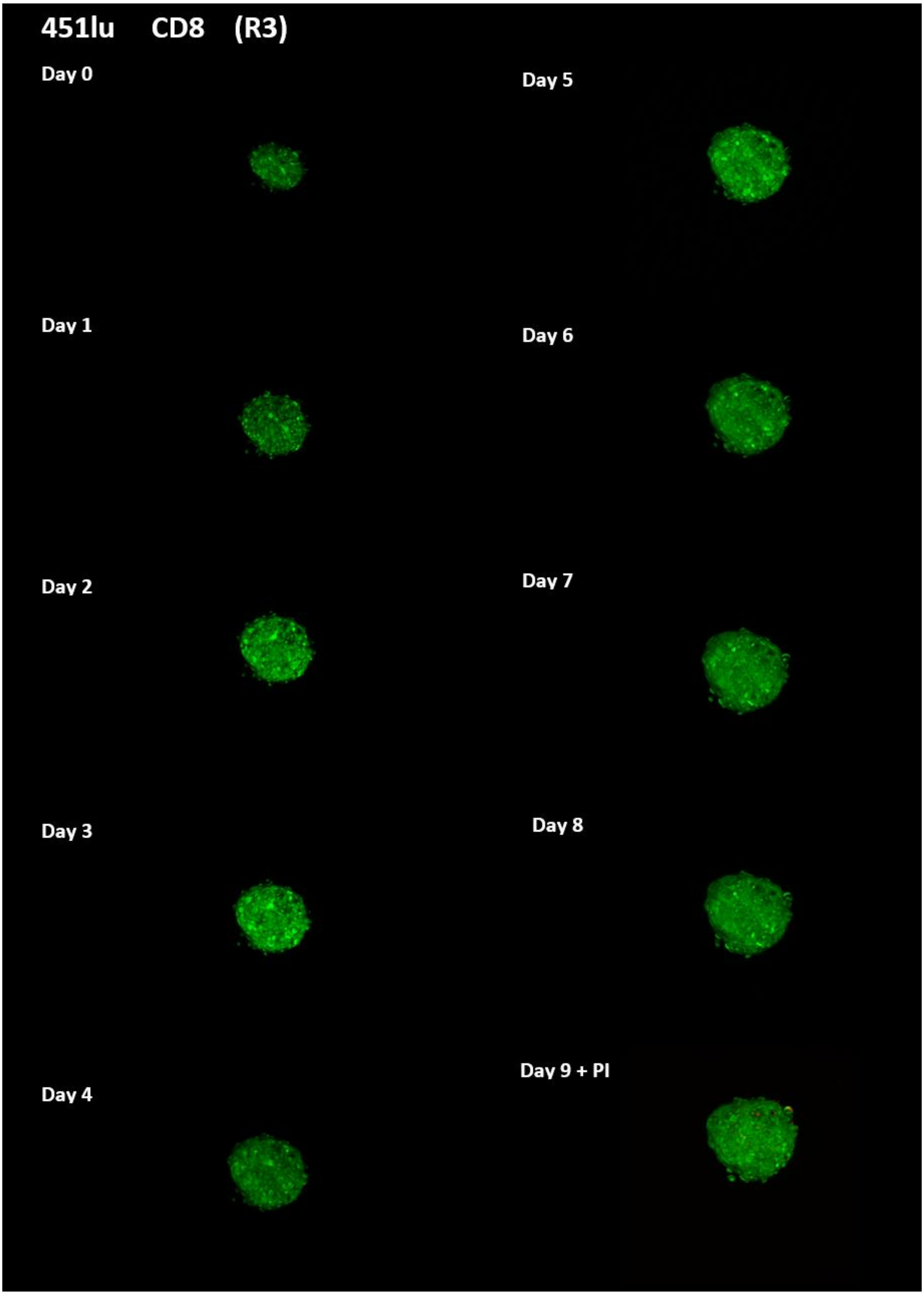
451lu CD8 (R3)
Day 0
Day 1
Day 2
Day 3
Day 4
Day 5
Day 6
Day 7
Day 8
Day 9 + PI

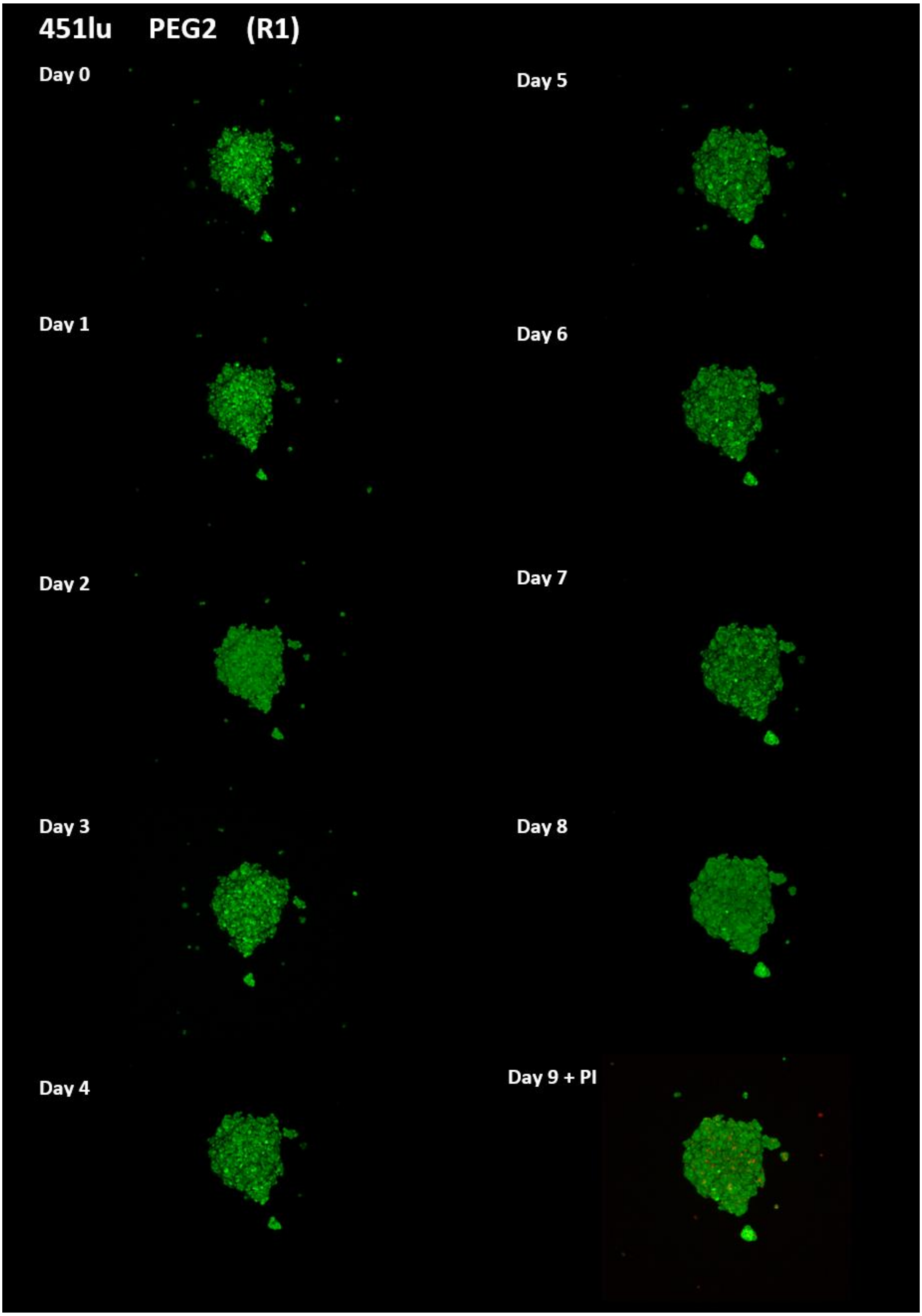
451lu PEG2 (R1)
Day 0
Day 5
Day 1
Day 6
Day 2
Day 7
Day 3
Day 8
Day 4
Day 9 + PI

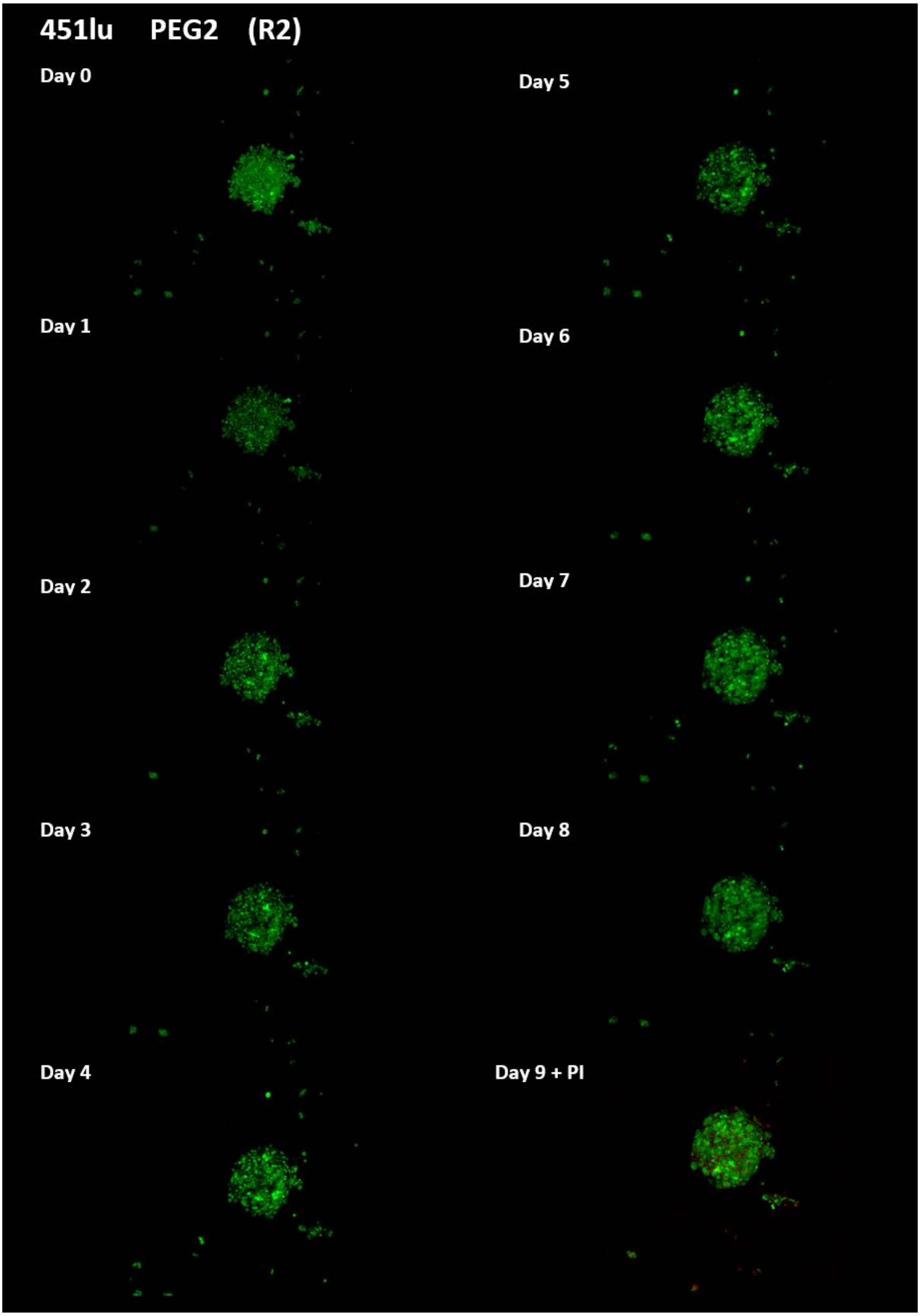
451lu PEG2 (R2)
Day 0
Day 5
Day 1
Day 6
Day 2
Day 7
Day 3
Day 8
Day 4
Day 9 + PI

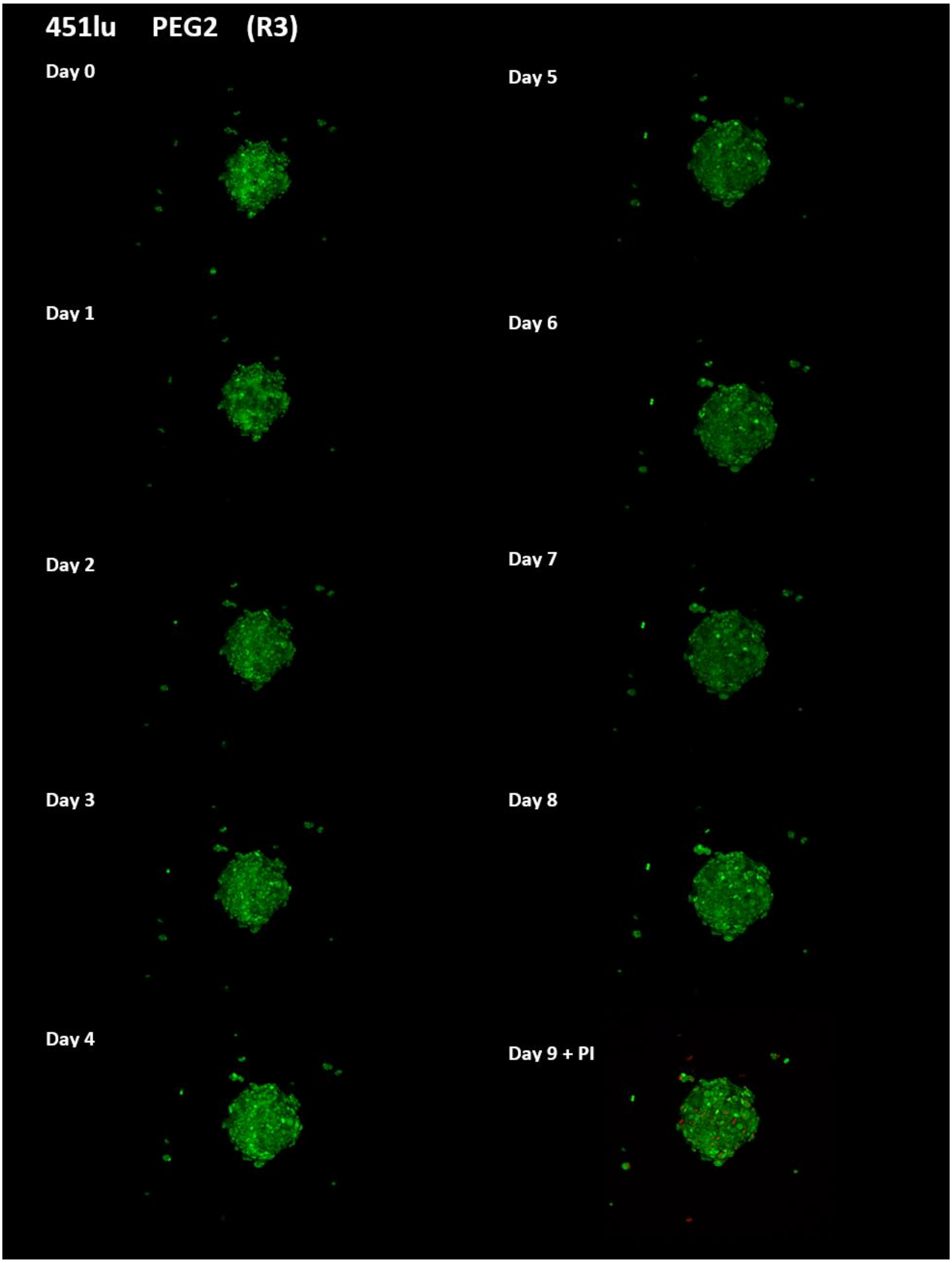
451lu PEG2 (R3)
Day 0
Day 5
Day 1
Day 6
Day 2
Day 7
Day 3
Day 8
Day 4
Day 9 + PI

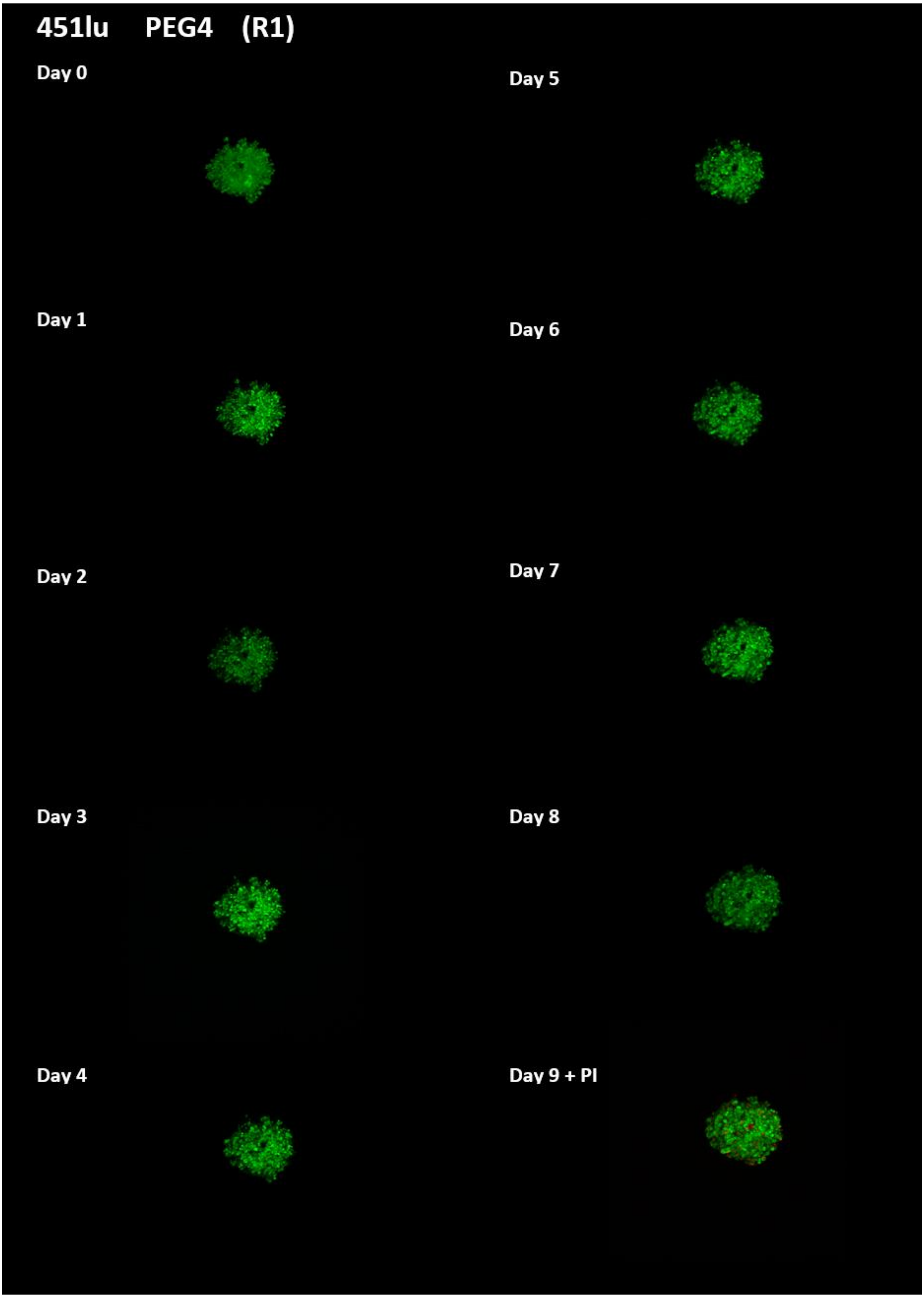
451lu PEG4 (R1)
Day 0
Day 5
Day 1
Day 6
Day 2
Day 7
Day 3
Day 8
Day 4
Day 9 + PI

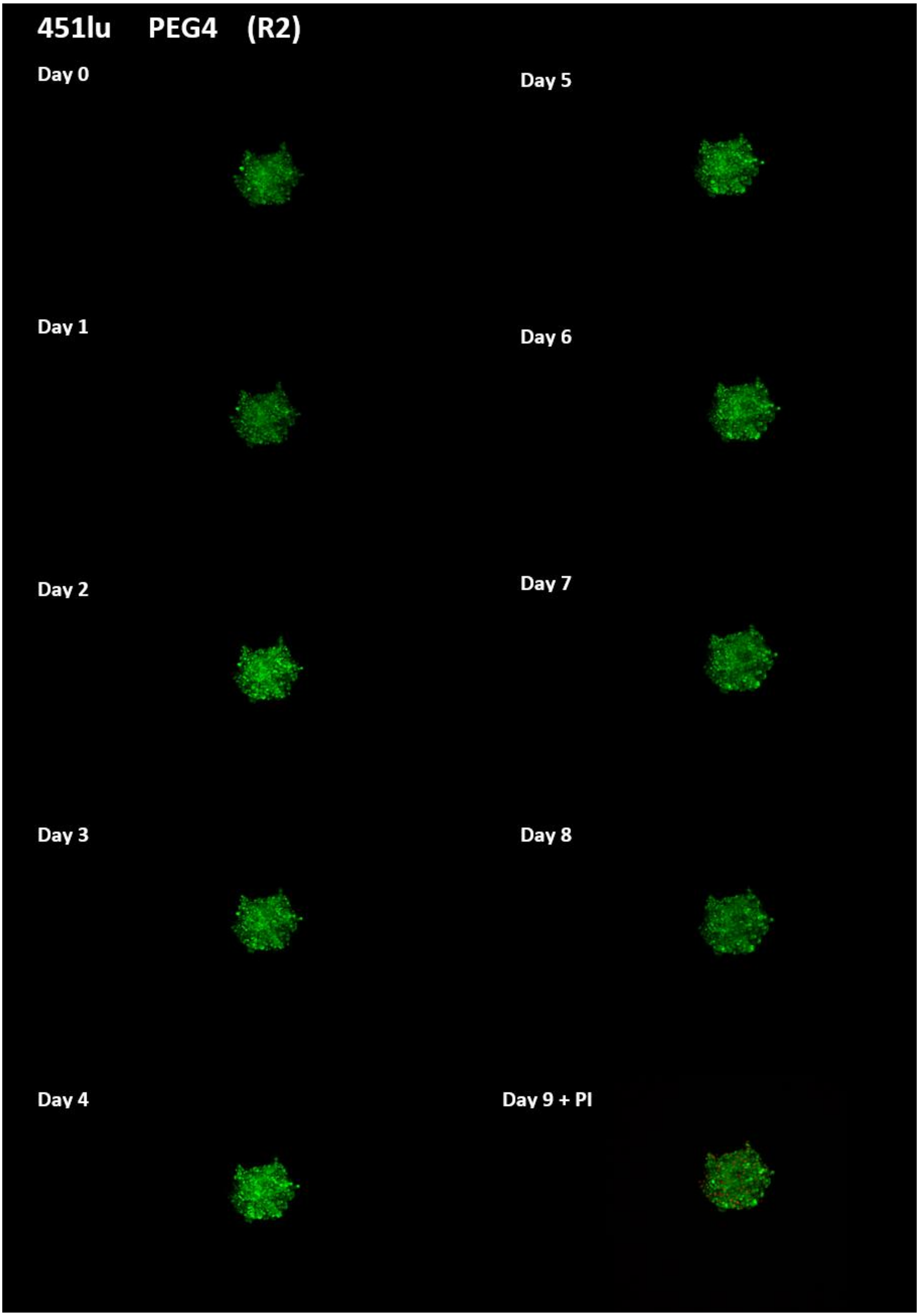
451lu PEG4 (R2)
Day 0
Day 5
Day 1
Day 6
Day 2
Day 7
Day 3
Day 8
Day 4
Day 9 + PI

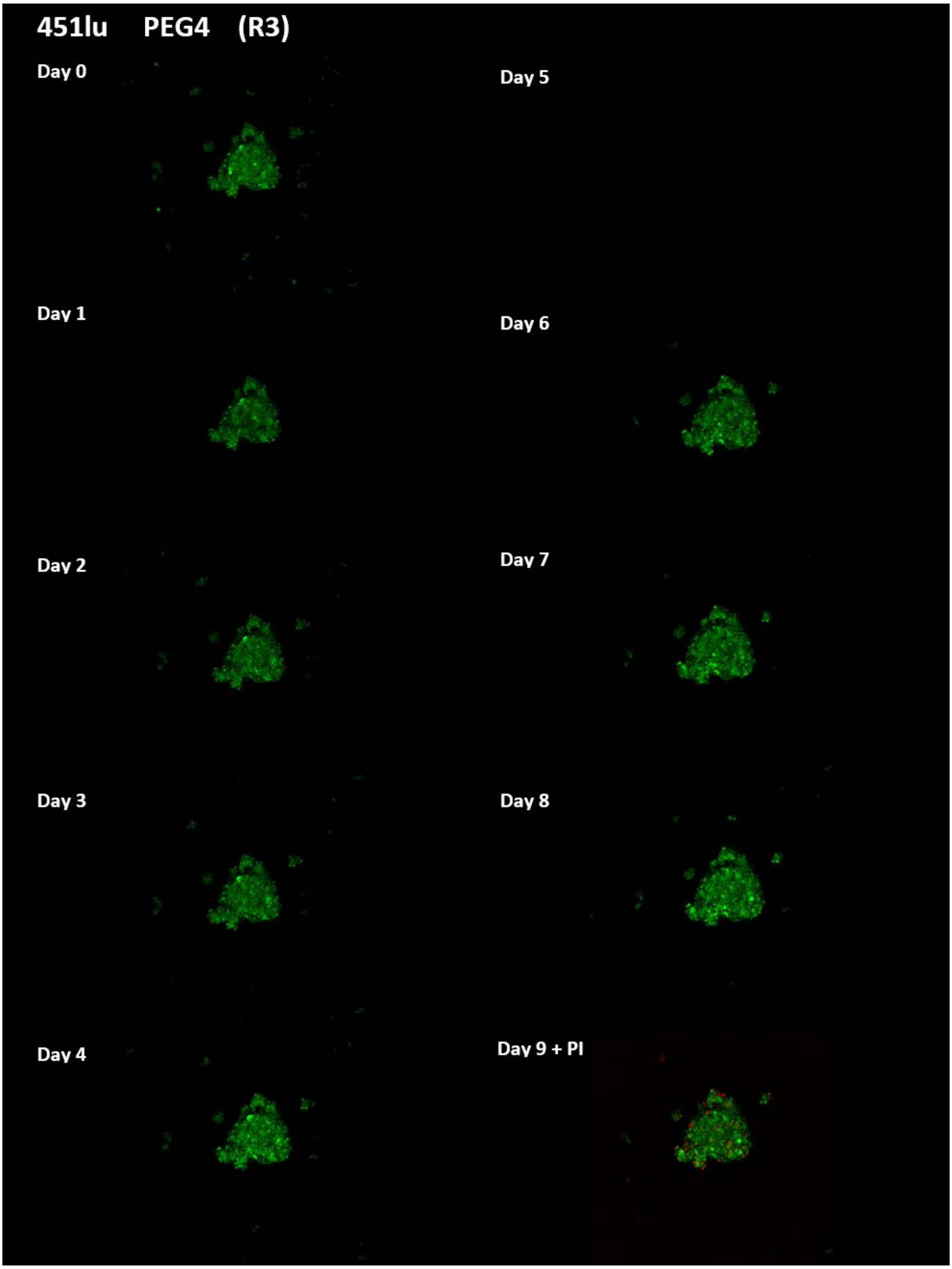
451lu PEG4 (R3)
Day 0
Day 5
Day 1
Day 6
Day 2
Day 7
Day 3
Day 8
Day 4
Day 9 + PI

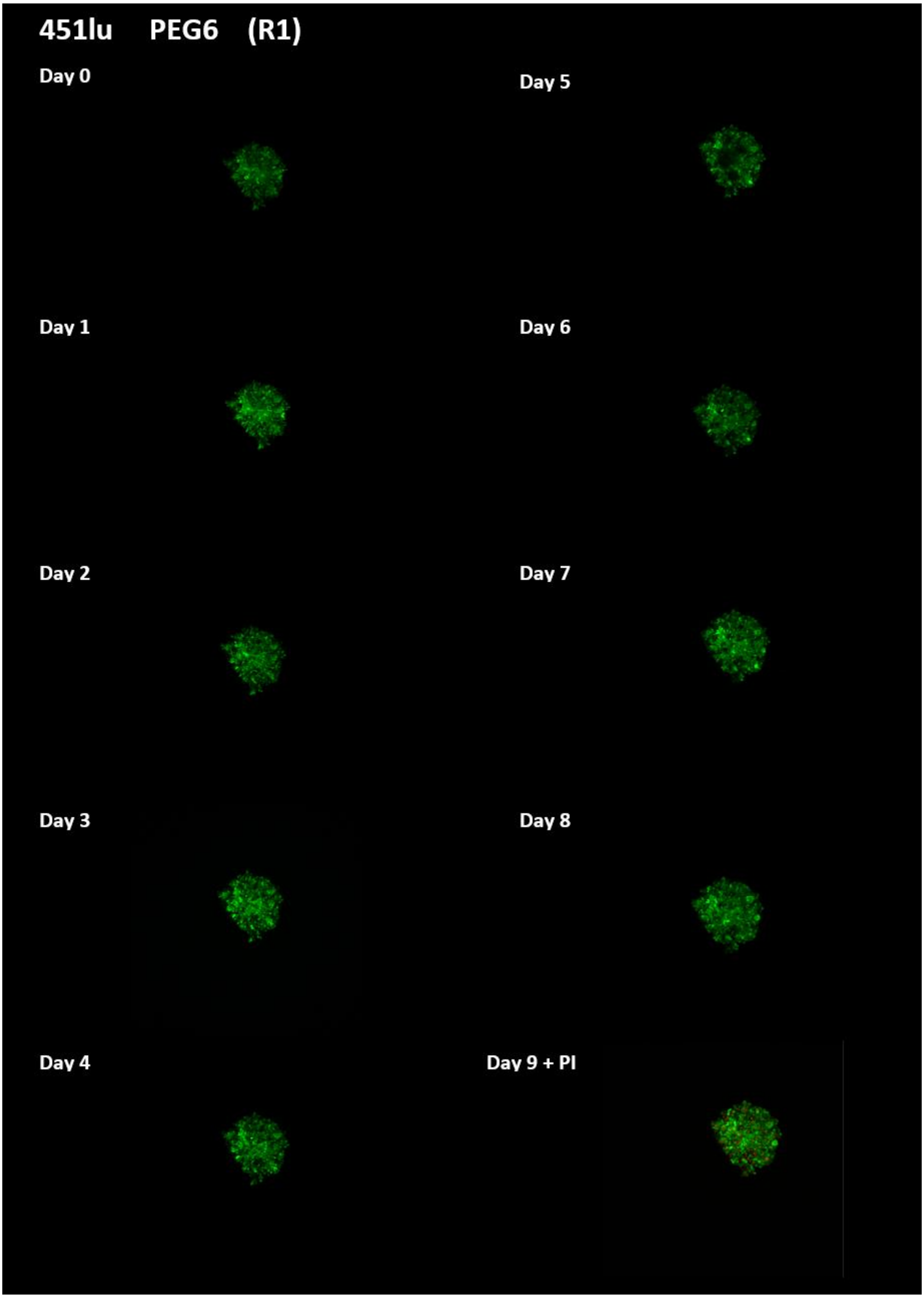
451lu PEG6 (R1)
Day 0
Day 5
Day 1
Day 6
Day 2
Day 7
Day 3
Day 8
Day 4
Day 9 + PI

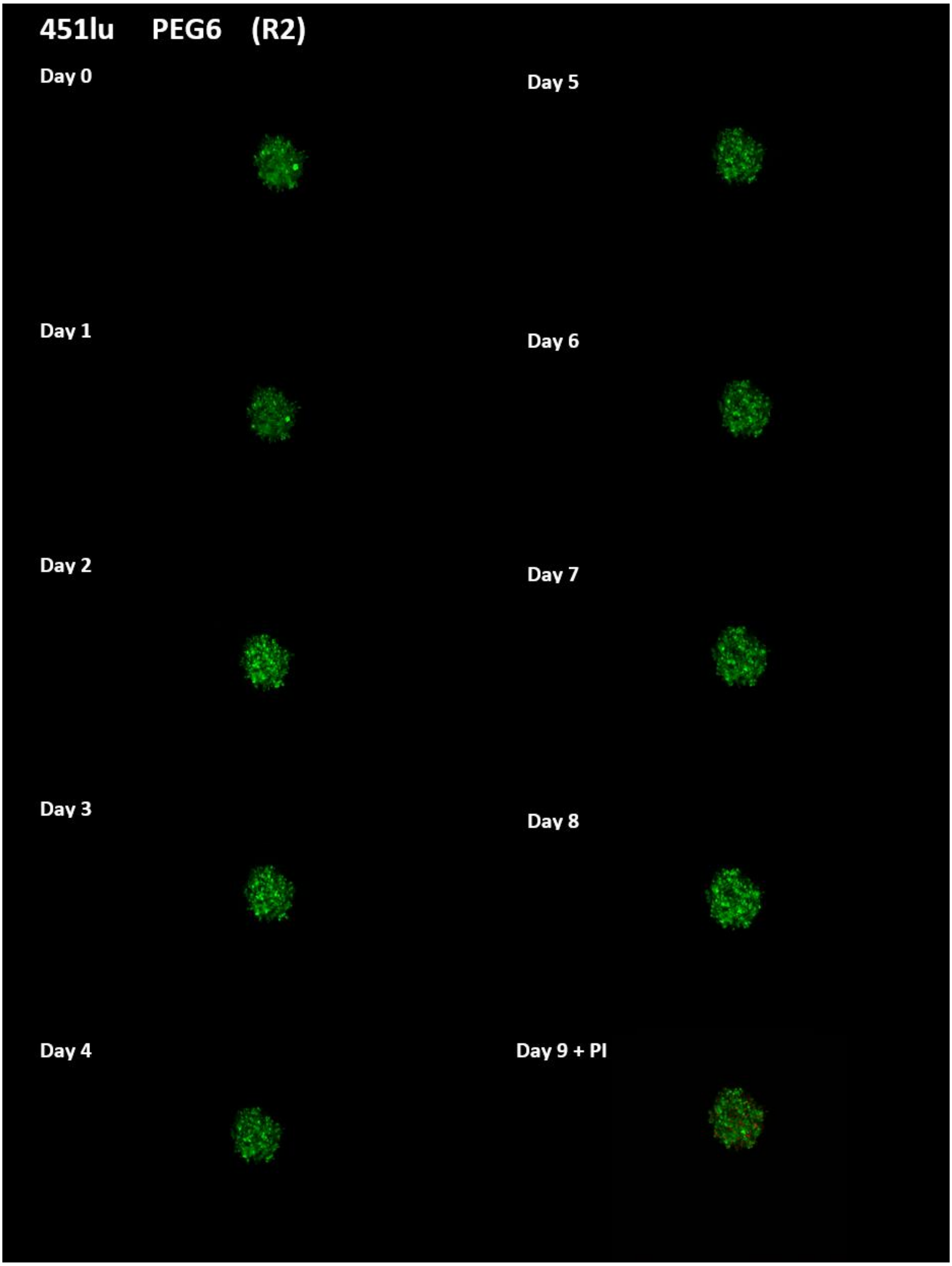
451lu PEG6 (R2)
Day 0
Day 5
Day 1
Day 6
Day 2
Day 7
Day 3
Day 8
Day 4
Day 9 + PI

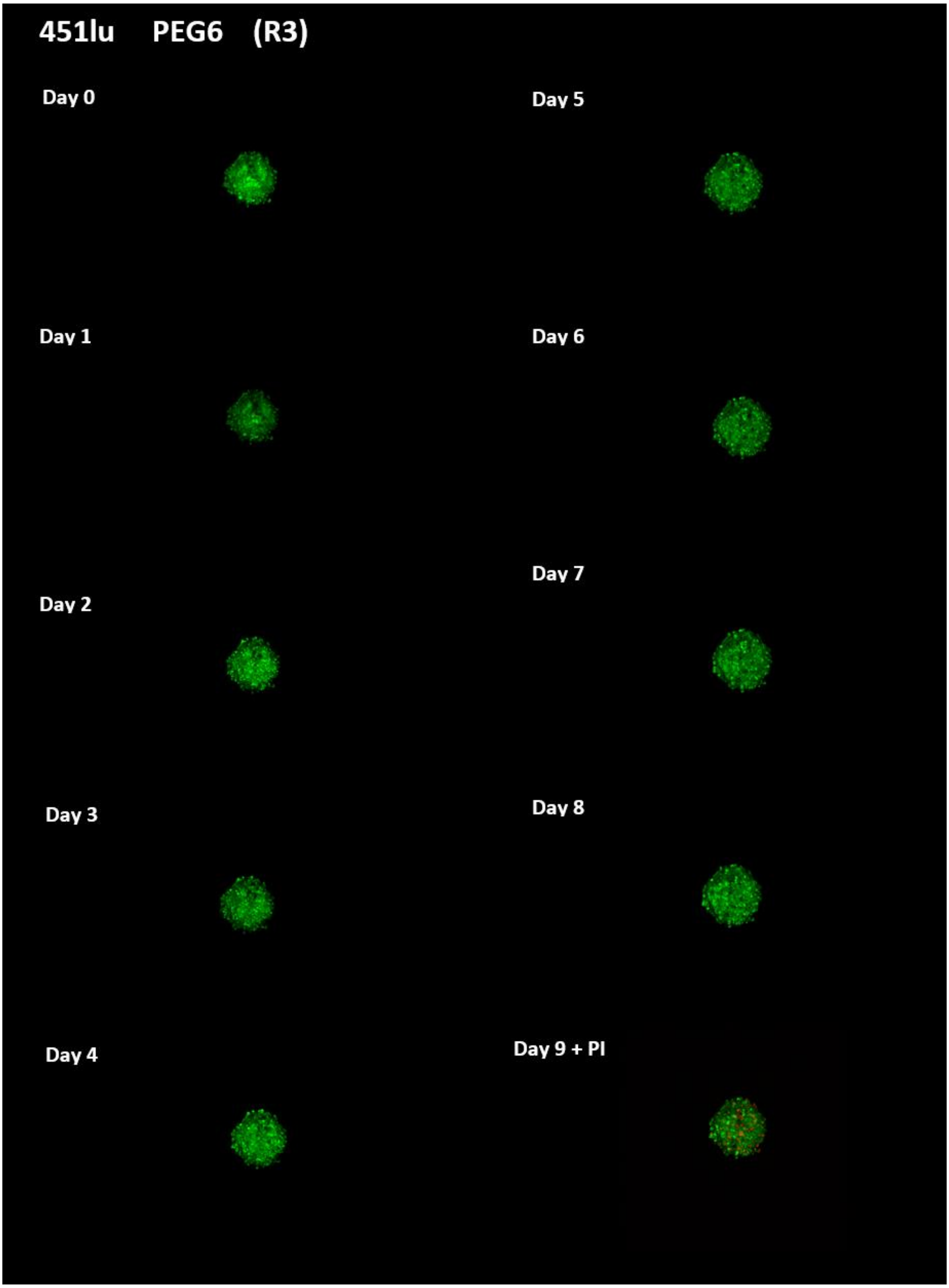
451lu PEG6 (R3)
Day 0
Day 5
Day 1
Day 6
Day 2
Day 7
Day 3
Day 8
Day 4
Day 9 + PI

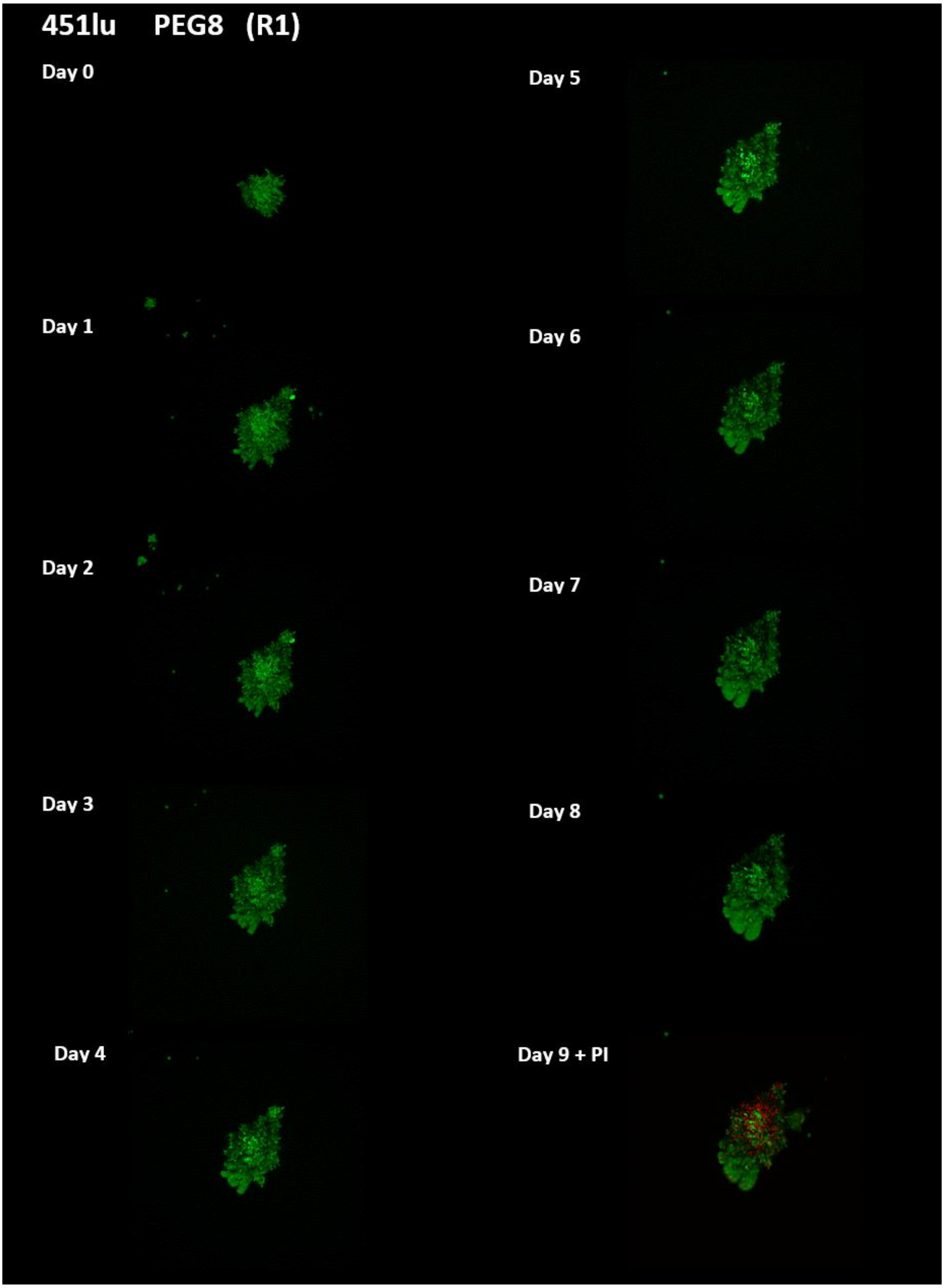
451lu PEG8 (R1)
Day 0
Day 1
Day 2
Day 3
Day 4
Day 5
Day 6
Day 7
Day 8
Day 9 + PI

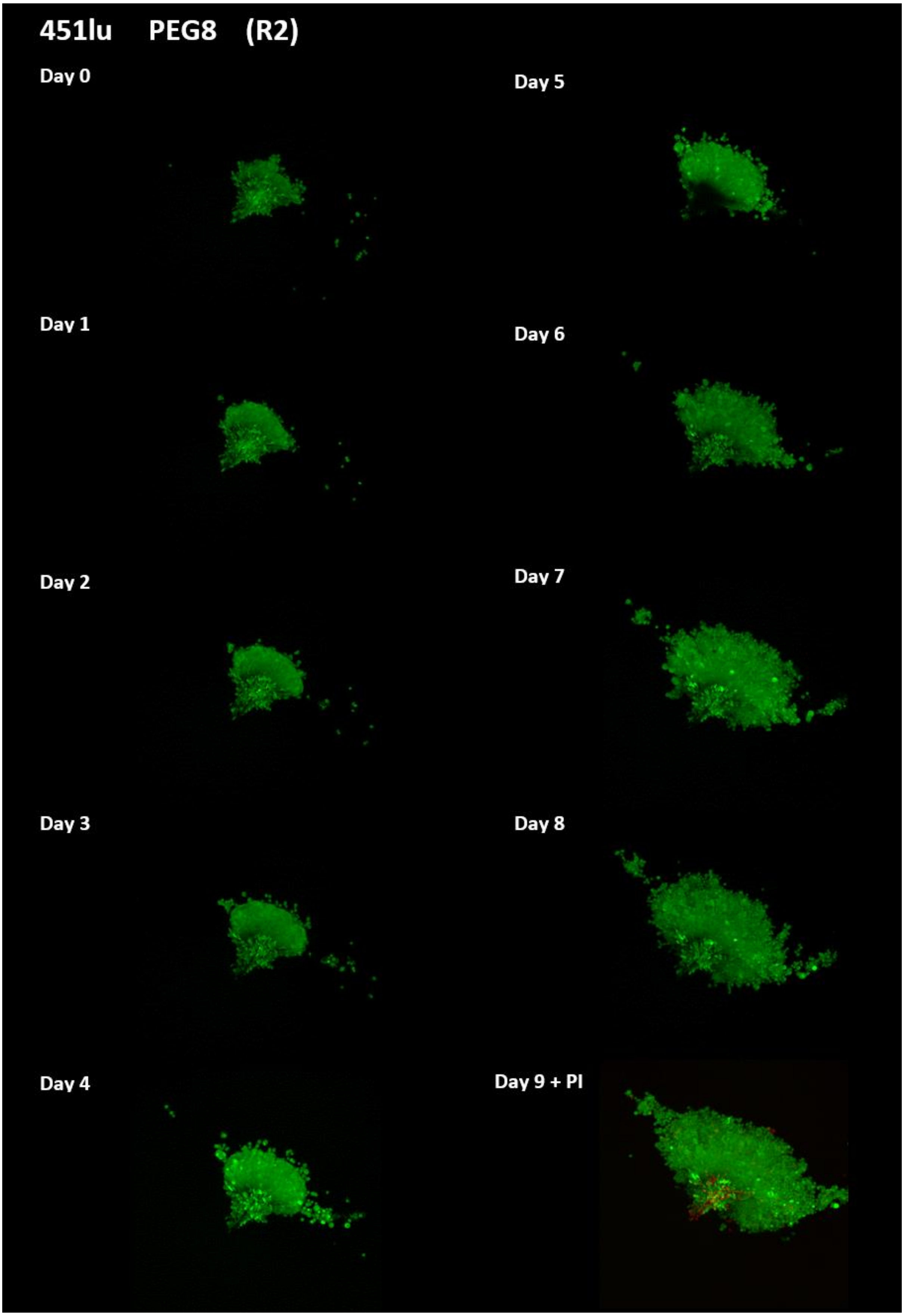
451lu PEG8 (R2)
Day 0
Day 5
Day 1
Day 6
Day 2
Day 7
Day 3
Day 8
Day 4
Day 9 + PI

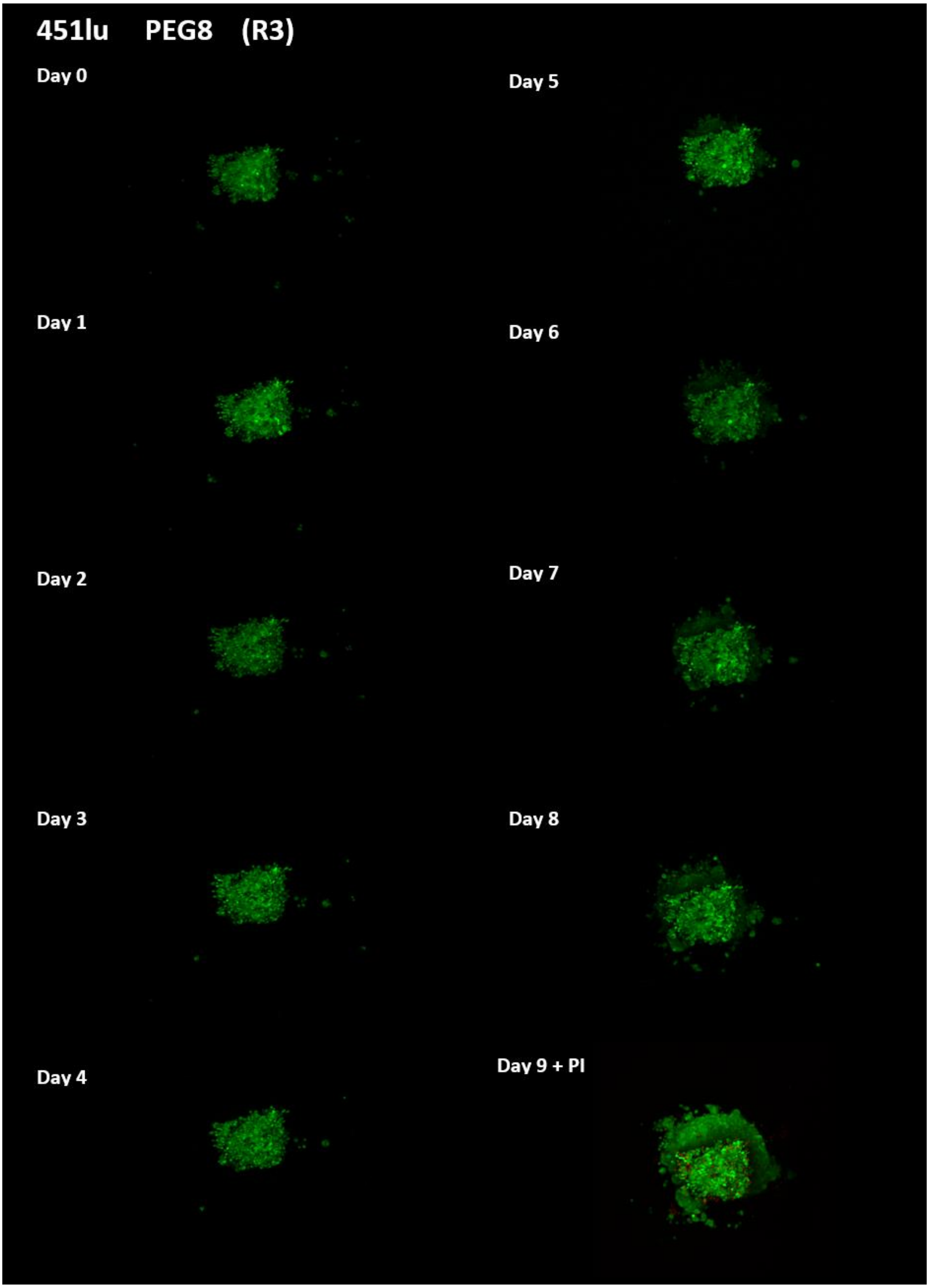
451lu PEG8 (R3)
Day 0
Day 5
Day 1
Day 6
Day 2
Day 7
Day 3
Day 8
Day 4
Day 9 + PI
