# Supplementary material for: Mechanistically Coupled PK (MCPK) Model to Describe Enzyme Induction and Occupancy Dependent DDI of Dabrafenib Metabolism
Source: Pharmaceutics. 2022 Jan 28;14(2):310. doi: 10.3390/pharmaceutics14020310 (PMC8875124; doi:10.3390/pharmaceutics14020310)
Supplement: Supplementary file 1 [file pharmaceutics-14-00310-s001.zip › pharmaceutics-1479084-supplementary/Supplementary_Information/Supplemental_Information_S4.pdf]

Supplemental Information S4: Mechanistically Coupled PK (MCPK) model to describe enzyme induction and occupancy dependent DDI of dabrafenib metabolism

Marco Albrecht[1,2], Yuri Kogan[3], Dagmar Kulms[4], and Thomas Sauter[5]

[1] *Université du Luxembourg; Systems Biology Group; 4367 Belvaux; Luxembourg; Email: marco.albrecht@posteo.eu*
[2] *esqLABS GmbH; 26683 Saterland; Germany; Email: marco.albrecht@esqlabs.com*
[3] *Institute for Medical Biomathematics (IMBM); 60991 Bnei Atarot; Israel; Email: yuri@imbm.com*
[4] *Technical University of Dresden; Experimental Dermatology; 01307 Dresden; Germany; Tel.: +49 (0)351 458-18973 ; Email: dagmar.kulms@uniklinikum-dresden.de*
[5] *Université du Luxembourg; Systems Biology Group; 4367 Belvaux; Luxembourg; Tel.: (+352) 46 66 44 6296; Email: thomas.sauter@uni.lu*

Spheroid Image Data of A375 Spheroid Growth

Spheroid Image Data of A375 Spheroid Growth 1
Legend 1
A375– First Iteration 2
A375– Second Iteration (with or without Fibronectin) 6
A375– Third Iteration 19

Legend

CD: Gel with cell degradable linker
PEG: Gel with non-degradable polyethylene glycol linker
R1-3: Biological Replicate 1 to 3.
A375: Metastatic Melanoma Cell Line with **green** flourecent protein
PI: Propidium iodide as a **red** cell death marker at last day

A375– First Iteration

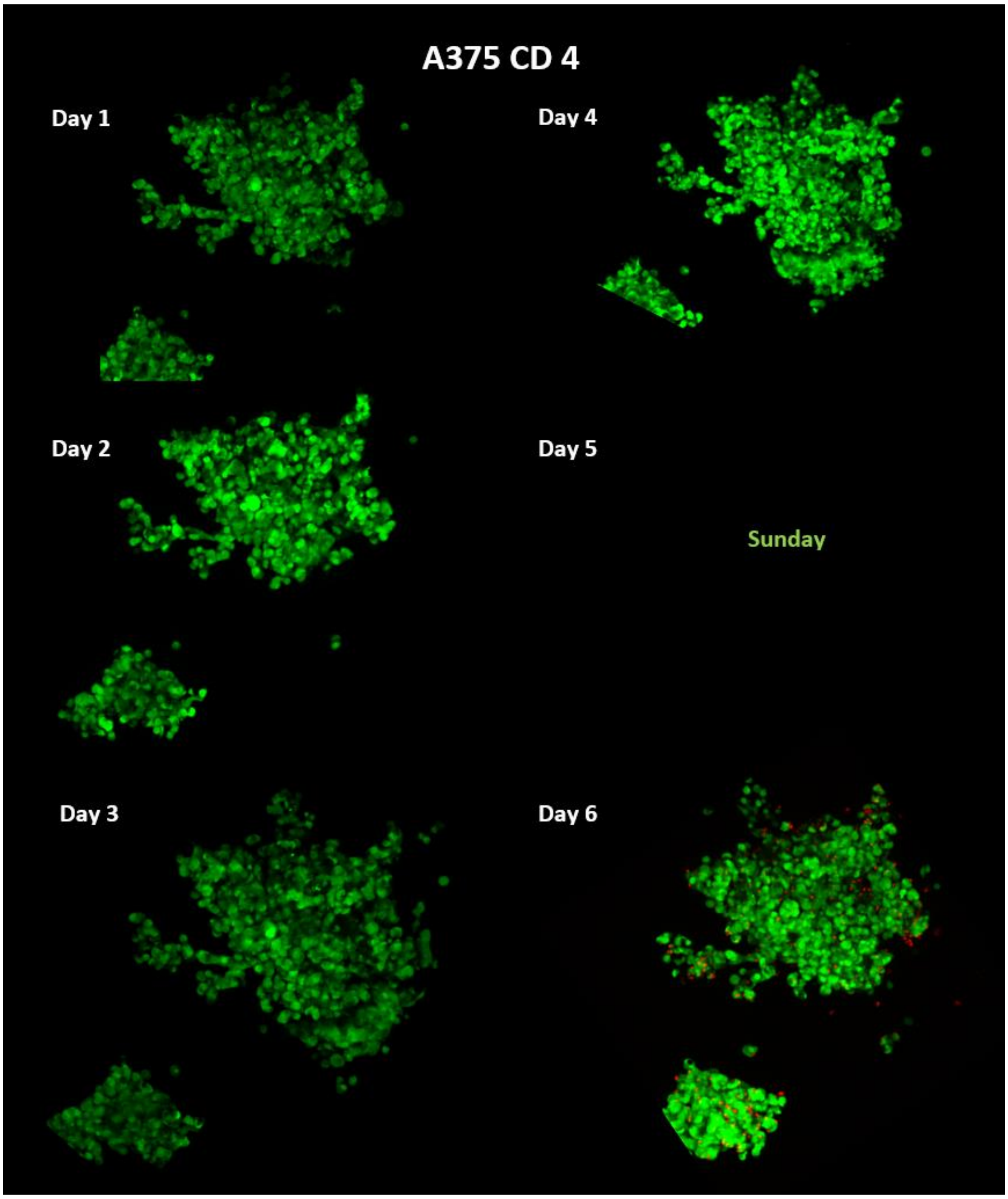

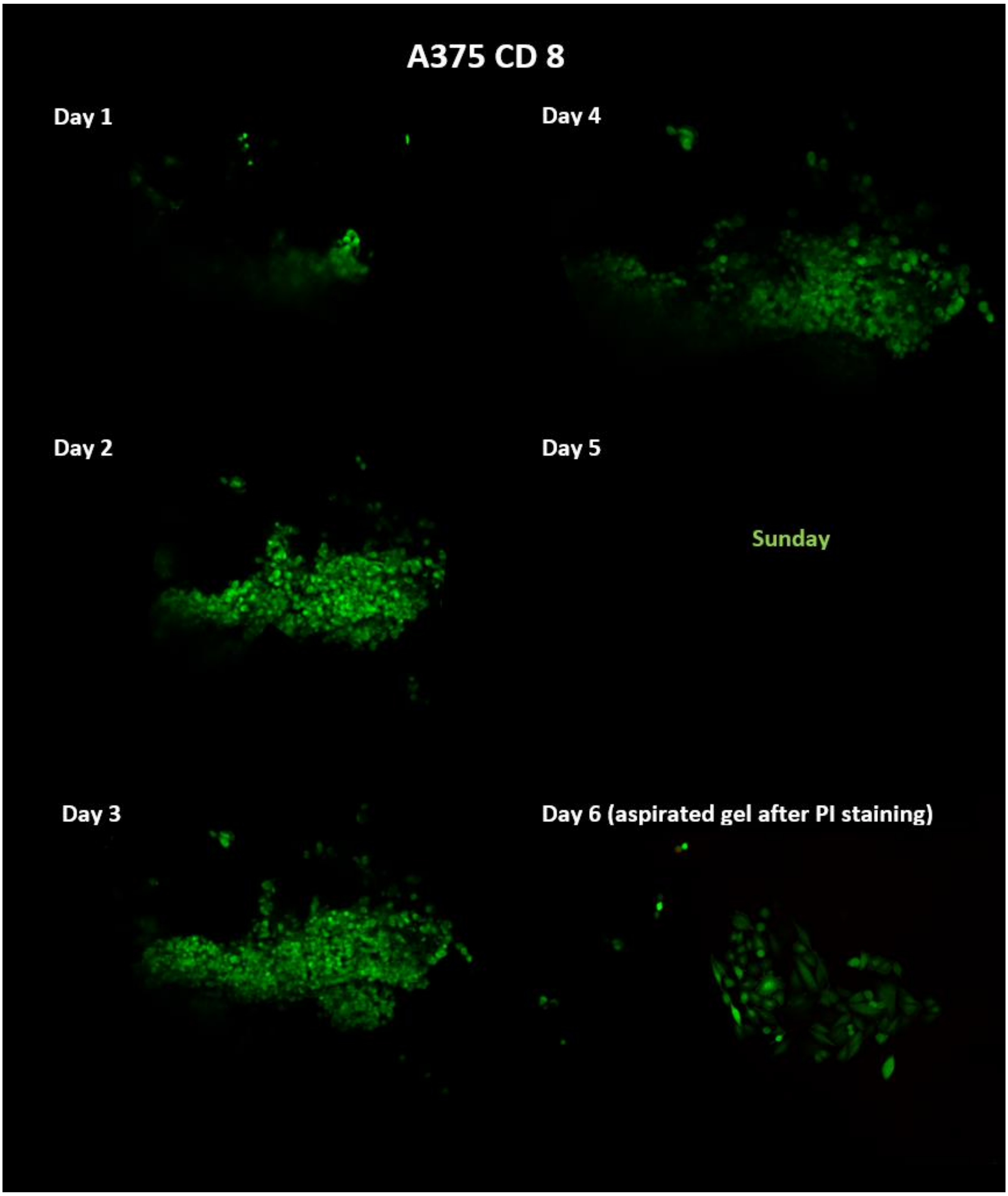
A375 CD 8
Day 1
Day 4
Day 2
Day 5
Sunday
Day 3
Day 6 (aspirated gel after PI staining)

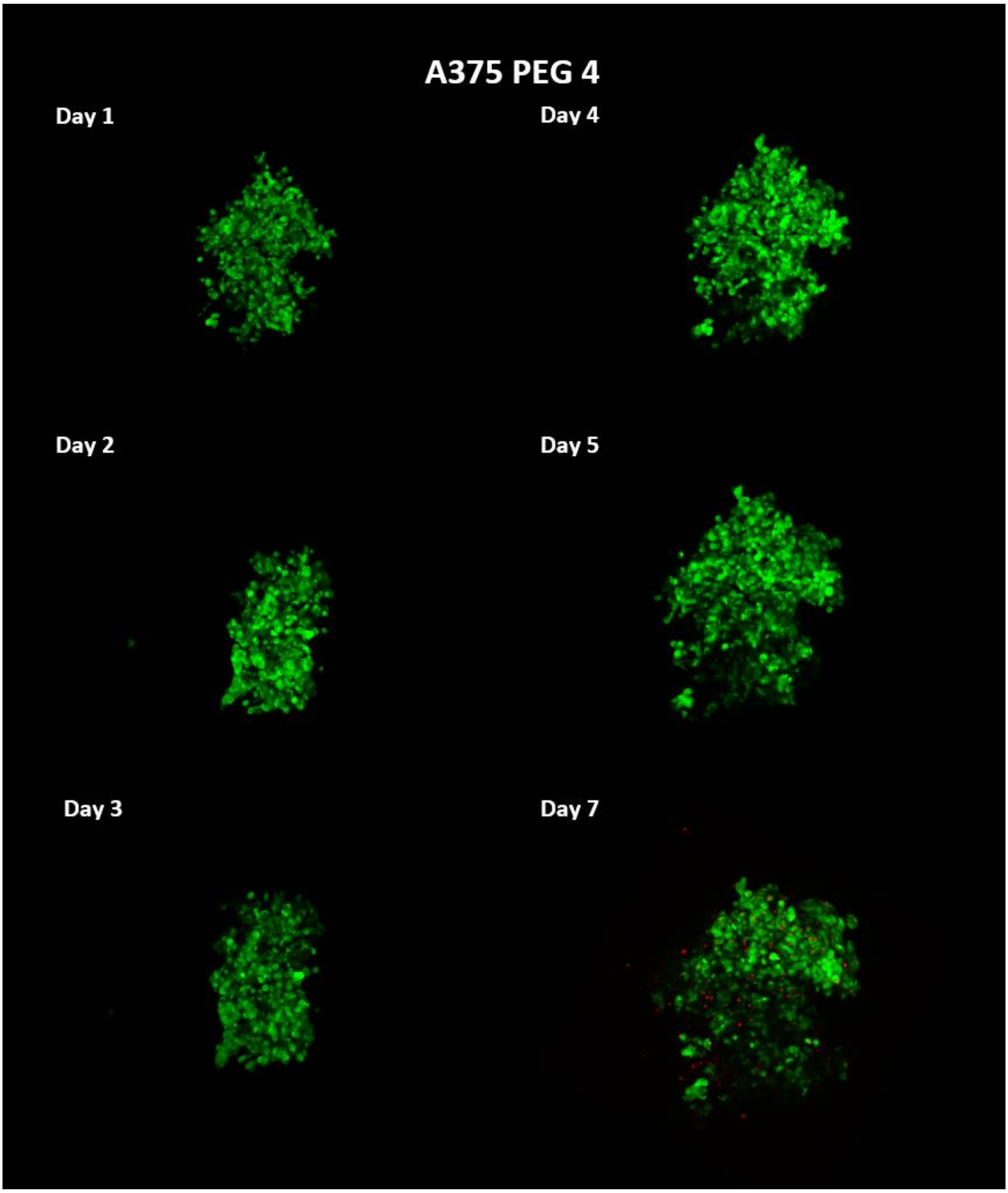
A375 PEG 4
Day 1
Day 4
Day 2
Day 5
Day 3
Day 7

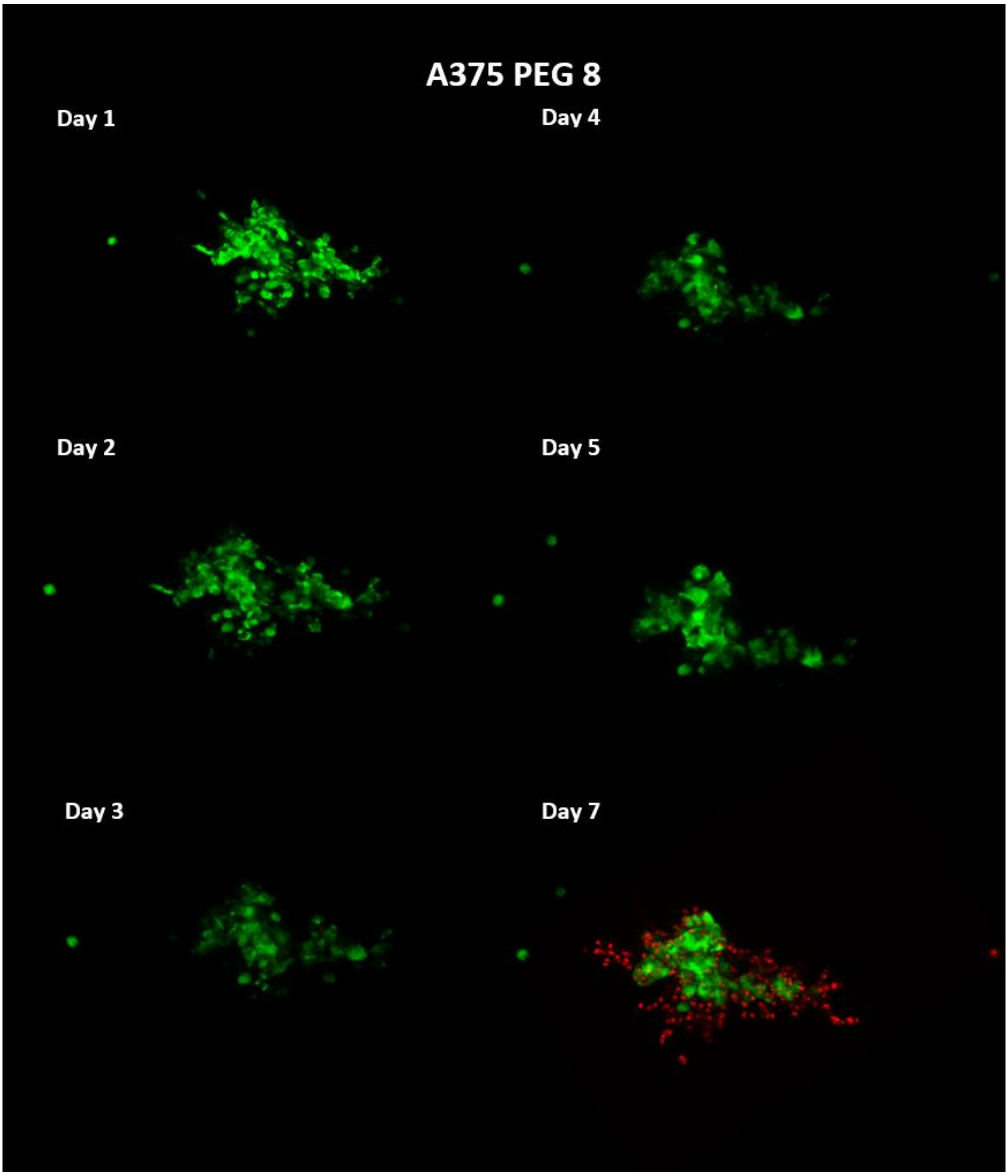
A375 PEG 8
Day 1
Day 4
Day 2
Day 5
Day 3
Day 7

A375– Second Iteration (with or without Fibronectin)

2	2 without Fibronectin	4	4 without Fibronectin	6	8
14.8	16.6	10.7	12.5	5.7	0.7
2.4	2.4	2.5	2.5	2.5	2.5
2	2	4	4	6	8
1.8	0	1.8	0	1.8	1.8
6	6	5	5	5	5
3	3	6	6	9	12
30	30	30	30	30	30

Ingredients	PEG Link	CD-Link
Water	539	539
10xCB PH 7.2	580	485
Thiol-reactive polymer	544	544
Fibronectin	SLBP0436V	SLBP0436V
Cell suspension		
CD or PEG Link	565	553

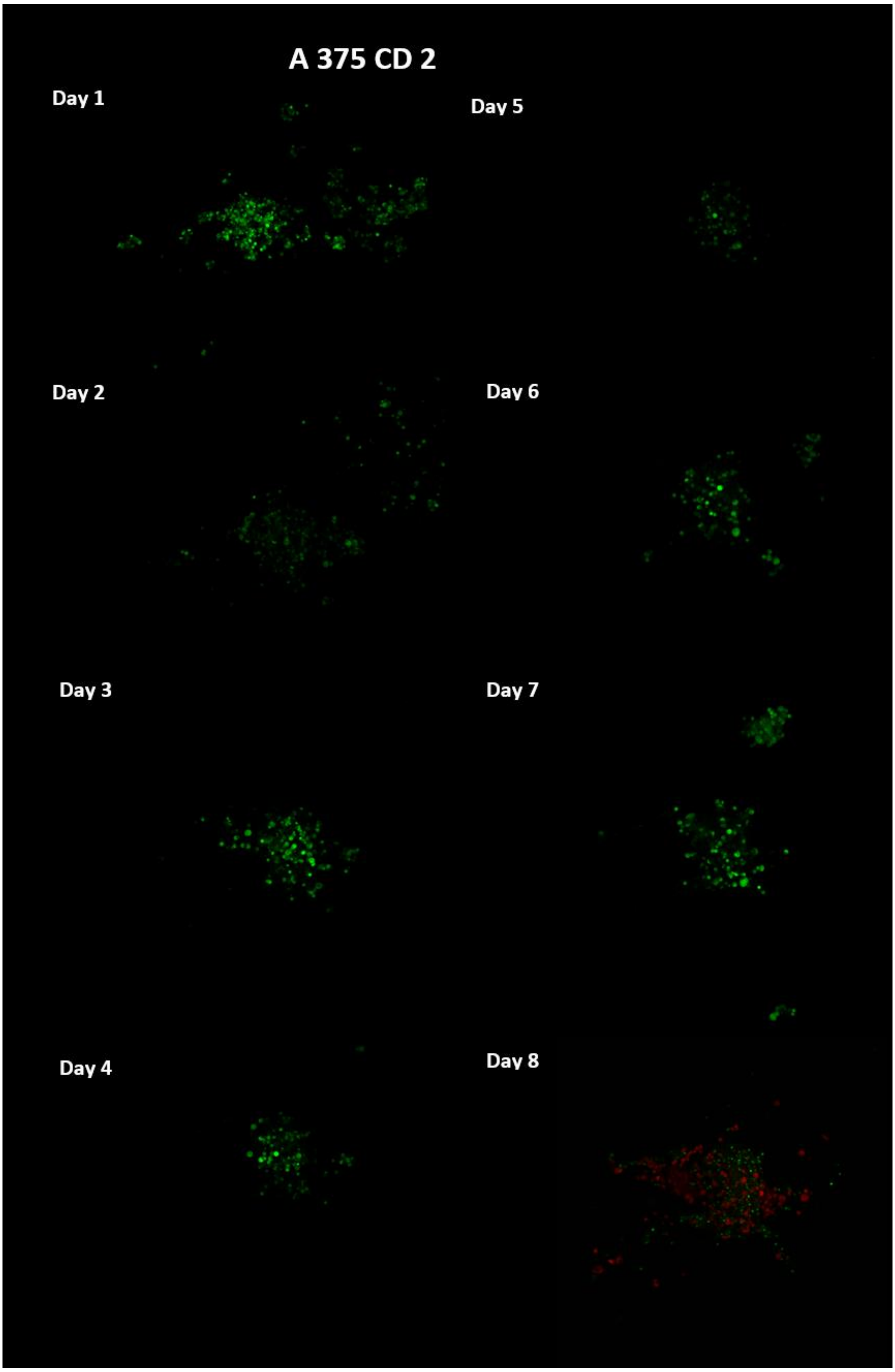
A 375 CD 2
Day 1
Day 5
Day 2
Day 6
Day 3
Day 7
Day 4
Day 8

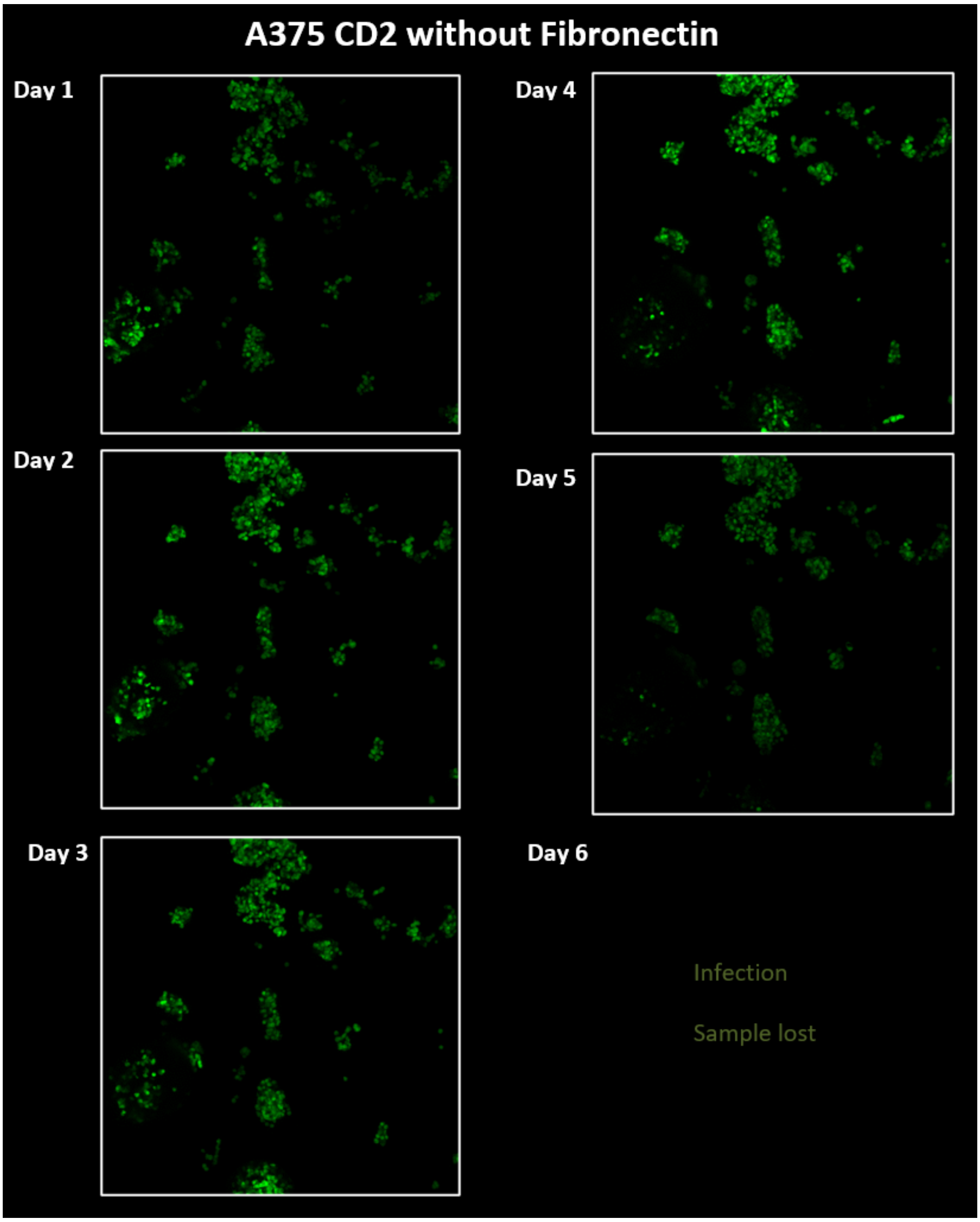
A375 CD2 without Fibronectin
Day 1
Day 4
Day 2
Day 5
Day 3
Day 6
Infection
Sample lost

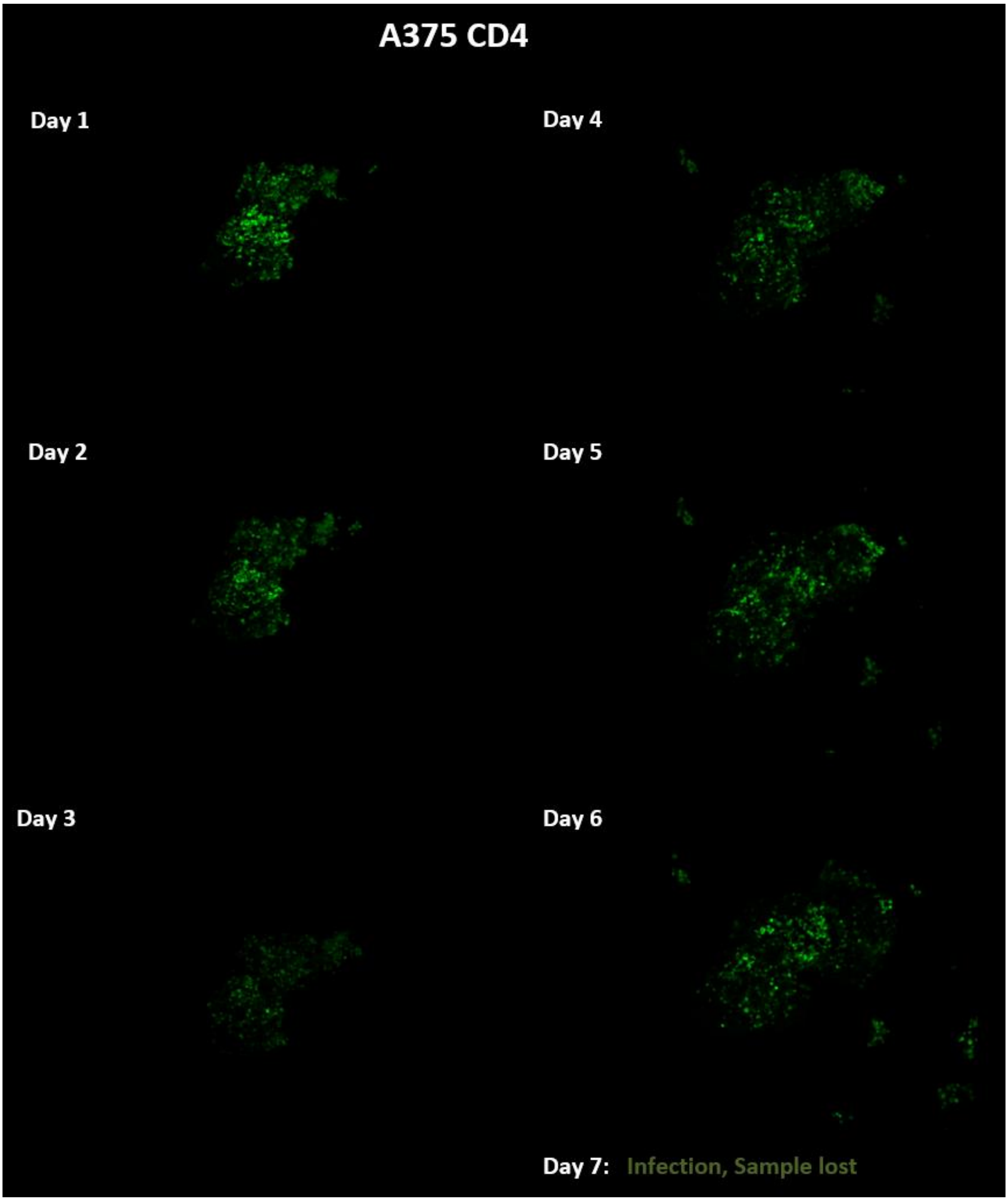
A375 CD4
Day 1
Day 4
Day 2
Day 5
Day 3
Day 6
Day 7: Infection, Sample lost

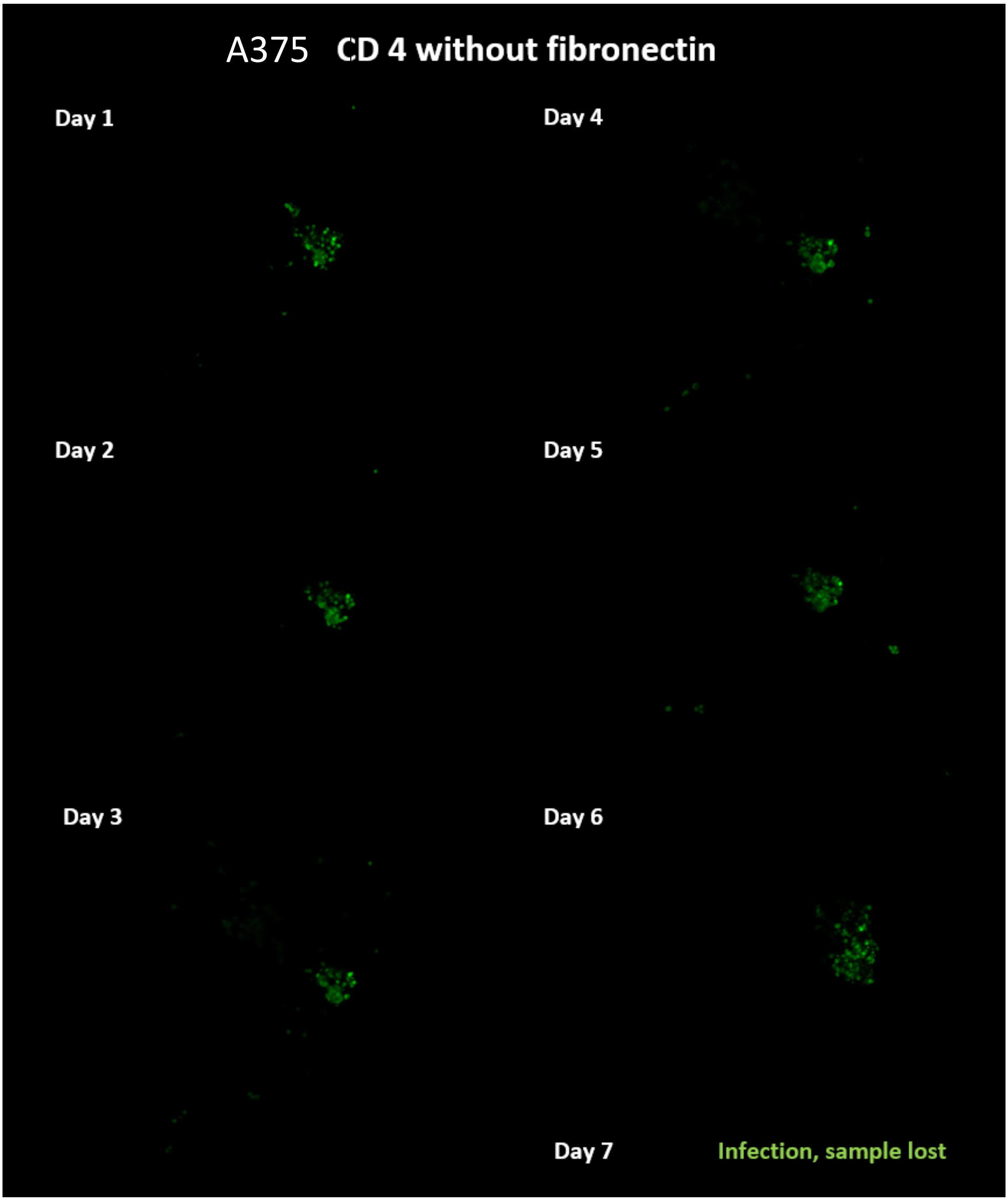
A375 CD 4 without fibronectin
Day 1
Day 4
Day 2
Day 5
Day 3
Day 6
Day 7
Infection, sample lost

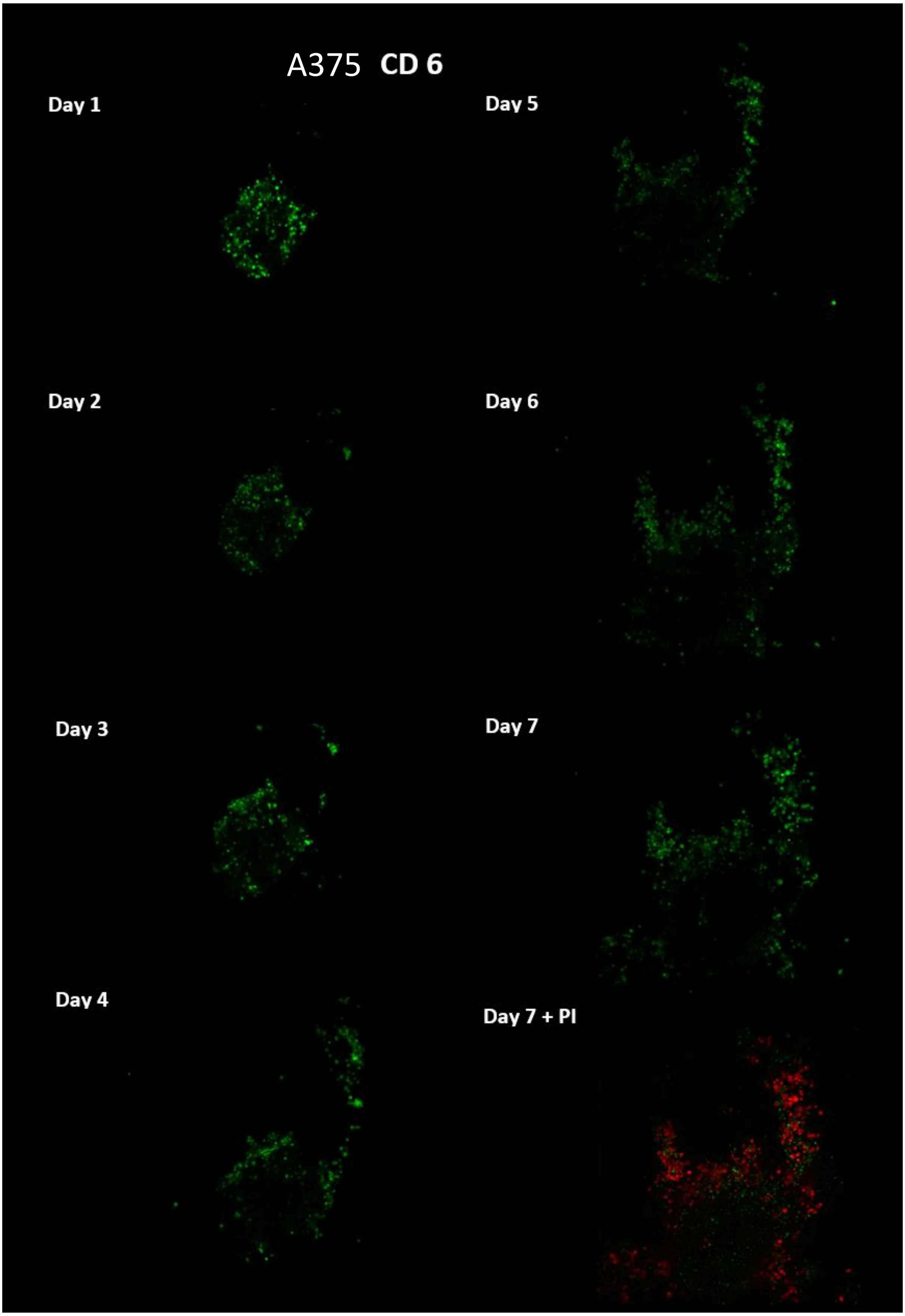
A375 CD 6
Day 1
Day 5
Day 2
Day 6
Day 3
Day 7
Day 4
Day 7 + PI

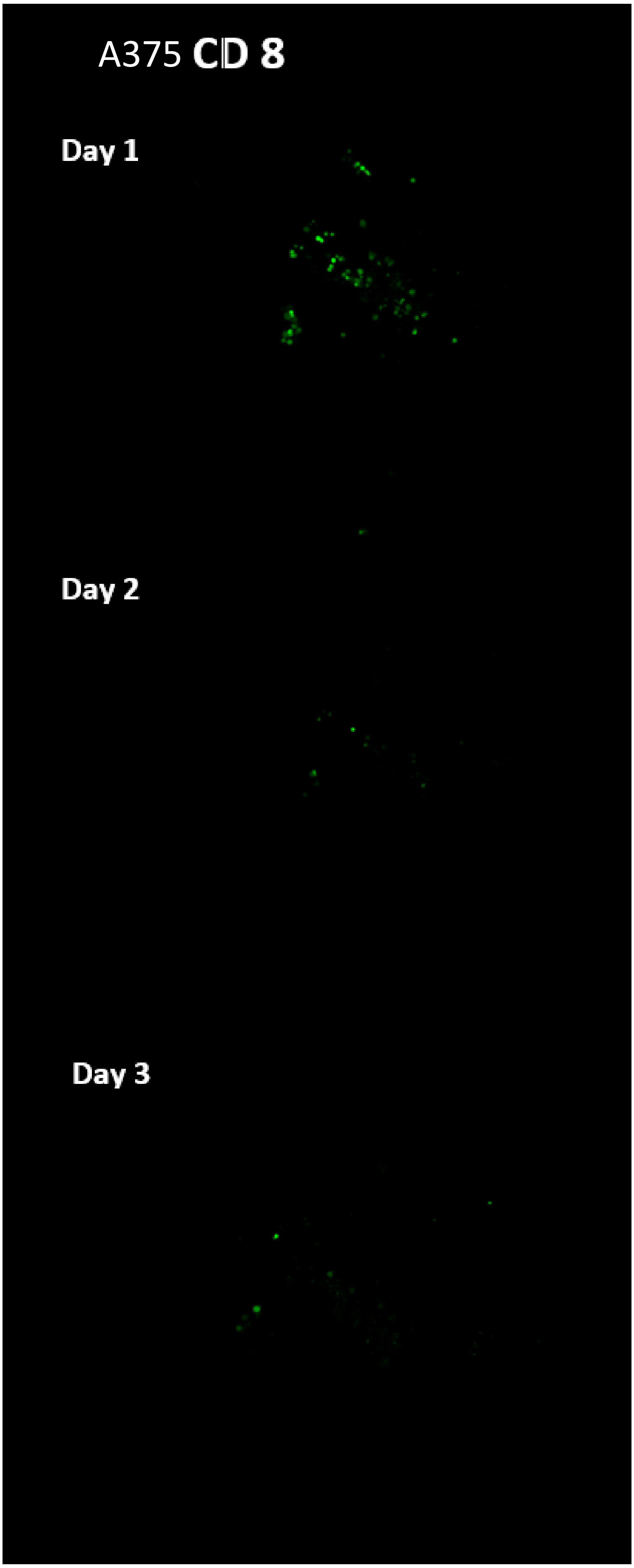
A375 CD 8
Day 1
Day 2
Day 3

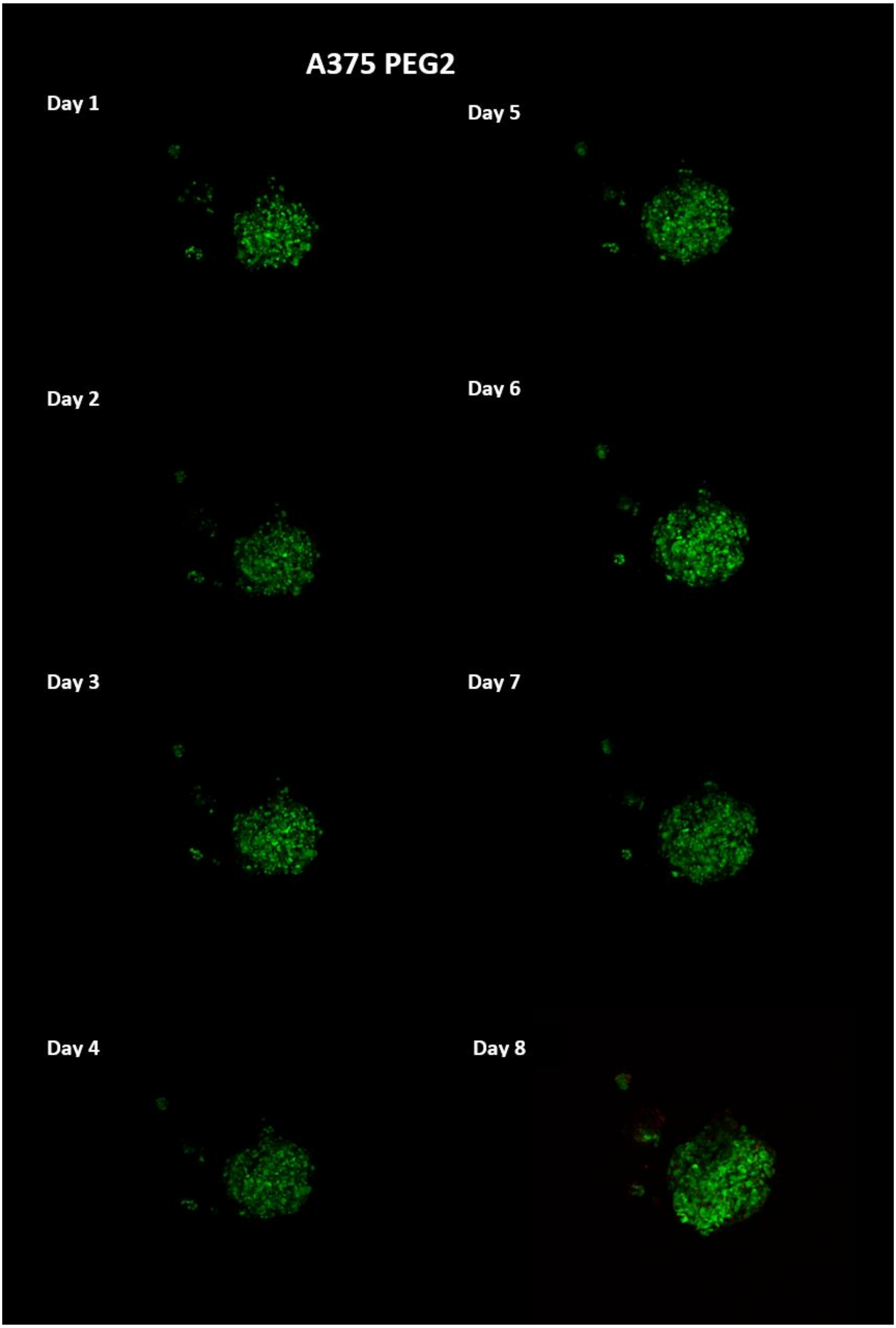
A375 PEG2
Day 1
Day 5
Day 2
Day 6
Day 3
Day 7
Day 4
Day 8

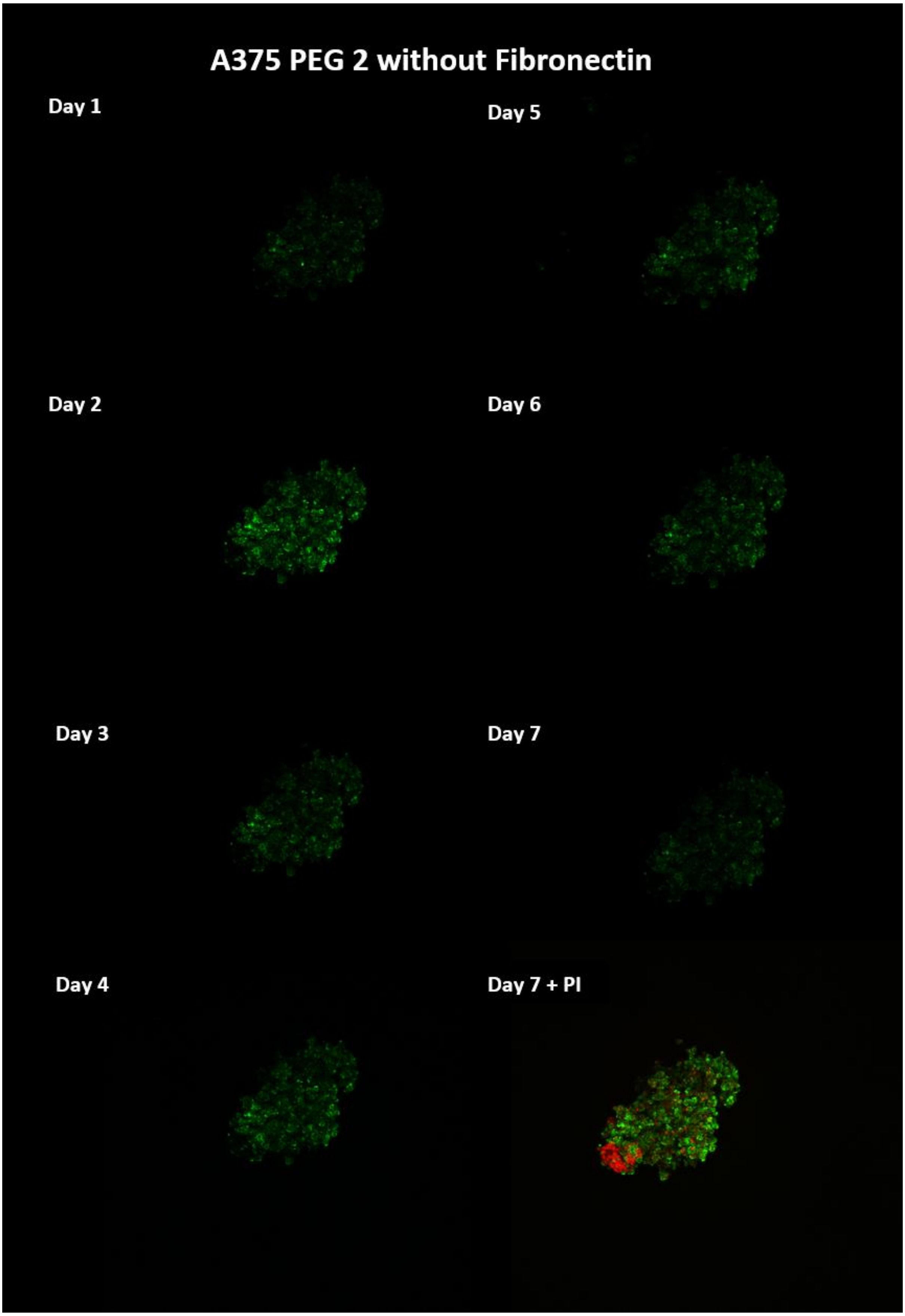
A375 PEG 2 without Fibronectin
Day 1
Day 5
Day 2
Day 6
Day 3
Day 7
Day 4
Day 7 + PI

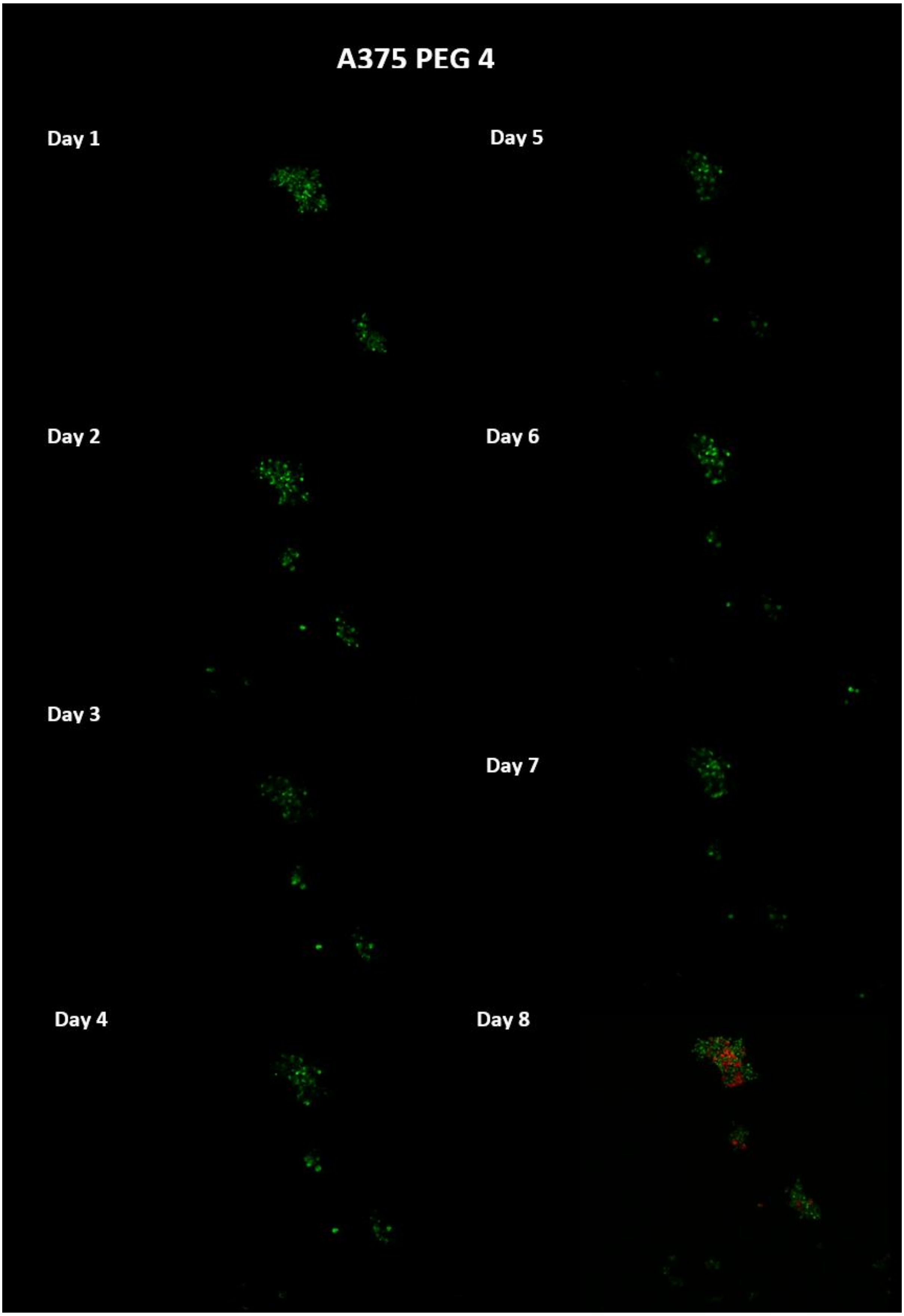
A375 PEG 4
Day 1
Day 5
Day 2
Day 6
Day 3
Day 7
Day 4
Day 8

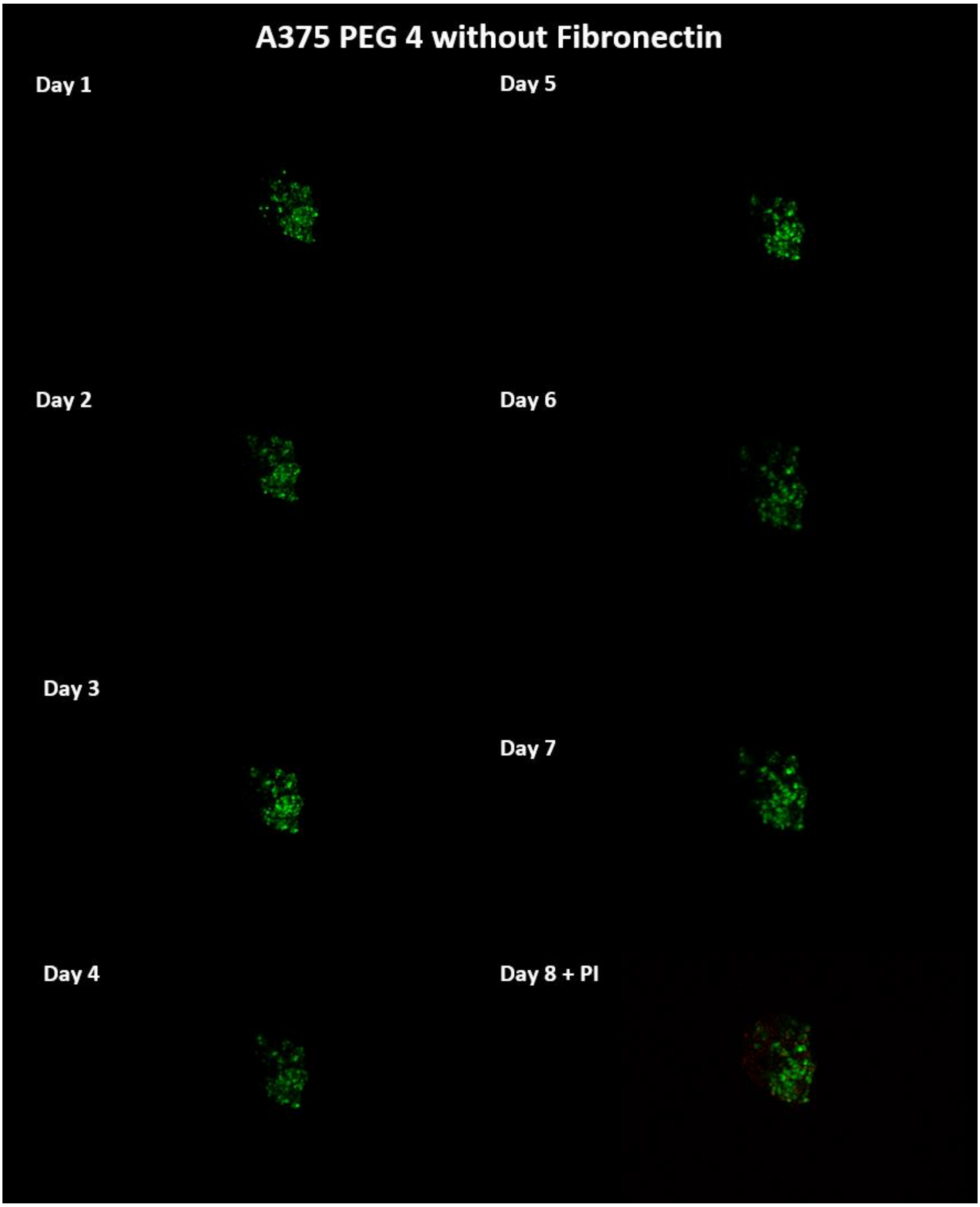
A375 PEG 4 without Fibronectin
Day 1
Day 5
Day 2
Day 6
Day 3
Day 7
Day 4
Day 8 + PI

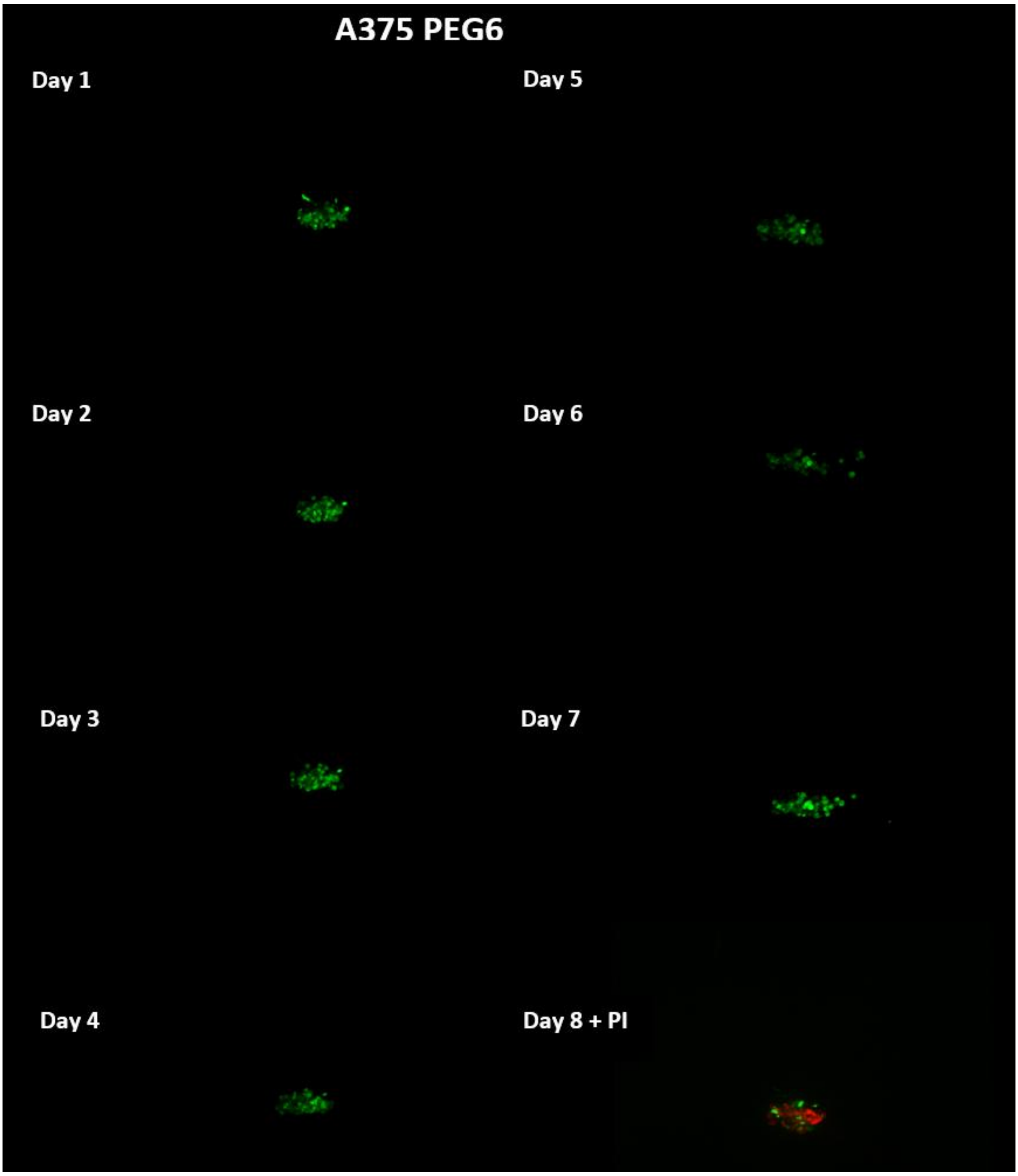
A375 PEG6
Day 1
Day 5
Day 2
Day 6
Day 3
Day 7
Day 4
Day 8 + PI

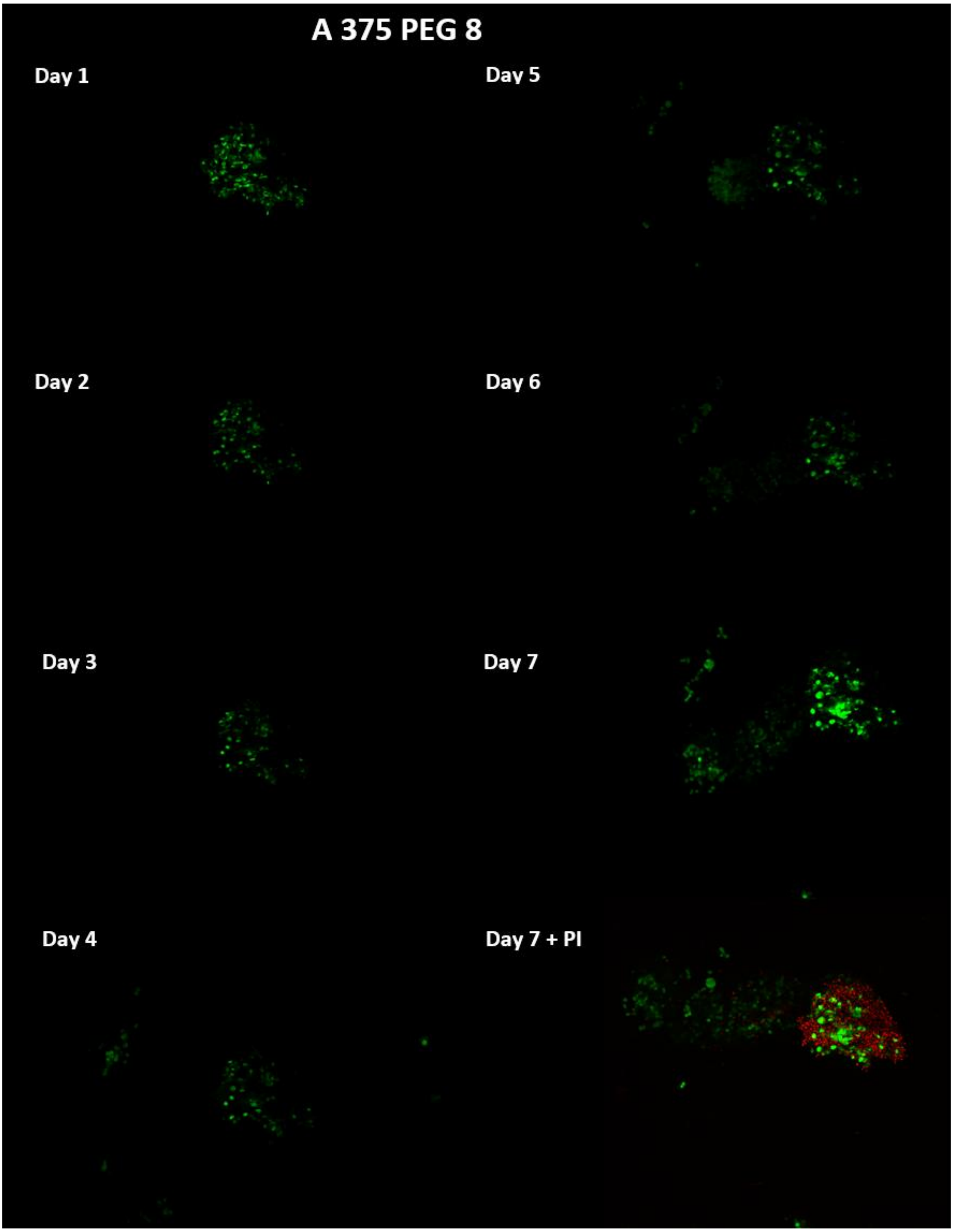
A 375 PEG 8
Day 1
Day 5
Day 2
Day 6
Day 3
Day 7
Day 4
Day 7 + PI

A375– Third Iteration

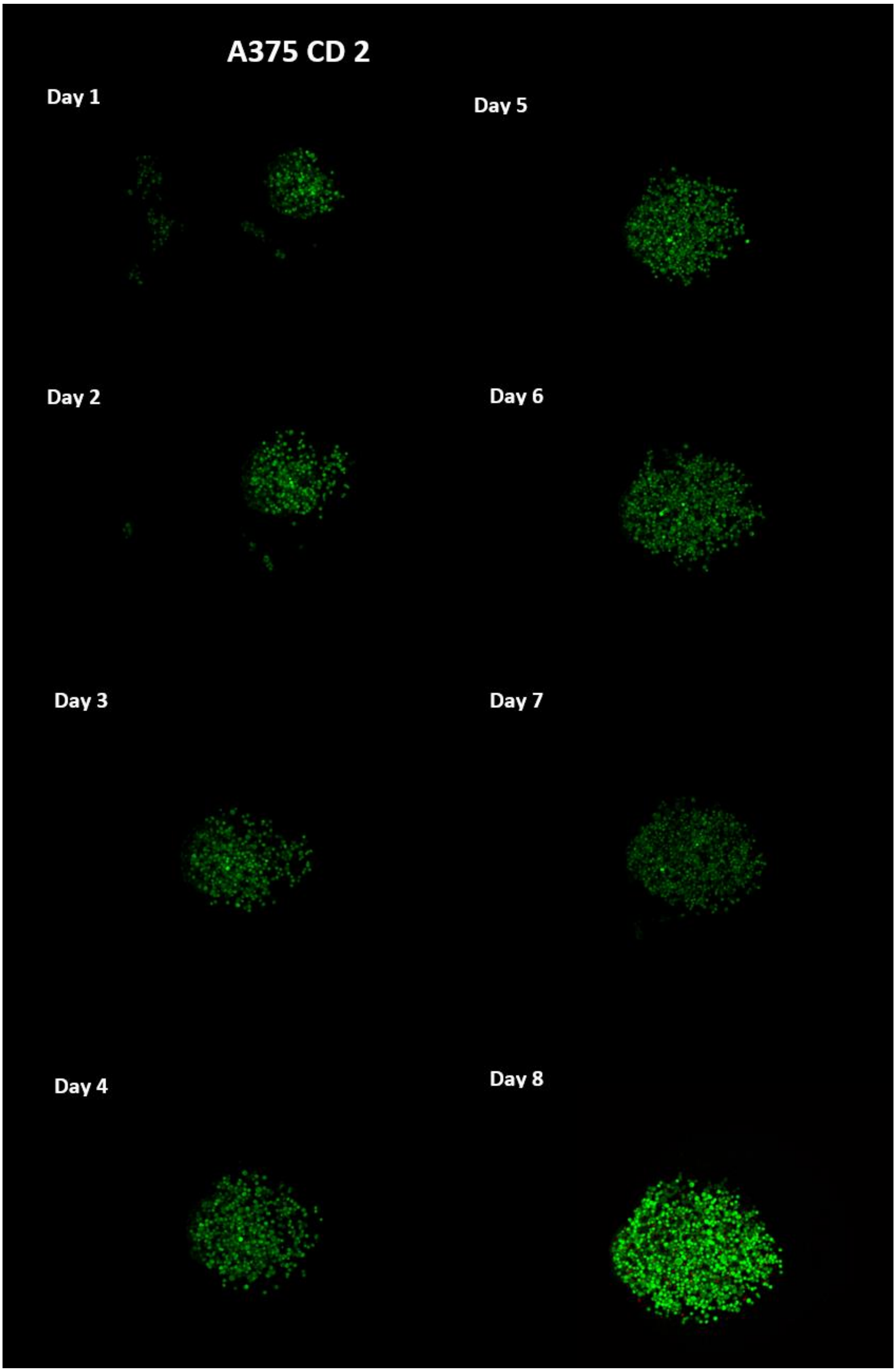

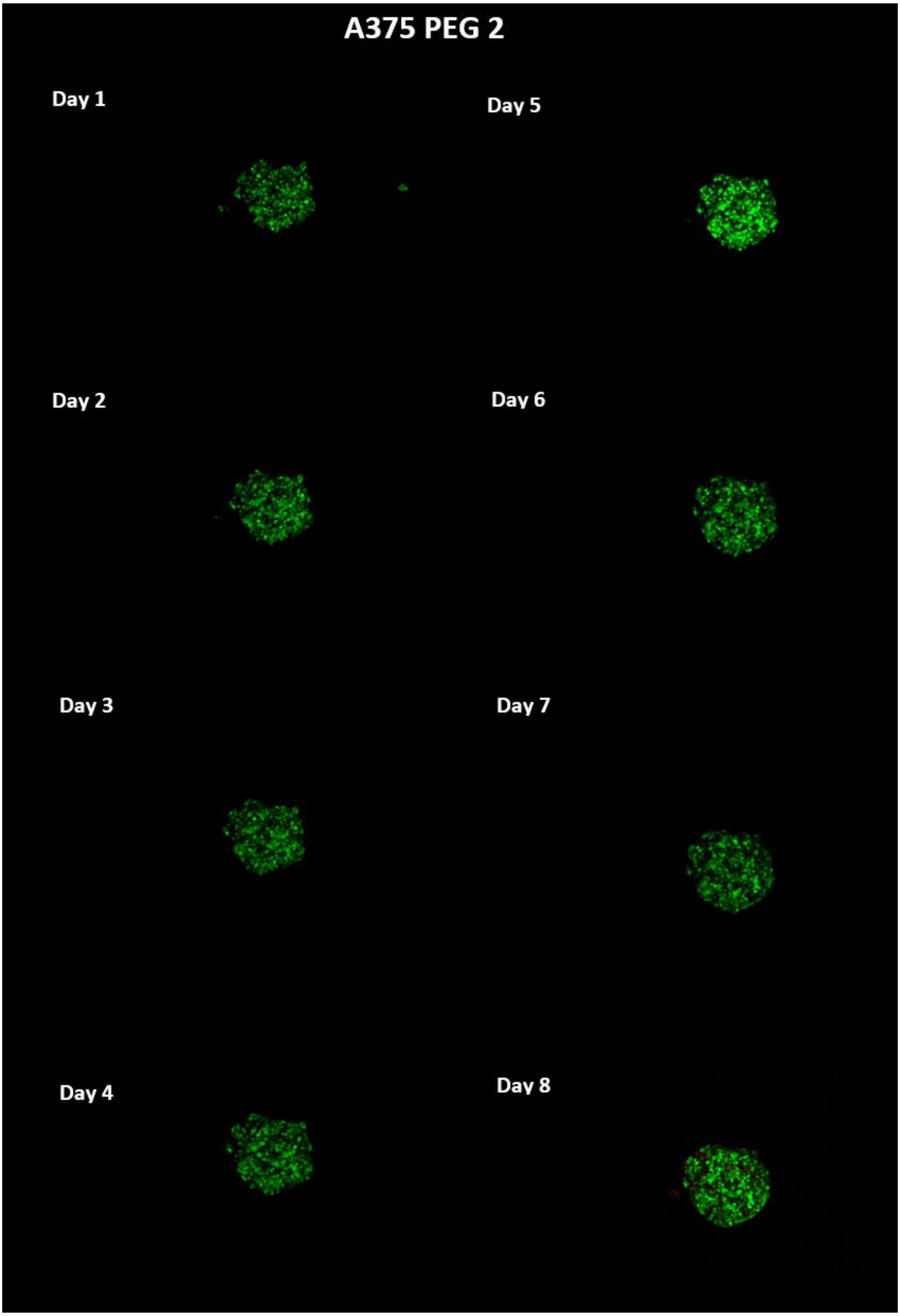
A375 PEG 2
Day 1
Day 5
Day 2
Day 6
Day 3
Day 7
Day 4
Day 8
