# Supplementary material for: Mechanistically Coupled PK (MCPK) Model to Describe Enzyme Induction and Occupancy Dependent DDI of Dabrafenib Metabolism
Source: Pharmaceutics. 2022 Jan 28;14(2):310. doi: 10.3390/pharmaceutics14020310 (PMC8875124; doi:10.3390/pharmaceutics14020310)
Supplement: Supplementary file 1 [file pharmaceutics-14-00310-s001.zip › pharmaceutics-1479084-supplementary/Supplementary_Information/Supplemental_Information_S5.pdf]

Supplemental Information S5: Mechanistically Coupled PK (MCPK) model to describe enzyme induction and occupancy dependent DDI of dabrafenib metabolism

Marco Albrecht[1,2], Yuri Kogan[3], Dagmar Kulms[4], and Thomas Sauter[5]

[1]*Université du Luxembourg; Systems Biology Group; 4367 Belvaux; Luxembourg; Email: marco.albrecht@posteo.eu*
[2]*esqLABS GmbH; 26683 Saterland; Germany; Email: marco.albrecht@esqlabs.com*
[3]*Institute for Medical Biomathematics (IMBM); 60991 Bnei Atarot; Israel; Email: yuri@imbm.com*
[4]*Technical University of Dresden; Experimental Dermatology; 01307 Dresden; Germany; Tel.: +49 (0)351 458-18973 ; Email: dagmar.kulms@uniklinikum-dresden.de*
[5]*Université du Luxembourg; Systems Biology Group; 4367 Belvaux; Luxembourg; Tel.: (+352) 46 66 44 6296; Email: thomas.sauter@uni.lu*

Spheroid Image Data of SKmel2 Spheroid Growth

Spheroid Image Data of SKmel2 Spheroid Growth 1
Legend 1
SKmel2– First Iteration 2

Legend

CD: Gel with cell degradable linker
PEG: Gel with non-degradable polyethylene glycol linker
R1: Biological Replicate 1
SKmel2: Metastatic Melanoma Cell Line with **green** flourecent protein
PI: Propidium iodide as a **red** cell death marker at last day

SKmel2– First Iteration

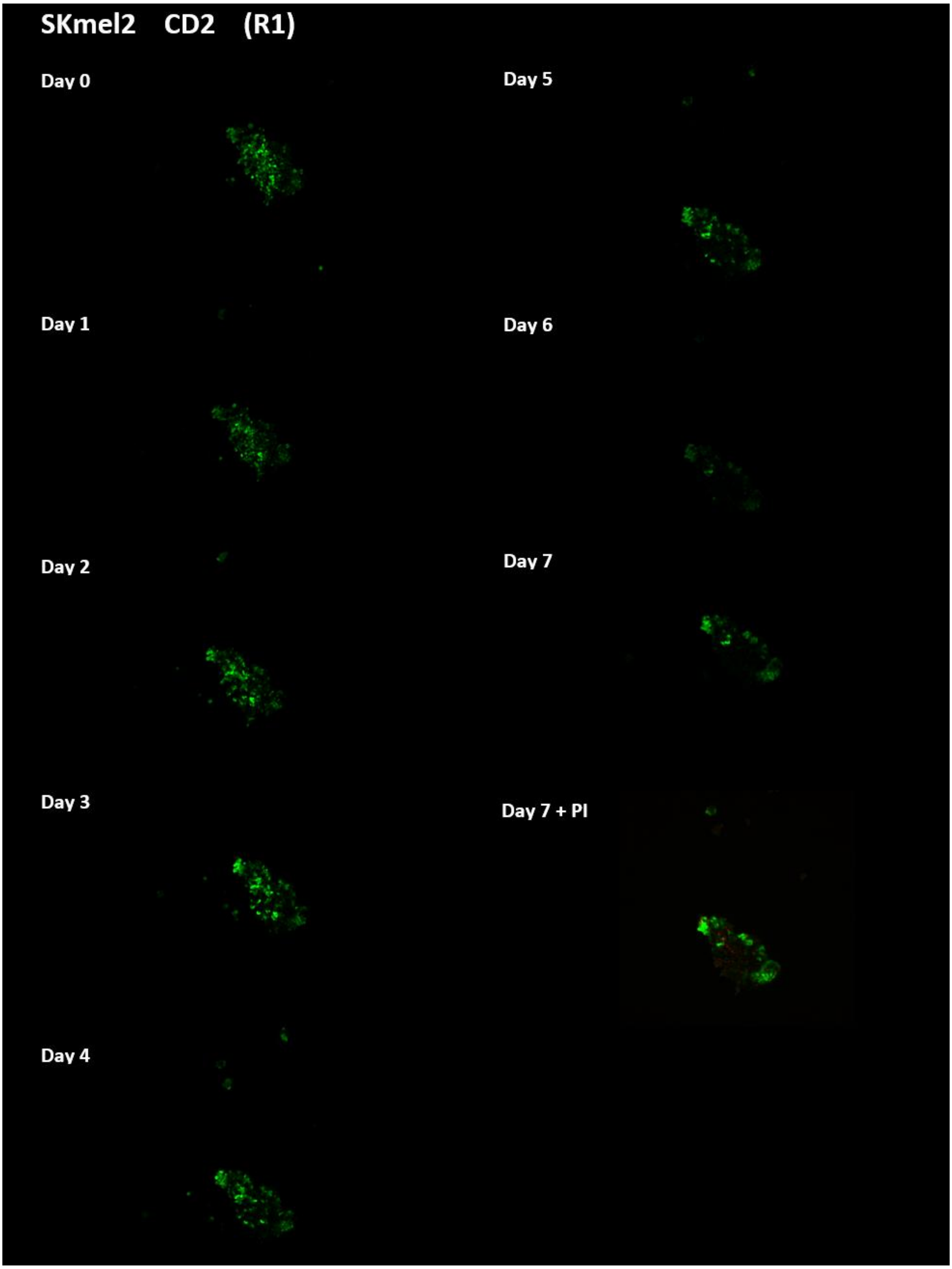

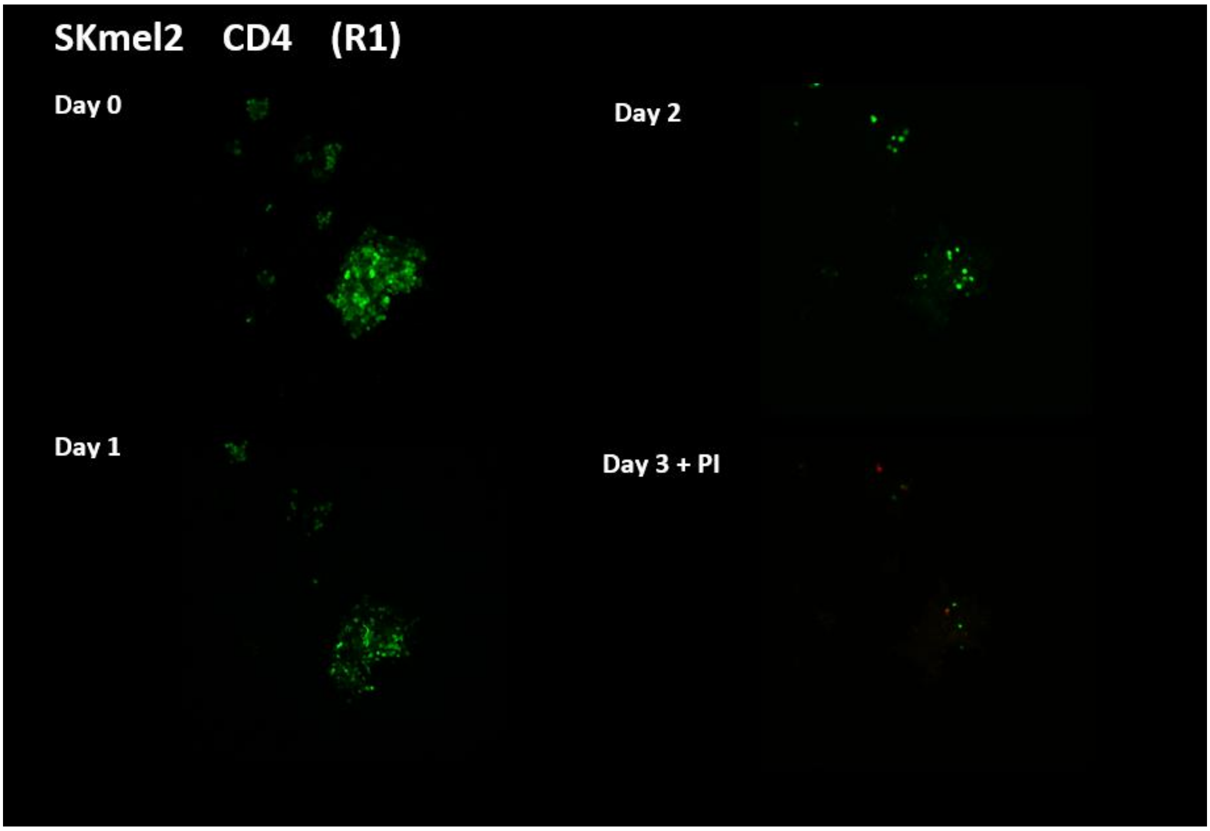
SKmel2 CD4 (R1)
Day 0
Day 2
Day 1
Day 3 + PI

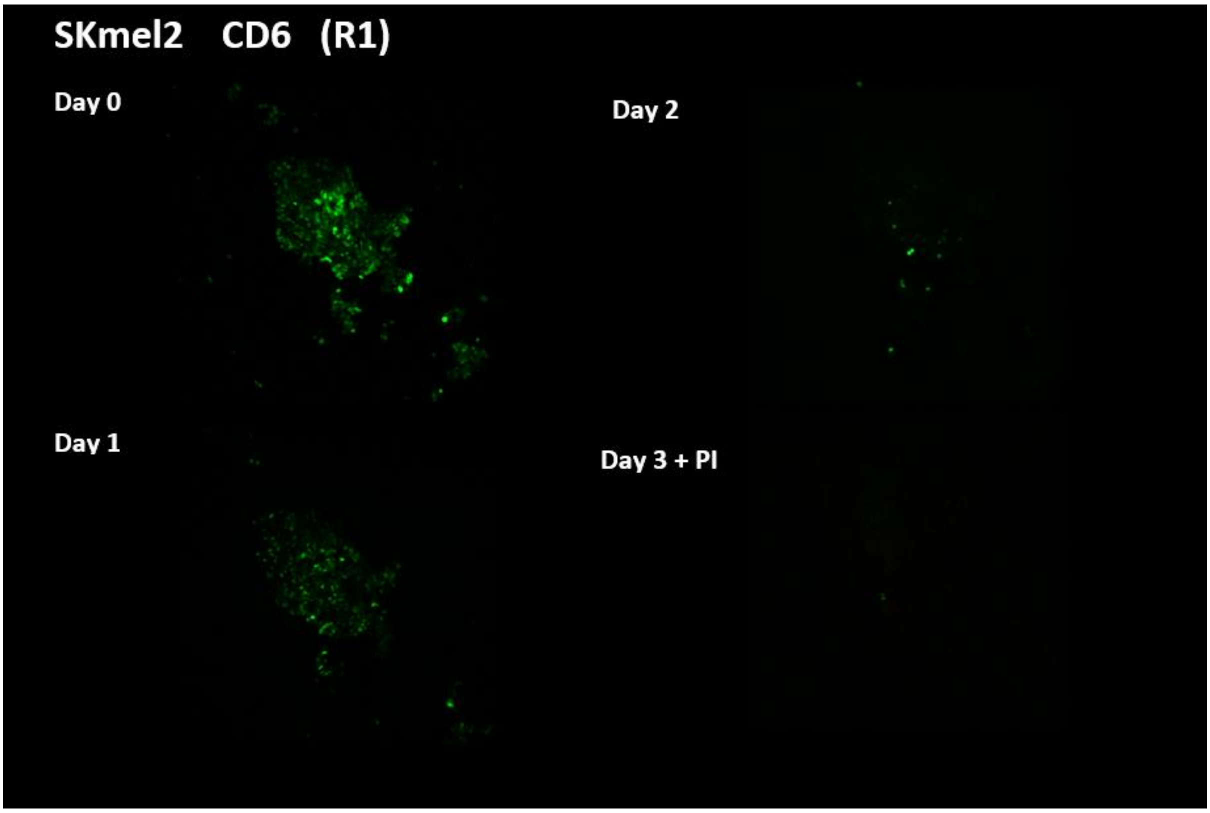
SKmel2 CD6 (R1)
Day 0
Day 2
Day 1
Day 3 + PI

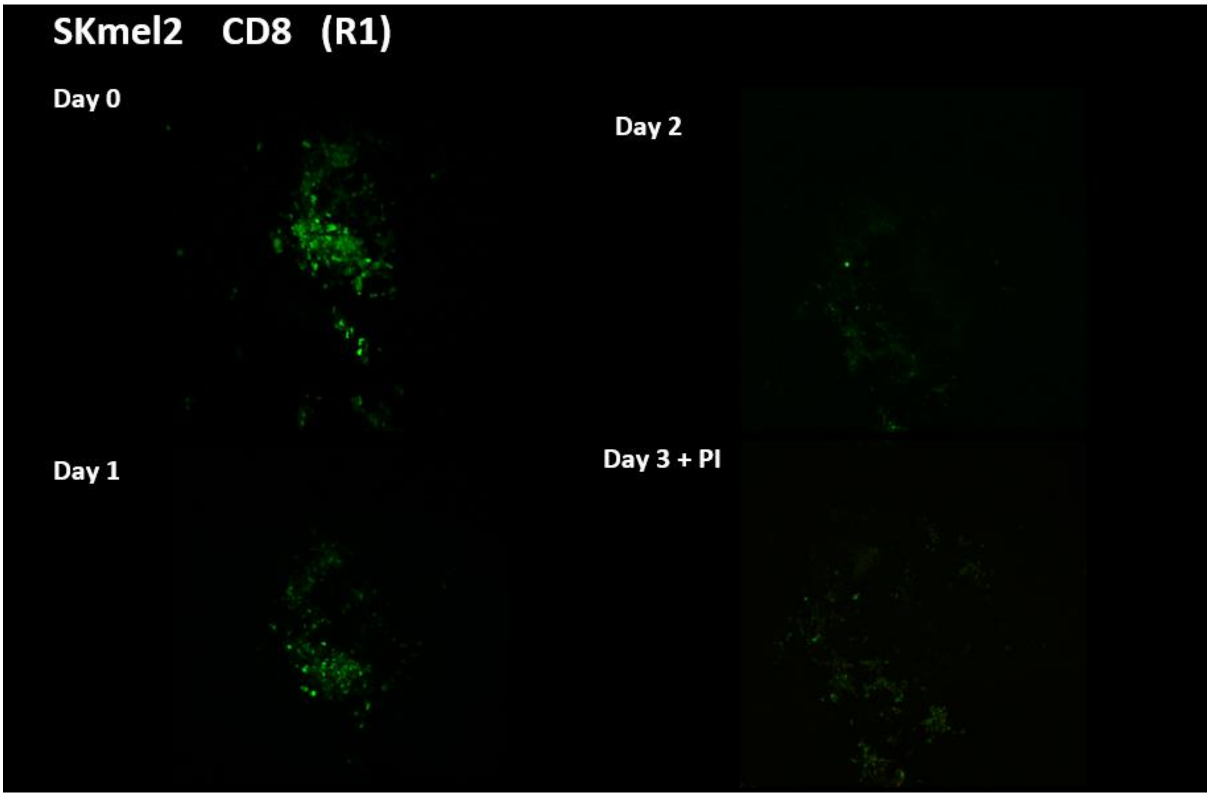
SKmel2 CD8 (R1)
Day 0
Day 2
Day 1
Day 3 + PI

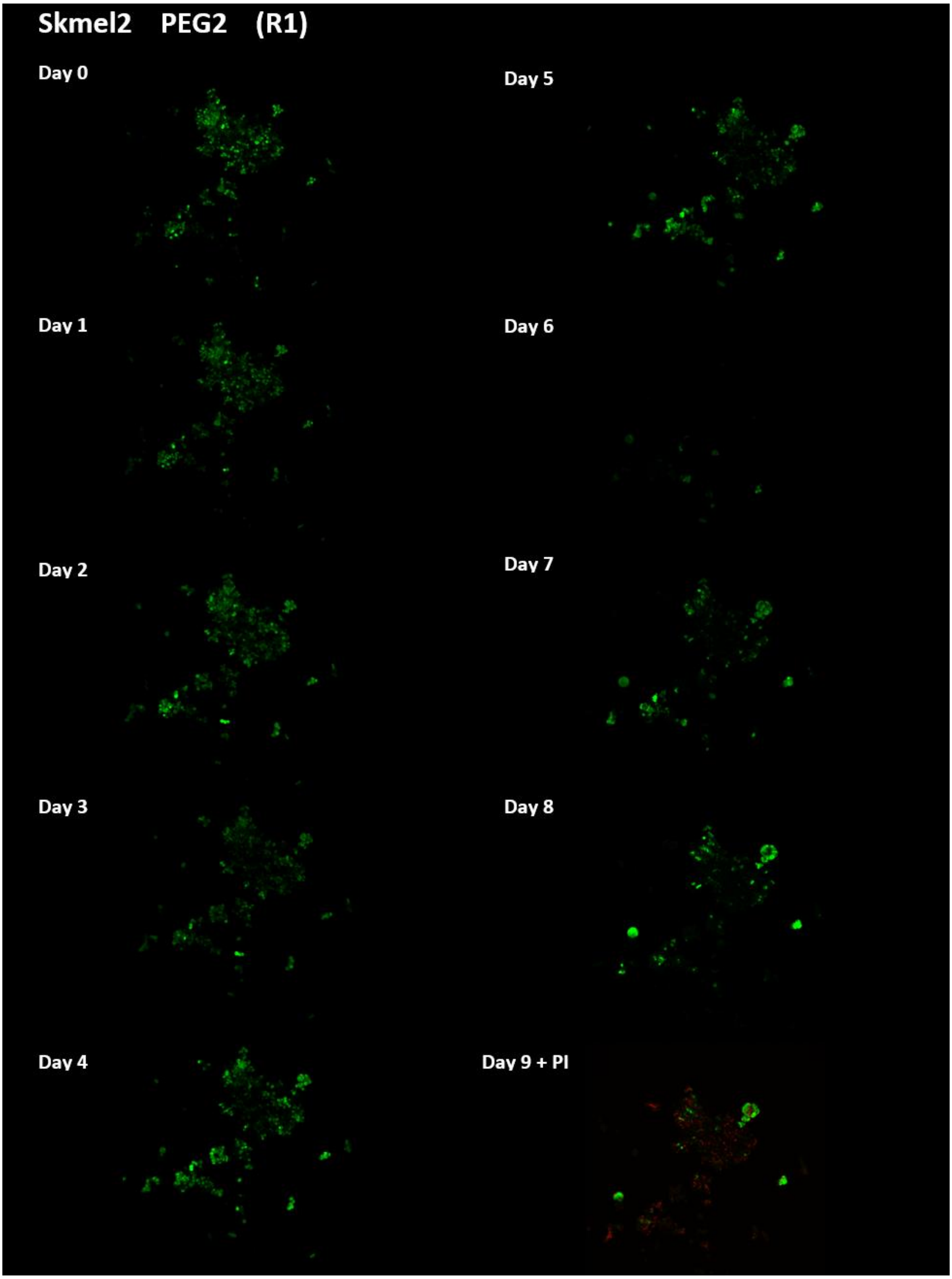
Skmel2 PEG2 (R1)
Day 0
Day 5
Day 1
Day 6
Day 2
Day 7
Day 3
Day 8
Day 4
Day 9 + PI

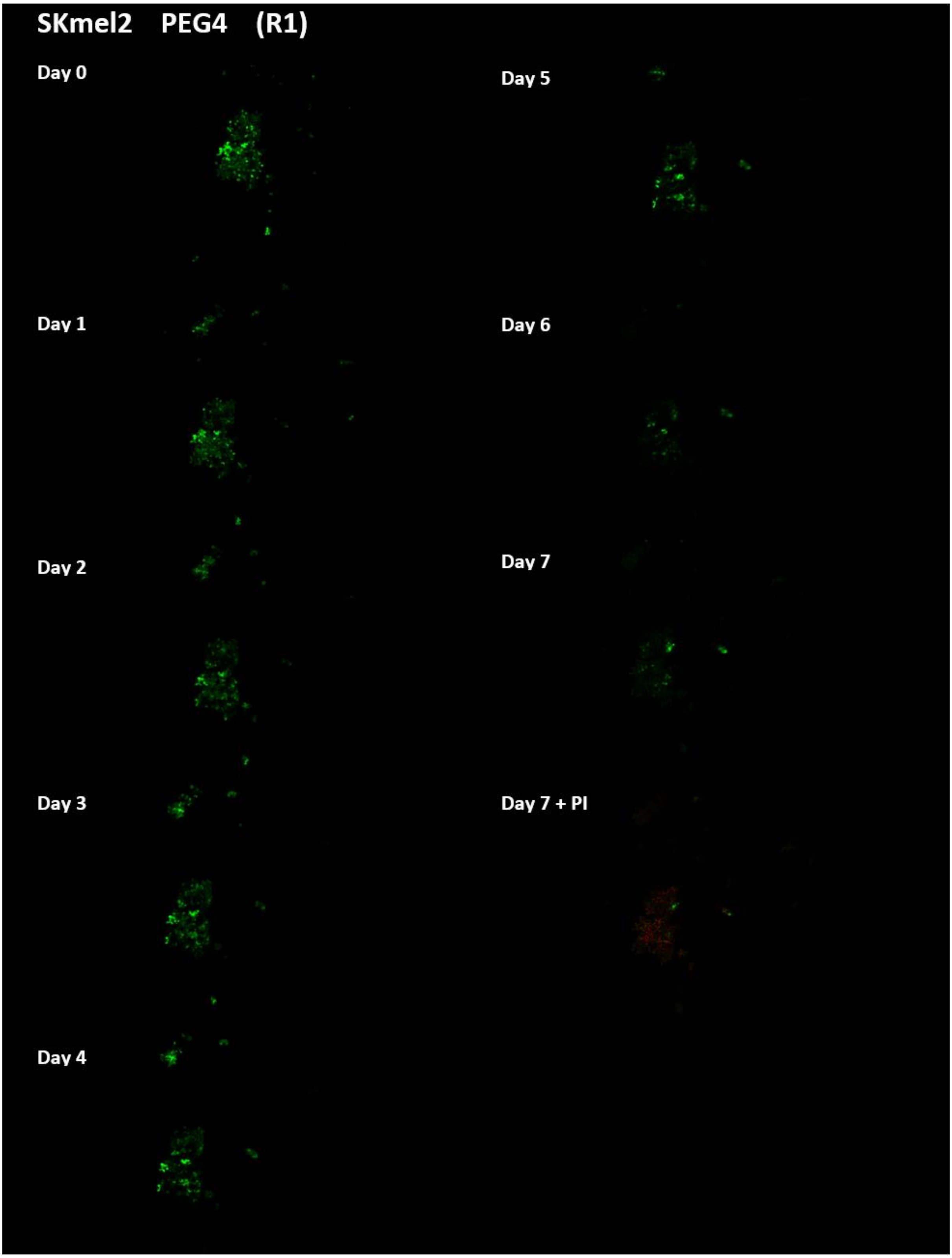
SKmel2 PEG4 (R1)
Day 0
Day 5
Day 1
Day 6
Day 2
Day 7
Day 3
Day 7 + PI
Day 4

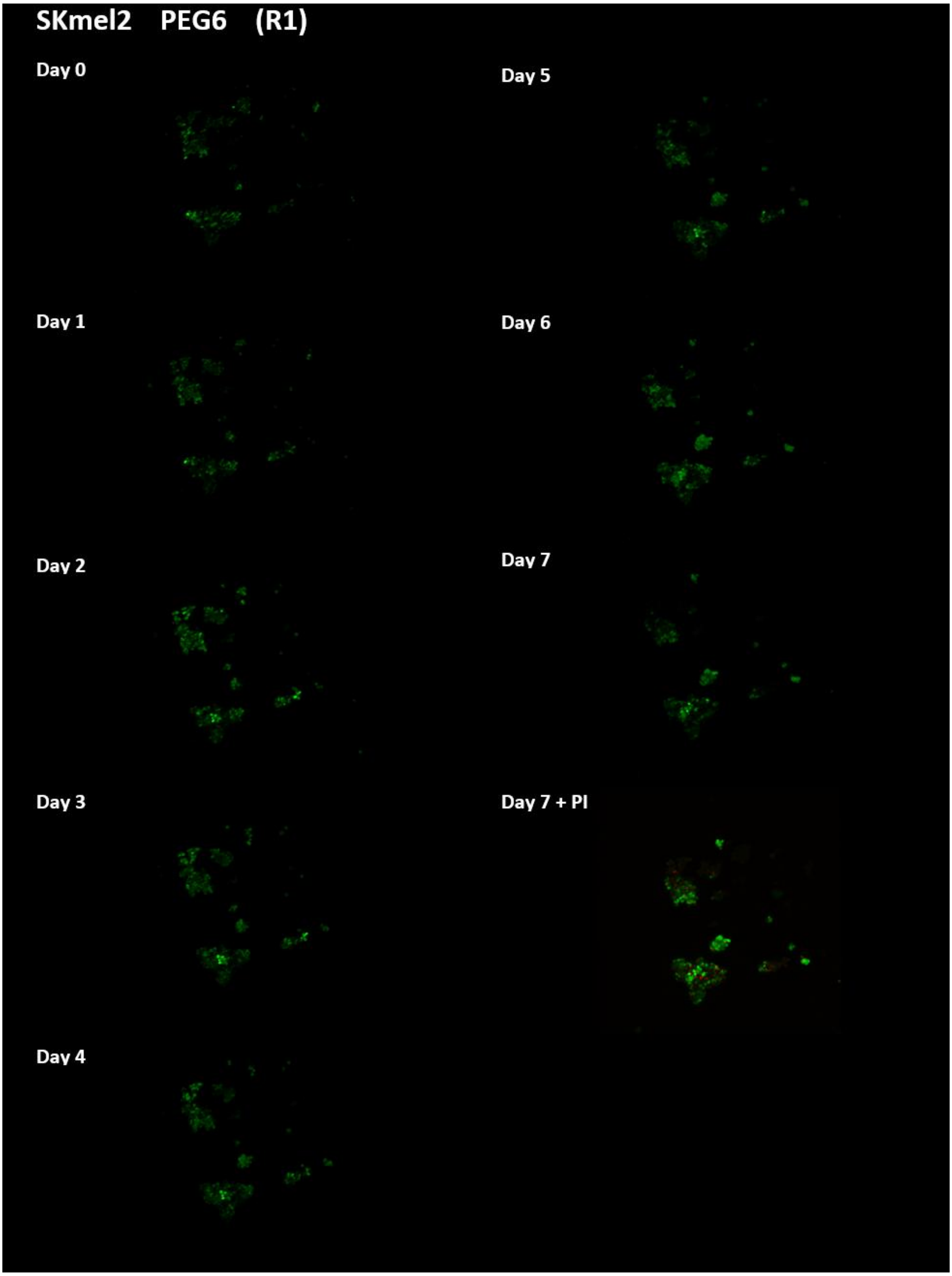
SKmel2 PEG6 (R1)
Day 0
Day 5
Day 1
Day 6
Day 2
Day 7
Day 3
Day 7 + PI
Day 4

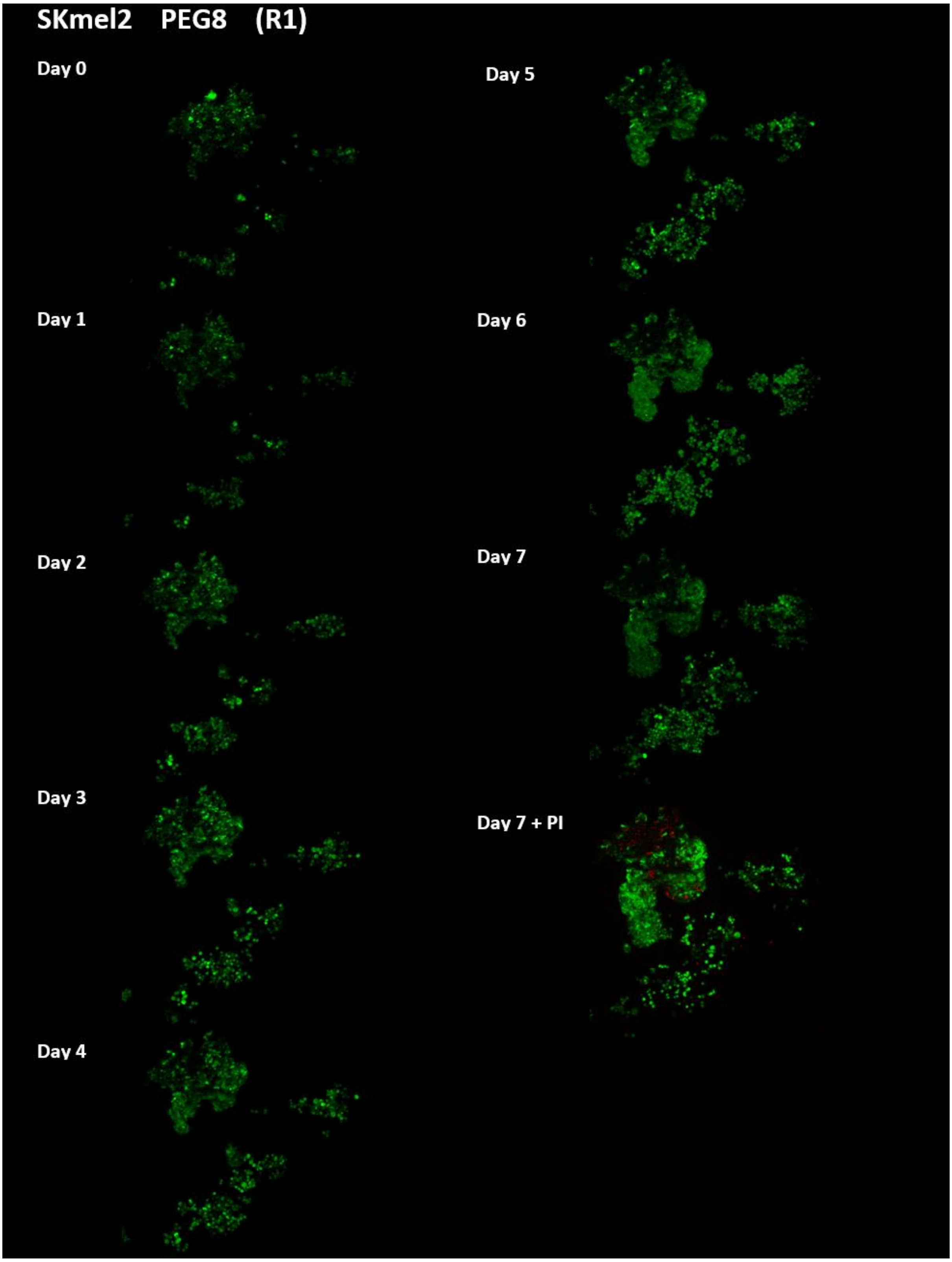
SKmel2 PEG8 (R1)
Day 0
Day 1
Day 2
Day 3
Day 4
Day 5
Day 6
Day 7
Day 7 + PI
